# Ultrasound radiomics based on axillary lymph nodes images for predicting lymph node metastasis in breast cancer

**DOI:** 10.3389/fonc.2023.1217309

**Published:** 2023-10-26

**Authors:** Yu-Long Tang, Bin Wang, Tao Ou-Yang, Wen-Zhi Lv, Shi-Chu Tang, An Wei, Xin-Wu Cui, Jiang-Sheng Huang

**Affiliations:** ^1^ Department of Thyroid Surgery, The Second Xiangya Hospital of Central South University, Changsha, China; ^2^ Department of Medical Ultrasound, Yueyang Central Hospital, Yueyang, China; ^3^ Department of Medical Ultrasound, Hunan Cancer Hospital/The Afliated Cancer Hospital of Xiangya School of Medicine, Central South University, Changsha, China; ^4^ Department of Artificial Intelligence, Julei Technology, Wuhan, China; ^5^ Department of Ultrasound, Hunan Provincial People’s Hospital, Changsha, China; ^6^ Department of Medical Ultrasound, Tongji Hospital, Tongji Medical College, Huazhong University of Science and Technology, Wuhan, China

**Keywords:** radiomics signature, axillary lymph nodes metastasis, breast cancer, ultrasound, prediction

## Abstract

**Objectives:**

To determine whether ultrasound radiomics can be used to distinguish axillary lymph nodes (ALN) metastases in breast cancer based on ALN imaging.

**Methods:**

A total of 147 breast cancer patients with 41 non-metastatic lymph nodes and 109 metastatic lymph nodes were divided into a training set (105 ALN) and a validation set (45 ALN). Radiomics features were extracted from ultrasound images and a radiomics signature (RS) was built. The Intraclass correlation coefficients (ICCs), Spearman correlation analysis, and least absolute shrinkage and selection operator (LASSO) methods were used to select the ALN status–related features. All images were assessed by two radiologists with at least 10 years of experience in ALN ultrasound examination. The performance levels of the model and radiologists in the training and validation subgroups were then evaluated and compared.

**Result:**

Radiomics signature accurately predicted the ALN status, achieved an area under the receiver operator characteristic curve of 0.929 (95%CI, 0.881-0.978) and area under curve(AUC) of 0.919 (95%CI, 95%CI, 0.841-0.997) in training and validation cohorts respectively. The radiomics model performed better than two experts’ prediction of ALN status in both cohorts (P<0.05). Besides, prediction in subgroups based on baseline clinicopathological information also achieved good discrimination performance, with an AUC of 0.937, 0.918, 0.885, 0.930, and 0.913 in HR+/HER2-, HER2+, triple-negative, tumor sized ≤ 3cm and tumor sized>3 cm, respectively.

**Conclusion:**

The radiomics model demonstrated a good ability to predict ALN status in patients with breast cancer, which might provide essential information for decision-making.

## Introduction

Breast cancer (BC) is the most commonly diagnosed cancer among women and accounts for 12.5% of all new annual cancer cases worldwide (1, 2). B etween 30.2–69.8% of BC patients have lymph node metastases (3). Acquisition of the regional lymph node status is necessary to achieve precision therapy and a good prognosis (4). Some studies use clinical and pathological features of breast tumors, such as molecular subtype and maximum lesion diameter to predict axillary lymph node tumor burden (5). However there is no consensus on the point of predicting lymph node metastasis based on clinicalpathological characteristics.

Currently, the main approach to gaining lymph node status is sentinel node biopsy (6). However, this is an invasive method, with potential complications of arm pain, hematoma, seroma, lymphedema, and infection. Clinical trial ACOSOG Z0011 has shown that patients with limited sentinel lymph node metastatic breast cancer who received sentinel lymph node dissection (SLND) alone compared with ALN dissection did not lead to an inferior survival (7). However, the false negative rate of SLND ranges from 7.8% to 27.3%, which cannot be ignored, which is a common problem in patients with risk factors such as upper outer breast cancer and lead to adverse consequences, including incorrect tumor staging and increasing the risk of recurrence (8–10). There is no highly accurate and non-invasive method for the identification of ALN metastases in breast cancer at present.

Preoperative noninvasive ALN assessment methods include axillary ultrasonography (US), magnetic resonance imaging, and mammography. Axillary US can evaluate nodal morphology in real-time and guide fine-needle biopsies. Asian women have higher-density breasts than other ethnic groups (11). Besides, female patients in Asian countries were mainly concentrated in a younger age group (12). Thus, ultrasound (US) has become an effective method for diagnosing breast neoplasm and ALN lesions (13). It can benefit for preoperative evaluation of ALN status and help choose patients with an extremely low possibility of non sentinel lymph node(SLN) metastasis, for whom ALN dissection can be omitted (14). However, ultrasound has several defects, such as the high dependency on radiologists. Unnecessary biopsies may be caused when images were evaluated by inexperienced radiologists, and the diagnostic performance of axillary US was poor in determining the ALN status (15). Therefore, quantitative and non-invasive methods are still needed to predict ALN metastases of breast cancer (16).

With the rapid development of artificial intelligence (AI), AI has been widely used for processing large sets of medical images, including image reconstruction, image segmentation, analysis, and model prediction, leading to a boom in radiomics. Radiomics is the application of bioinformatics methods to extract multiple quantitative imaging features from medical images, which can obtain additional information to predict potential tumor biological behavior. Plenty of studies have shown good performance in using a radiomics approach to improve the accuracy of malignant lesion discrimination and facilitate the classification of tumor types and grades (17). However, in studies using ultrasound radiomics approaches, few analyses were based on ALN images and aimed to illuminate whether radiomics is capable of classifying enlarged axillary lymph nodes.

The aim of our study was to devise a model able to predict the ALN metastatic status based on radiomics features extracted from ultrasound images of ALN in patients with breast cancer.

## Materials and methods

### Patients and clinicopathological information

**Figure 1:**
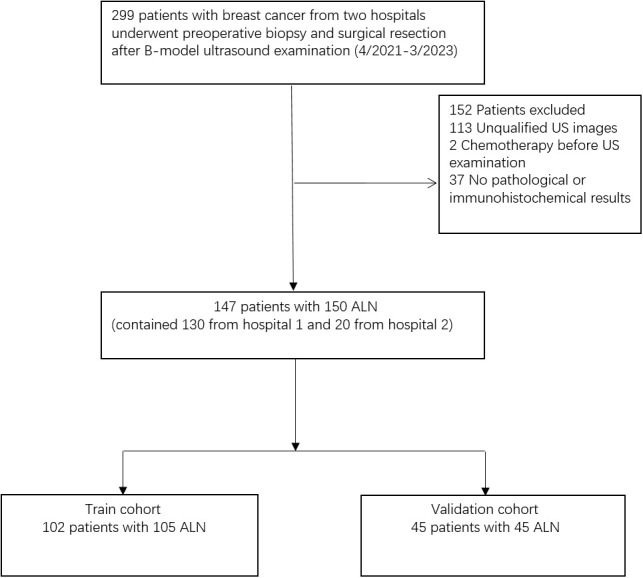
Flow chart of the study population. US, ultrasound; ALN, axillary lymph nodes.

This retrospective study was approved by the ethics committee of the Yueyang Central Hospital and informed consent was waived. Patients with breast cancer in two hospitals were evaluated, 259 and 40 ALN ultrasound images were obtained from Hunan Cancer Hospital (Hospital 1) and Yue Yang Central Hospital (Hospital 2) respectively. These images were produced by ultrasound instrument of Super Sonic Imagine. The flowchart of the study population is shown in **Figure 1**.

The inclusion criteria were as follows: (i) the patient with qualified images; (ii) the patient with complete pathologic information; (iii) the patient with complete baseline characteristics. The exclusion criteria were as follows: (i) the image was blurred or has been artificially marked; (ii) the patient has received chemotherapy before ultrasound examination; (iii) incomplete baseline characteristics.

The baseline clinicopathological information was derived from the patient medical record, including age, tumor size, pathological findings, and immunohistochemical (IHC) results of estrogen receptor (ER), progesterone receptor (PR), and HER2 status. For IHC characteristics, ≥1% of cell staining was considered a positive ER/PR, and <1% of cell staining was considered a negative ER/PR (18). We defined HER2 as positive if the IHC result was +3 or the FISH result was positive, otherwise the HER2 status was considered negative (19).

### US image acquisition

B-mode ultrasound and color Doppler flow images were acquired with a Super Sonic Aixplorer system (Super Sonic Imagine, Aix-en-Provence, France) using a 5-14 MHz linear transducer. The patient was placed in a supine or contralateral-side-down oblique position on the table, with the ipsilateral hand placed behind the head. US scanning typically started from the lower part of the axilla and continued upward toward the axillary fossa. Transverse and sagittal planes were imaged. ALL images in the two hospitals were obtained by two senior radiologists complying with the same protocol, so that can we reduce the deviation caused by different operators.

### Target ALN segmentation and radiomics feature extraction

**Figure 2:**
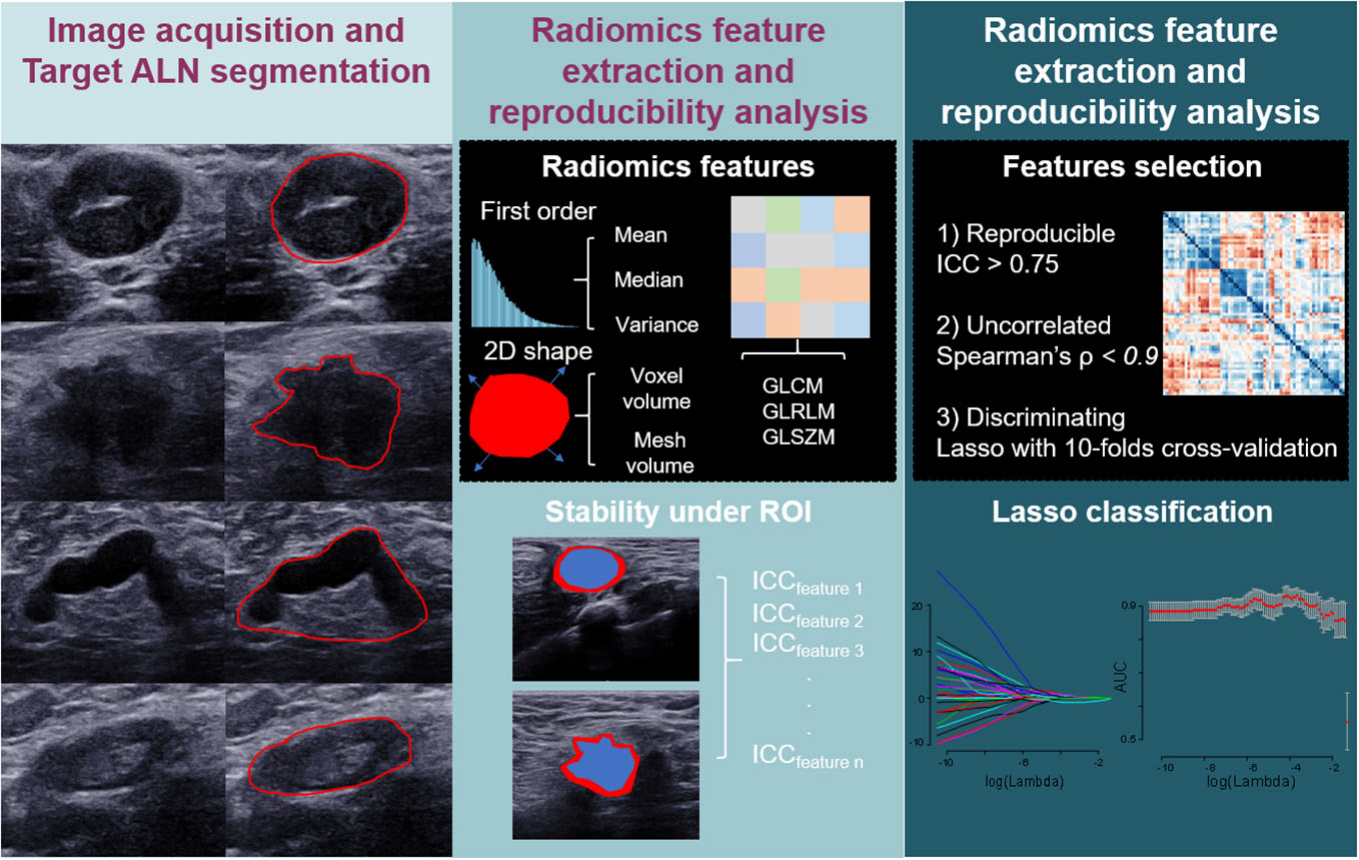
Diagram shows workflow of modeling for ALN status prediction in patients with breast cancer.

150 ALN were eligible for the inclusion criteria, among them 132 lymph nodes were selected corresponding to the ultrasound-guided biopsy images, usually the biggest ipsilateral lymph node. Biopsies of ALN were performed under the guidance of ultrasound by using an 18G core needle. And 18 lymph nodes without preoperative biopsy were considered non-ALN metastasis, because their postoperative pathological results were lymph node negative, indicating there is no metastasis on this side armpit. One US image with the largest diameter of each ALN lesion was used for analysis. The region of interest (ROI) was manually delineated on the US image using ITK-SNAP 3.8 software (http://www.itksnap.org). At the initial stage, the manual segmentations of 150 images were performed by (Y.-L.T.), a breast surgeon who received breast ultrasound training. To evaluate interobserver reliability, all images were manually re-delineated by (T.O-Y.), a senior radiologist with 10 years of US experience. They both finished without knowing the pathological results. The two-dimensional ROI of the ALN was depicted on the ultrasound image and the radiomics features were extracted automatically from each image by using the open-source python package Pyradiomic (https://pyradiomics.readthedocs.io/en/latest/) (20). To evaluate the intraobserver reliability, the ROI segmentation of 50 randomly chosen images in a blind method was performed by (Y.-L.T.) two weeks later. Intraclass correlation coefficients (ICCs) greater than 0.75 indicate good agreement of ALN segmentation (21, 22). The process is presented in **Figure 2**.

### Radiomics model construction and evaluation

Differences between the negative and positive ALN in training and validation cohorts were determined by the Mann-Whitney test (non-normal distribution) or t-test (normal distribution), p<0.05. We used Spearman’s correlation coefficient to evaluate the redundancy of the features, and eliminated features with a Spearman correlation coefficient ≥ 0.9, with only the most reliable one left for further analysis. And supervised learning algorithm was applied to select those most representative features. The least absolute shrinkage and selection operator (LASSO) regression using tenfold cross-validation was applied to select the most predictive ALN status–related features from the training set (23). The formulas for the US radiomics signature were built using the respective selected feature. After that, the radiomics signature (RS) was built to predict the ALN metastasis in breast cancer. The discrimination ability was evaluated using the area under the receiver operator characteristic (AUROC) curve. The optimal cut-off value was calculated with the Youden index. The performance of the optimal cut-off value was assessed by diagnostic sensitivity, specificity, and accuracy. Furthermore, radiomics model performance in subgroups was conducted. The subgroups were set based on baseline clinicopathological information.

### Radiologist evaluation

The US images of ALN were assessed by two radiologists without knowing pathological results (R1:L.Q. and R2:S.-C.T., with 15 and 30 years of experience respectively), based on cortex, morphology, margins, and lymphatic hilum status of lymph nodes (24). US images were reviewed by expert radiologists and binary classification was made (N0 or NX). The areas under the AUROC of the two radiologists was calculated respectively. The diagnostic sensitivity, specificity, and accuracy were calculated. And then we evaluated two radiologist’s performance in subgroups.

### Statistical analysis

The DeLong test was calculated to distinguish the differences between AUCs. Statistical analysis was conducted using SPSS 21 software (SPSS Inc., Chicago, IL). All levels of statistical significance are bilateral, with a P value less than 0.05. In univariate analysis, the differences in clinical characteristics between the patients of different groups were compared using the Mann–Whitney U test for continuous variables, and the χ2 test for categorical variables. The False Discovery Rate was calculated by using the Benjamini-Hochberg method.

## Results

### Patient characteristics

In total, 147 patients with 150 ALN registered from April 2021 to March 2023 in two hospitals were obtained. Training cohort all come from hospital 1(105 ALN, age, 52.55 ± 11.36 years; range, 30-80 years). The validation cohort consists of 25 ALNs from hospital 1 and 20 ALNS from hospital 2 (45 ALN, age, 52.09 ± 10.98 years; range, 32-76 years).

The baseline characteristics of patients and pathological results in the training and validation cohorts are displayed in **Table 1**. There were no significant differences between these two cohorts in age, breast tumor size, status of HR, HER2. Among the total 150 ALN, according to the results of pathological results, 77 and 33 were positive ALN, and 28 and 12 were negative ALN in the training and validation cohorts, respectively. There was no significant difference in ALN status between the two cohorts. Among the 110 metastatic lymph nodes of cancer, 56 had breast tumors larger than 3 cm and 54 had tumors no larger than 3 cm, while among 40 non-metastatic lymph nodes, 15 had breast tumors larger than 3cm and 25 had tumors no larger than 3 cm.

**Table 1:** Clinical characteristics of patients in the training and validation cohorts.

Characteristic	Training (n=105)	Validation (n=45)	*p value*	*FDR*
Age, mean±SD, years	52.55 ± 11.36	52.09 ± 10.98	0.801¶	0.935
ALN metastasis			0.999 §	0.999
NO	28 (26.7)	12 (26.7)		
NX	77 (73.3)	33 (73.3)		
US-reported tumor size(cm)			0.722 §	0.935
≤3cm	54 (51.4)	25 (55.6)		
> 3cm	51 (48.6)	20 (44.4)		
Estrogenic receptor (%)			0.284 §	0.663
Positive	62 (59.0)	22 (48.9)		
Negative	43 (41.0)	23 (51.1)		
Progesterone receptor (%)			0.479 §	0.838
Positive	54 (51.4)	20 (44.4)		
Negative	51 (48.6)	25 (55.6)		
HER2 (%)			0.215 §	0.663
Positive	42 (40.0)	23 (51.1)		
Negative	63 (60.0)	22 (48.9)		
RS, median (interquartile range)	1.77 (0.42-2.61)	1.09 (-0.31-2.55)	0.137¶	0.663

Data expressed as n (%), unless otherwise stated.

US, ultrasound; ALN, axillary lymph node; RS, radiomics signature; FDR, false discovery rate.

¶ By the Mann–Whitney U test.

§ By the Chi-square test.

### Feature selection and construction of radiomics model

Radiomics features were extracted from each US image and a total of 651 imaging features were obtained. A total number of 614 features were thought to be robust (ICC>0.75) and considered in subsequent analysis. Favorable interobserver and intraobserver reproducibility were achieved with these features, with intraobserver ICCs ranging from 0.750 to 0.999 and interobserver ICCs ranging from 0.752 to 0.999. There were 149 features that had no significant difference between the two groups (N0 group and NX group) were reduced. After eliminating redundant features by Spearman correlation analysis, we got 66 features. Finally, nine ALN status–related features were selected by LASSO regression with 10-fold cross-validation. Nine features were represented by letters A to I, details are shown in **Table 2**. The US radiomics signature calculation formulas are presented as follows: Rad-score = 1.2918+(-0.9559 × A) + (0.0890 × B) + (0.1083 × C) + (0.1388 × D) + (0.2123 × E) + (0.2238 × F) + (0.2699 × G) + (0.3875 × H) + (0.3907 × I).

**Table 2:** Radiomic features selection result.

Variables	Radiomics feature name	Image type	Feature type	Coefficients
A	Logarithm_ngtdm_Strength	Logarithm	Ngtdm	-0.9559
B	Exponential_glszm_SizeZoneNonUniformity	Exponential	Glszm	0.0890
C	Logarithm_firstorder_Skewness	Logarithm	Firstorder	0.1083
D	Wavelet.H_glszm_SmallAreaHighGrayLevelEmphasis	Wavelet.H	Glszm	0.1388
E	Original_glcm_Idn	Original	Glcm	0.2123
F	Square_glcm_Idmn	Square	Glcm	0.2238
G	Wavelet.L_gldm_SmallDependenceLowGrayLevelEmphasis	Wavelet.L	Gldm	0.2699
H	Squareroot_glszm_SmalAreaEmphasis	Squareroot	Glszm	0.3875
I	Squareroot_glszm_SizeZoneNonUniformity	Squareroot	Glszm	0.3907

### Model validation

**Figure 3:**
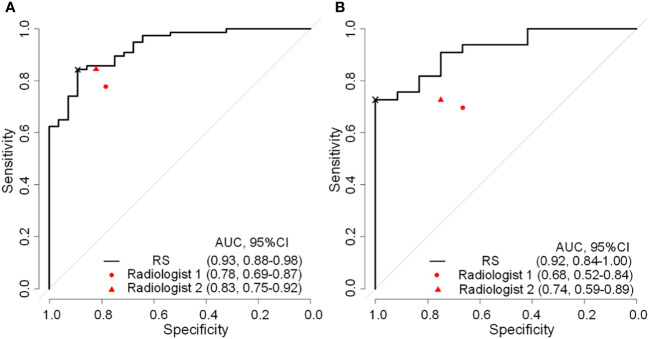
Reciever operating characteristic curves of radiomics signature and expert points of two radiologist in the training **(A)** and validation **(B)** cohorts. RS, radiomatics signature; AUC, area under the receiver operator characteristic curve; ×, cutoff point.

As shown in **Table 3**, there was a significant statistical difference in radiomics signature between N0 and NX ALN in the training group (*p*<0.001) and validation group (*p*<0.001). As shown in **Figure 3**, the radiomics signature achieved an AUC of 0.929 (95%CI, 0.881-0.978) and AUC of 0.919 (95%CI, 0.841-0.997) in training and validation cohorts respectively. Meanwhile, the ROC curves of two radiologists (R1 and R2) were drawn for comparison, R1 achieved the AUC of 0.782 (95%CI, 0.692-0.873) and 0.682 (95%CI, 0.521-0.842) in the training and validation group respectively, R2 achieved the AUC of 0.833 (95%CI, 0.750-0.916) and 0.738 (95%CI, 0.589-0.888) in the training and validation group respectively. Based on the Youden index, the threshold of the total points to predict ALN status was determined to be 0.902. As shown in **Table 4**, The radiomics model achieved an accuracy, sensitivity, and specificity of 85.71%, 84.42%, and 89.29%, respectively, for the training group, and 80.00%, 72.73%, and 100%, respectively, for the validation group. The accuracy, sensitivity, and specificity of R1 were 78.10%, 77.92%, and 78.57%, respectively, in the training group, 68.89%, 69.70%, and 67.67%, respectively, in the validation group. The accuracy, sensitivity, and specificity of R2 were 83.81%, 84.42%, and 82.14%, respectively, in the training group, 73.33%, 72.73%, and 75.00%, respectively, in the validation group. DeLong’s test for two correlated ROC curves was conducted, and the radiomics model performed better than the two experts’ prediction of ALN status in both cohorts (P<0.05).

**Table 3:** Clinical characteristics in training and validation cohort between N0 and NX.

Characteristic	Training cohort (105)				Validation cohort (45)			
	N0 (28)	NX (77)	*p*	*FDR*	N0 (13)	NX (32)	*p*	*FDR*
Age, mean ± SD, years	51.82 ± 8.87	52.82 ± 11.90	0.775¶	0.775	53.08 ± 10.499	51.688 ± 11.31	0.890¶	0.890
US-reported tumor size			0.049 §	0.147			0.883 §	0.890
≤ 3cm	9 (32.1)	35 (45.4)			7 (53.85)	18 (56.25)		
> 3cm	19 (67.9)	42 (54.5)			6 (46.15)	14 (43.75)		
Estrogenic receptor (%)			0.370 §	0.618			0.815§	0.890
Positive	19 (67.9)	43 (55.8)			6 (46.15)	16 (50.00)		
Negative	9 (32.1)	34 (44.2)			7 (53.85)	16 (50.00)		
Progesterone receptor (%)			0.515 §	0.618			0.419§	0.838
Positive	16 (57.1)	38 (49.4)			7 (53.85)	13 (40.63)		
Negative	12 (42.9)	39 (50.6)			6 (46.15)	19 (59.37)		
HER2 (%)			0.501 §	0.618			0.279§	0.837
Positive	13 (46.4)	29 (37.7)			5 (38.46)	18 (56.25)		
Negative	15 (53.6)	48 (62.3)			8 (61.54)	14 (43.75)		
			<0.001¶	0.006			<0.001¶	0.006
RS, median (interquartile range)	-0.36 (-1.66-0.58)	2.26 (1.31-2.85)			-0.56 (-2.28-0.42)	1.75 (0.61-2.8)		

Data expressed as n (%), unless otherwise stated.

US, ultrasound; RS, radiomics signature; FDR, false discovery rate.

¶ By the Mann–Whitney U test.

§ By the χ2 test.

**Table 4:** Performance of the radiomics model and two radiologists for predicting ALN status in training and validation groups.

Index	Training cohort	Validation cohort
Radiomics model		
TP	65	24
TN	25	12
FP	3	0
FN	12	9
Accuracy, %	85.71 (90/105)	80.00 (36/45)
Sensitivity, %	84.42 (65/77)	72.73 (24/33)
Specificity, %	89.29 (25/28)	100.00 (12/12)
Radiologist 1		
TP	60	23
TN	22	8
FP	6	4
FN	17	10
Accuracy, %	78.10 (82/105)	68.89 (31/45)
Sensitivity, %	77.92 (60/77)	69.70 (23/33)
Specificity, %	78.57 (22/28)	66.67 (8/12)
Radiologist 2		
TP	65	24
TN	23	9
FP	5	9
FN	12	3
Accuracy, %	83.81 (88/105)	73.33 (33/45)
Sensitivity, %	84.42 (65/77)	72.72 (24/33)
Specificity, %	82.14 (23/28)	75.00 (9/12)

TP, True Positive; TN, True Negative; FP, False Positive; FN, False Negative.

**Figure 4:**
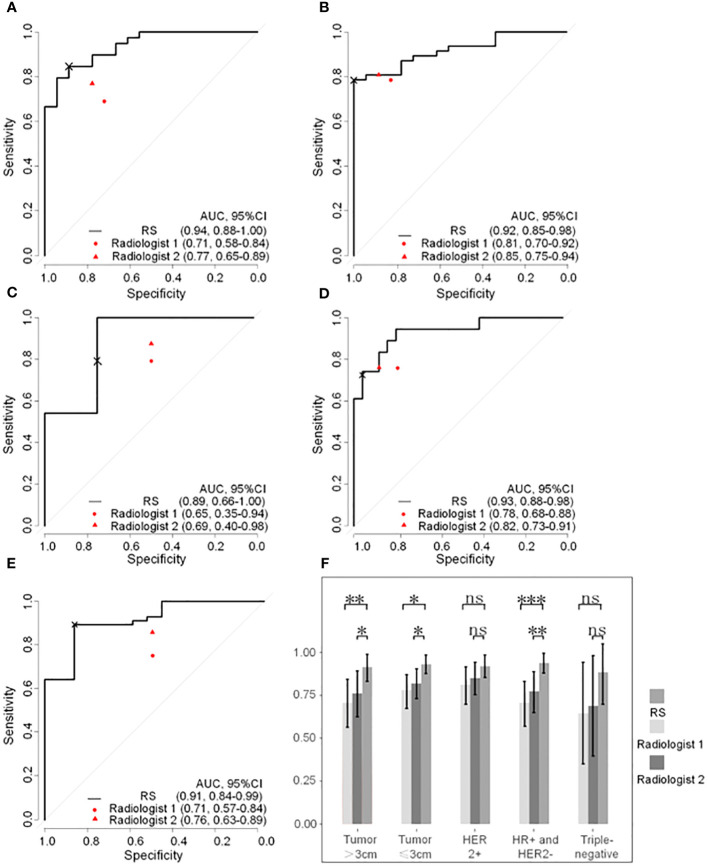
Reciever operating characteristic curves of radiomatics signature and expert points of two radiologists in subgroups based on breast cancer molecular subtypes and tumor size. **(A)** HR+ and HER2- subgroup. **(B)** HER2+ subgroup. **(C)** Triple-negative subgroup. **(D)** tumor size ≤3cm subgroup. **(E)** tumor size >3cm subgroup. **(F)** Column chart of DeLongohort’s test fot ROC curves between radiomatics signatures and two radiologists. HR, hormone receptor, HER2, human epidermal growth factor receptor 2; RS, radiomatoics signature; AUC, area under the receiver operator characteric curve; ns, nonsense; *, p < 0.05; **, p <0.01; ***, p <0.001; ×, cutoff point.

As shown in **Figure 4**, prediction in the HR+/HER2-, HER2+, triple-negative, and tumor sized ≤ 3cm and tumor sized>3 cm subgroups achieved discrimination performance in the whole group, yielding an AUC of 0.937 (95%CI, 0.879-0.995), 0.918 (95%CI, 0.855-0.982), 0.885 (95%CI, 0.655-0.999), 0.930 (95%CI, 0.876-0.985) and 0.913 (95%CI, 0.840-0.986) respectively. The evaluation of R1 achieved an AUC of 0.707 (95%CI, 0.578-0.837), 0.810 (95%CI, 0.704-0.917), 0.646 (95%CI, 0.351-0.941), 0.780 (95%CI, 0.681-0.878) and 0.708 (95%CI, 0.572-0.844) in the HR+/HER2-, HER2+, triple-negative, tumor sized ≤ 3cm and tumor sized>3 cm subgroups respectively. The evaluation of R2 achieved an AUC of 0.773 (95%CI, 0.654-0.893), 0.849 (95%CI, 0.755-0.943), 0.687 (95%CI, 0.397-0.978), 0.819(95%CI, 0.732-0.906) and 0.762 (95%CI, 0.630-0.893) in the HR+/HER2-, HER2+, triple-negative and tumor sized ≤ 3cm and tumor sized>3 cm subgroups respectively. The detailed comparison of statistical results is shown in **Table 5**.

**Table 5:** Performance of the radiomics model and two radiologists for predicting ALN status in subgroups.

Index	HR+/HER2-	HER2	Triple-negative	≤3cm	>3cm
Radiomics model					
TP	33	37	19	39	50
TN	17	18	3	25	13
FP	2	0	1	1	2
FN	5	10	5	6	
Accuracy, %	87.72 (50/57)	84.62 (55/65)	78.57 (22/28)	81.01 (64/79)	88.73 (63/71)
Sensitivity, %	86.84 (33/38)	78.72 (37/47)	79.17 (19/24)	73.58 (39/53)	89.29 (50/56)
Specificity, %	89.47 (17/19)	100.00 (18/18)	75.00 (3/4)	96.15 (25/26)	86.67 (13/15)
Radiologist 1					
TP	26	37	19	40	42
TN	13	15	2	20	10
FP	6	3	2	6	5
FN	12	10	5	13	14
Accuracy, %	68.42 (39/57)	80.00 (52/65)	75.00 (21/28)	75.95 (60/79)	73.24 (52/71)
Sensitivity, %	68.42 (26/38)	78.72 (37/47)	79.17 (19/24)	75.47 (40/53)	75.00 (42/56)
Specificity, %	68.42 (13/19)	83.33 (15/18)	50.00 (2/4)	76.92 (20/26)	66.67 (10/15)
Radiologist 2					
TP	29	38	21	41	48
TN	14	16	2	22	10
FP	5	2	2	3	5
FN	9	9	3	13	8
Accuracy, %	83.81 (43/57)	73.33 (54/65)	82.14 (23/28)	79.75 (63/79)	81.69 (58/71)
Sensitivity, %	84.42 (29/38)	72.72 (38/47)	87.50 (21/24)	77.36 (41/53)	85.71 (48/56)
Specificity, %	82.14 (14/19)	75.00 (16/18)	50.00 (2/4)	84.62 (22/26)	66.67 (10/15)

TP, True Positive; TN, True Negative; FP, False Positive; FN, False Negative.

## Discussion

In this study, we constructed and validated a model based on features derived from US images of ALN for the prediction of ALN status in breast cancer patients. This method is convenient and easy to conduct, which might help in making precise decisions for each patient.

Ultrasound is a common method to evaluate lymph node involvement in breast cancer patients. The sensitivity was reported between 49% to 87%, while the specificity was between 55% to 97% (25). In this study, both shape and intensity features were extracted from images, and the feature selection method removed all shape features. Radiologits mainly relied on the shape of lesions, while our selected features are based on intensity which might be ignored during the daily clinical operation. The performances of radiologists from two different hospitals were less effective than our model, which indicated that our radiomics signature performed better than the routine US-guide ALN examination.

Previous studies indicated that the same model could have different efficiencies among the different molecular subtypes in patients with breast cancer. M L G Vane et al. found a significant difference in negative predictive value (NPV) between triple-negative tumors and HER2+ tumors and between HER2+ and ER/PR+HER2- tumors in the axillary US examination (26). Jie Fei et al. found the ultrasound performance in the triple-negative subtype had the lowest positive predictive value for ALN status (73.2%) (27). Our model achieved good performance in the HR+/HER2-, HER2+, and triple-negative subgroups.

There is no uniform standard among studies related to clinicopathological factors, which might serve as an independent risk factor for the prediction of ALN status in breast cancer. M P Budzik et al. found the hormone receptor status and HER2 expression seemed to be related to the regional lymph node involvement (pN0-pN4) of malignant tumors (28). Illyes et al. found that primary tumors sized greater than 20 mm were significantly associated with a higher incidence of SLN metastasis (p<0.001), while primary tumors sized greater than 26 mm were associated with additional positive non-SLN (p>0.001) (29). In our study, no clinicopathological indicators were used to build a prediction model. In univariate analysis, tumor size greater than 30mm was associated with SLN metastasis in the training group, but the difference was not significant in the multivariate analysis, this was consistent with Nicla La Verde‘s study (30).

Despite plenty of studies using US parameters or image features of breast lesions to predict ALN status (31), most of them developed prediction models using radiomics based on images of breast lesions, while few of them concentrated on imaging features of ALN (32–34). Our prediction model has achieved a good diagnostic performance by using the radiomics signature derived from the US image of lymph nodes, which could be considered as an evaluation indicator when surgeons make plans specific to a patient’s situation.

Our study has some limitations. Firstly. it is a retrospective study that collected data from only two hospitals, a small number of patients and lymph nodes were selected. Secondly, There were three patients, for them the characteristics of bilateral lymph nodes in the same patient were considered independent. We believe patients can have both normal and metastatic lymph nodes or the tumor heterogeneity could happen in one patient. Still, it is possible to cause some potential bias. Besides, our research is focused on qualitative analysis based on US images of ALN, not quantitative analysis of ALN metastasis burden in breast cancer. Therefore, Multicenter studies incorporating more patients should be considered in future research and we should strive to advance qualitative research to quantitative research.

In summary, we built a model based on ALN images to predict ALN status in breast cancer, which might provide vital information for precise diagnosis and treatment based on ordinary examination. It also should be noted that a higher level of evidence is required before any breast surgery recommendation could be entirely based on it.

## Data availability statement

The original contributions presented in the study are included in the article/**Supplementary Material**. Further inquiries can be directed to the corresponding authors.

## Ethics statement

The studies involving humans were approved by Ethics Committee of Yueyang Central Hospital and Ethics Committee of Hunan Cancer Hospital. The studies were conducted in accordance with the local legislation and institutional requirements. The participants provided their written informed consent to participate in this study.

## Author contributions

Y-LT, J-SH, and X-WC conceived and designed this research. The data collection was finished by Y-LT, BW, and AW. ROI segmentation was completed by Y-LT and TO-Y. W-ZL performed radiomics signature extraction. S-CT finished the subjective evaluation of images. W-ZL provided computational analysis. Y-LT and W-ZL prepared the figures and tables. Y-LT drafted the manuscript. All authors contributed to the article and approved the submitted version.
