# Supplementary figures and images for: Ultrasound radiomics based on axillary lymph nodes images for predicting lymph node metastasis in breast cancer (part 2 of 2)
Source: Front Oncol. 2023 Oct 26;13:1217309. doi: 10.3389/fonc.2023.1217309 (PMC10641324; doi:10.3389/fonc.2023.1217309)

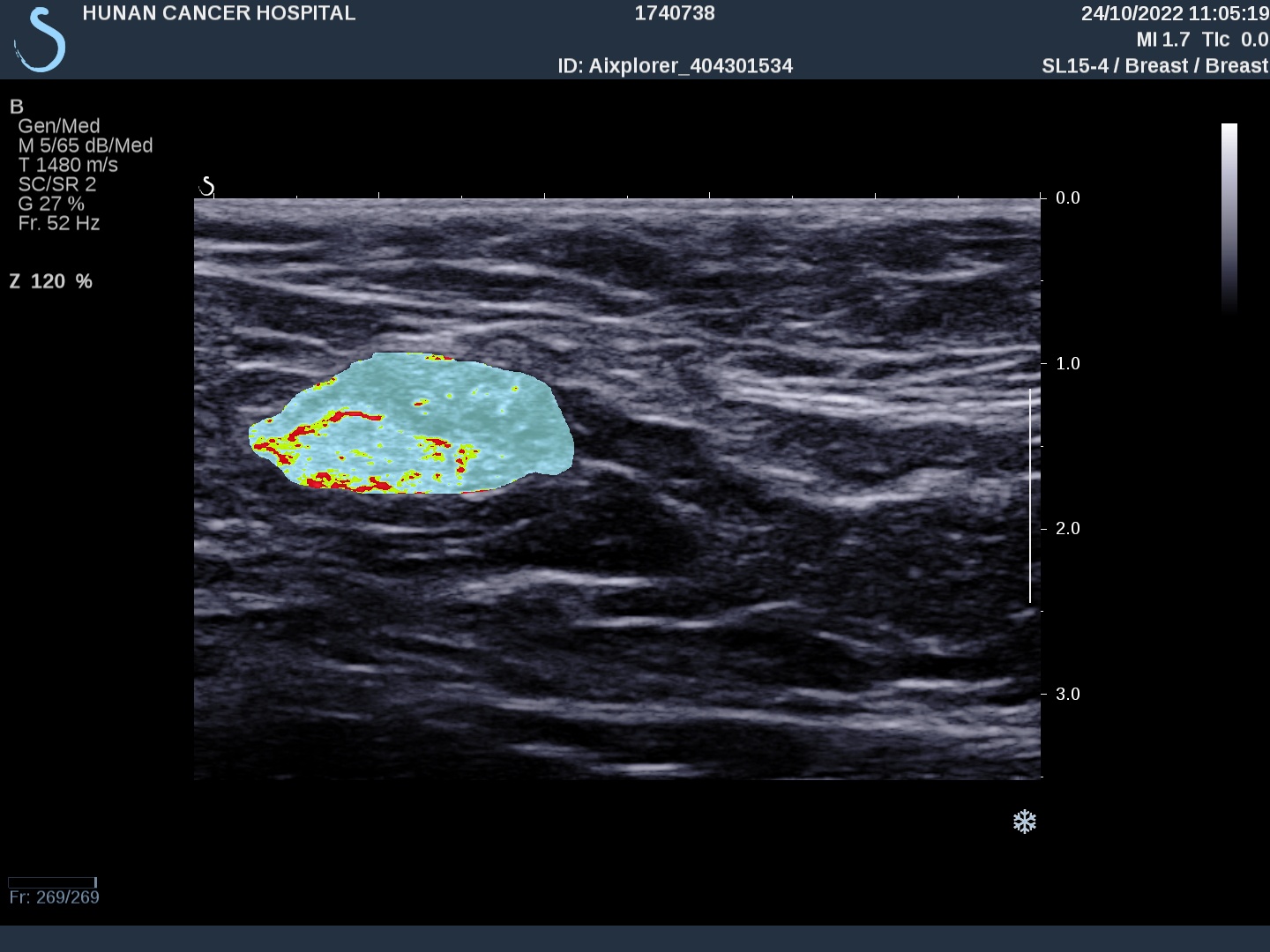

Supplement: Supplementary file 2 [file DataSheet_2.zip › ROI/1740738-1.jpg]

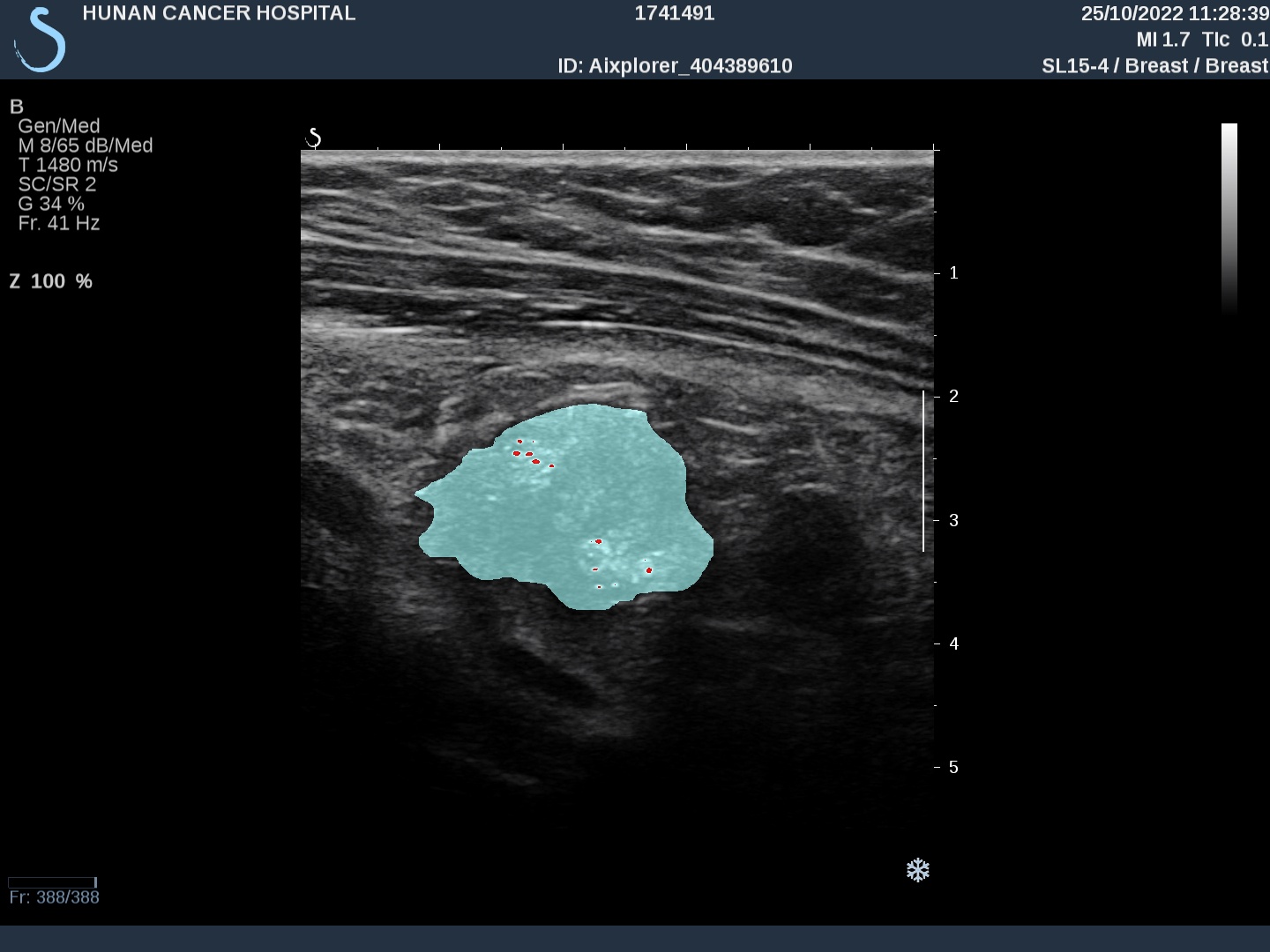

Supplement: Supplementary file 2 [file DataSheet_2.zip › ROI/1741491-1.jpg]

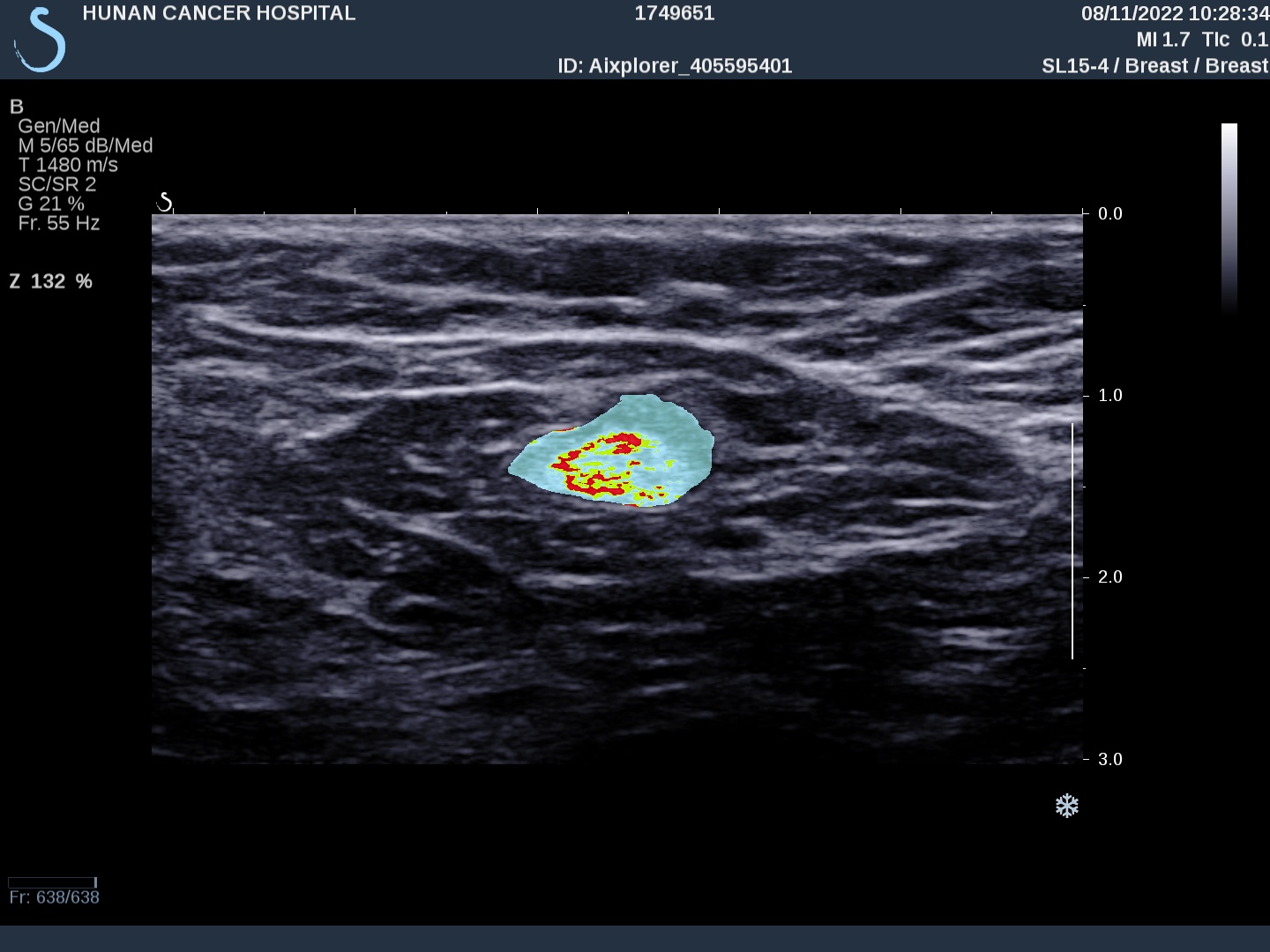

Supplement: Supplementary file 2 [file DataSheet_2.zip › ROI/1749651-1.jpg]

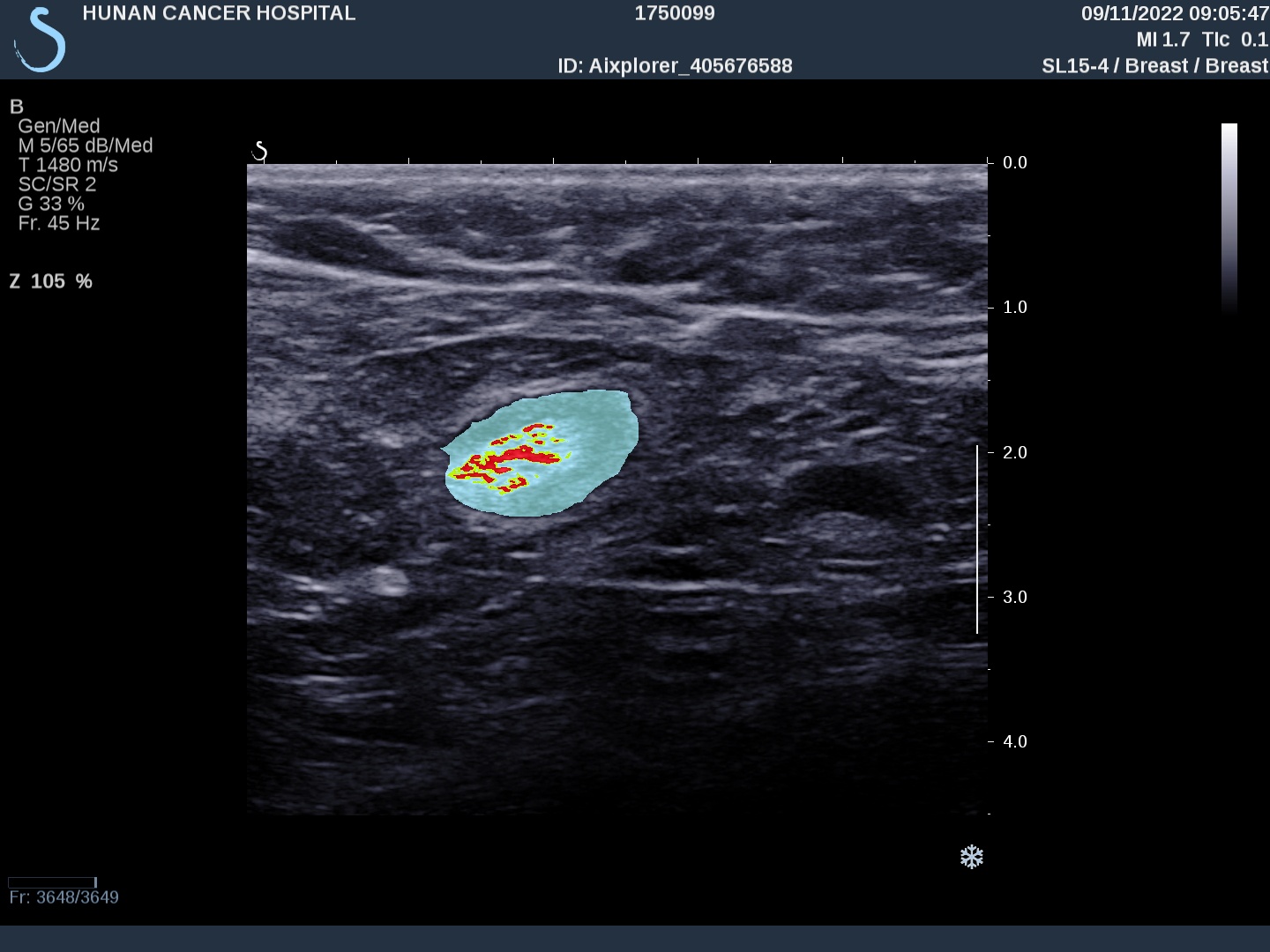

Supplement: Supplementary file 2 [file DataSheet_2.zip › ROI/1750099-1.jpg]

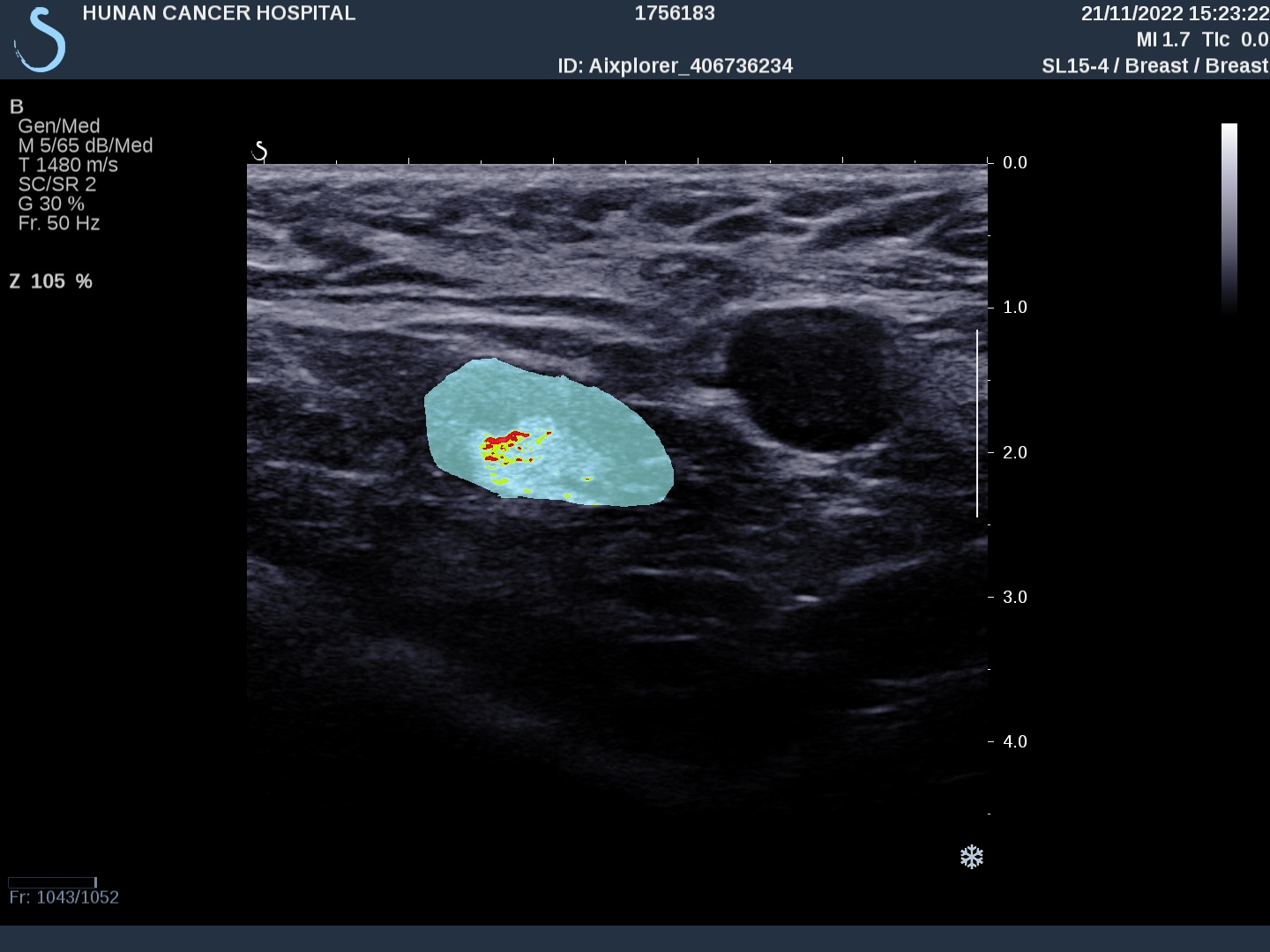

Supplement: Supplementary file 2 [file DataSheet_2.zip › ROI/1756183-1.jpg]

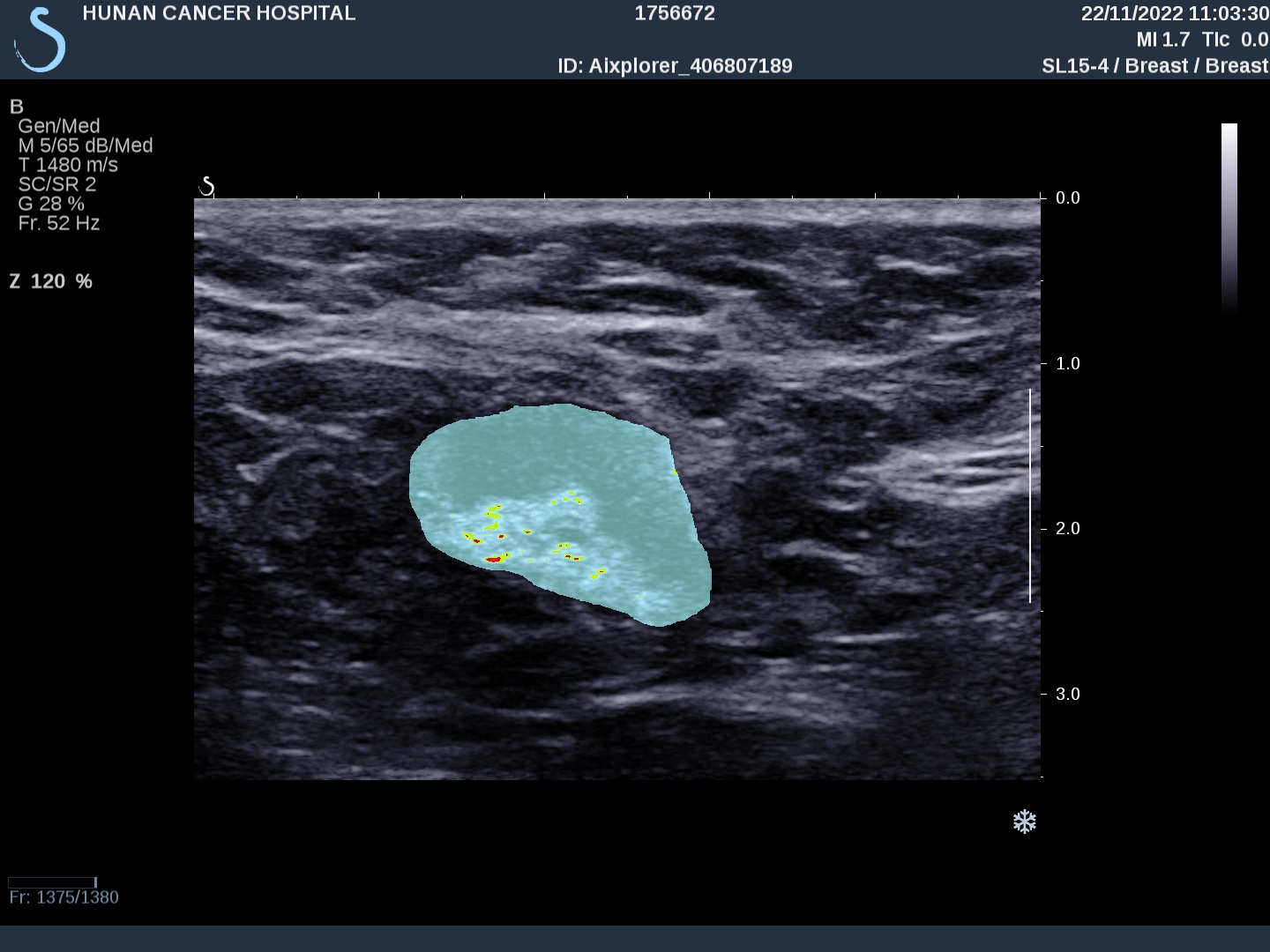

Supplement: Supplementary file 2 [file DataSheet_2.zip › ROI/1756672-1.jpg]

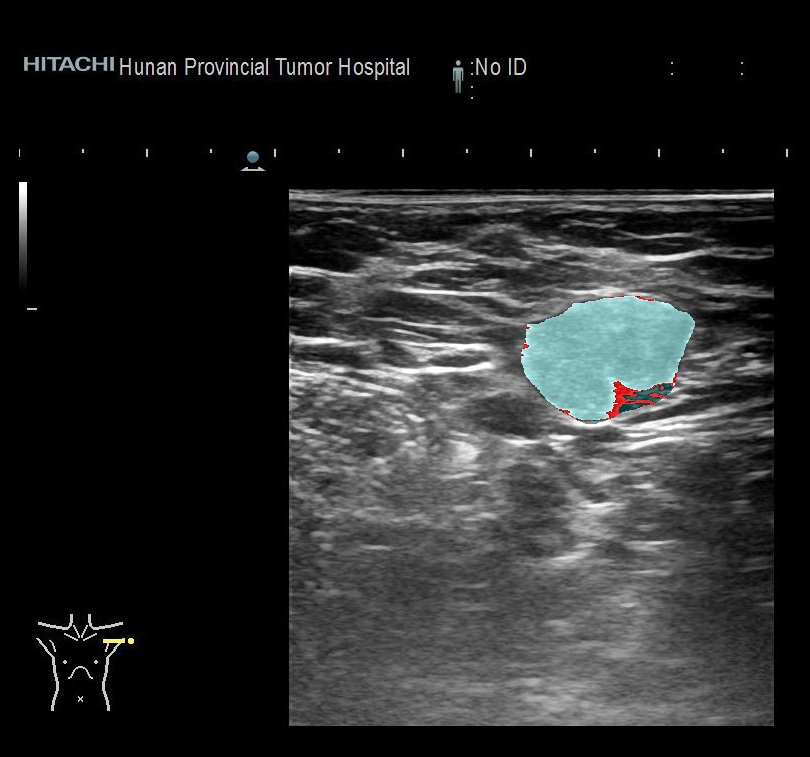

Supplement: Supplementary file 2 [file DataSheet_2.zip › ROI/1756910-1.jpg]

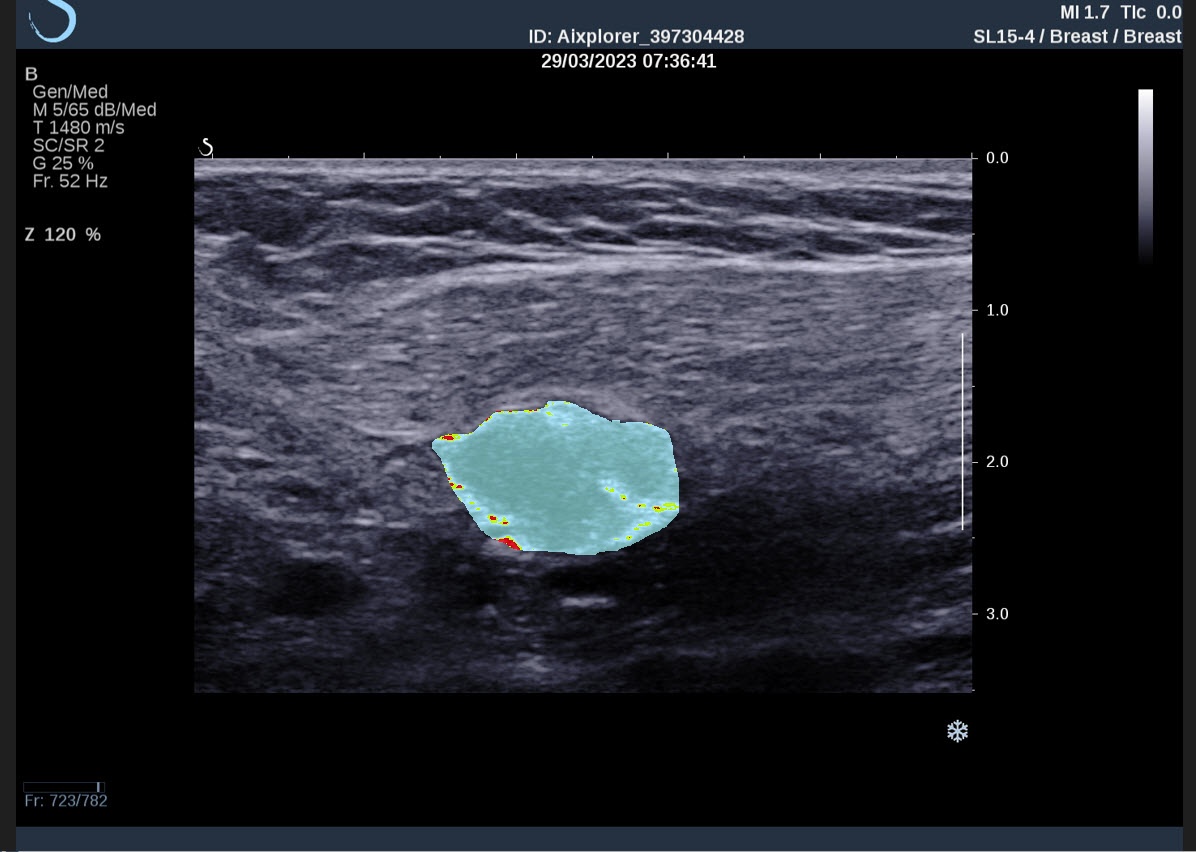

Supplement: Supplementary file 2 [file DataSheet_2.zip › ROI/1757927-1.jpg]

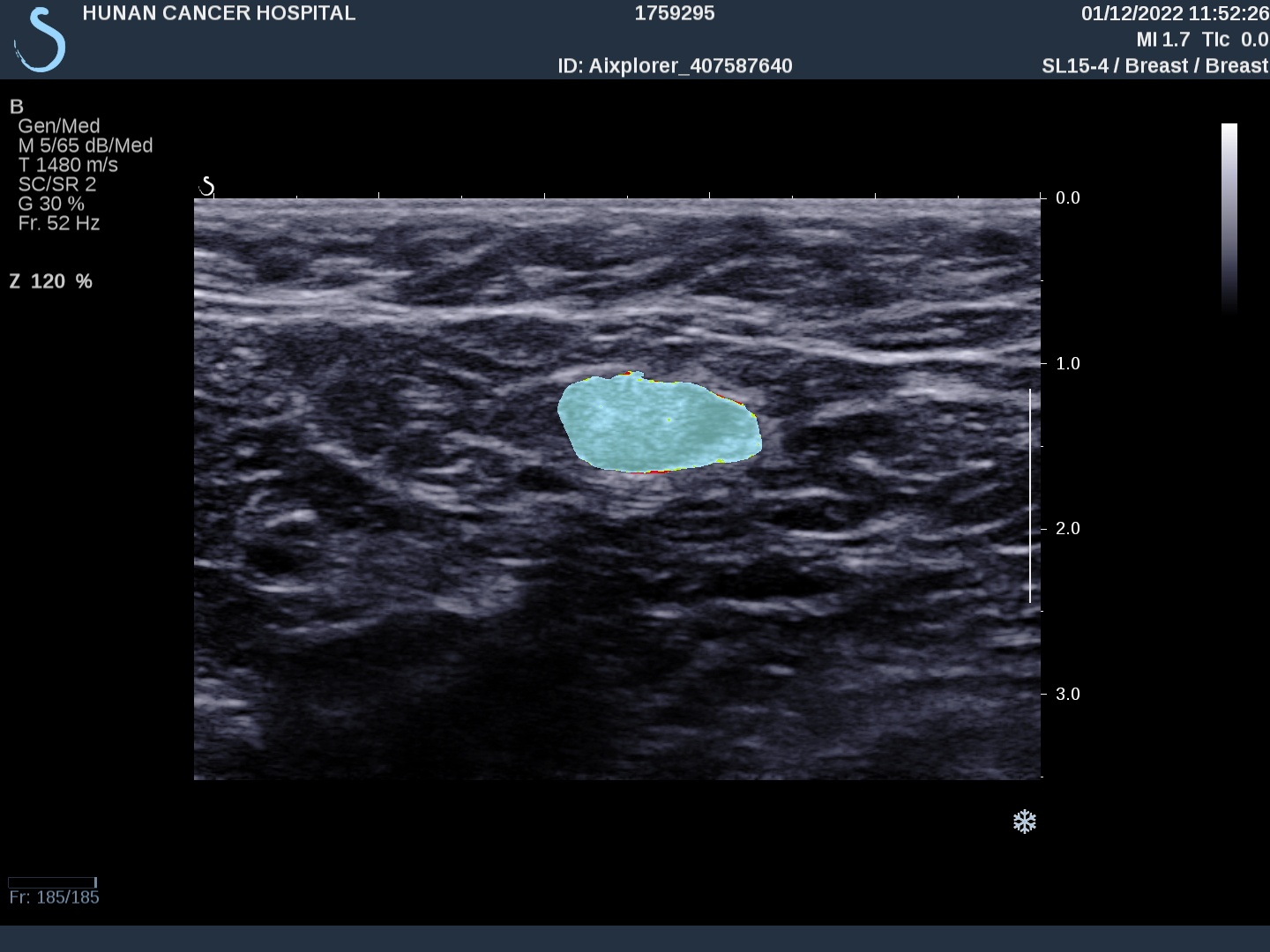

Supplement: Supplementary file 2 [file DataSheet_2.zip › ROI/1759295-1.jpg]

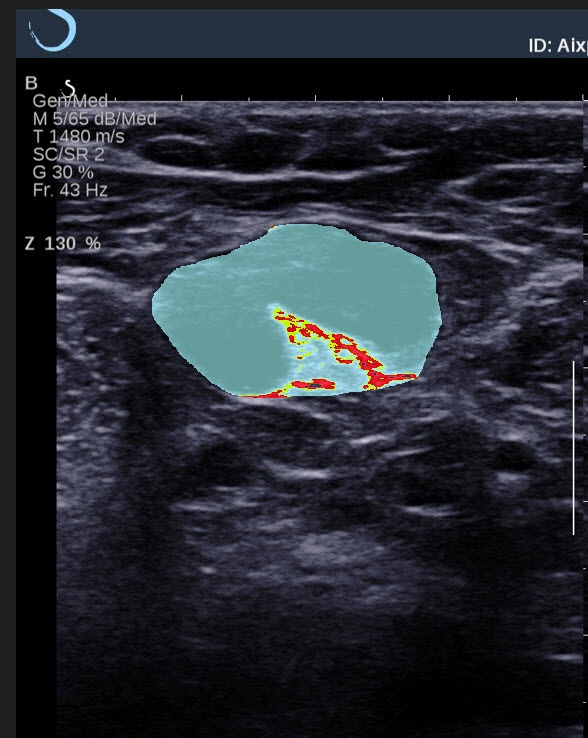

Supplement: Supplementary file 2 [file DataSheet_2.zip › ROI/1759492-1.jpg]

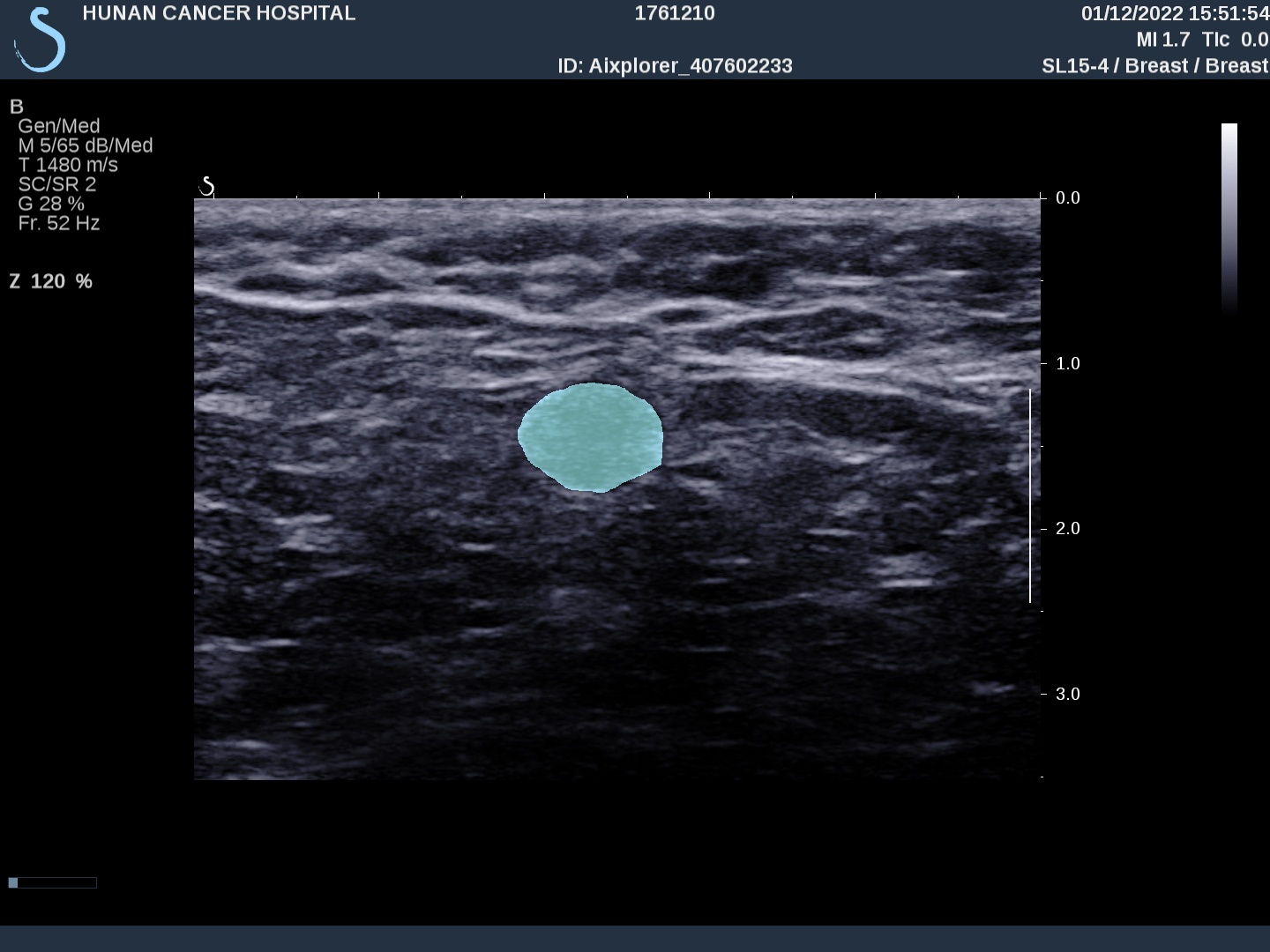

Supplement: Supplementary file 2 [file DataSheet_2.zip › ROI/1761210-2.jpg]

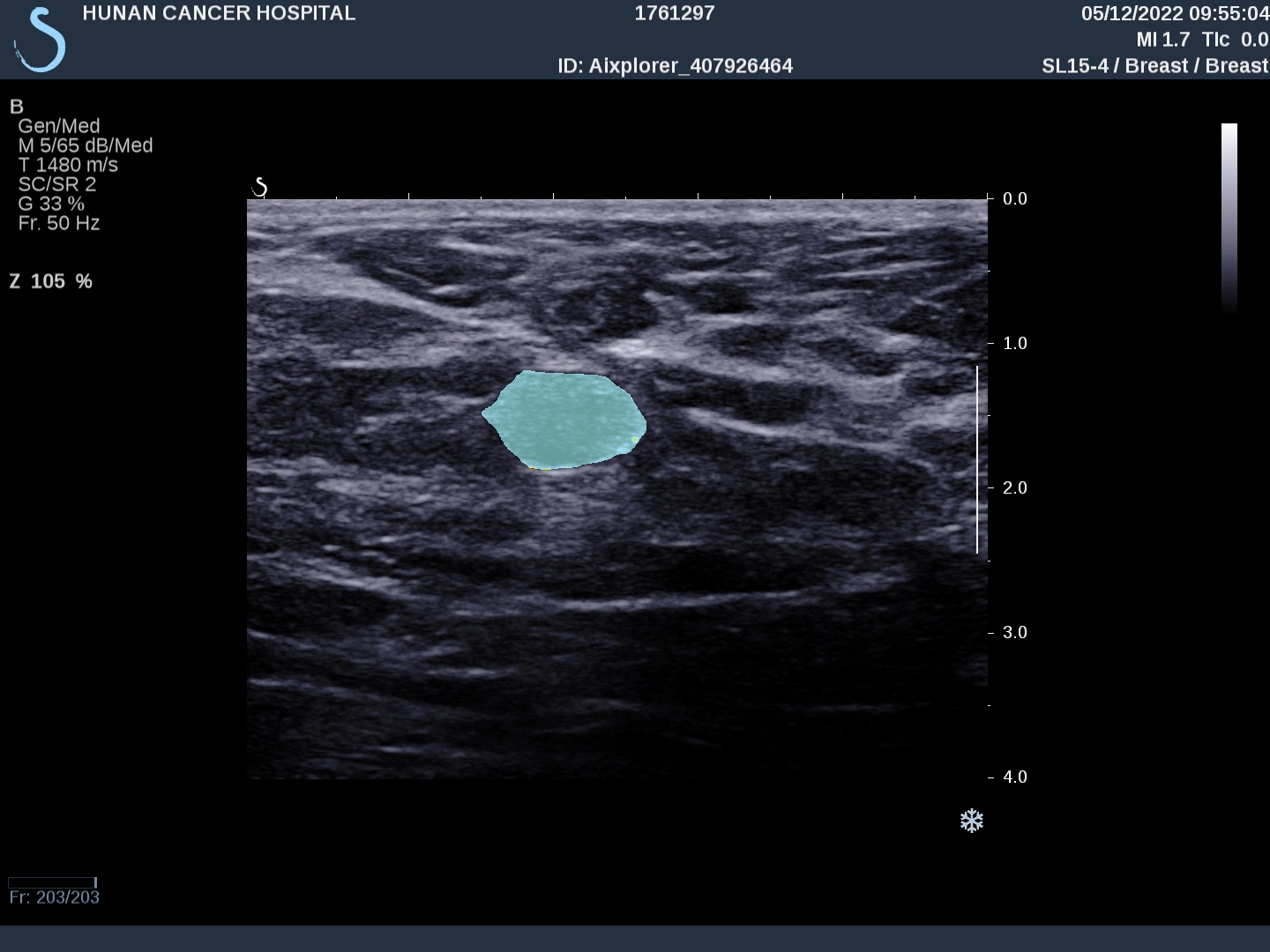

Supplement: Supplementary file 2 [file DataSheet_2.zip › ROI/1761297-1.jpg]

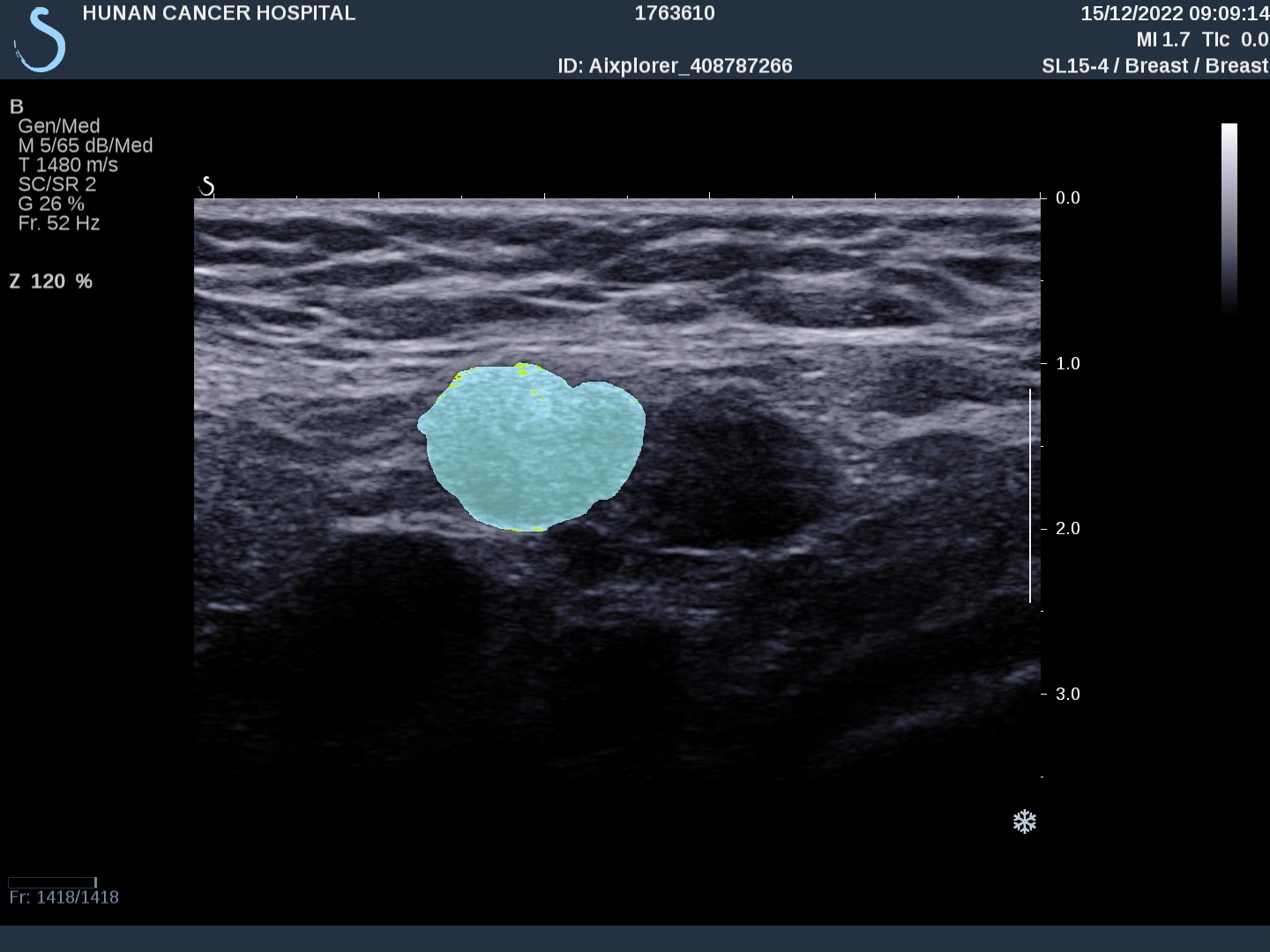

Supplement: Supplementary file 2 [file DataSheet_2.zip › ROI/1763610-1.jpg]

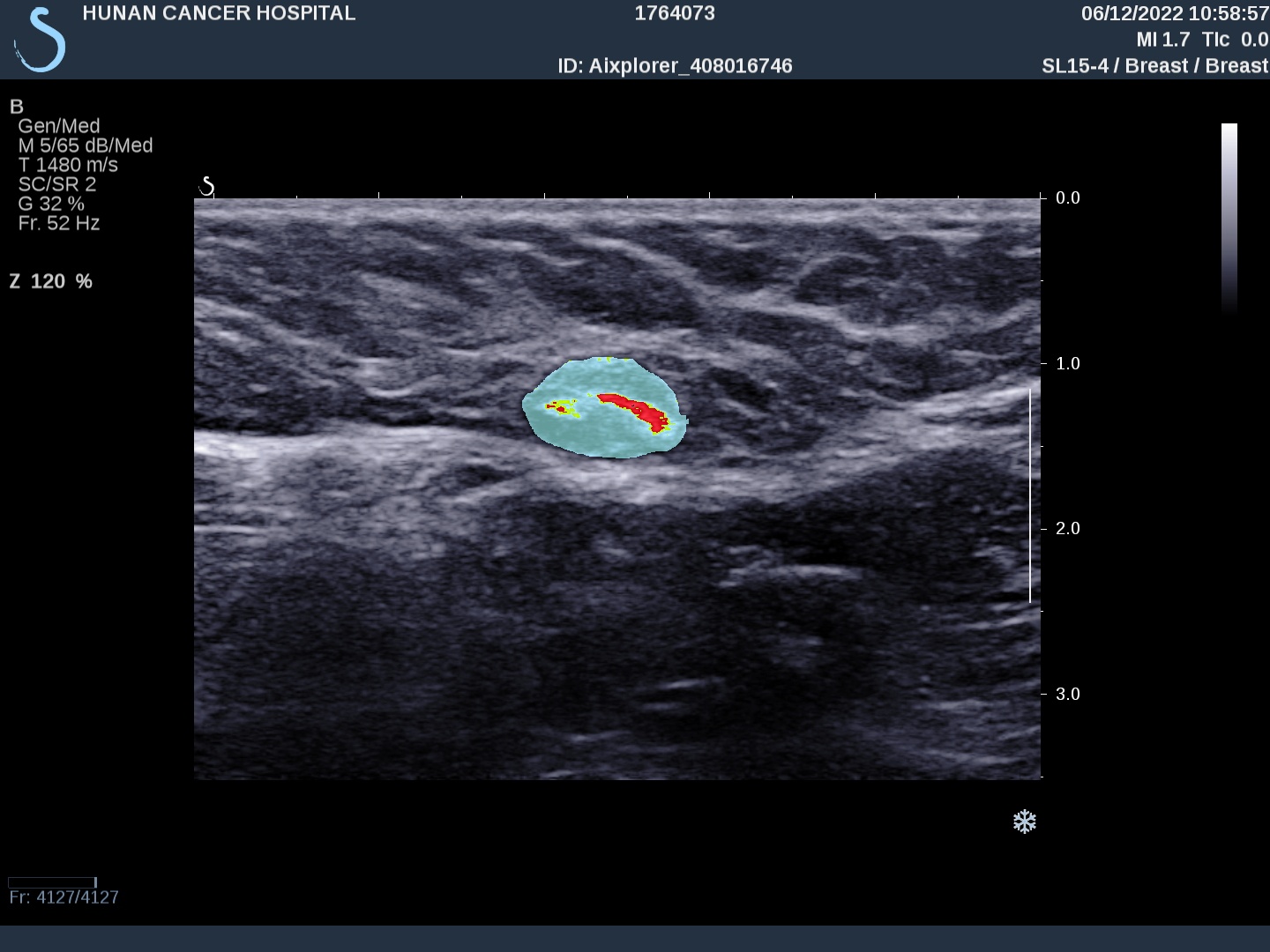

Supplement: Supplementary file 2 [file DataSheet_2.zip › ROI/1764073-1.jpg]

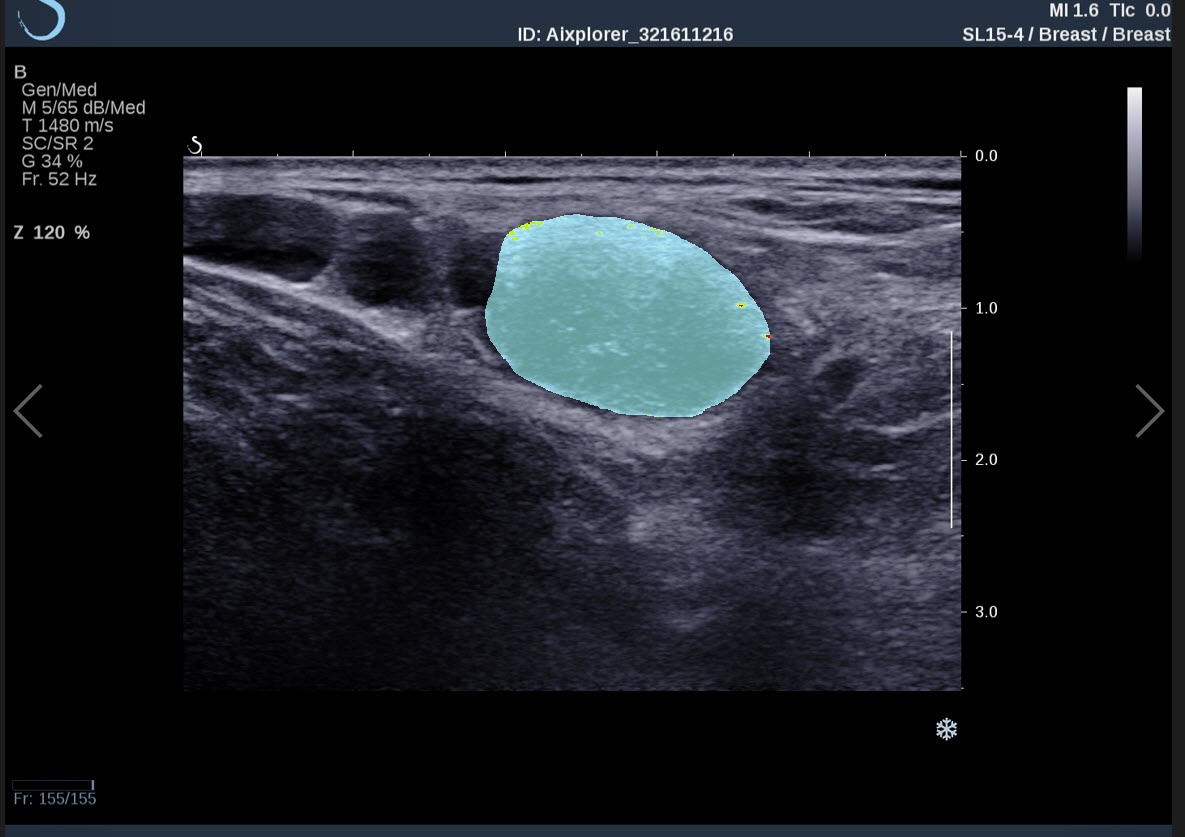

Supplement: Supplementary file 2 [file DataSheet_2.zip › ROI/1764353-1.jpg]

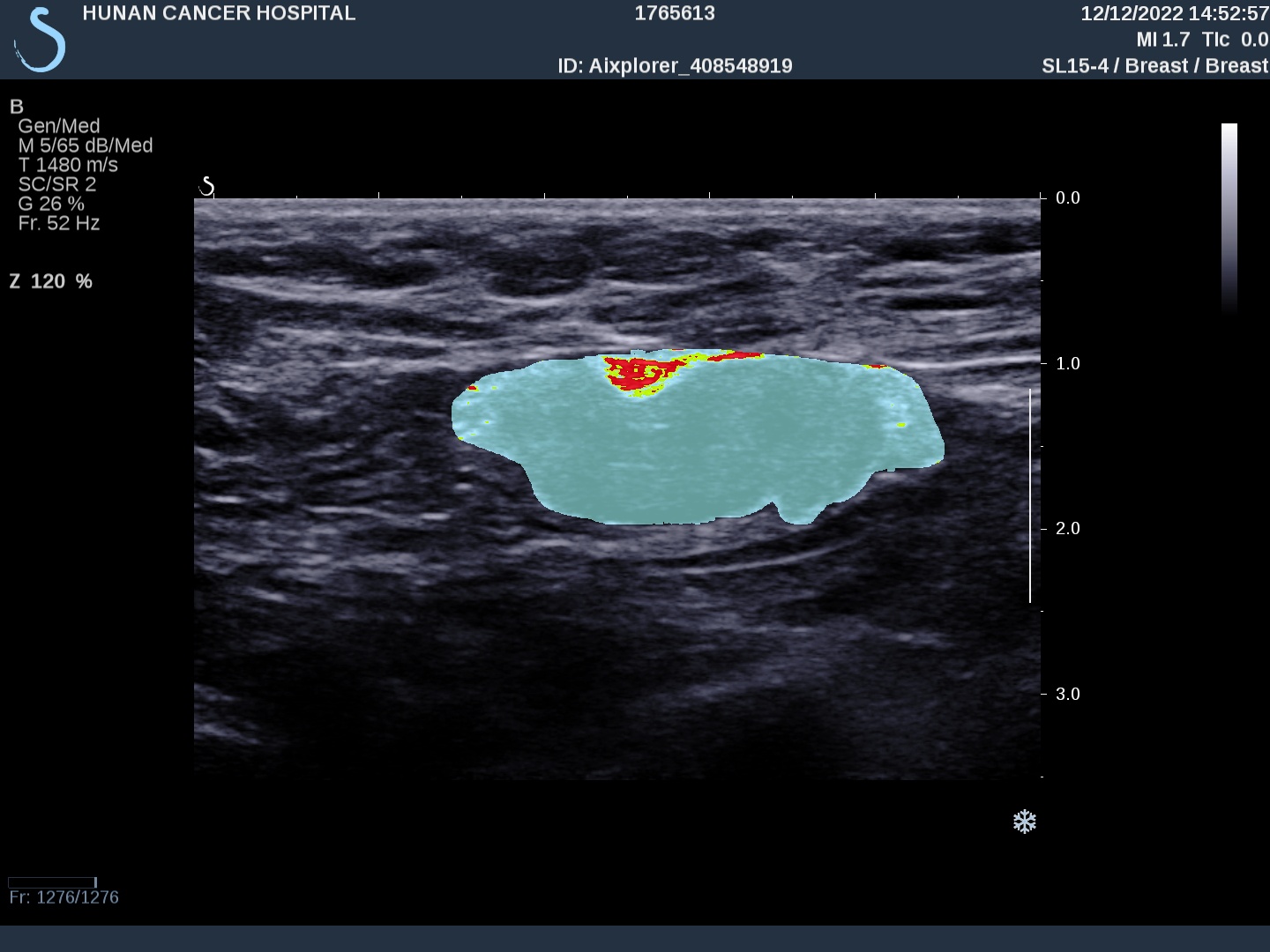

Supplement: Supplementary file 2 [file DataSheet_2.zip › ROI/1765613-1.jpg]

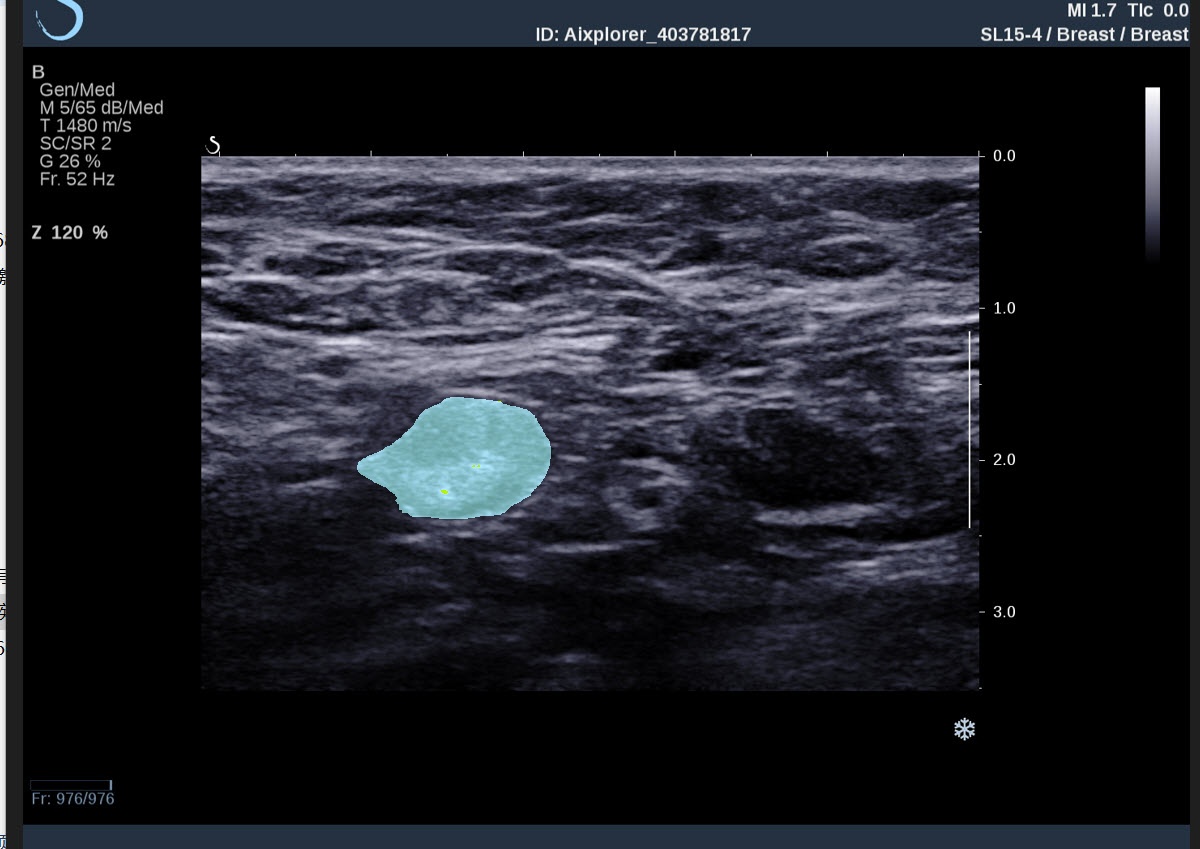

Supplement: Supplementary file 2 [file DataSheet_2.zip › ROI/1766285-3.jpg]

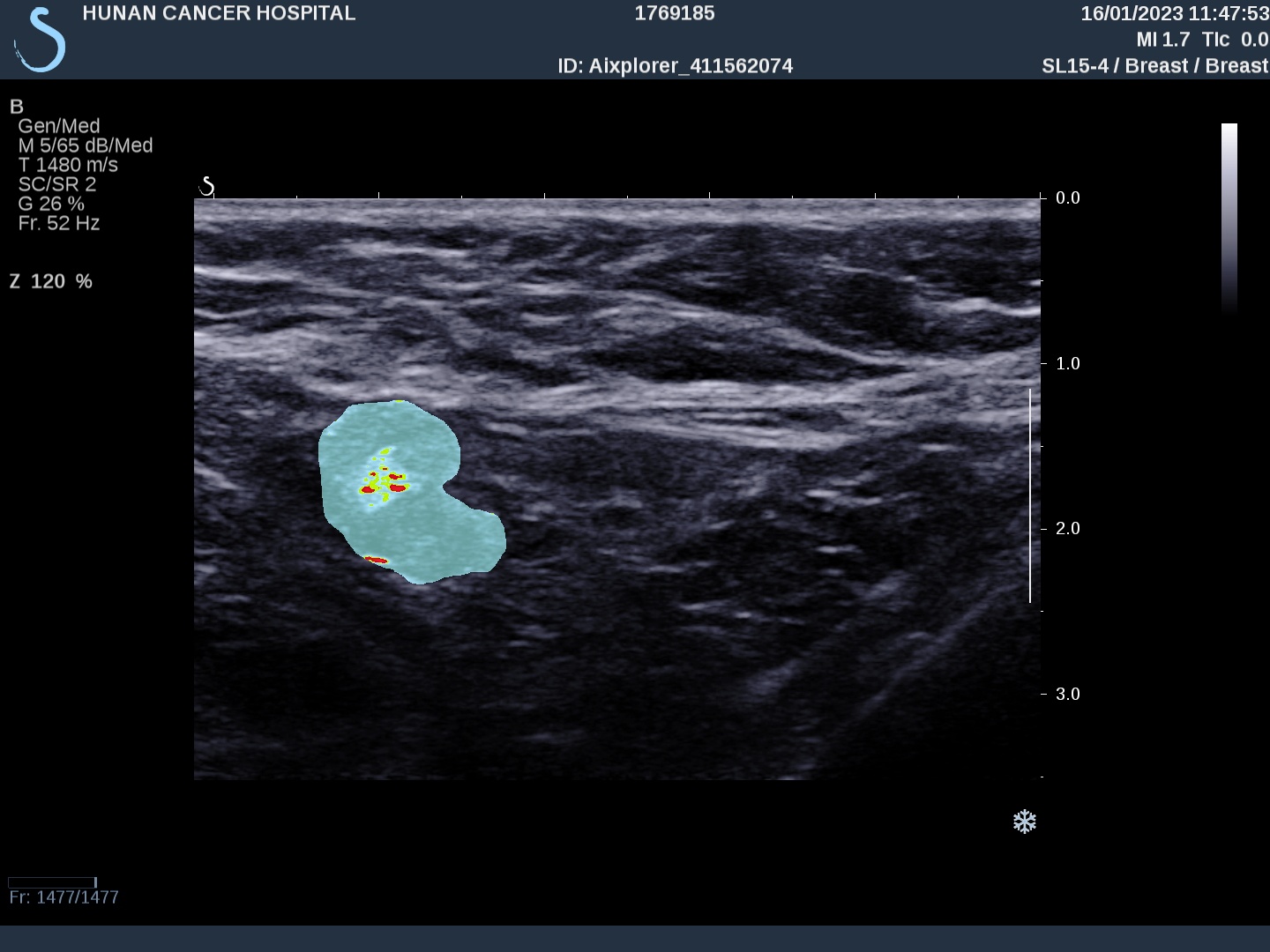

Supplement: Supplementary file 2 [file DataSheet_2.zip › ROI/1769185-1.jpg]

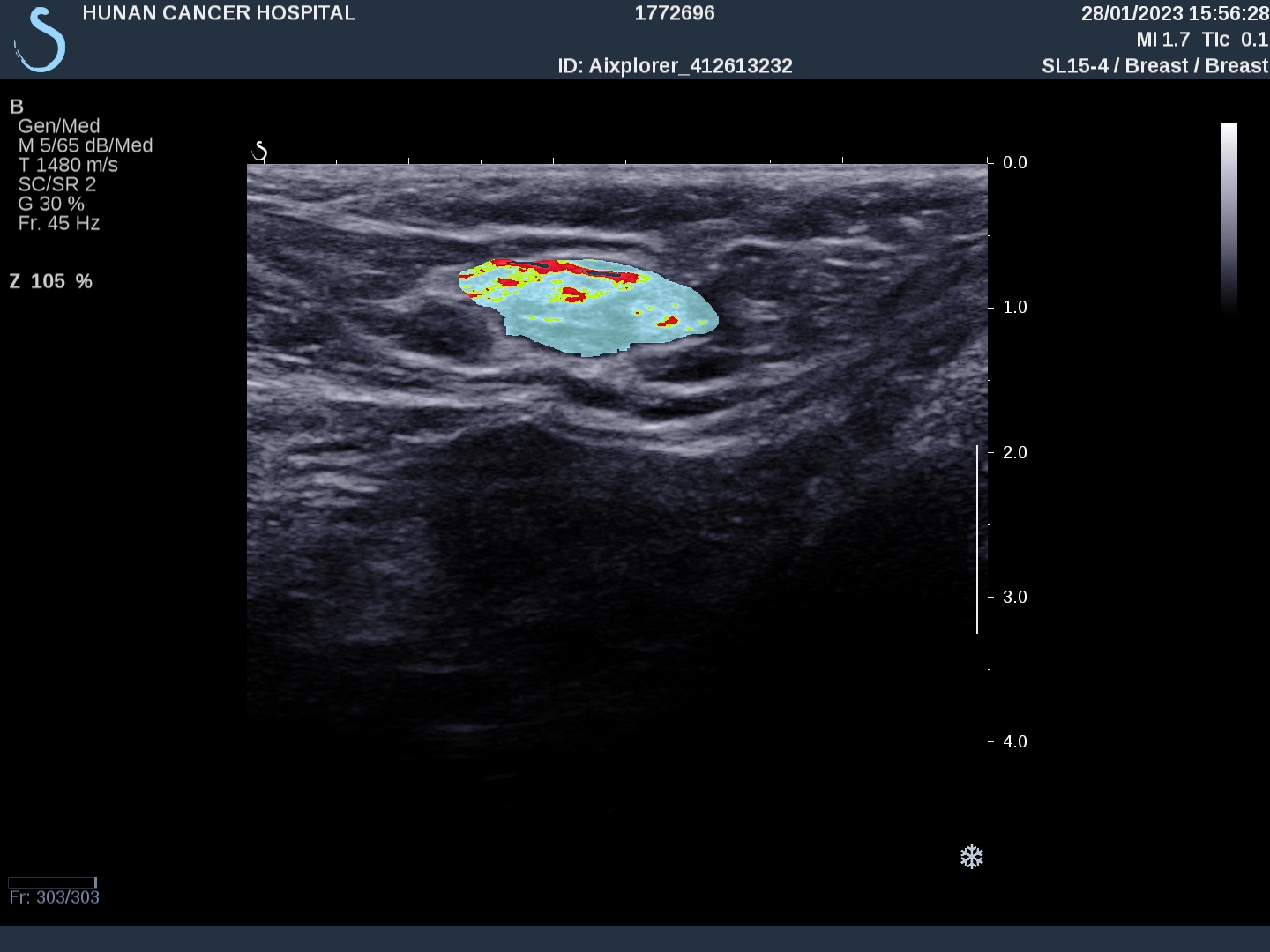

Supplement: Supplementary file 2 [file DataSheet_2.zip › ROI/1772696-1.jpg]

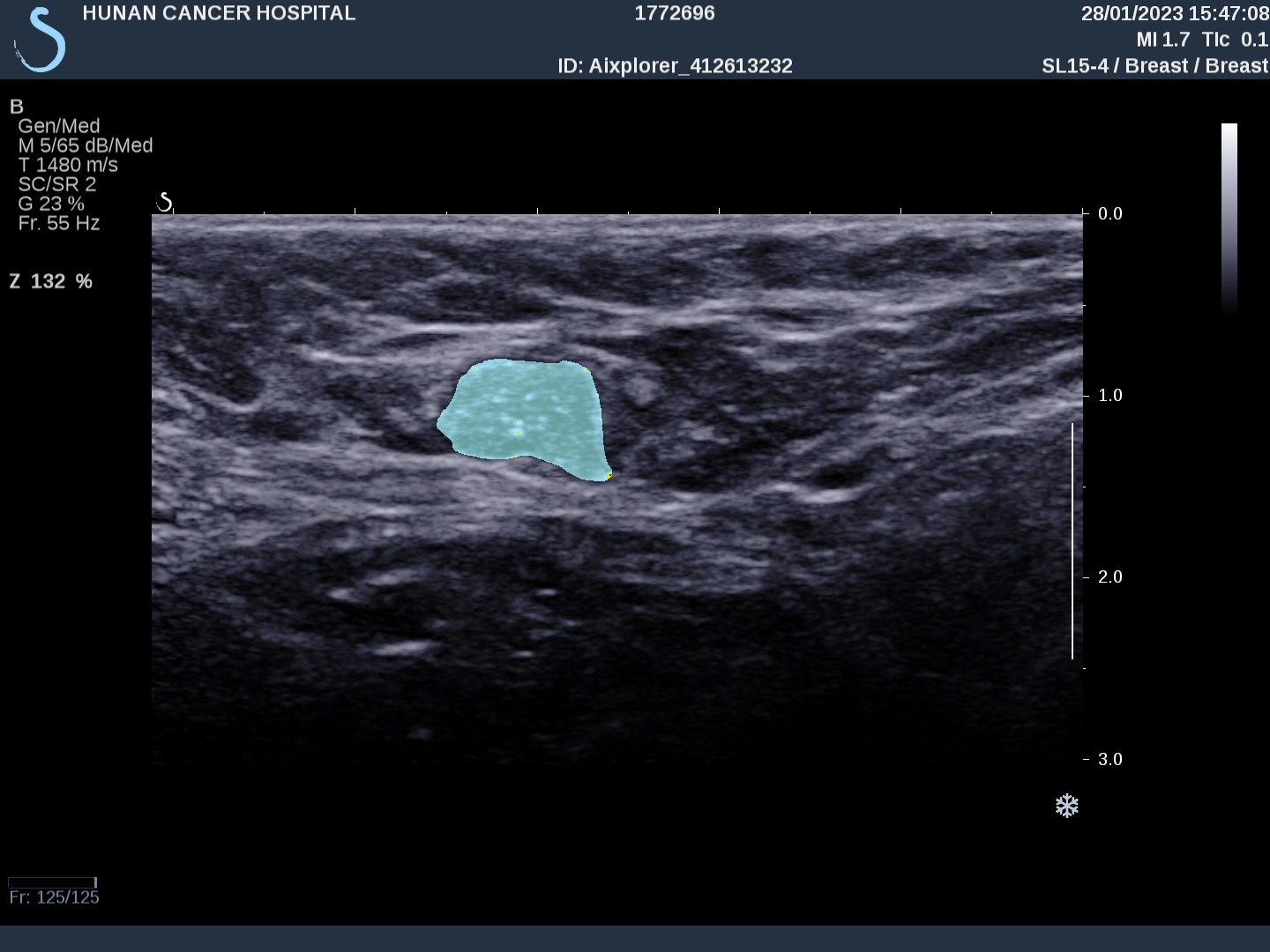

Supplement: Supplementary file 2 [file DataSheet_2.zip › ROI/1772696-5.jpg]

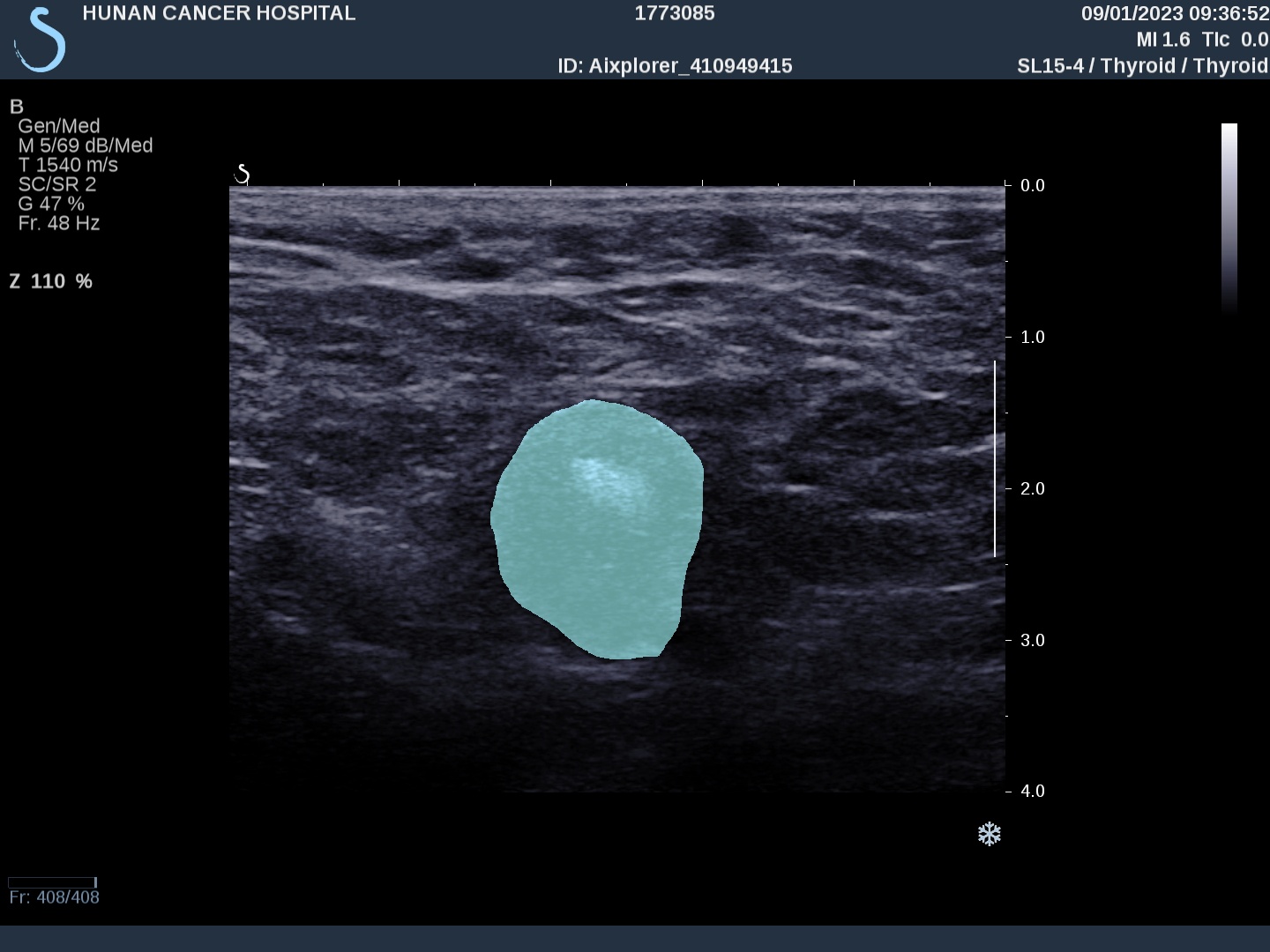

Supplement: Supplementary file 2 [file DataSheet_2.zip › ROI/1773085-1.jpg]

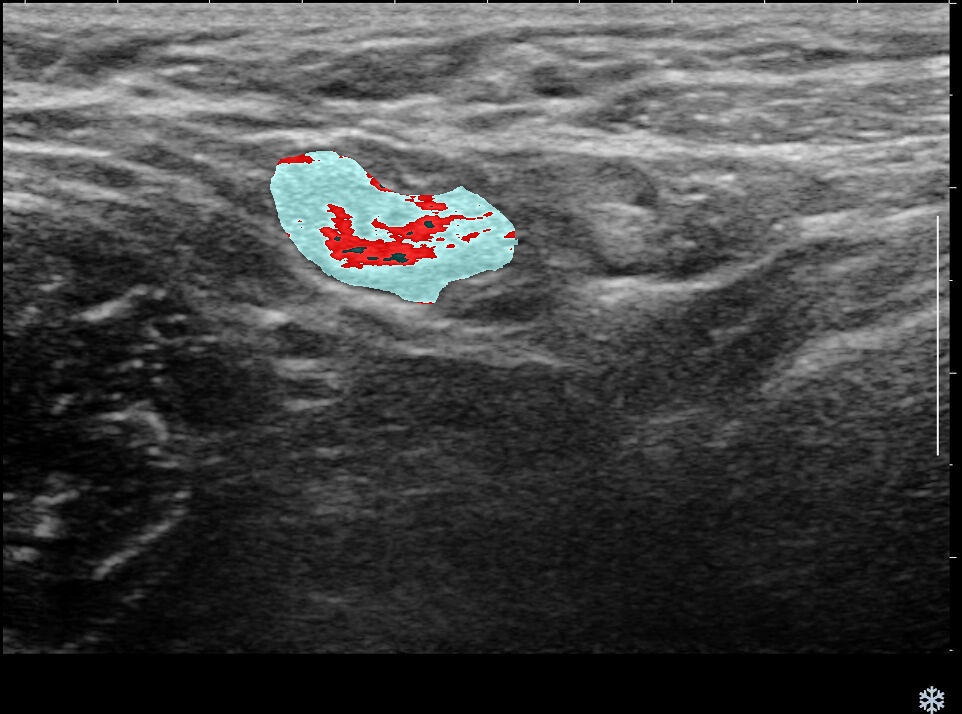

Supplement: Supplementary file 2 [file DataSheet_2.zip › ROI/1774777-1.jpg]

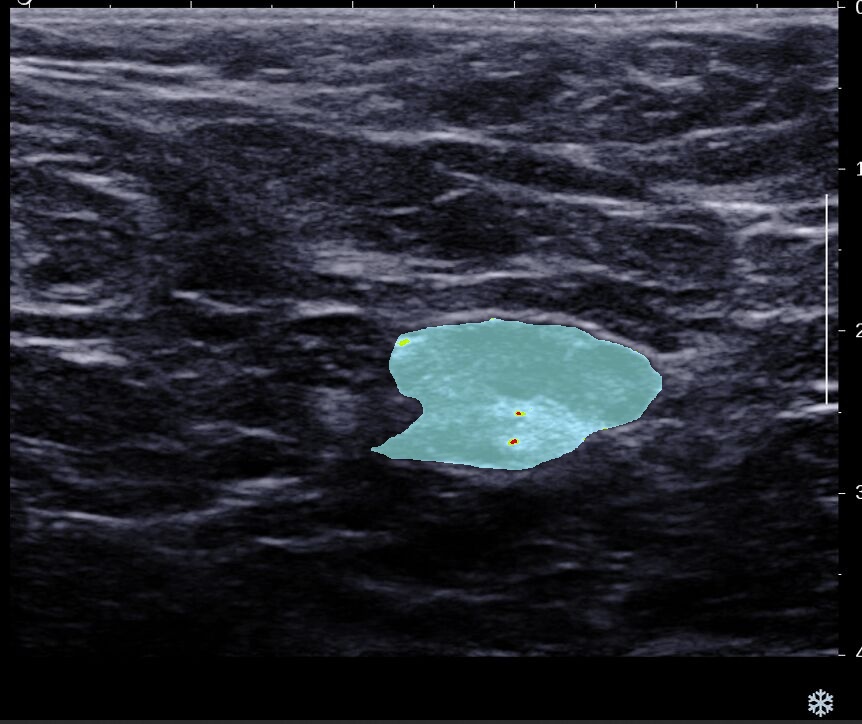

Supplement: Supplementary file 2 [file DataSheet_2.zip › ROI/1777152-2.jpg]

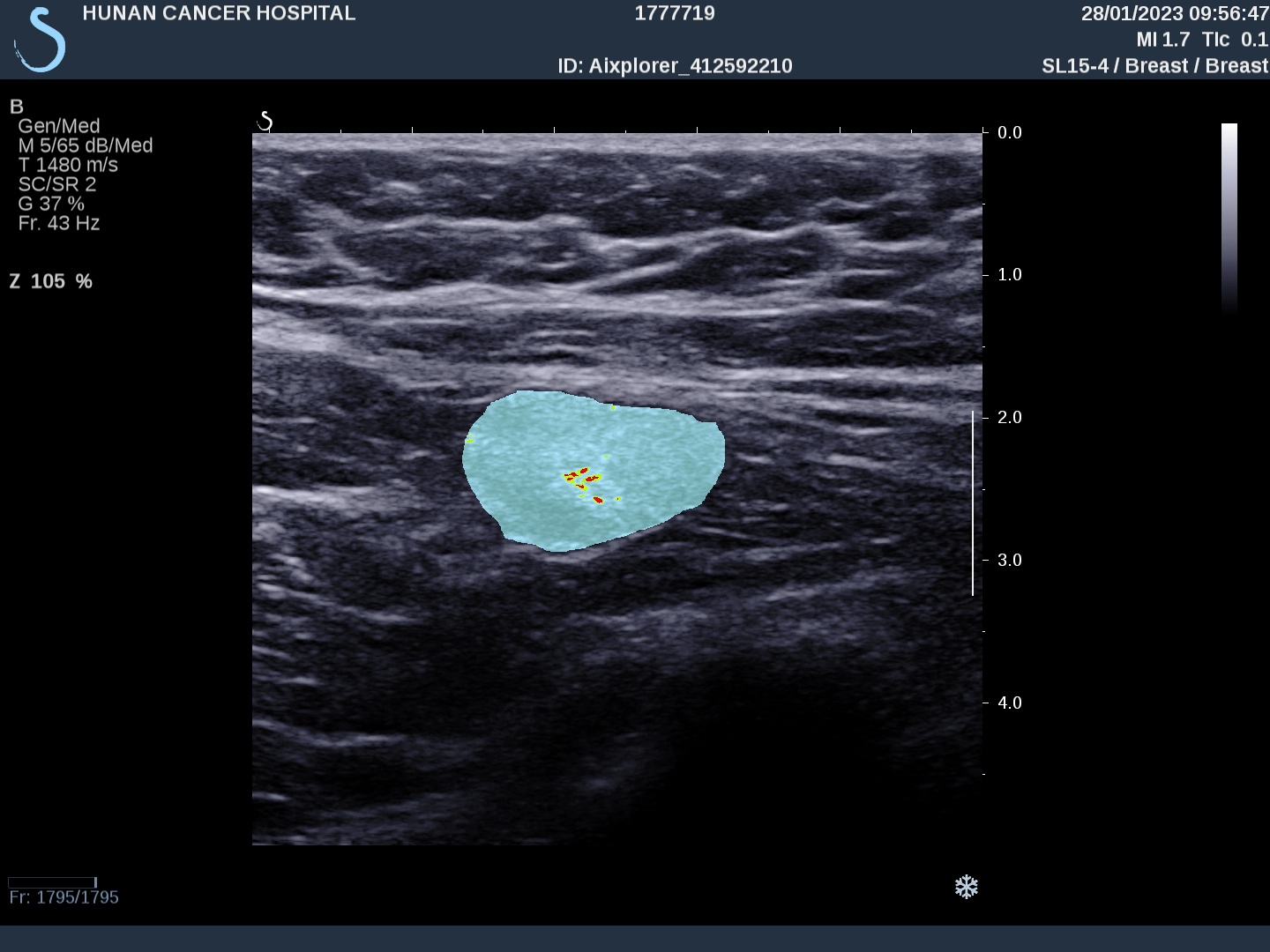

Supplement: Supplementary file 2 [file DataSheet_2.zip › ROI/1777719-1.jpg]

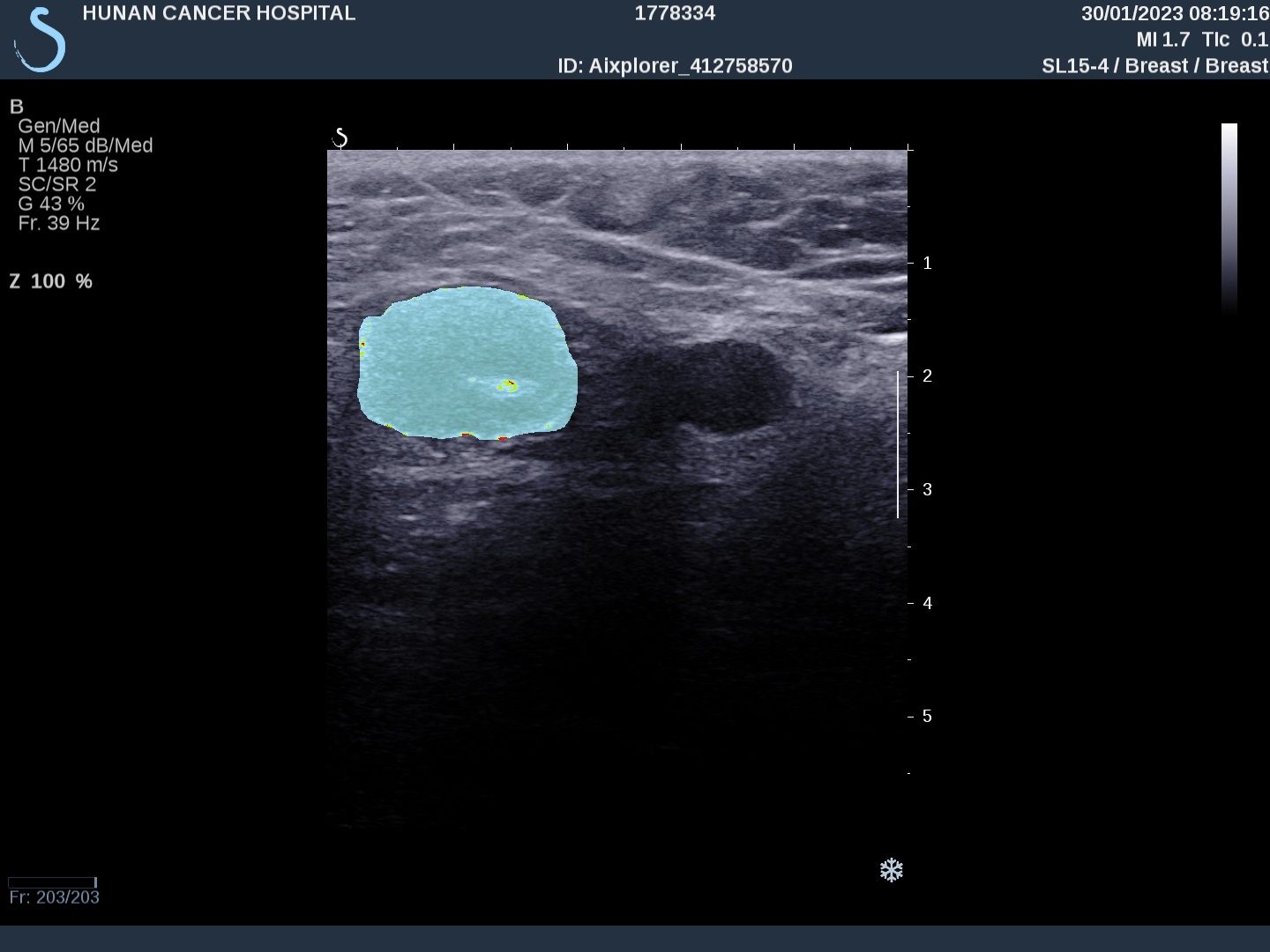

Supplement: Supplementary file 2 [file DataSheet_2.zip › ROI/1778334-1.jpg]

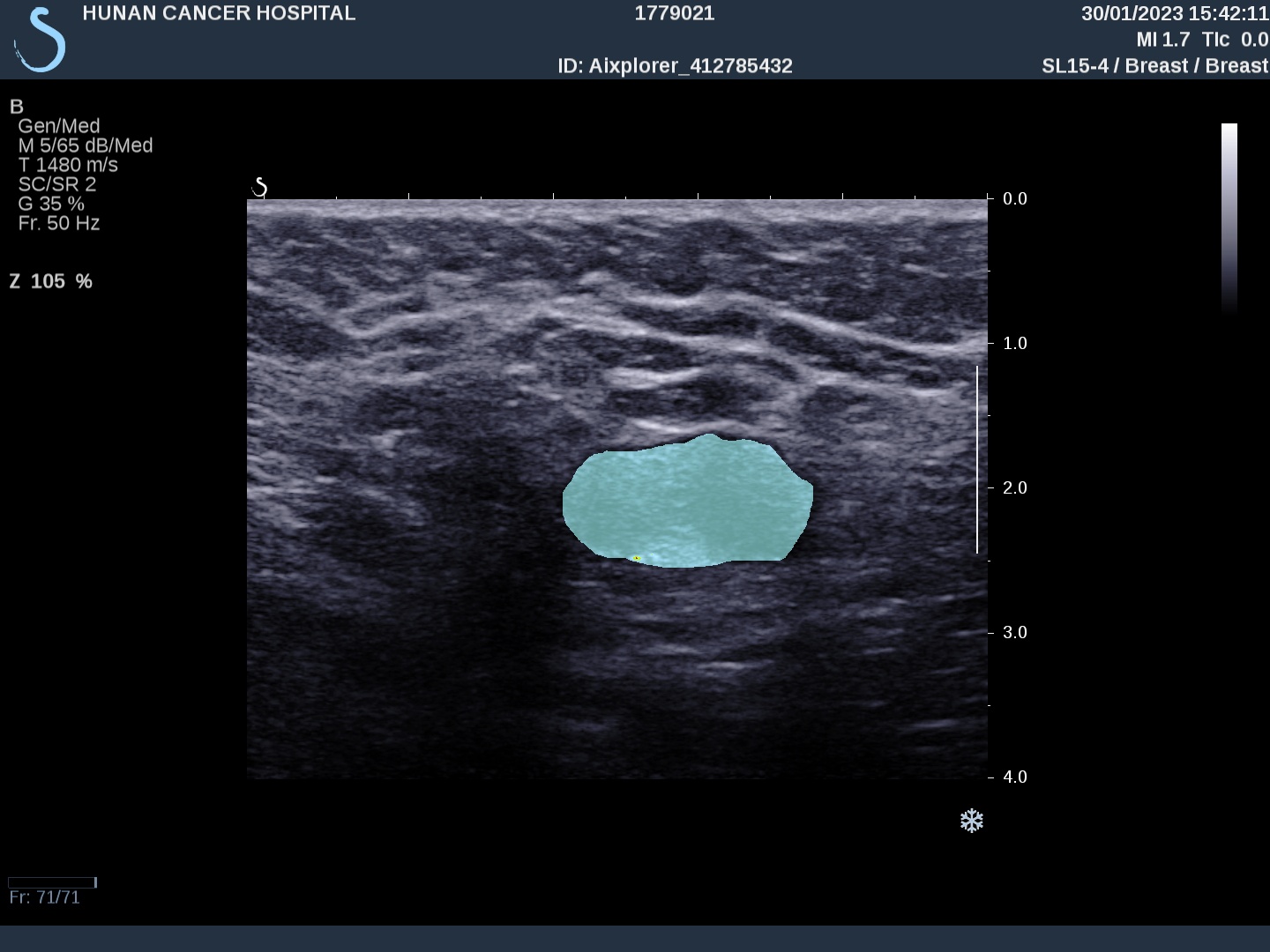

Supplement: Supplementary file 2 [file DataSheet_2.zip › ROI/1779021-5.jpg]

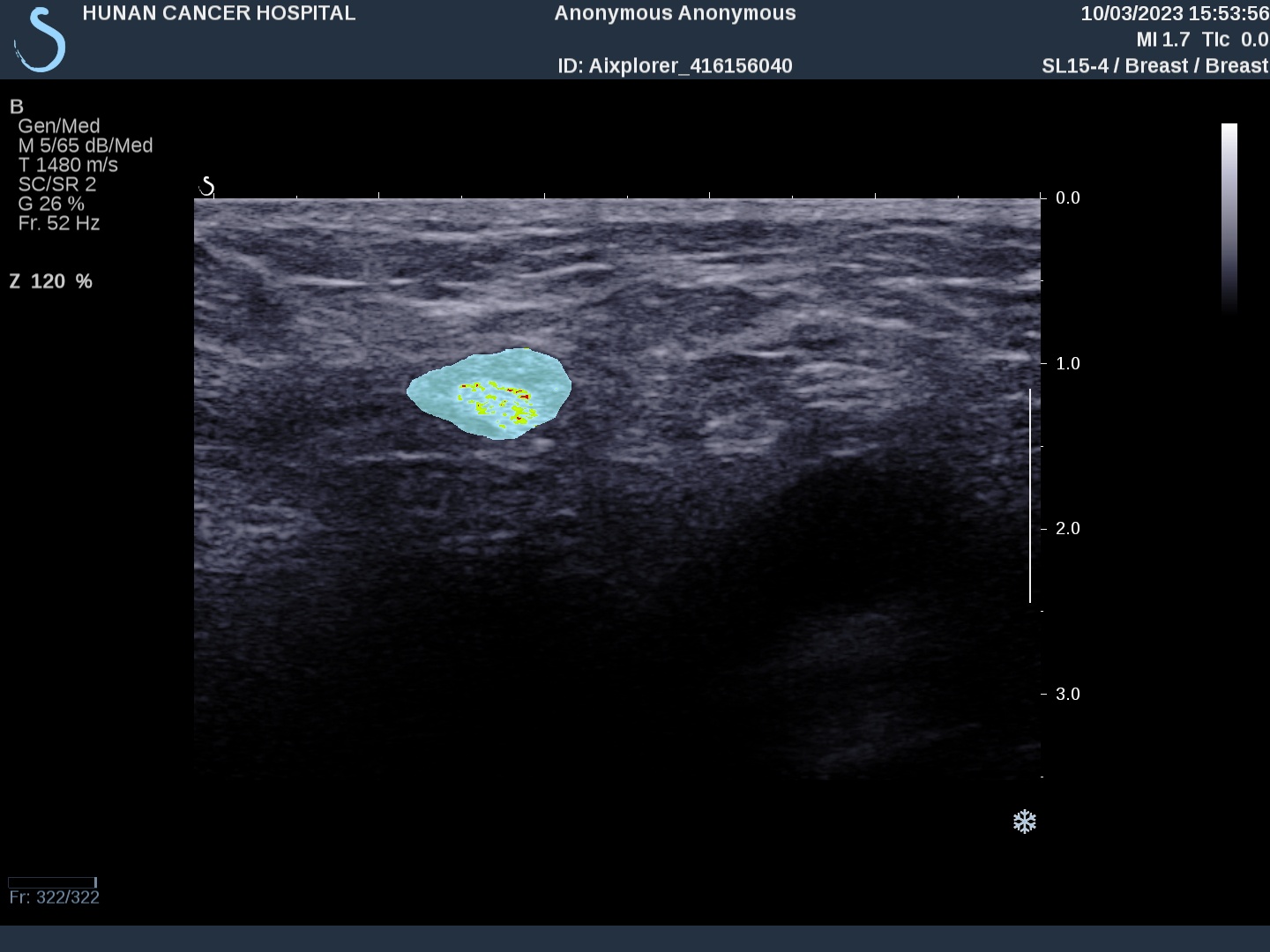

Supplement: Supplementary file 2 [file DataSheet_2.zip › ROI/1783491-1.jpg]

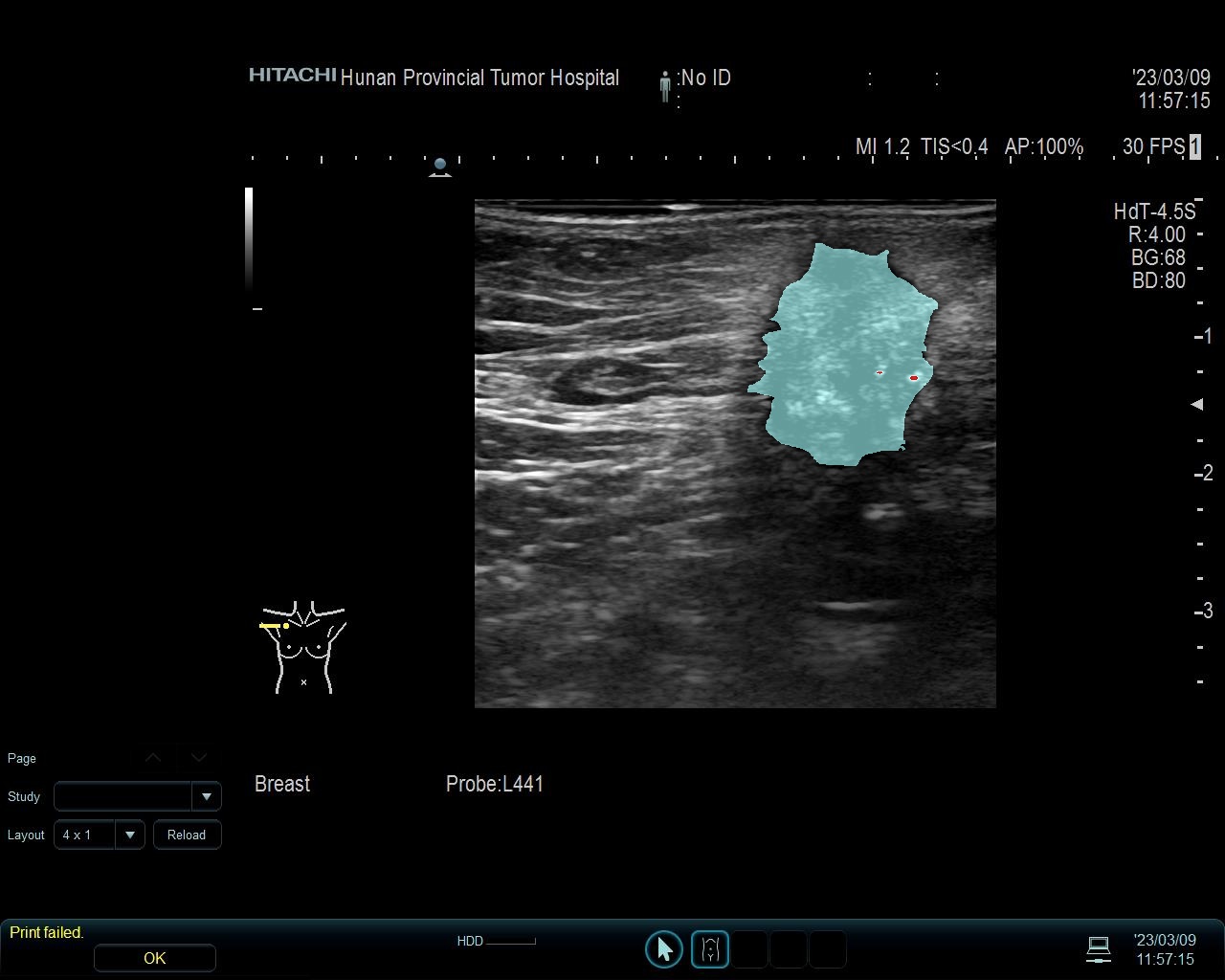

Supplement: Supplementary file 2 [file DataSheet_2.zip › ROI/1785072-1.jpg]

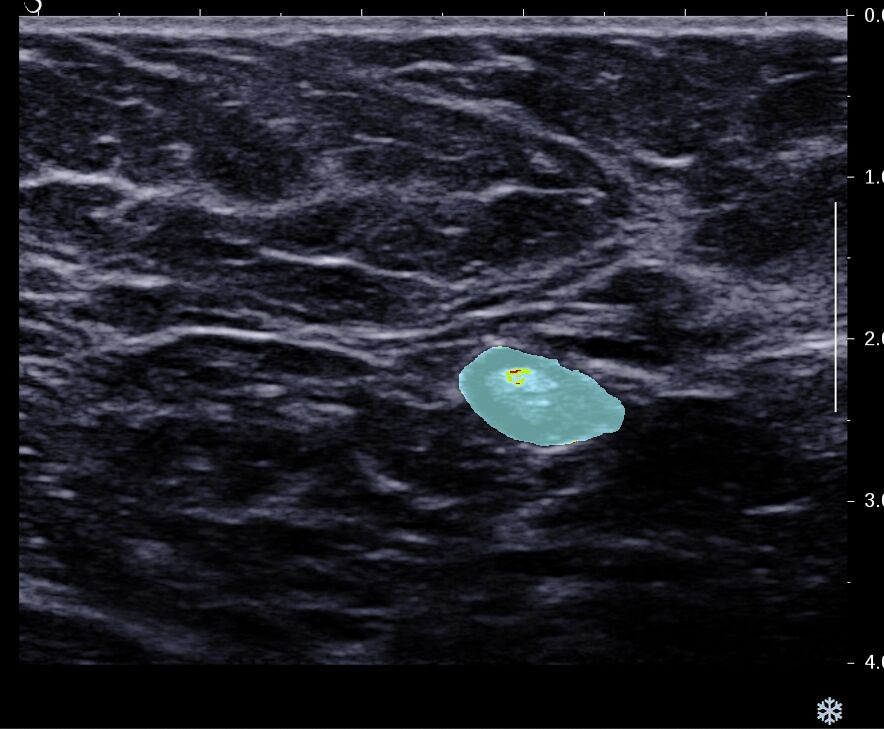

Supplement: Supplementary file 2 [file DataSheet_2.zip › ROI/1786472-3.jpg]

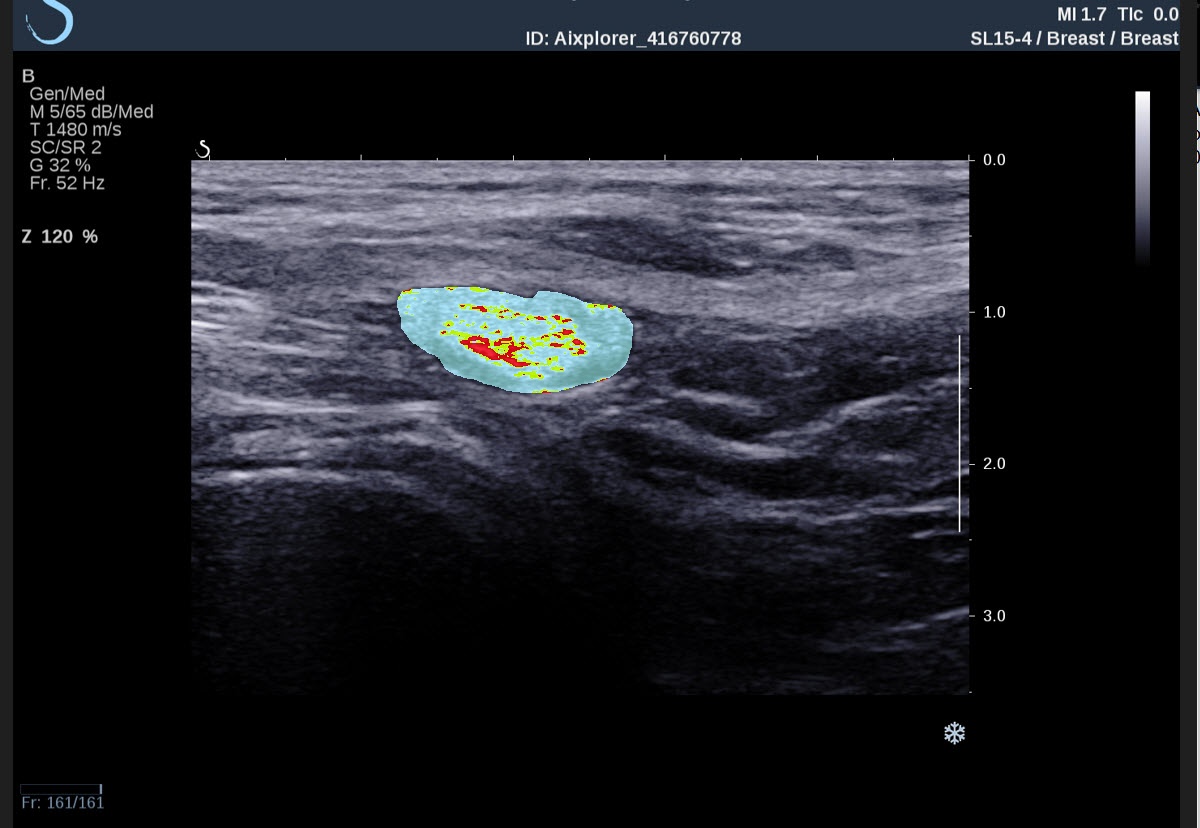

Supplement: Supplementary file 2 [file DataSheet_2.zip › ROI/1787135-2.jpg]

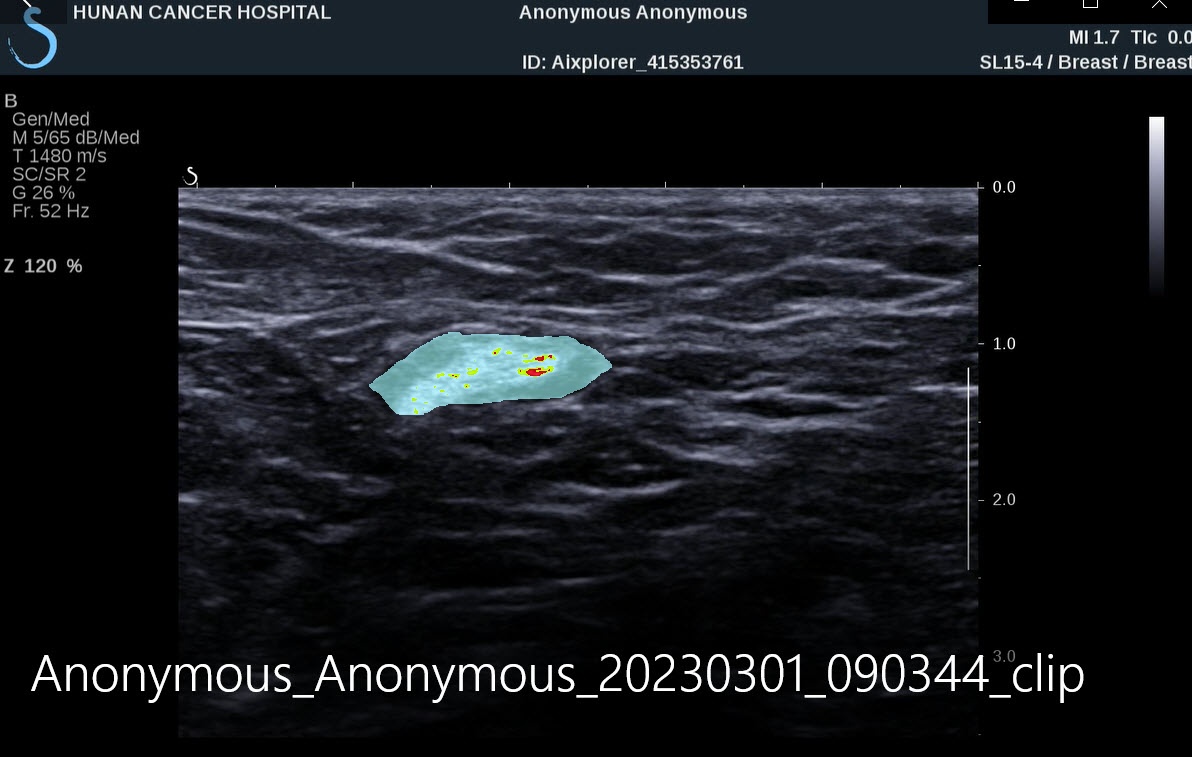

Supplement: Supplementary file 2 [file DataSheet_2.zip › ROI/1787268-1.jpg]

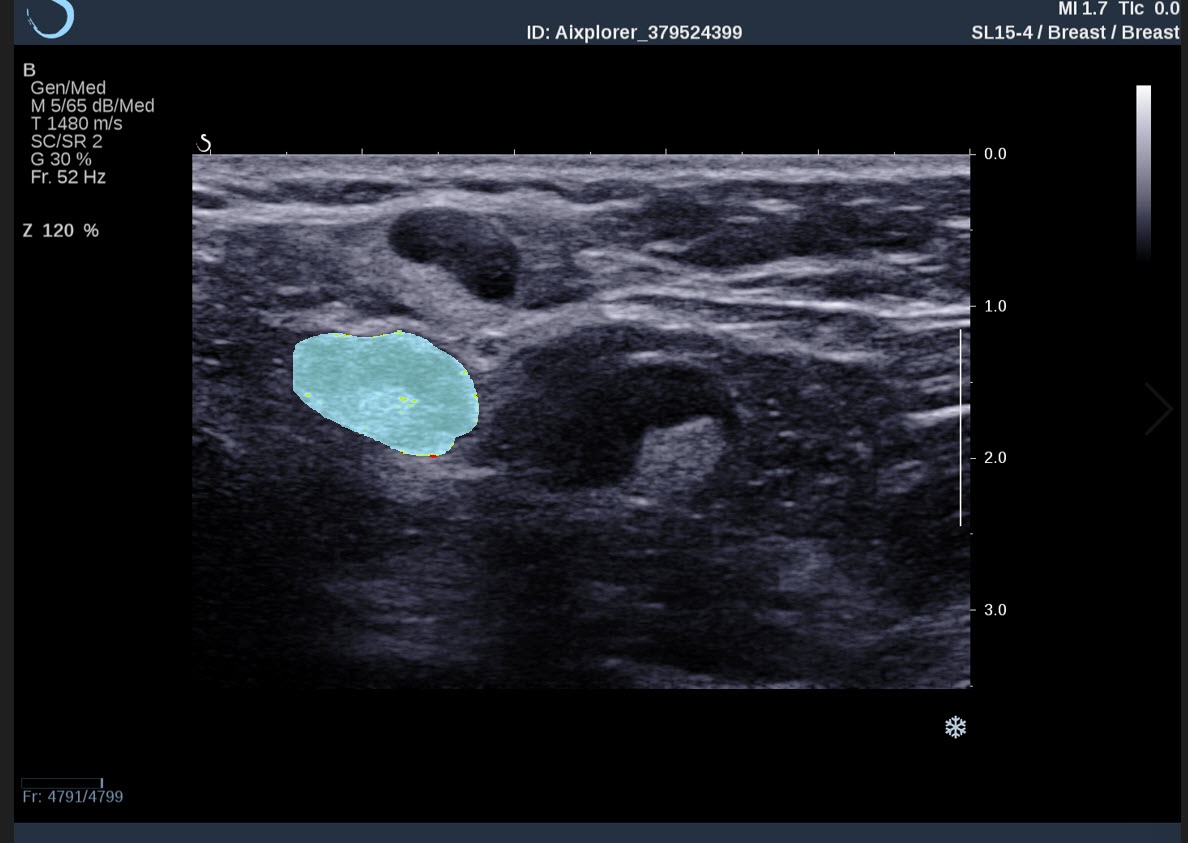

Supplement: Supplementary file 2 [file DataSheet_2.zip › ROI/1790768-1.jpg]

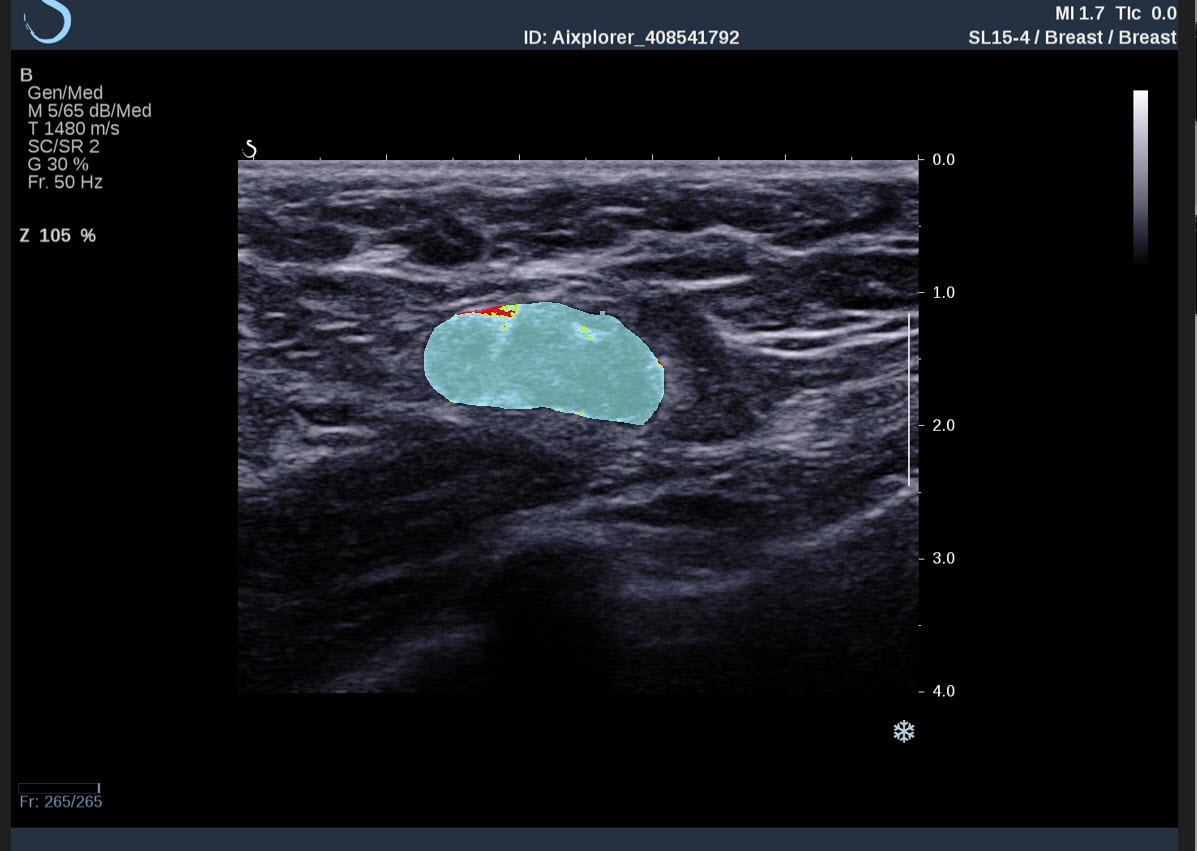

Supplement: Supplementary file 2 [file DataSheet_2.zip › ROI/1790966-1.jpg]

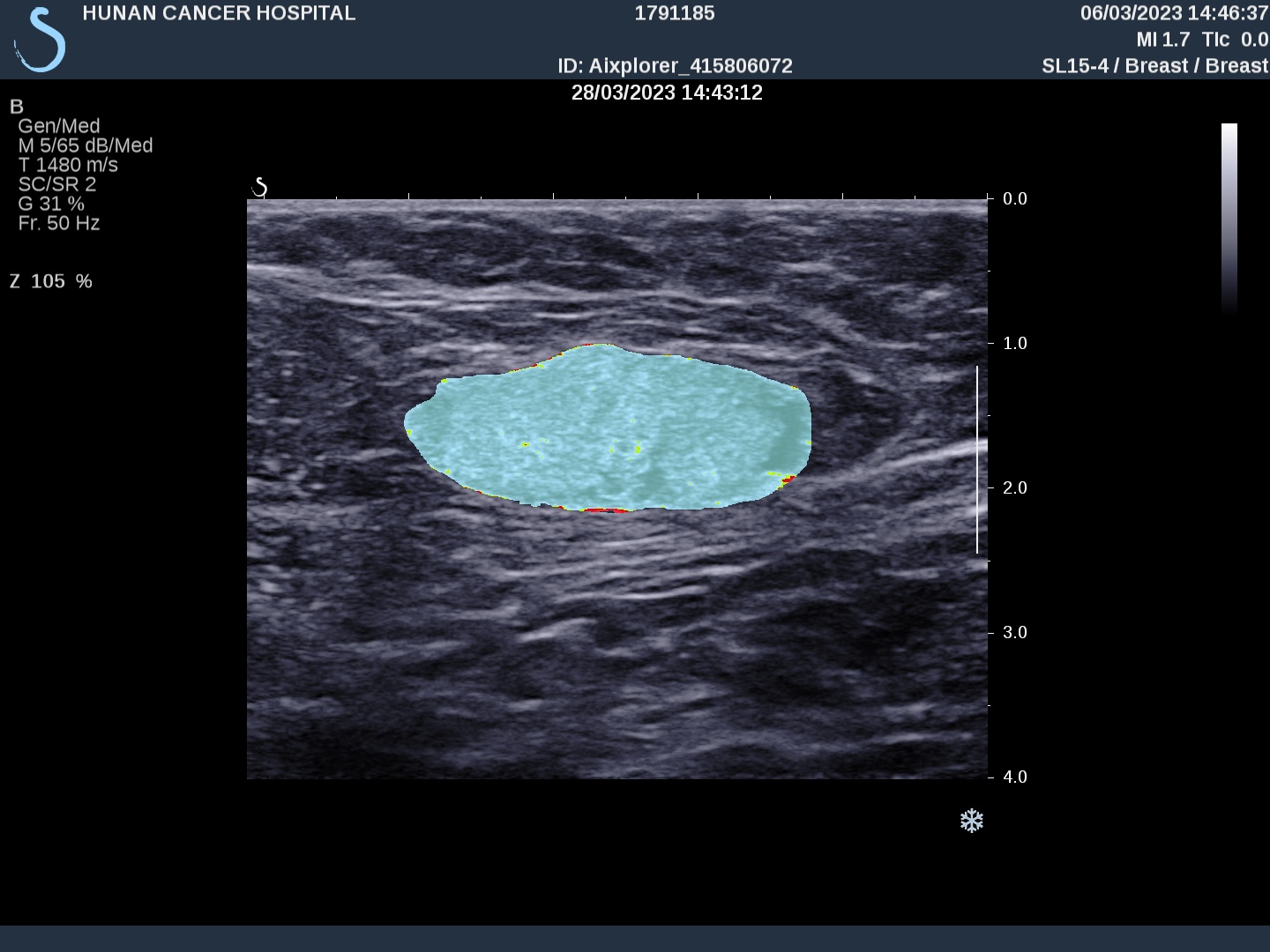

Supplement: Supplementary file 2 [file DataSheet_2.zip › ROI/1791185-1.jpg]

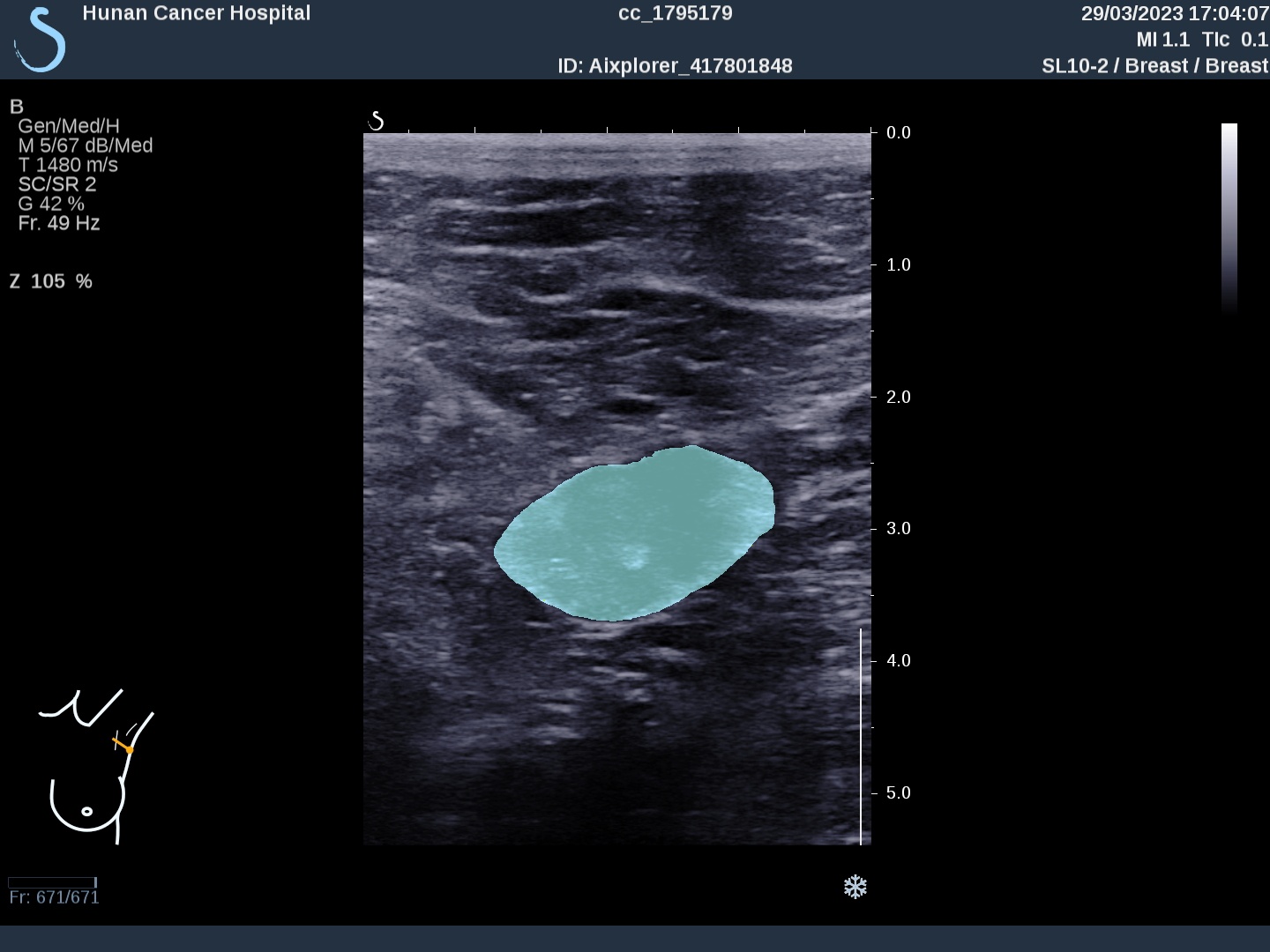

Supplement: Supplementary file 2 [file DataSheet_2.zip › ROI/1795179-1.jpg]

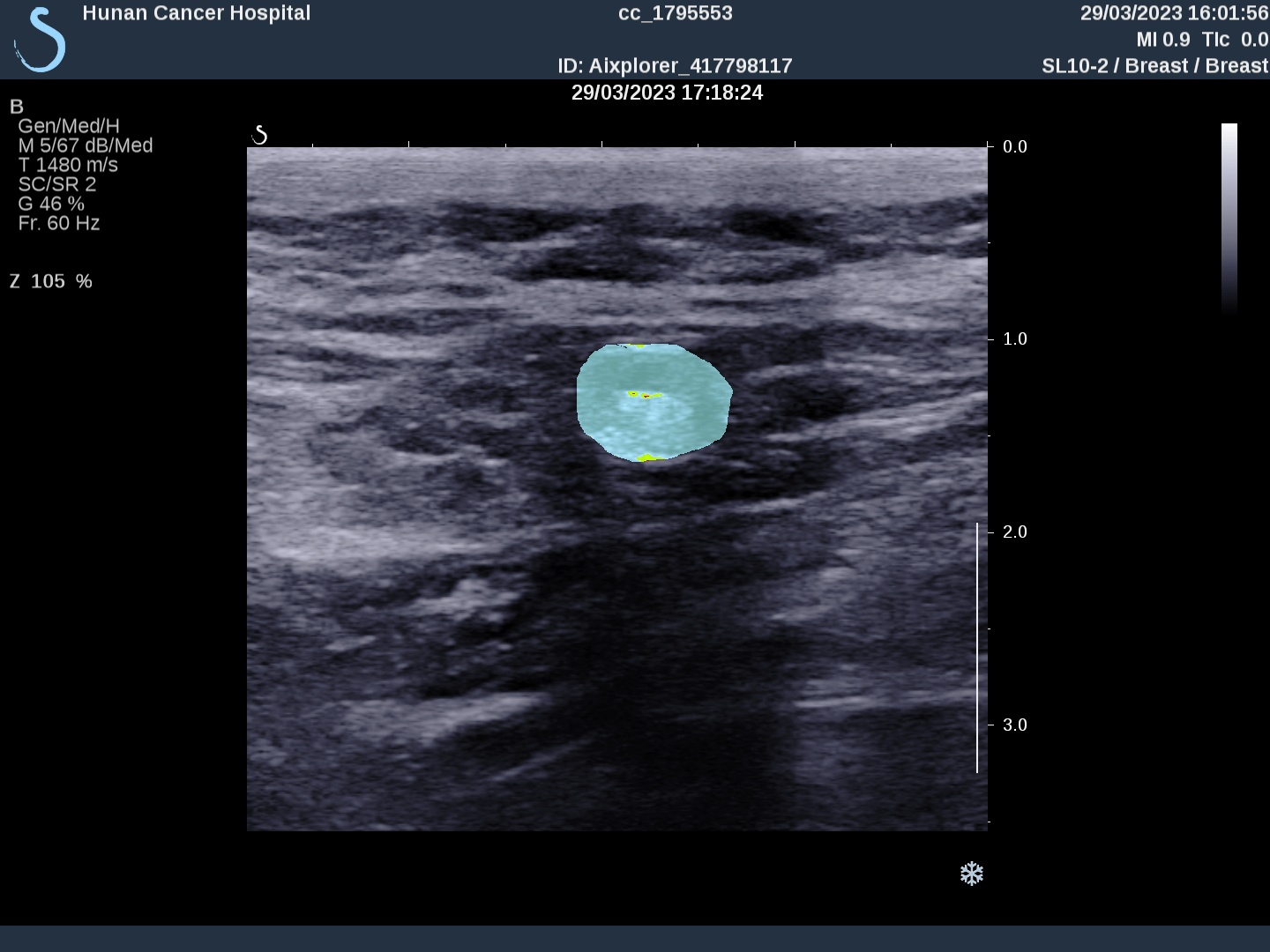

Supplement: Supplementary file 2 [file DataSheet_2.zip › ROI/1795553-1.jpg]

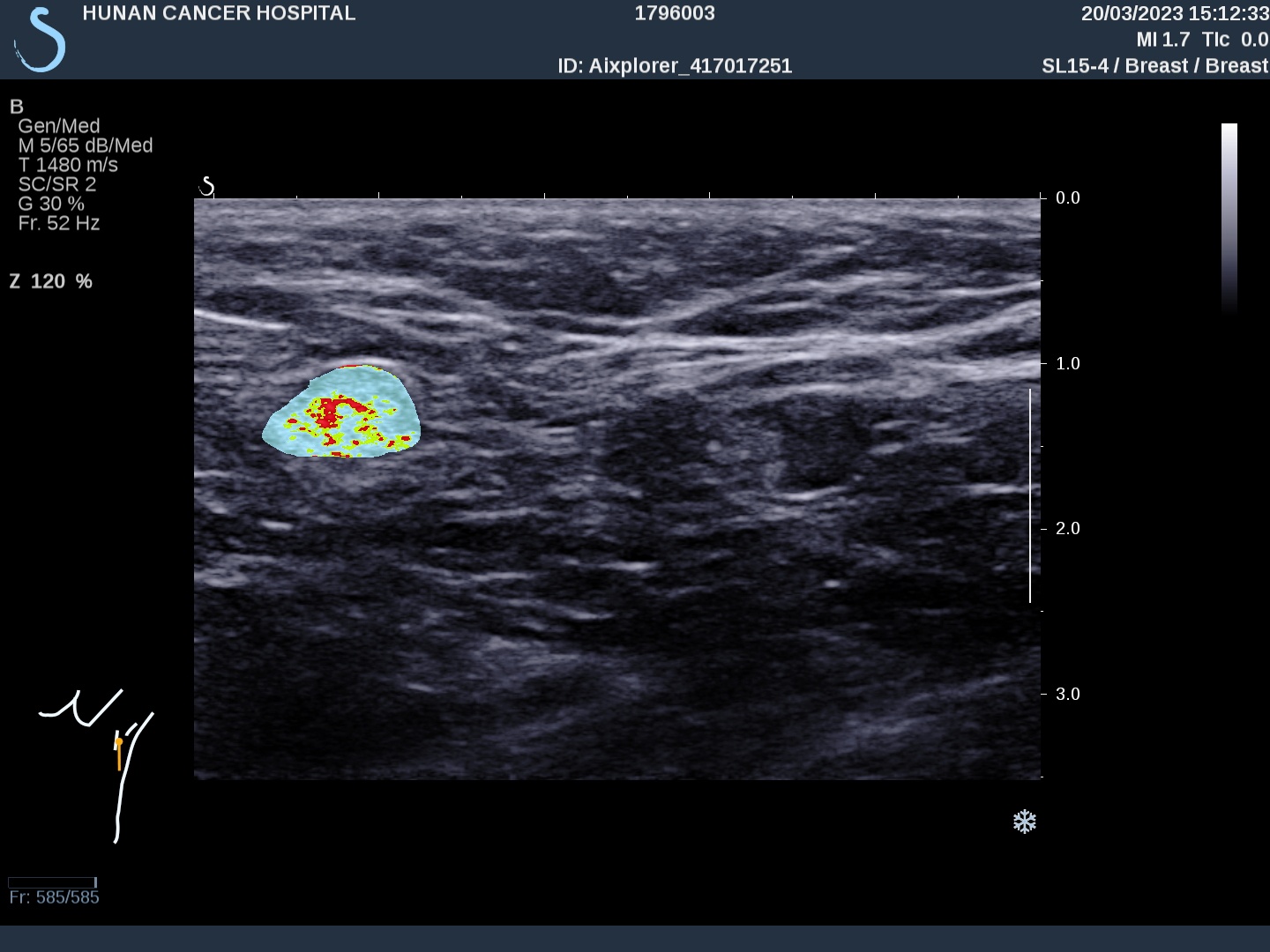

Supplement: Supplementary file 2 [file DataSheet_2.zip › ROI/1796003-1.jpg]

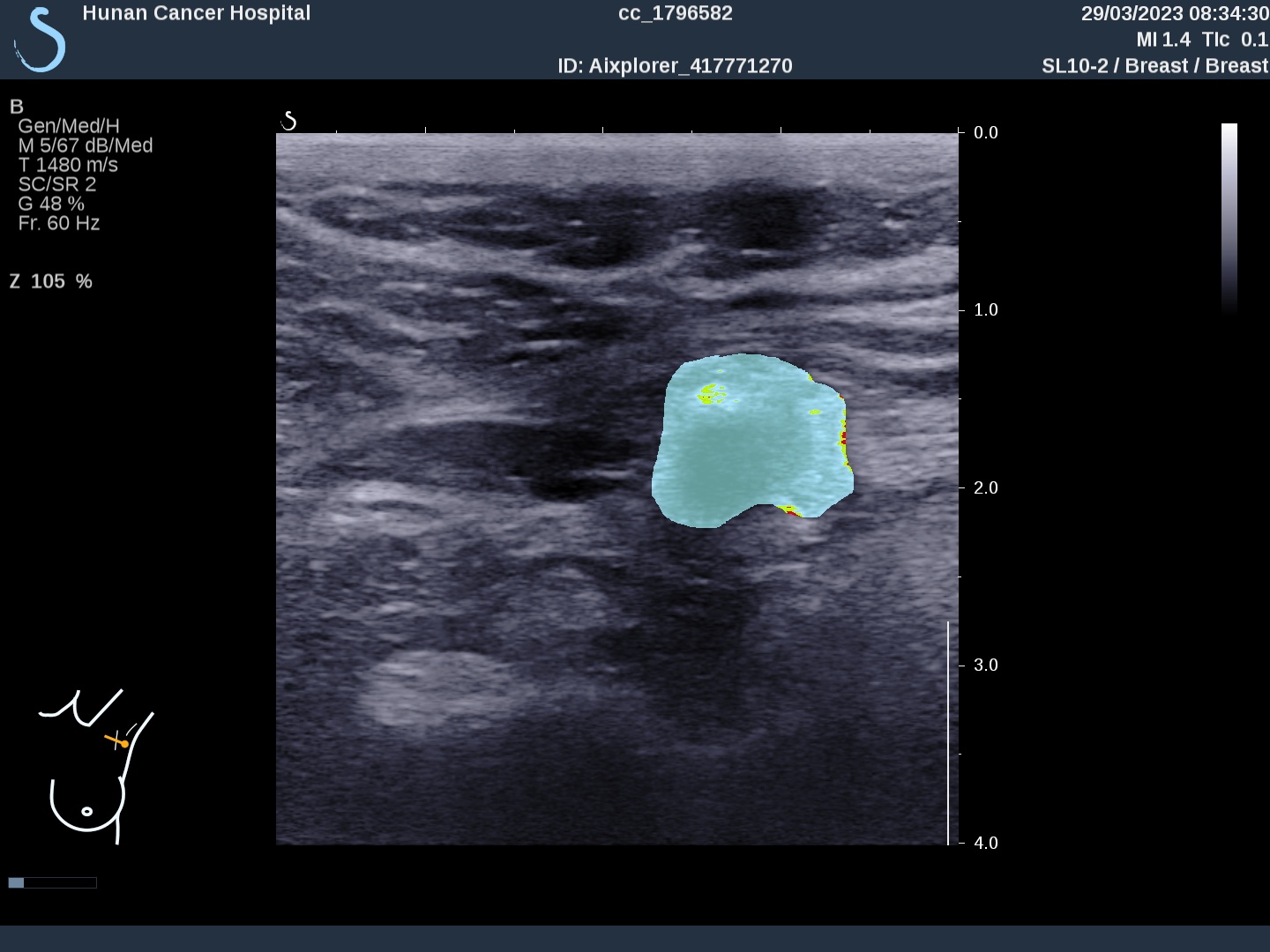

Supplement: Supplementary file 2 [file DataSheet_2.zip › ROI/1796582-1.jpg]

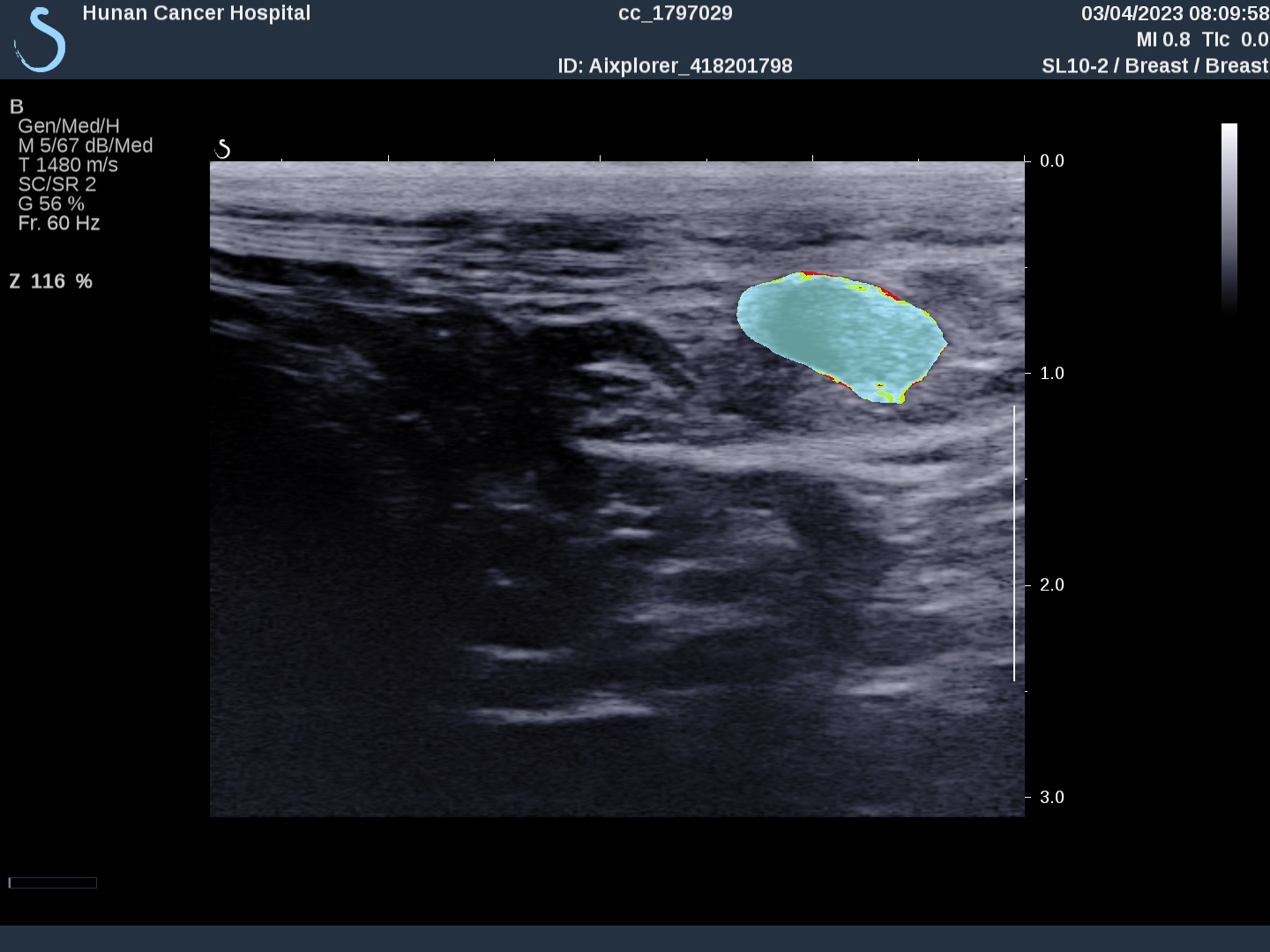

Supplement: Supplementary file 2 [file DataSheet_2.zip › ROI/1797029-1.jpg]

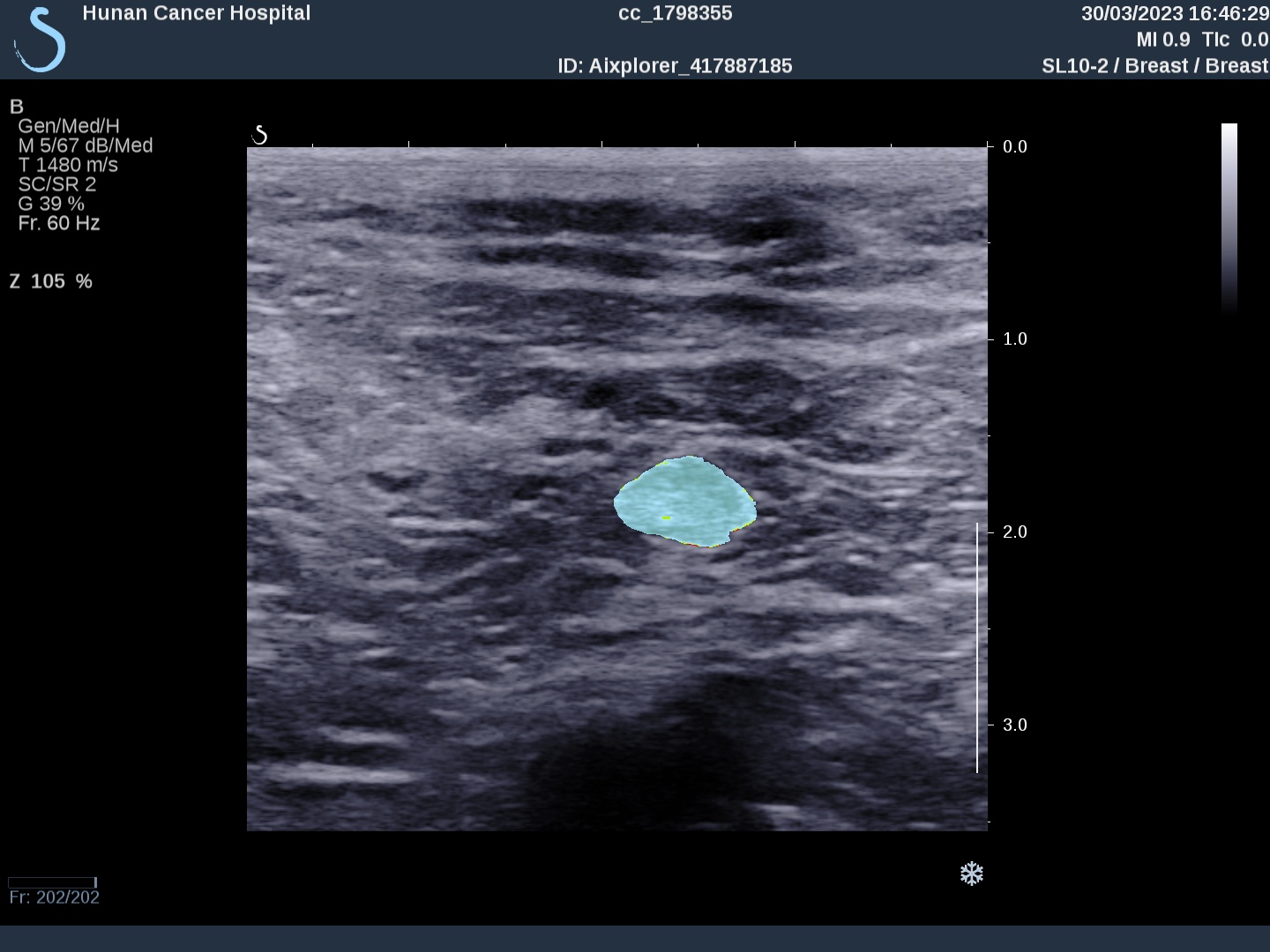

Supplement: Supplementary file 2 [file DataSheet_2.zip › ROI/1798355-1.jpg]

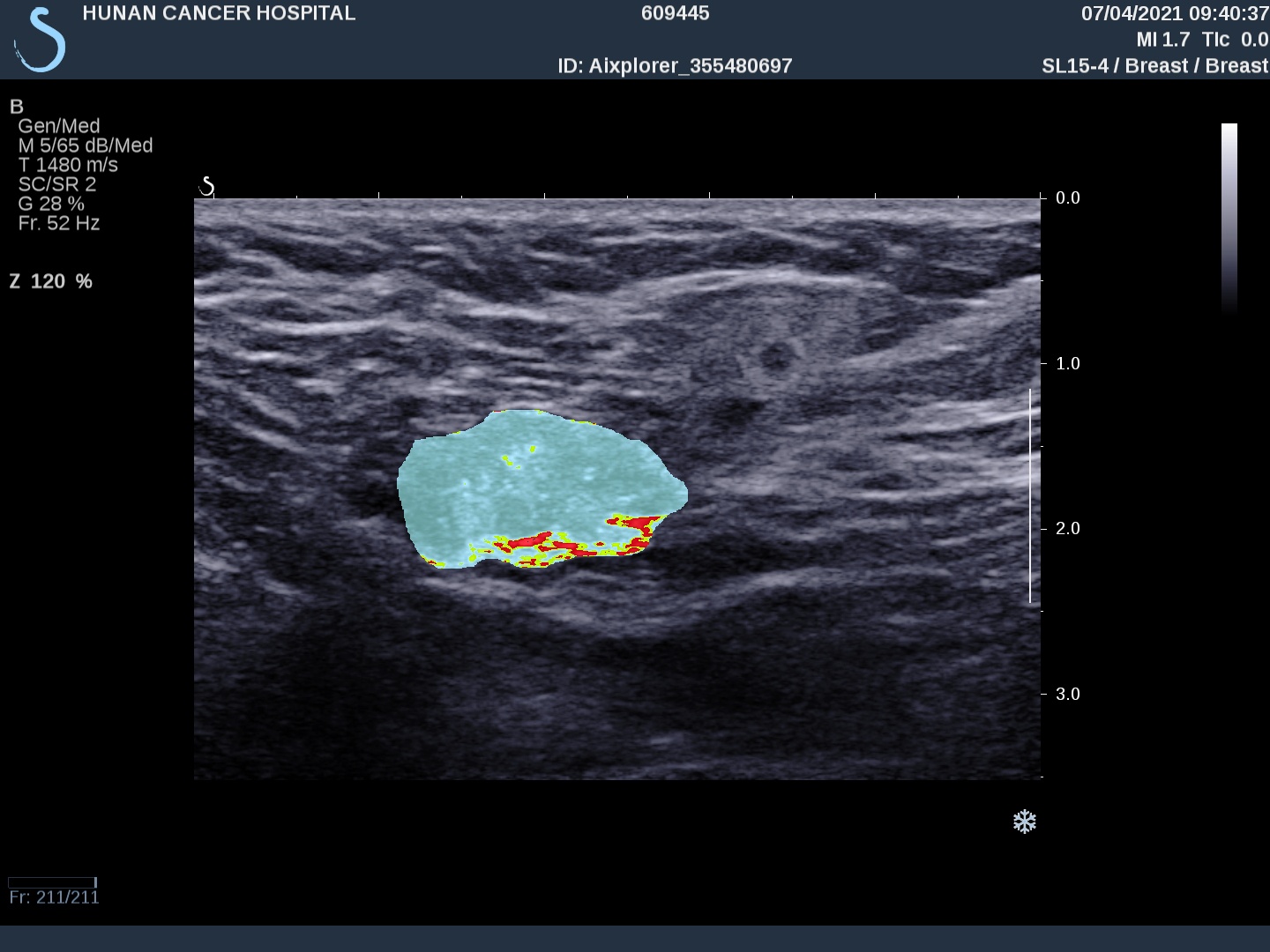

Supplement: Supplementary file 2 [file DataSheet_2.zip › ROI/609445-1.jpg]

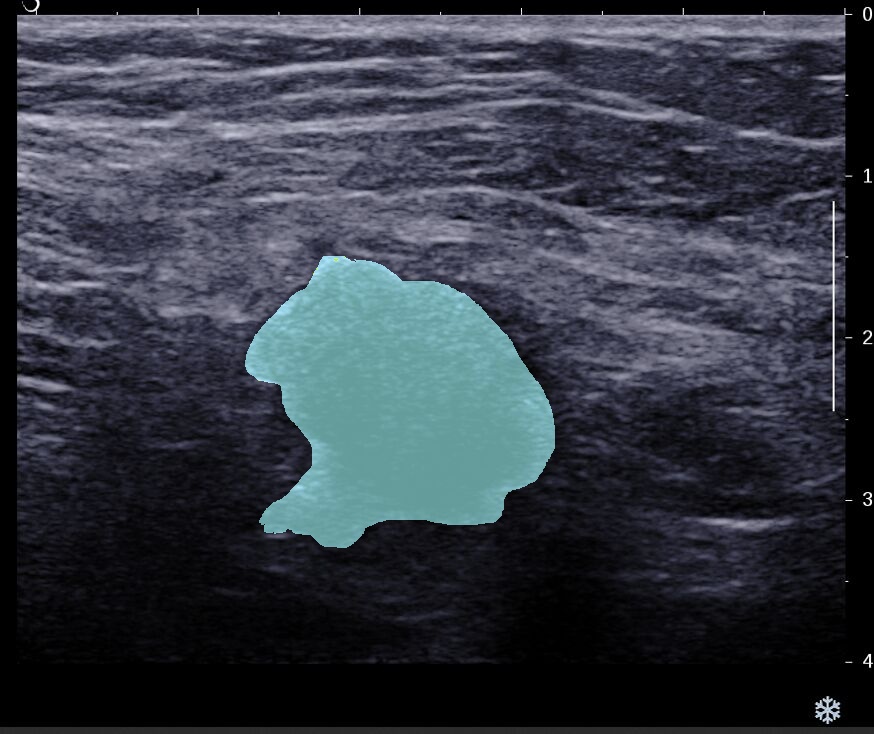

Supplement: Supplementary file 2 [file DataSheet_2.zip › ROI/773639-1.jpg]

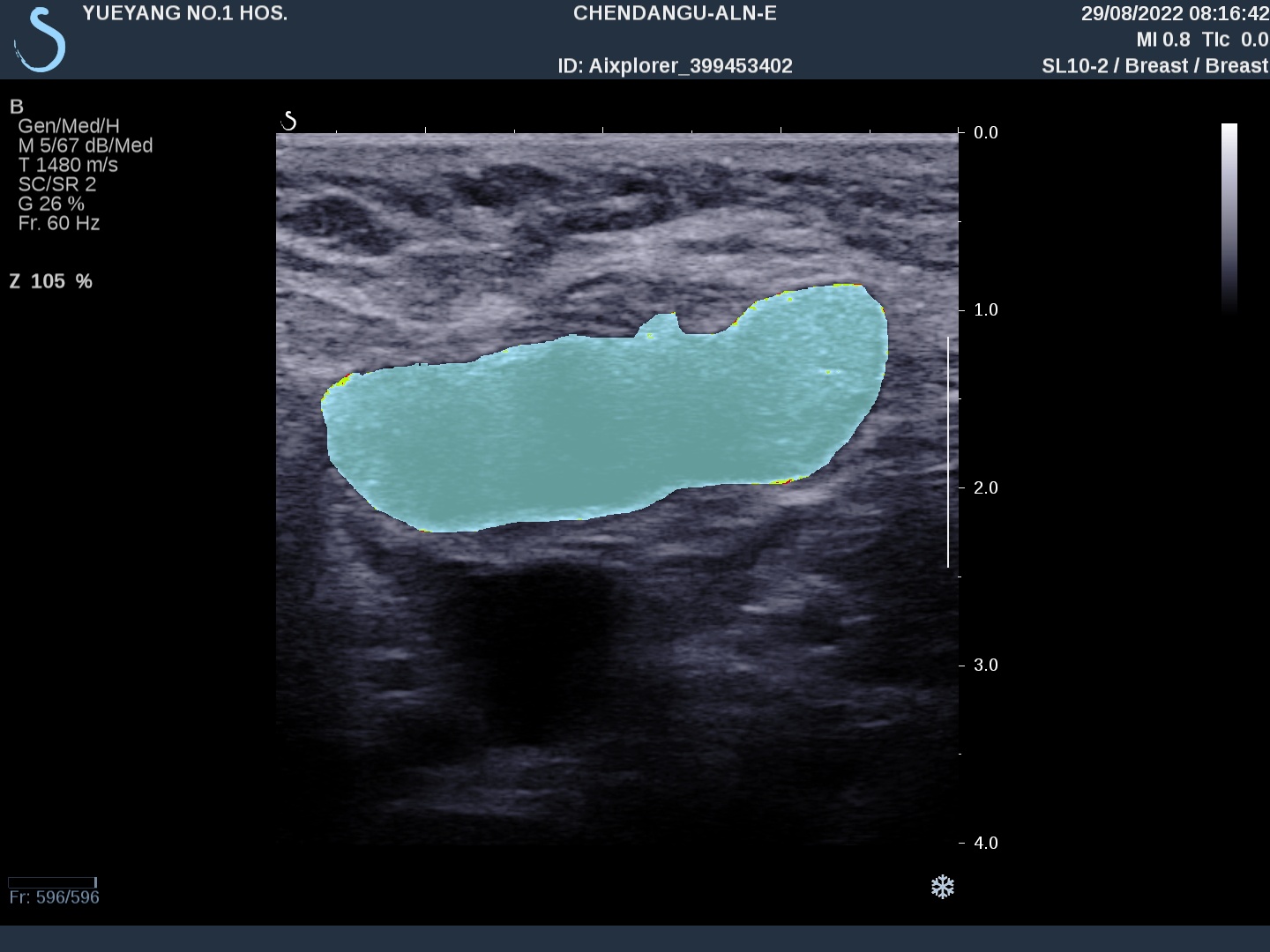

Supplement: Supplementary file 2 [file DataSheet_2.zip › ROI/chendangu-1.jpg]

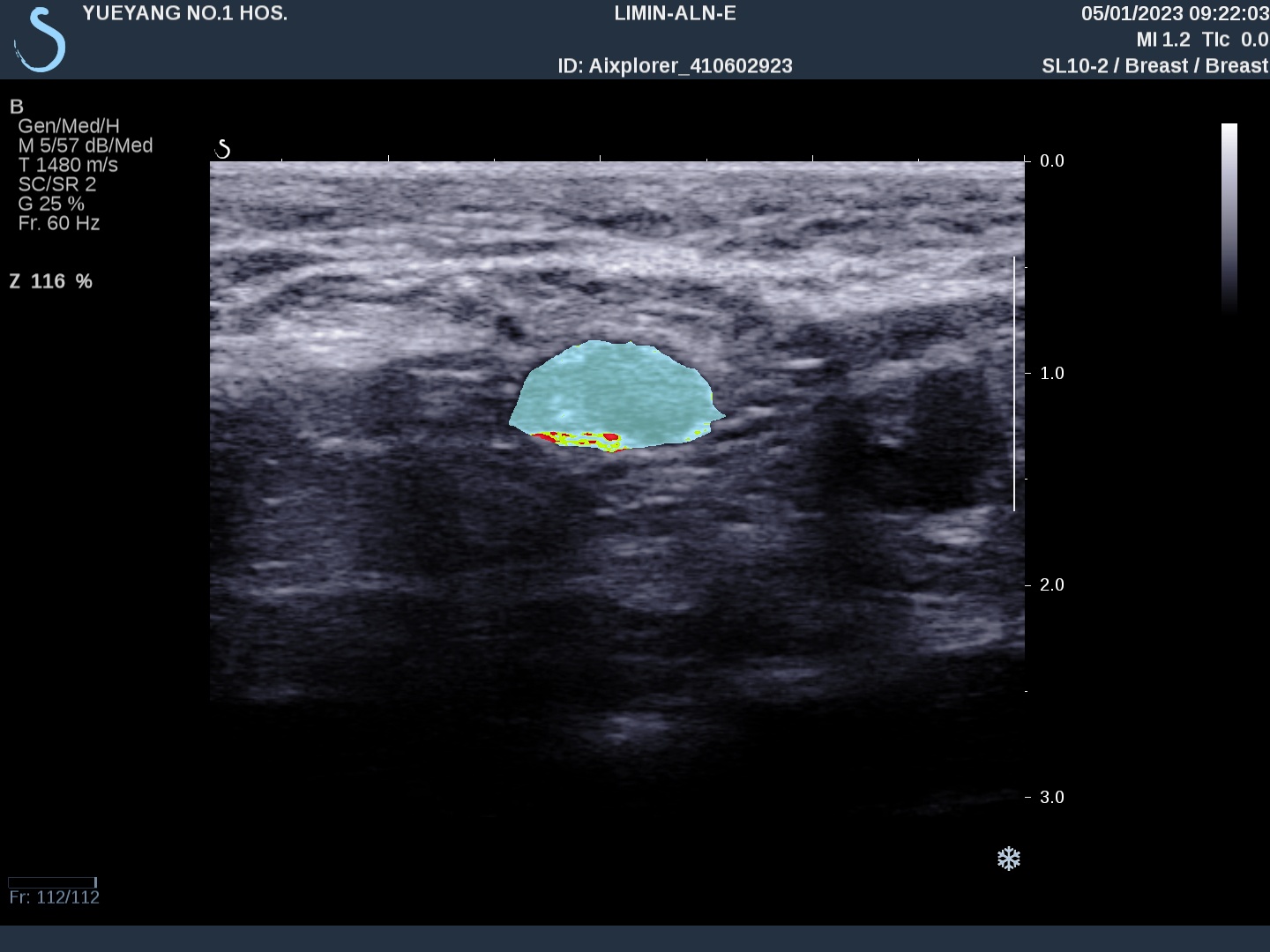

Supplement: Supplementary file 2 [file DataSheet_2.zip › ROI/limin-1.jpg]

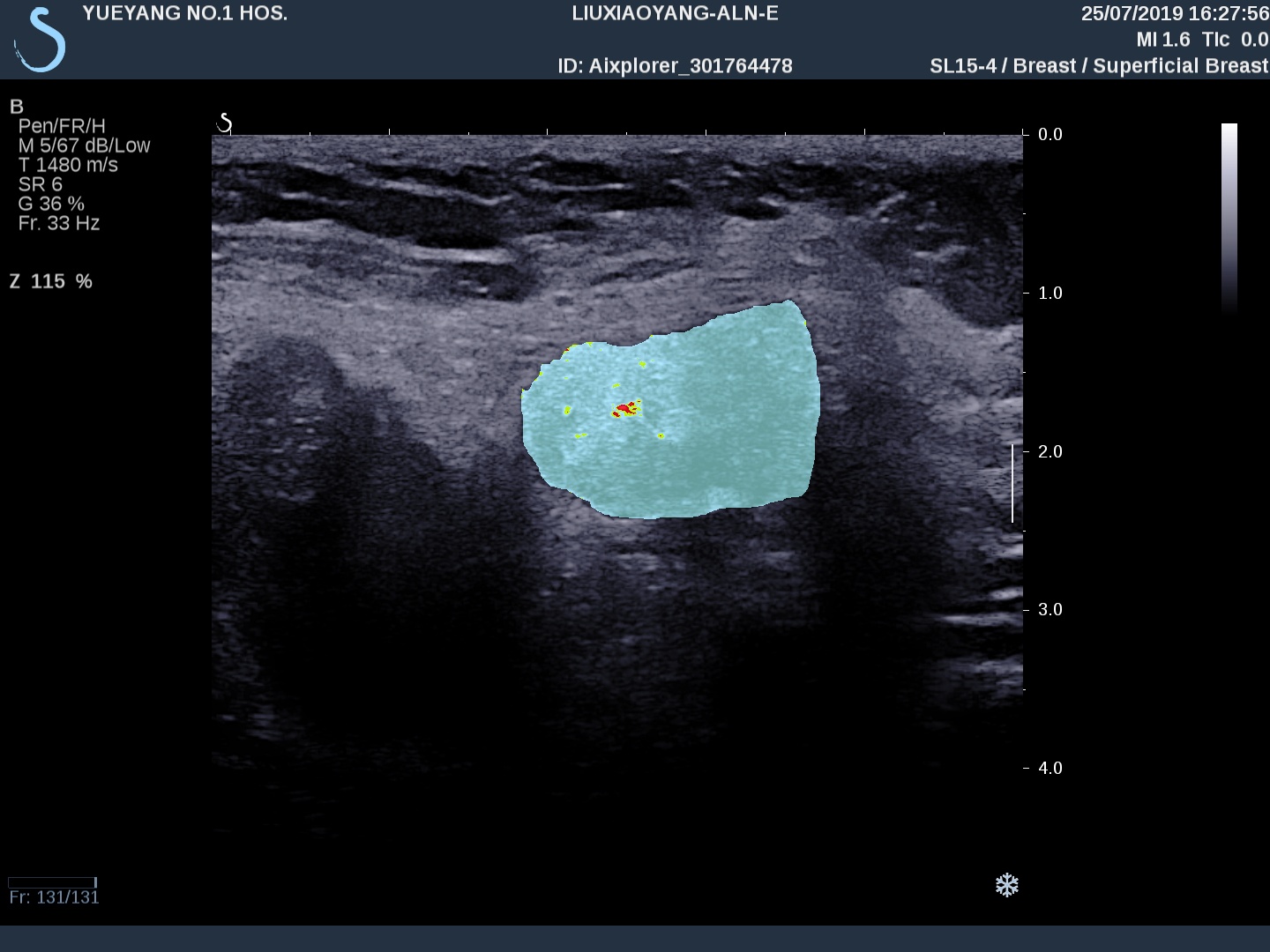

Supplement: Supplementary file 2 [file DataSheet_2.zip › ROI/liuxiaoyang-1.jpg]

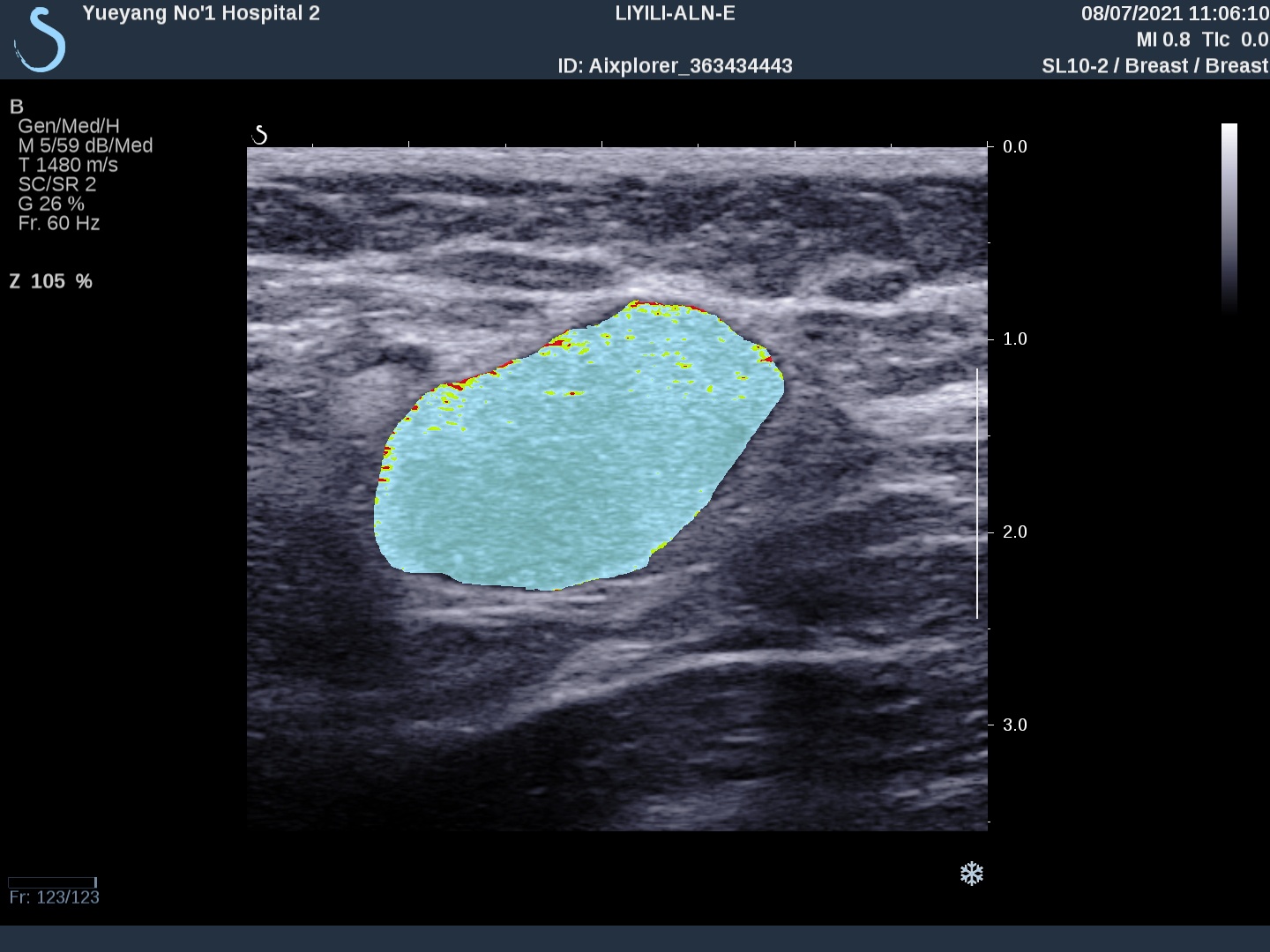

Supplement: Supplementary file 2 [file DataSheet_2.zip › ROI/liyili-1.jpg]

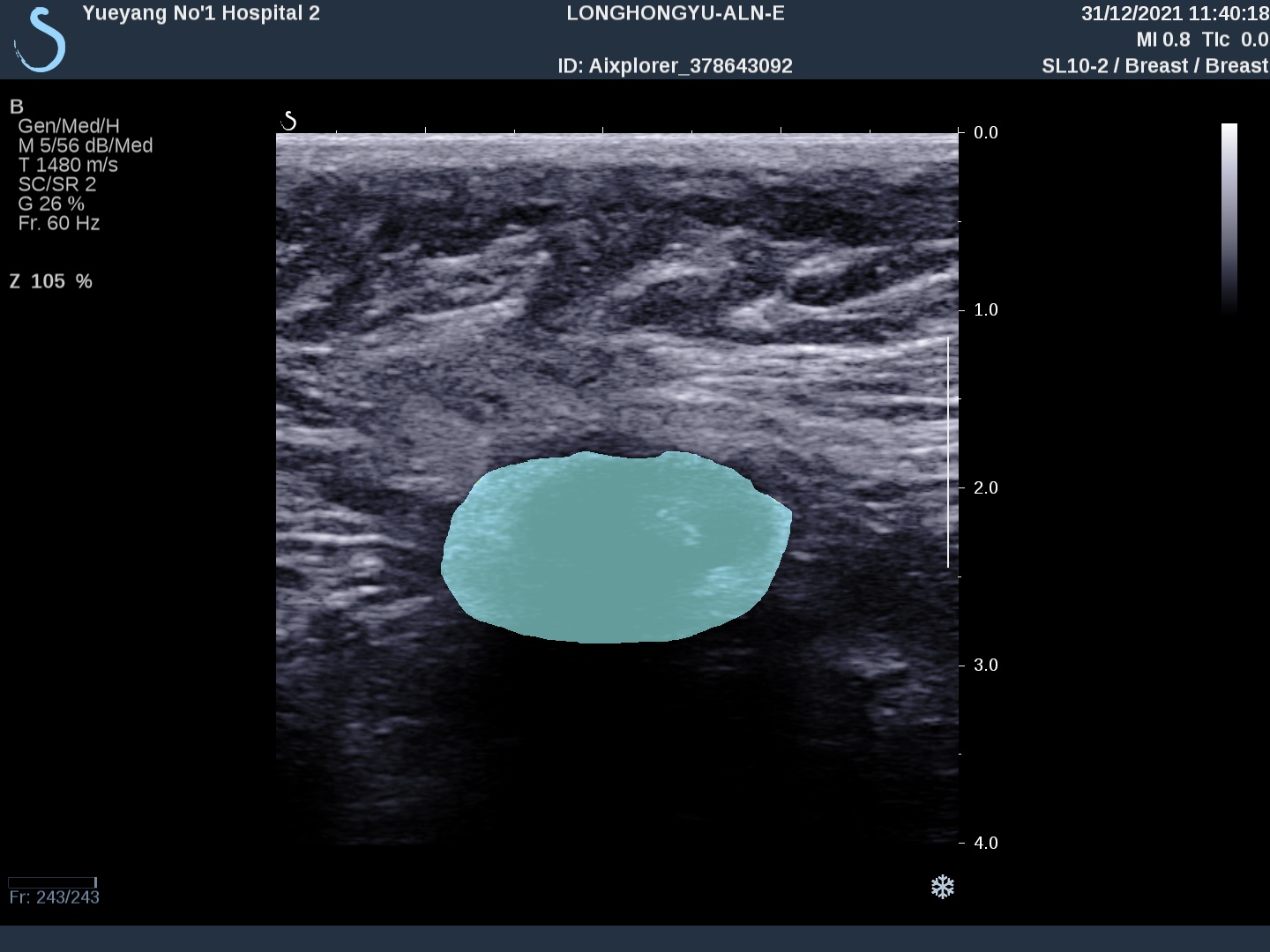

Supplement: Supplementary file 2 [file DataSheet_2.zip › ROI/longhongyu-1.jpg]

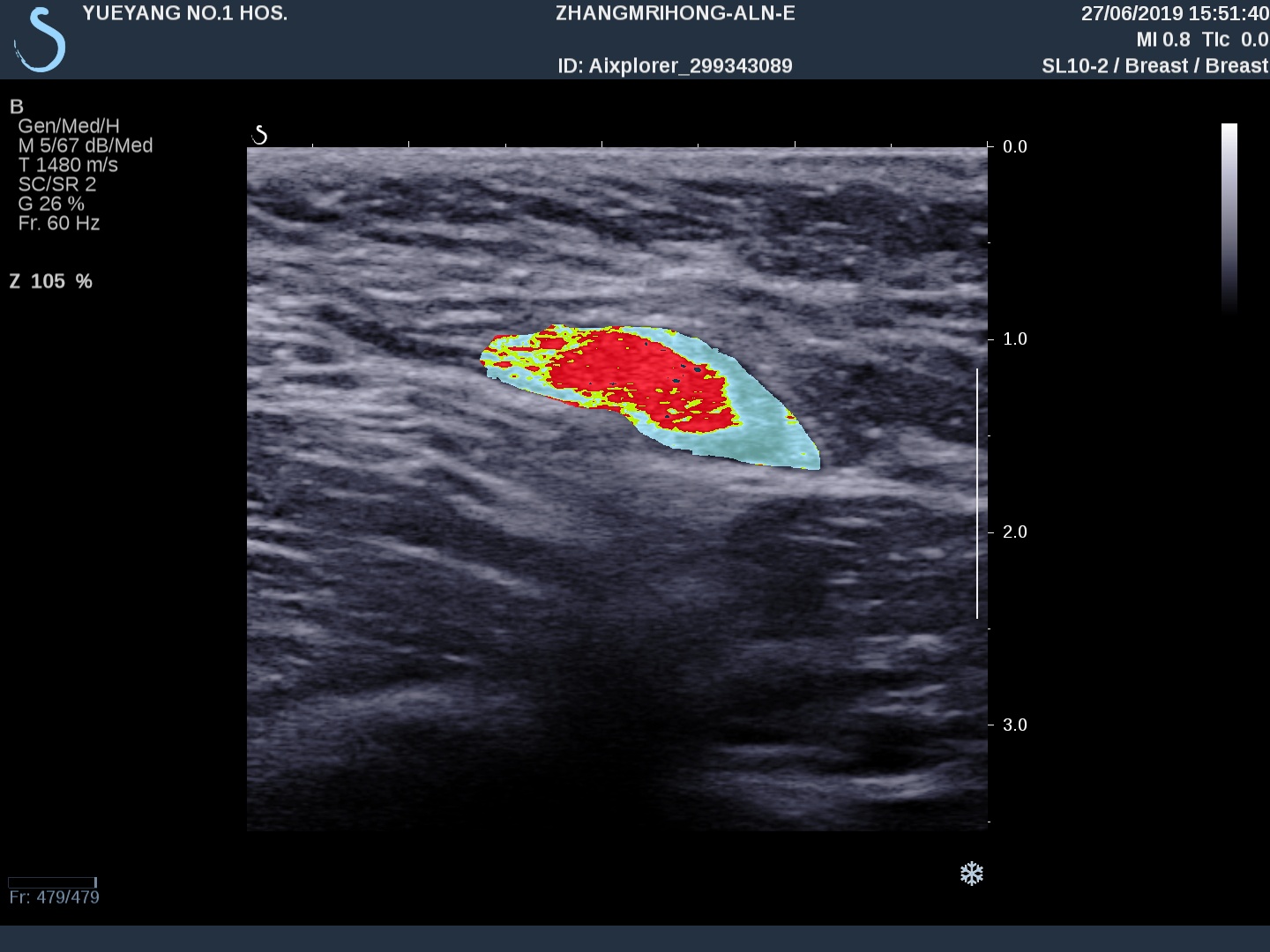

Supplement: Supplementary file 2 [file DataSheet_2.zip › ROI/zhangmeihong-1.jpg]

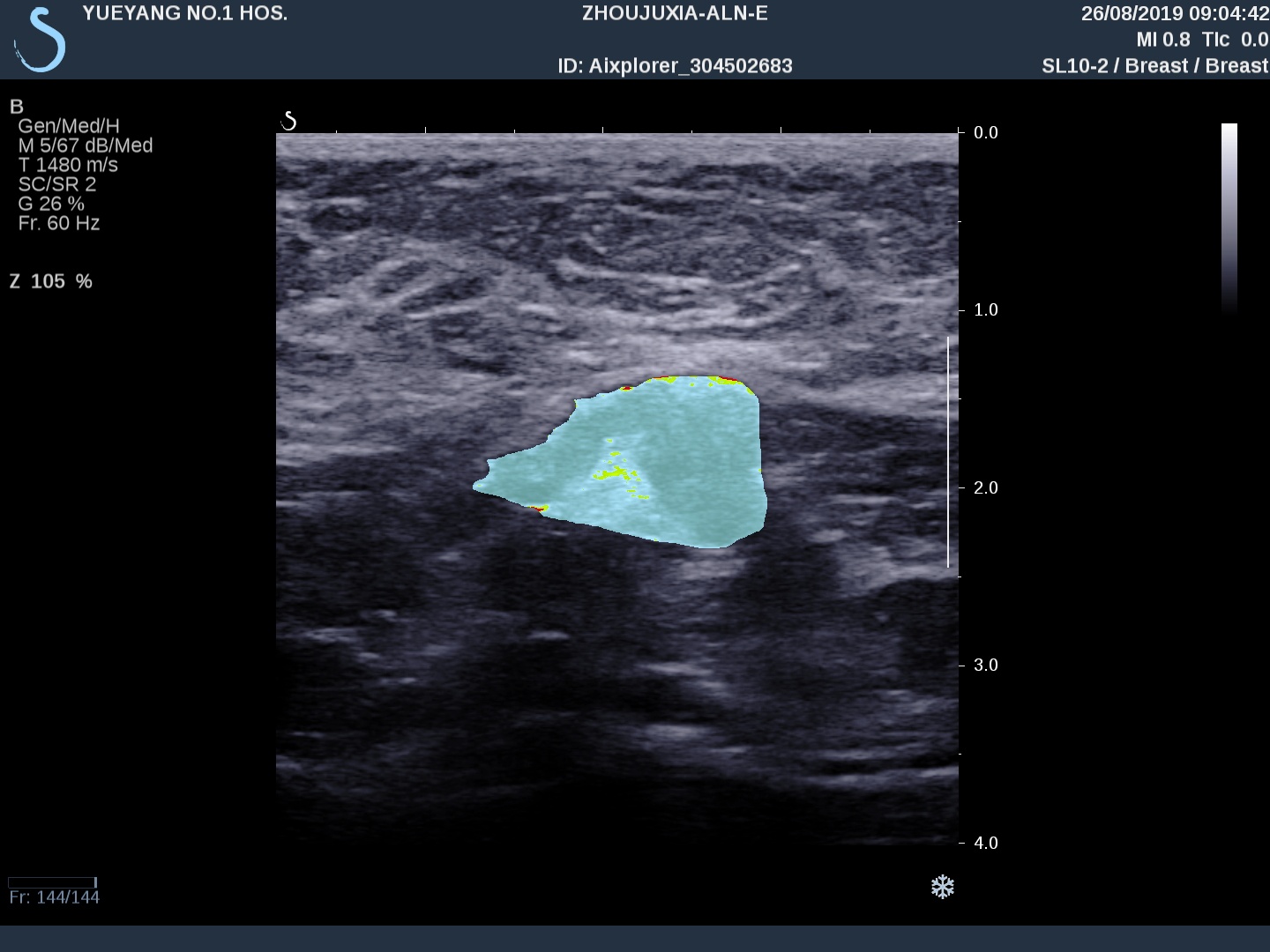

Supplement: Supplementary file 2 [file DataSheet_2.zip › ROI/zhoujuxia-1.jpg]

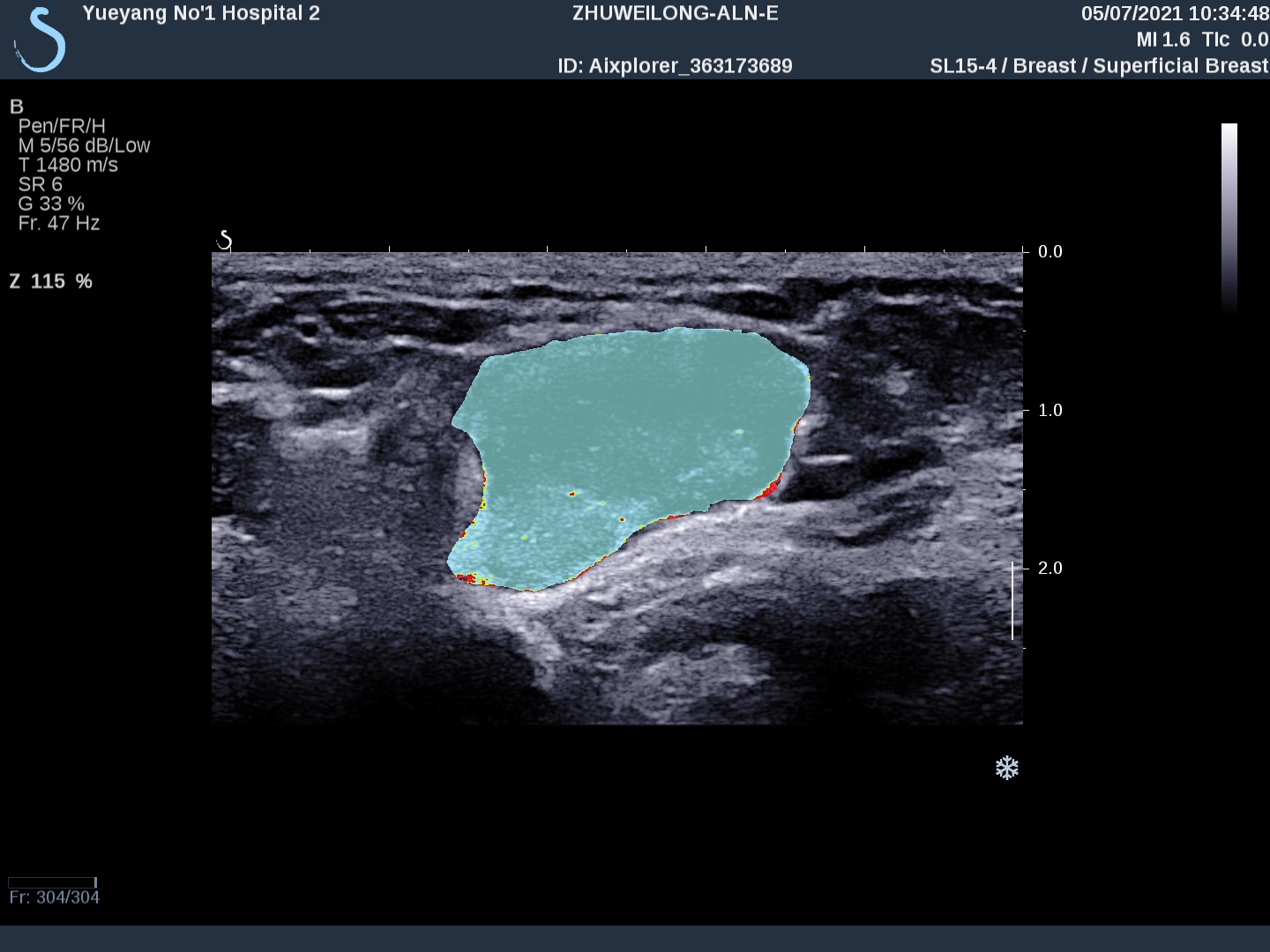

Supplement: Supplementary file 2 [file DataSheet_2.zip › ROI/zhuweilong-1.jpg]
