# Supplementary figures and images for: Ultrasound radiomics based on axillary lymph nodes images for predicting lymph node metastasis in breast cancer (part 1 of 2)
Source: Front Oncol. 2023 Oct 26;13:1217309. doi: 10.3389/fonc.2023.1217309 (PMC10641324; doi:10.3389/fonc.2023.1217309)

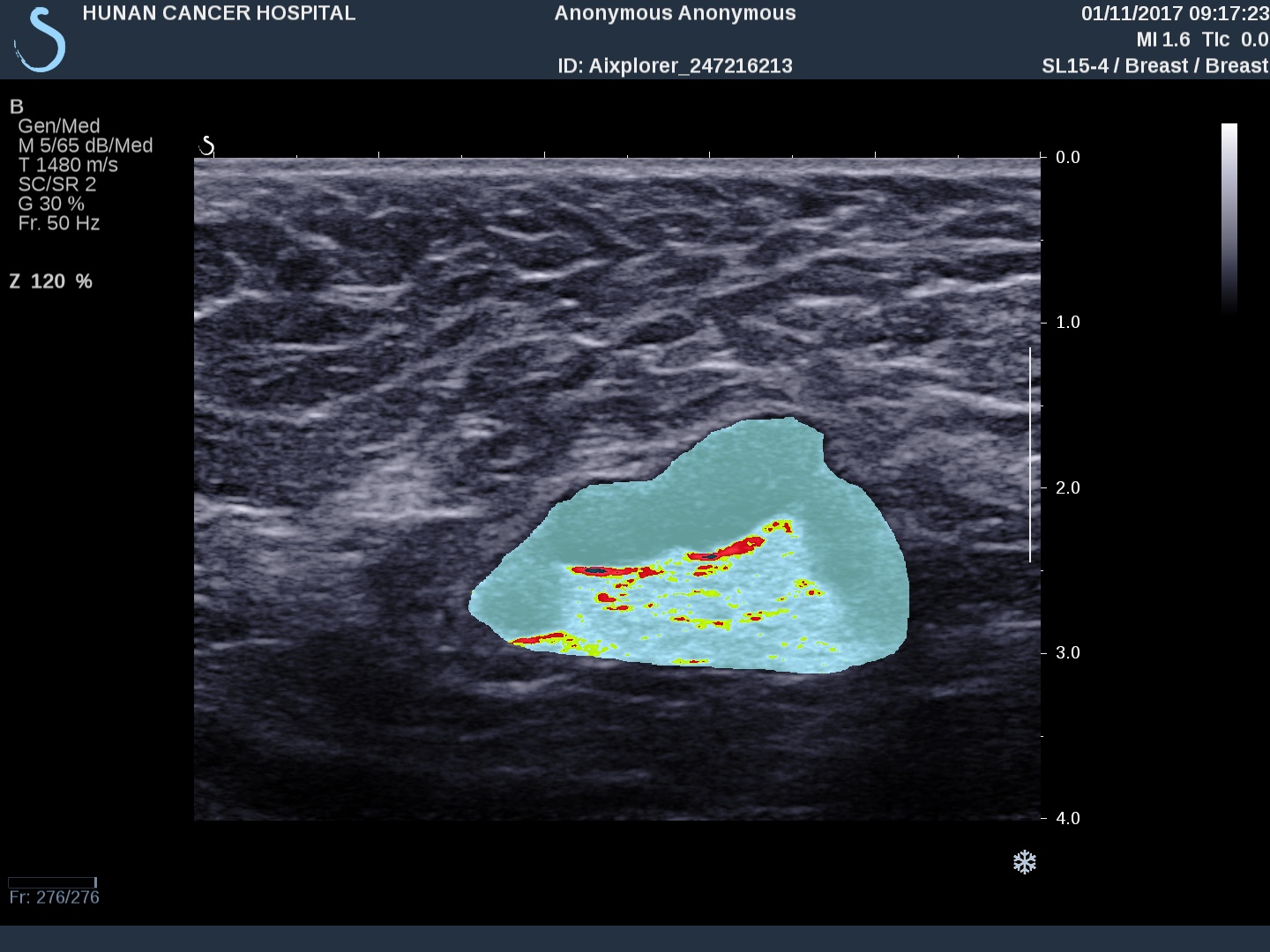

Supplement: Supplementary file 2 [file DataSheet_2.zip › ROI/1007491-3.jpg]

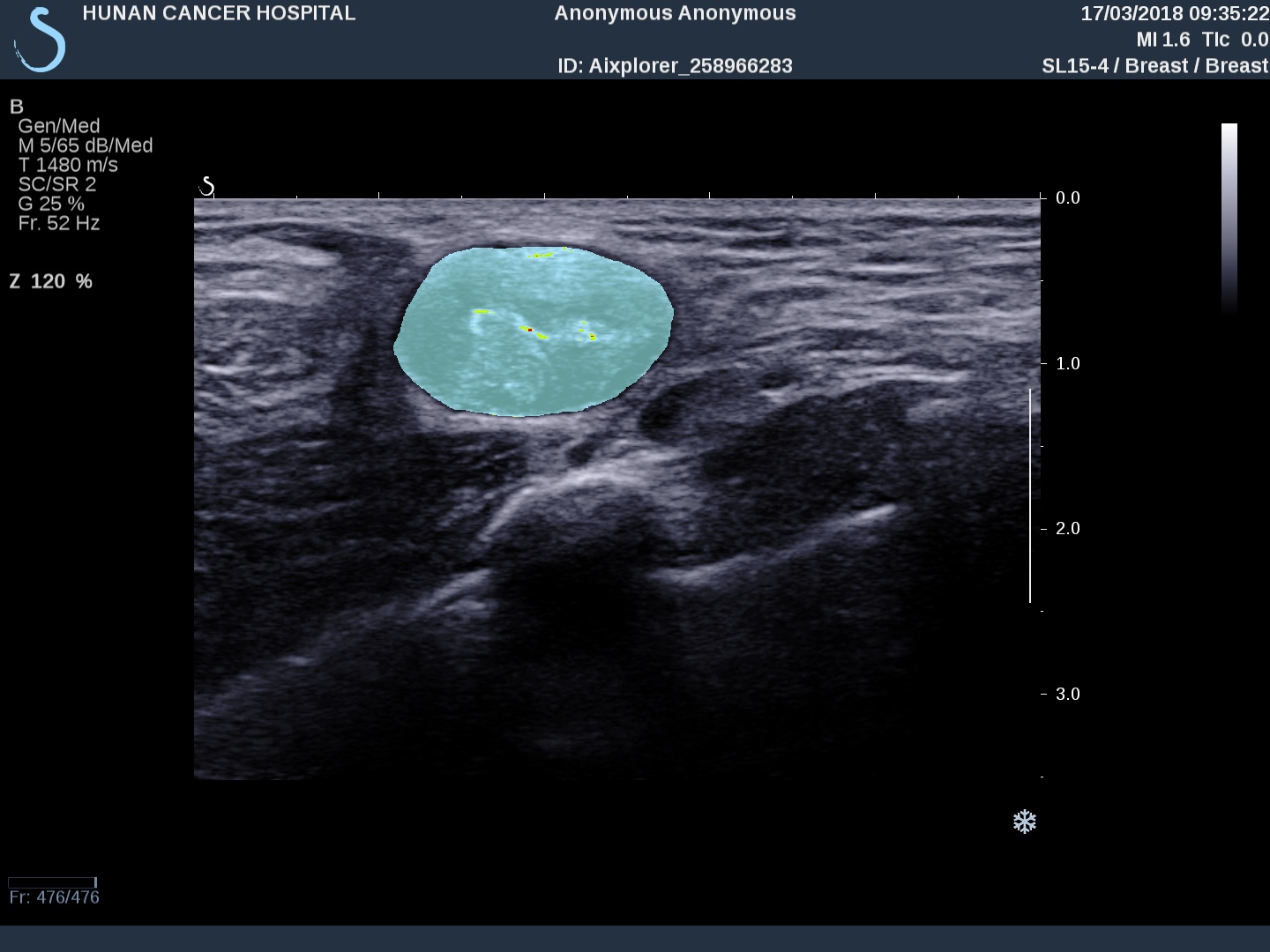

Supplement: Supplementary file 2 [file DataSheet_2.zip › ROI/1038429-1.jpg]

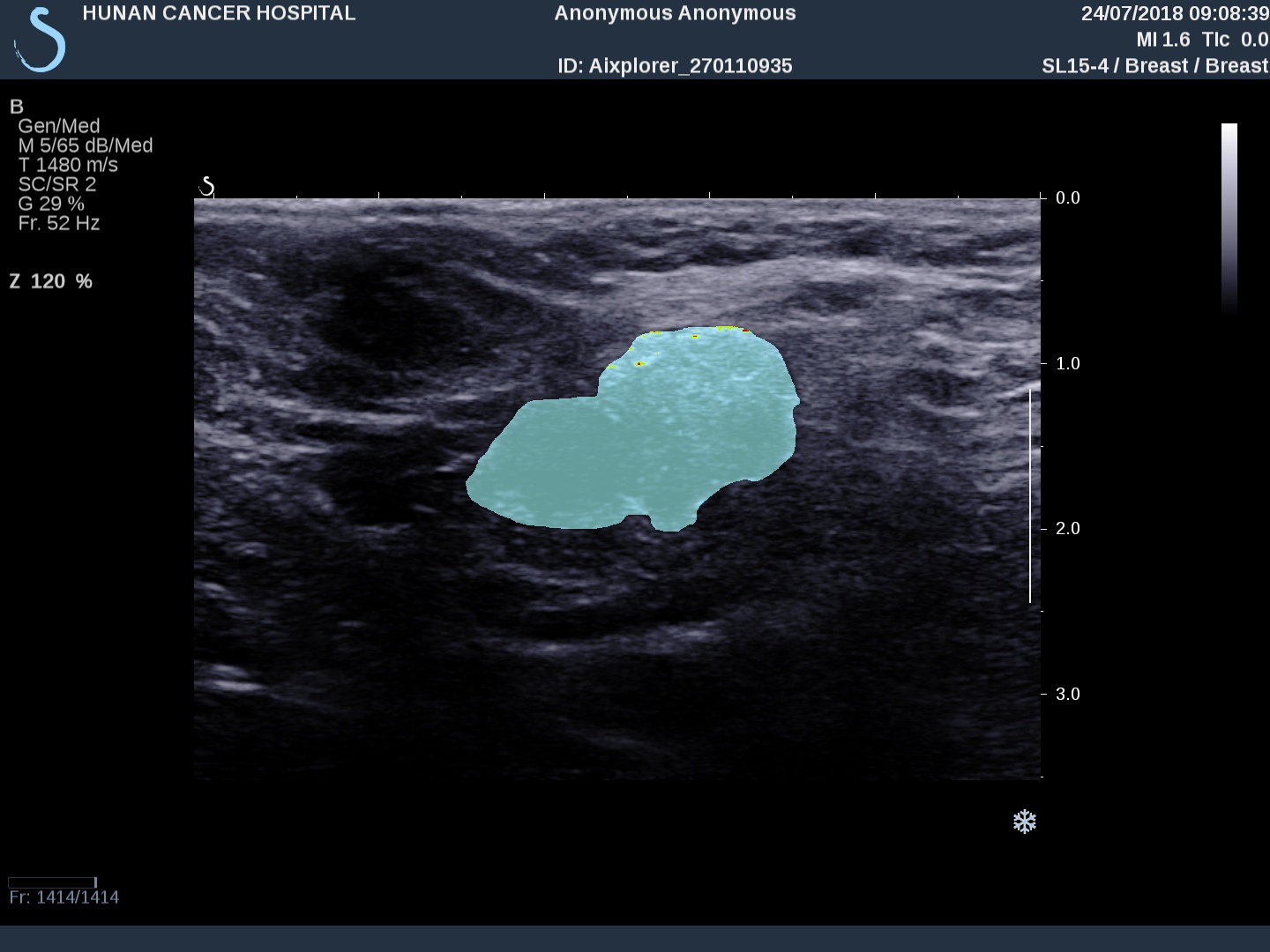

Supplement: Supplementary file 2 [file DataSheet_2.zip › ROI/1083040-1.jpg]

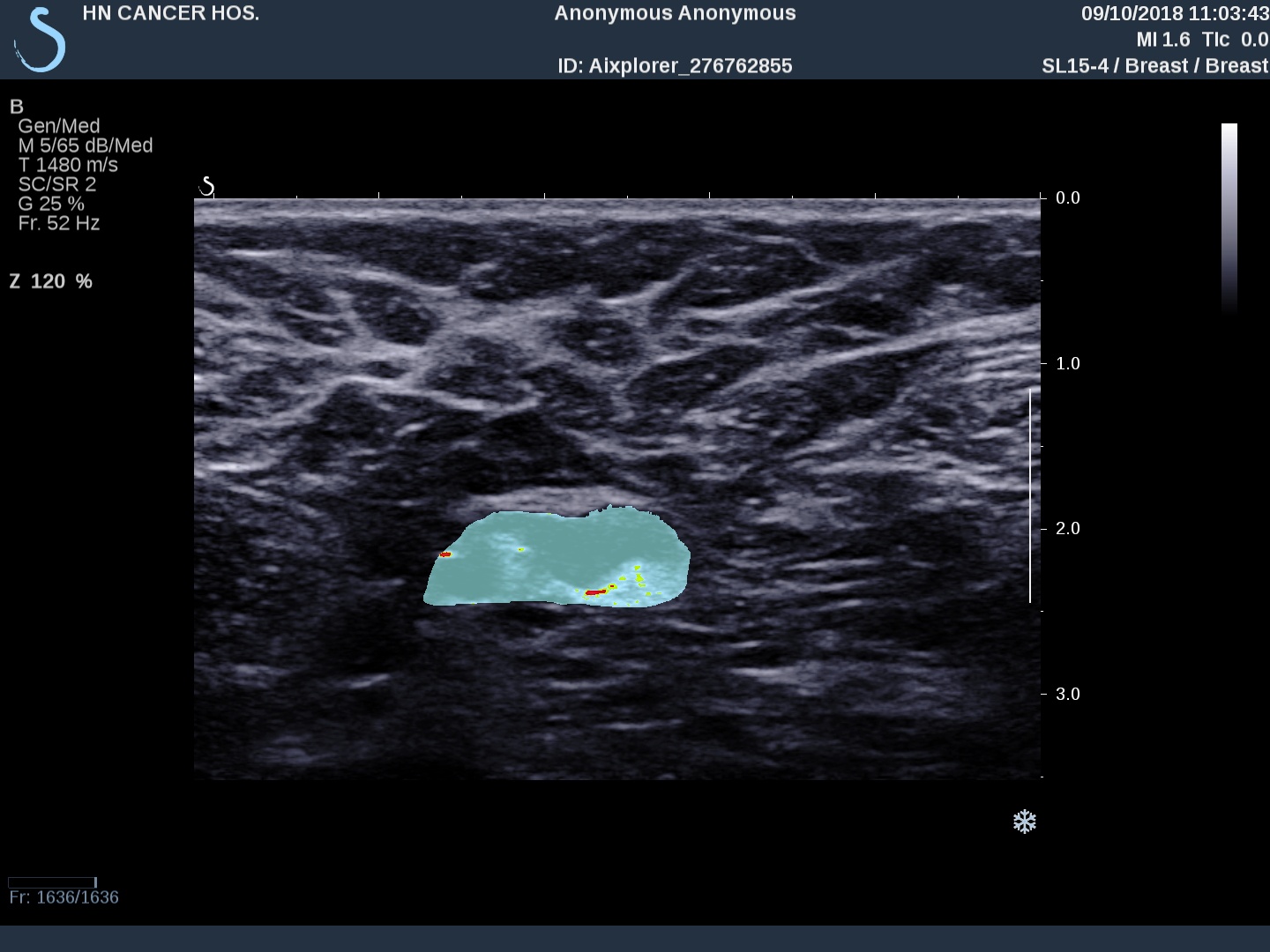

Supplement: Supplementary file 2 [file DataSheet_2.zip › ROI/1104521-1.jpg]

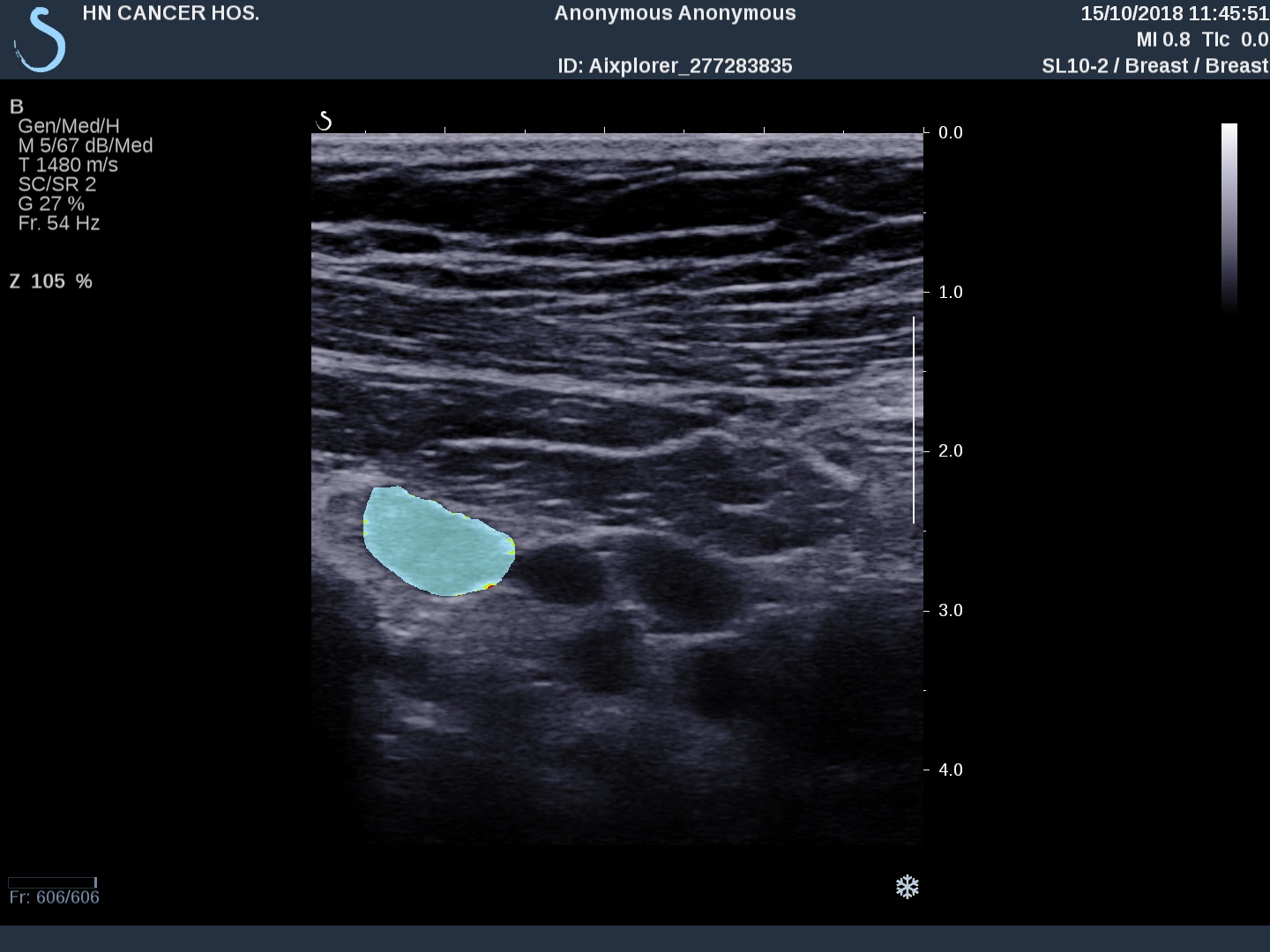

Supplement: Supplementary file 2 [file DataSheet_2.zip › ROI/1105802-1.jpg]

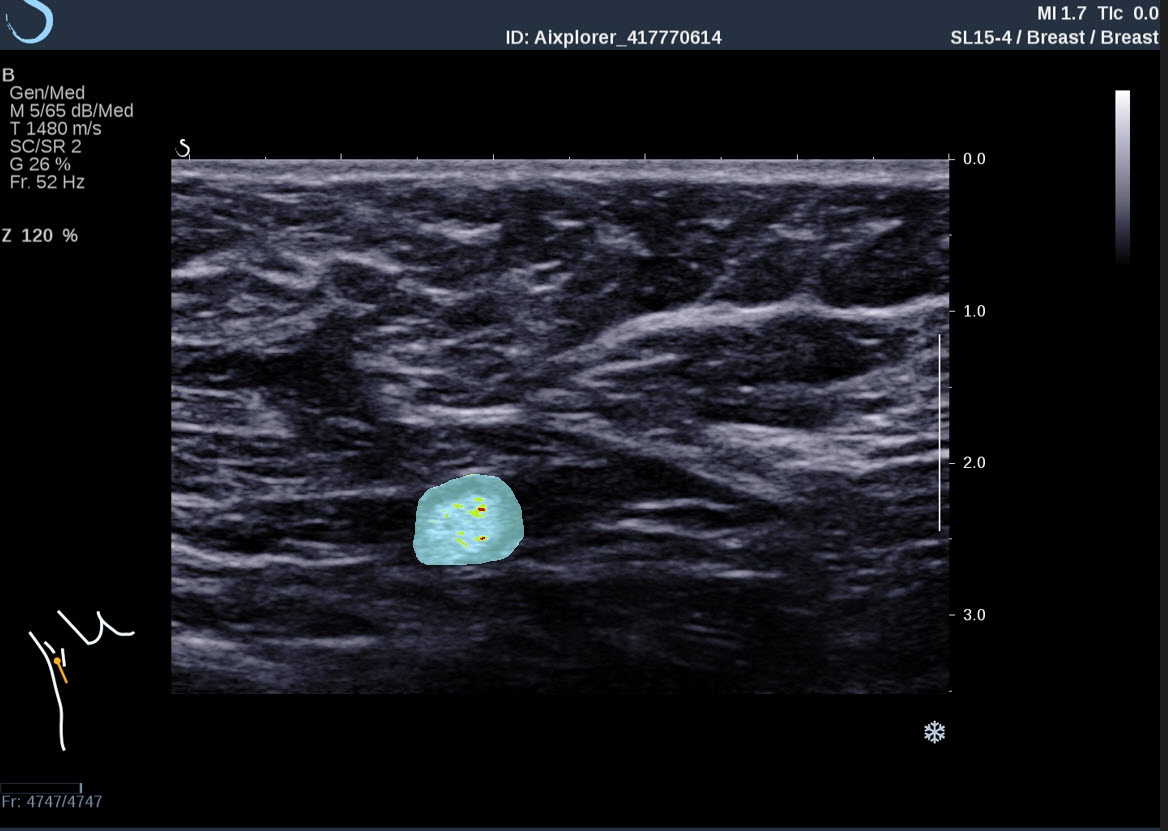

Supplement: Supplementary file 2 [file DataSheet_2.zip › ROI/1113968-1.jpg]

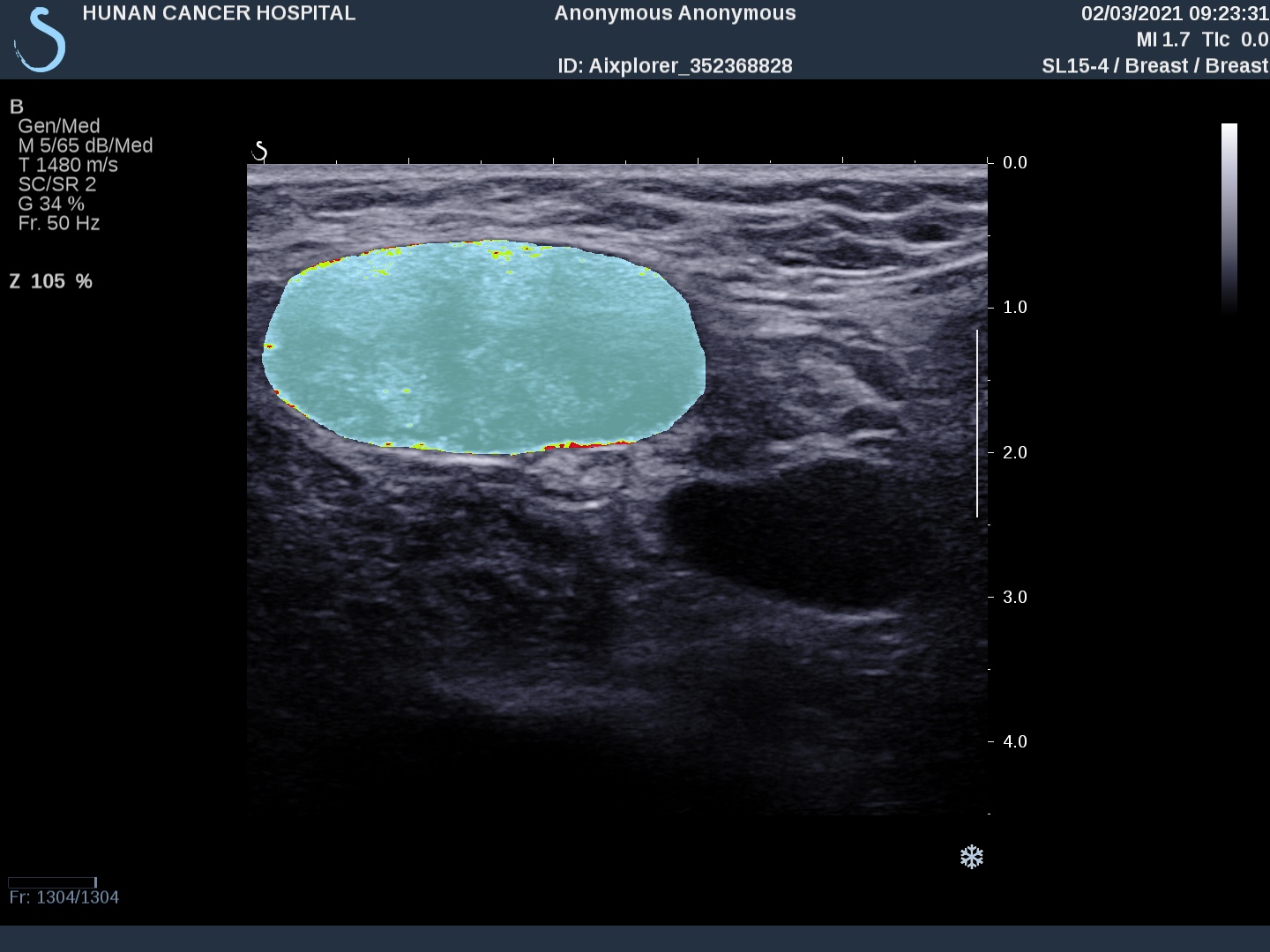

Supplement: Supplementary file 2 [file DataSheet_2.zip › ROI/1116690-1.jpg]

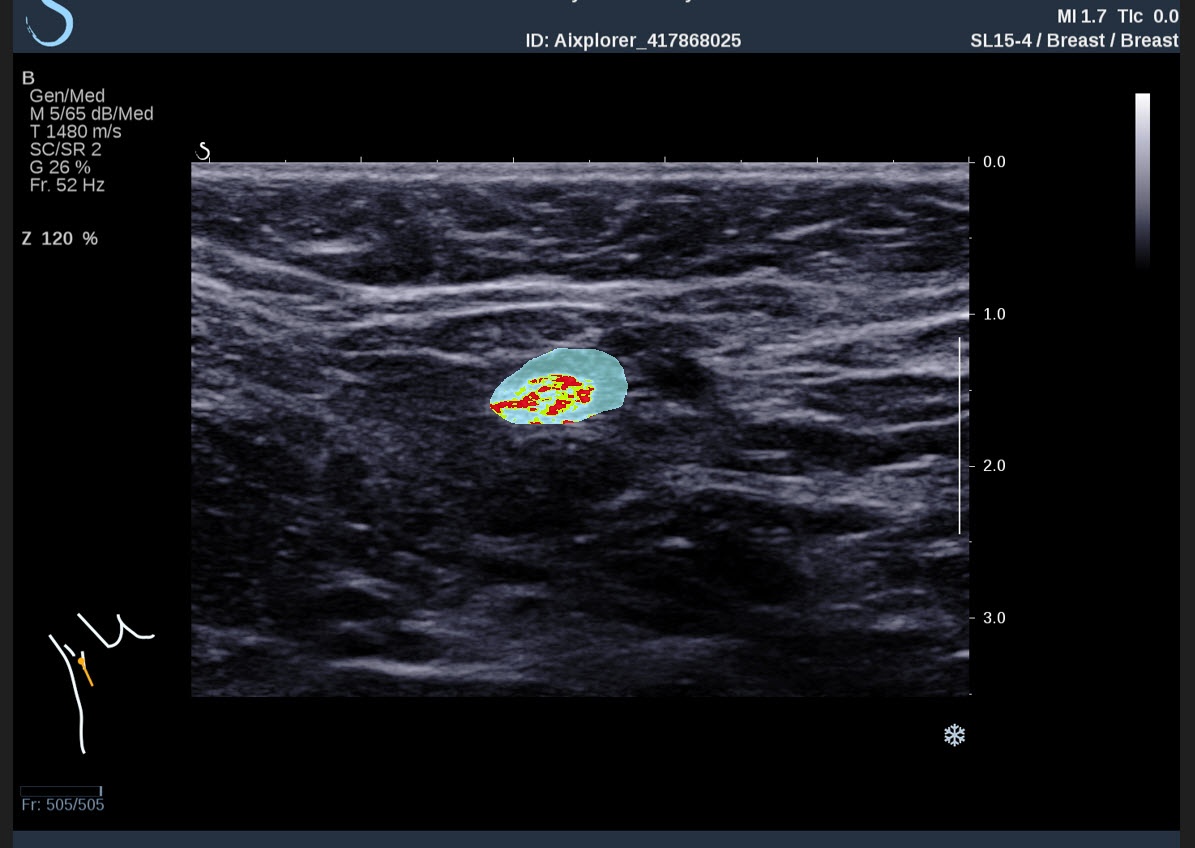

Supplement: Supplementary file 2 [file DataSheet_2.zip › ROI/1118867-1.jpg]

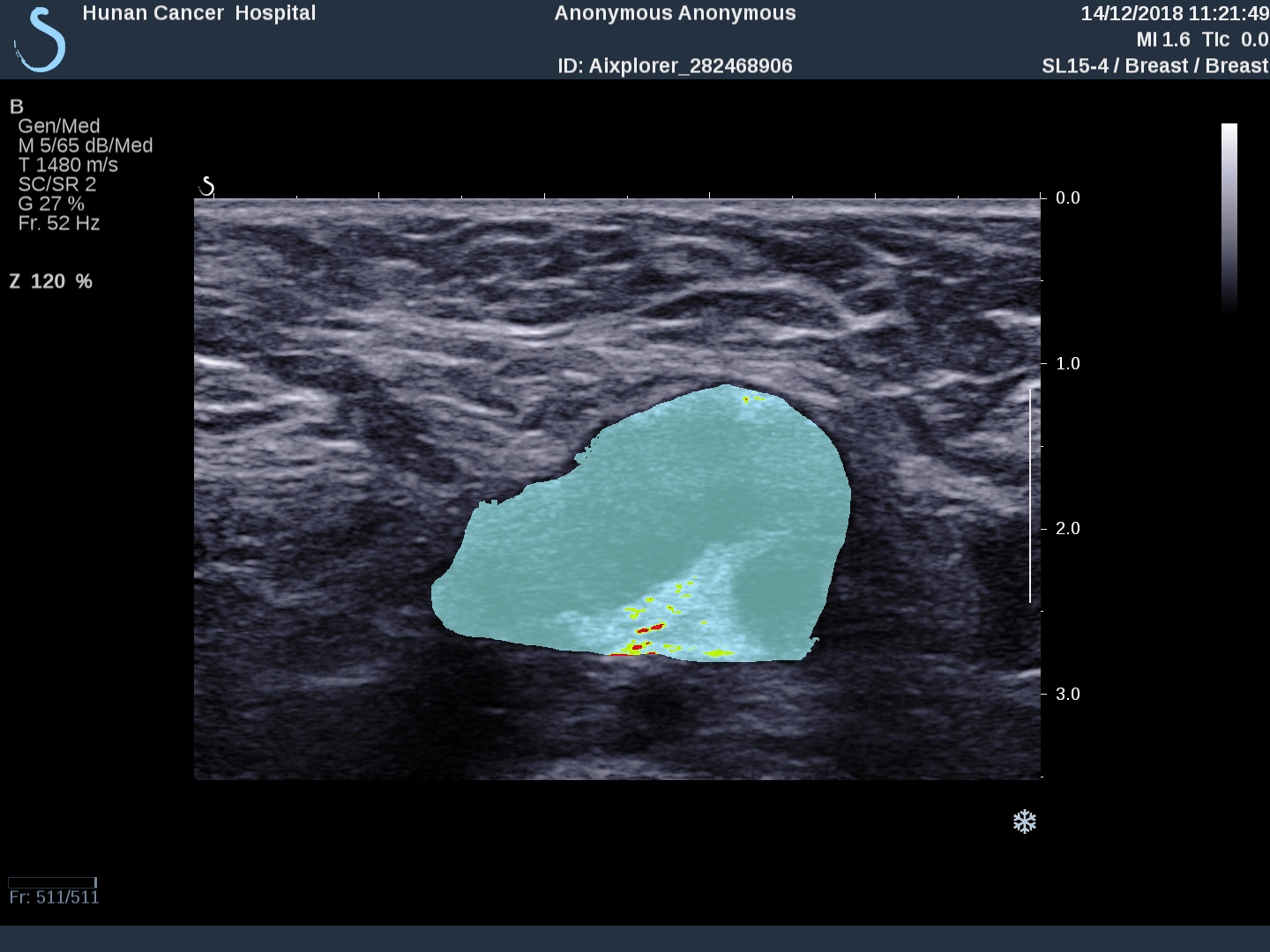

Supplement: Supplementary file 2 [file DataSheet_2.zip › ROI/1123604-1.jpg]

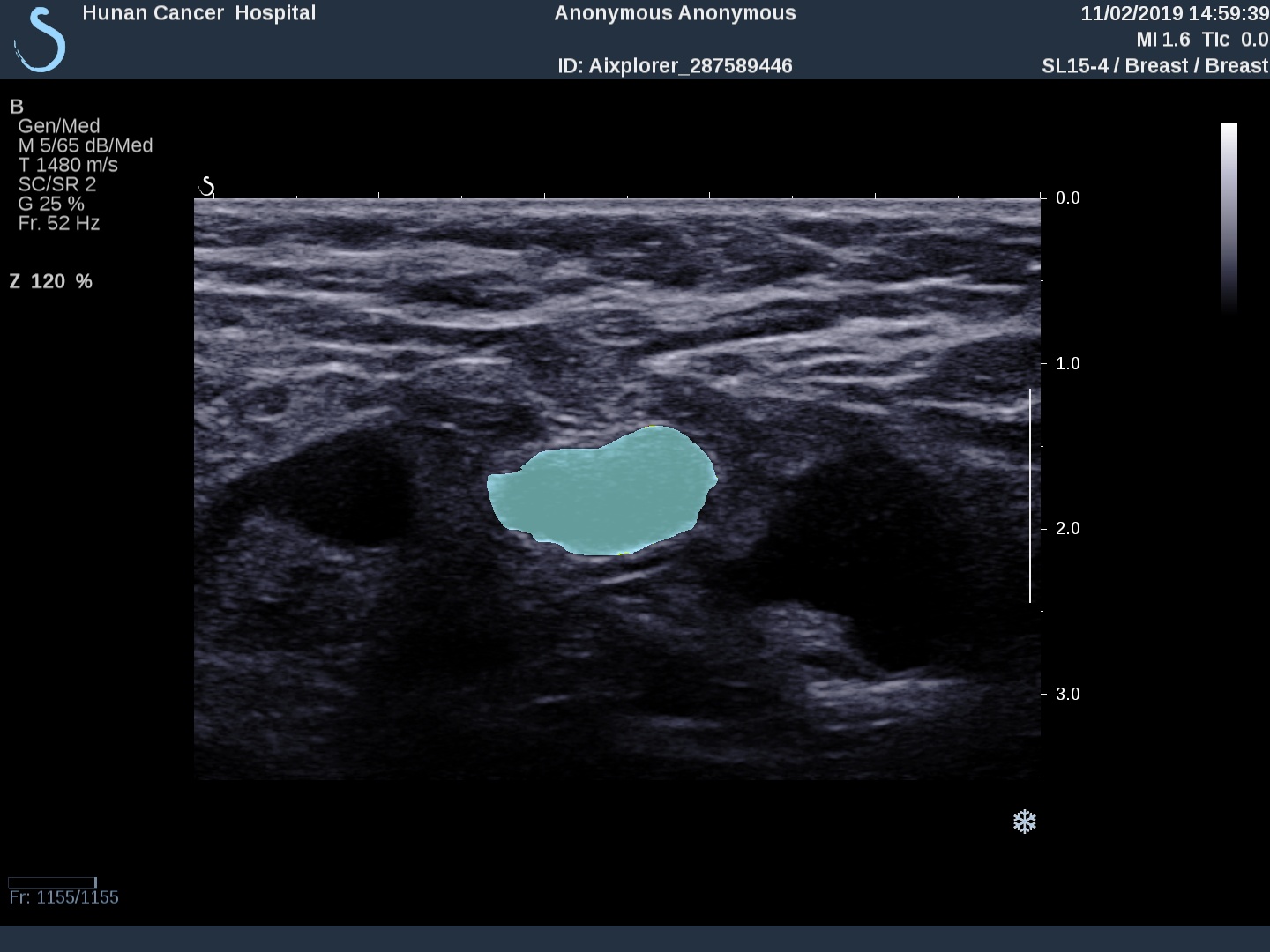

Supplement: Supplementary file 2 [file DataSheet_2.zip › ROI/1135654-1.jpg]

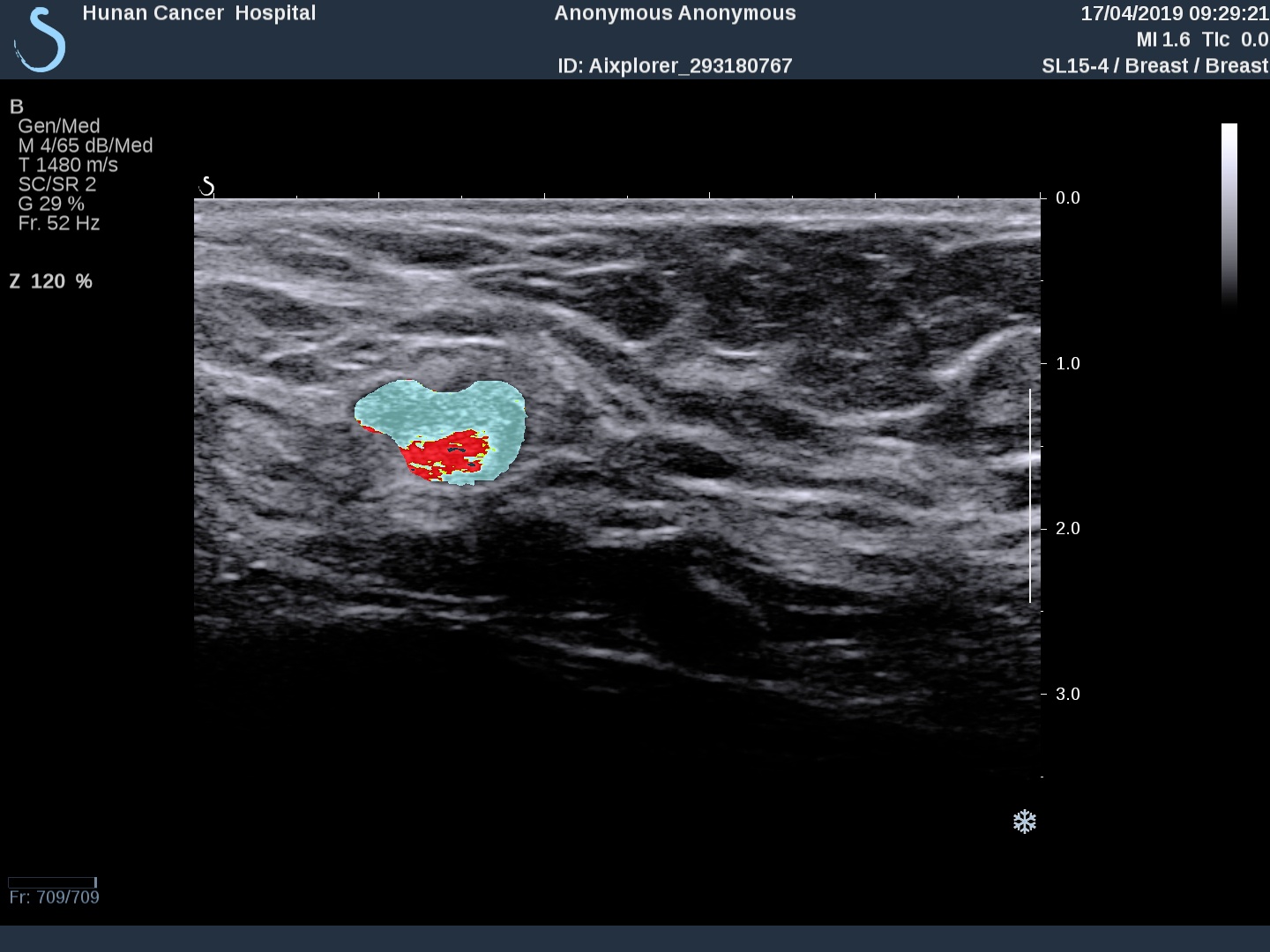

Supplement: Supplementary file 2 [file DataSheet_2.zip › ROI/1156548-1.jpg]

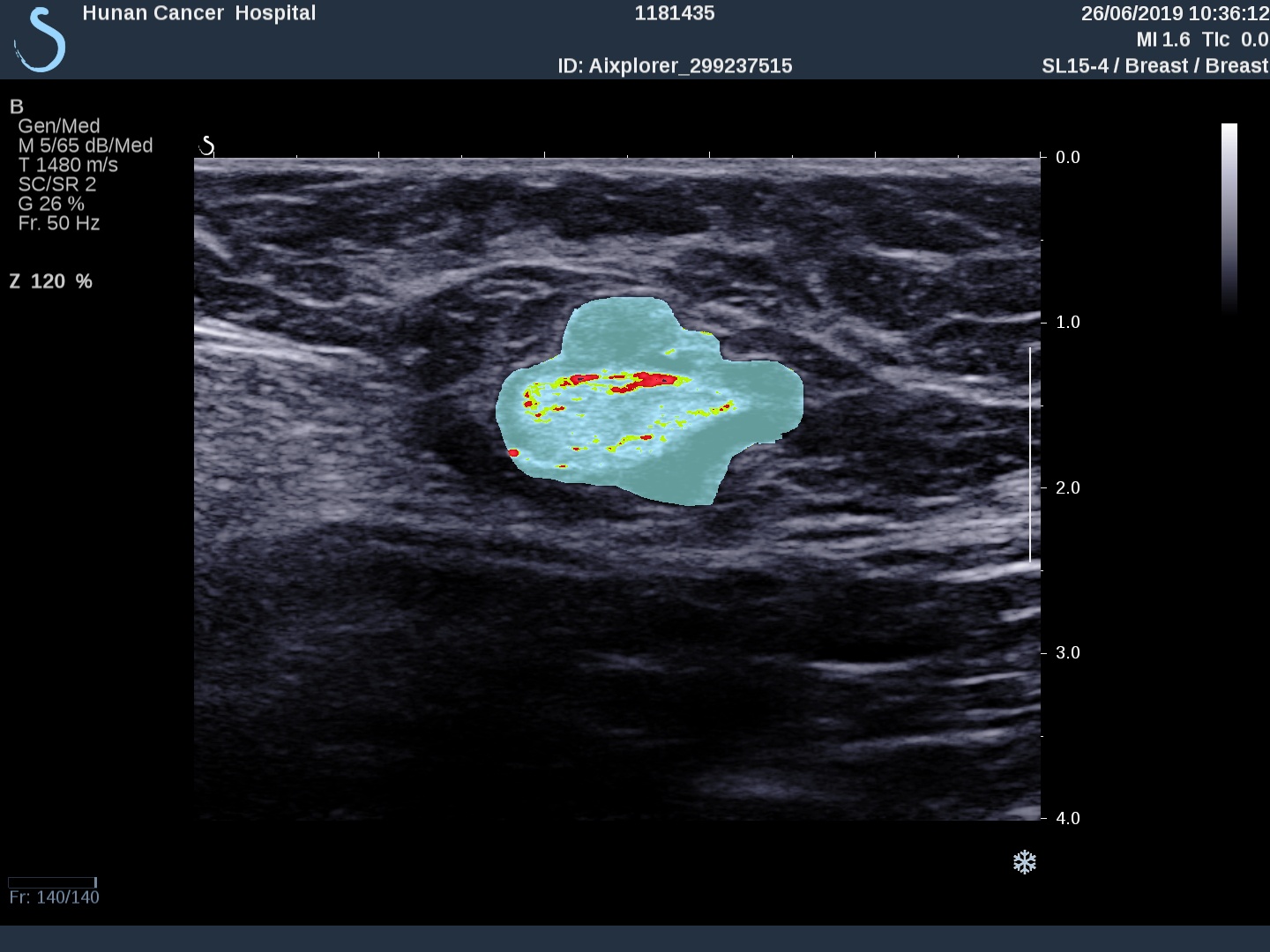

Supplement: Supplementary file 2 [file DataSheet_2.zip › ROI/1181435-1.jpg]

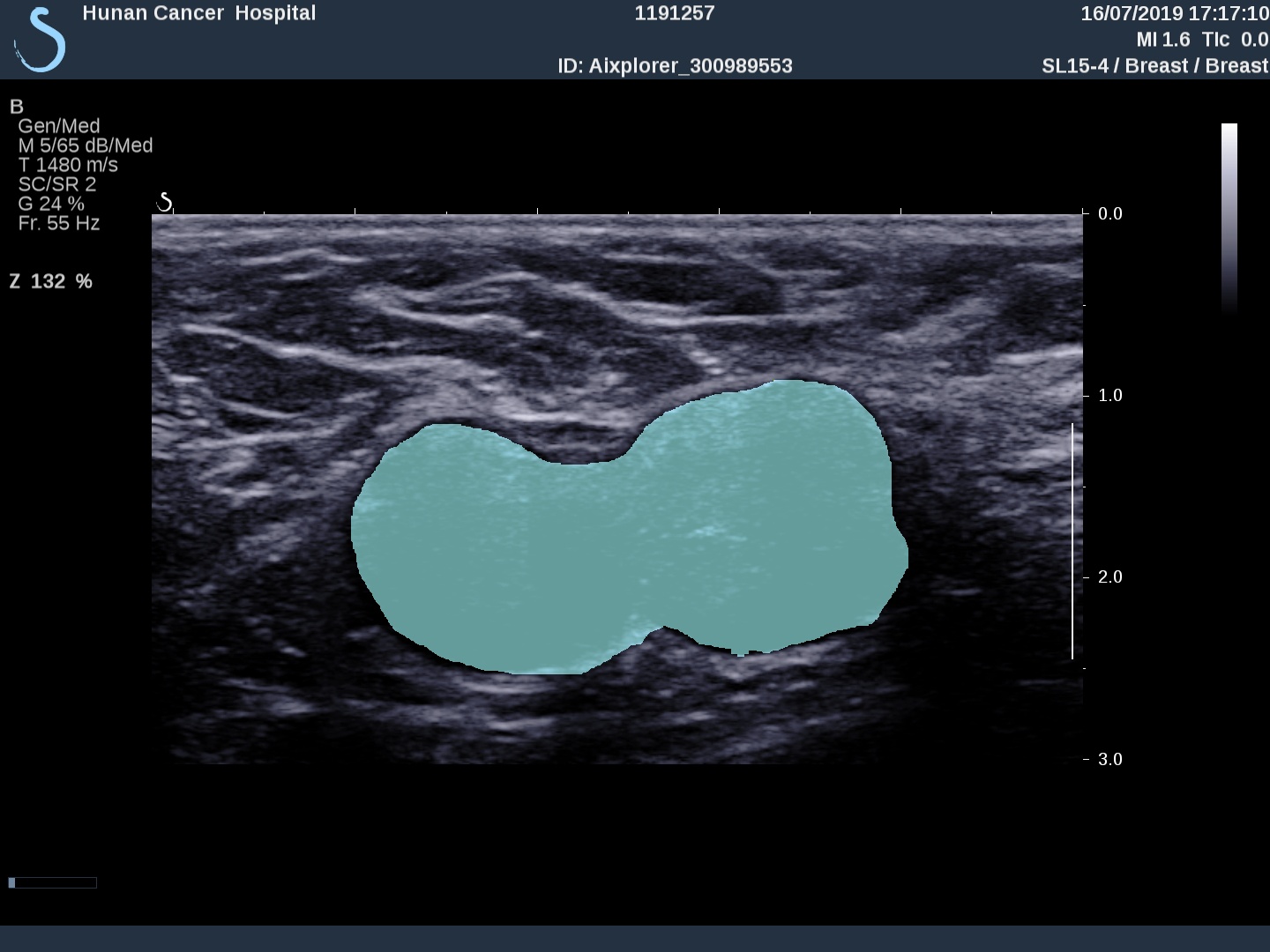

Supplement: Supplementary file 2 [file DataSheet_2.zip › ROI/1191257-1.jpg]

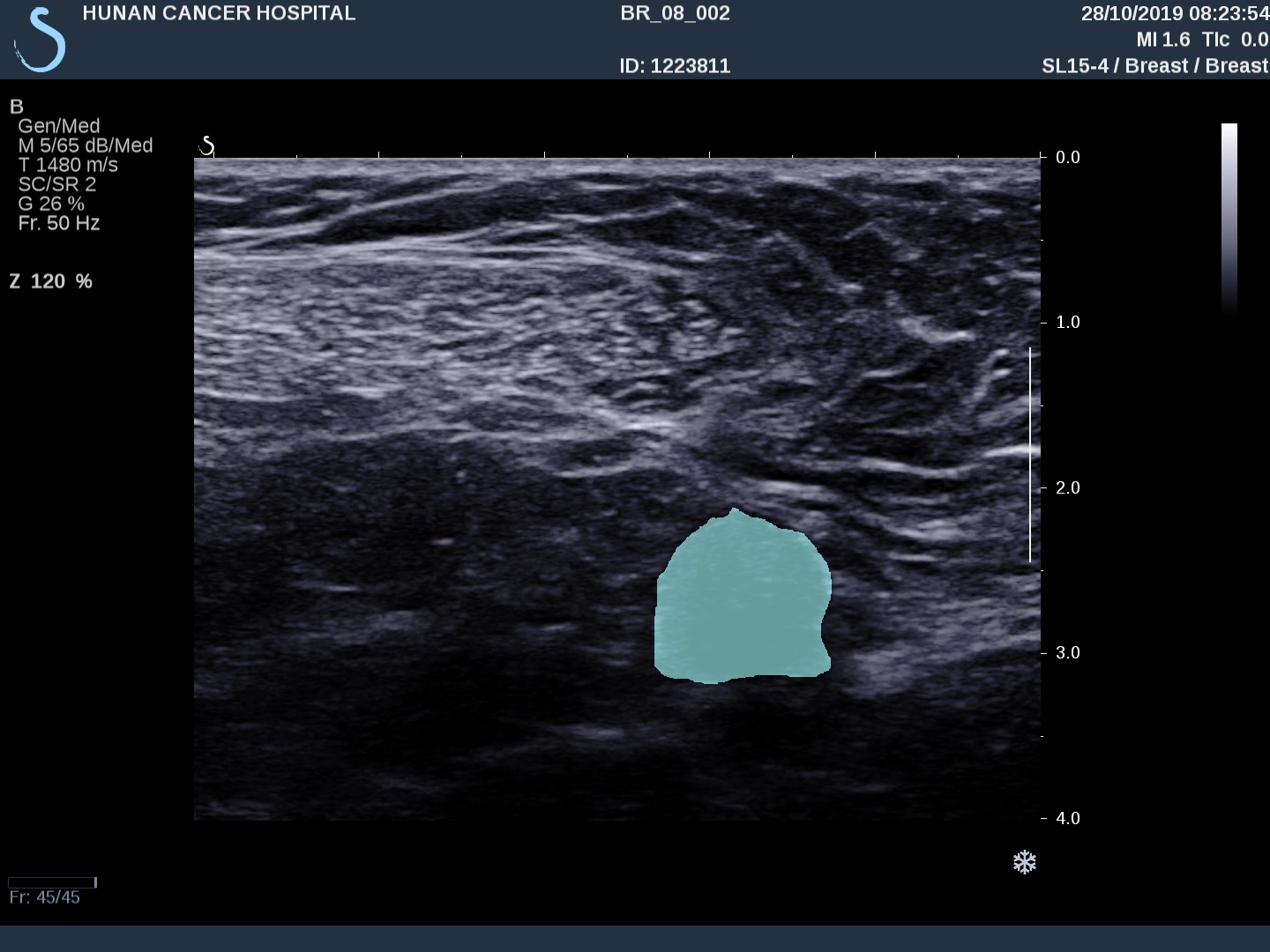

Supplement: Supplementary file 2 [file DataSheet_2.zip › ROI/1223811-1.jpg]

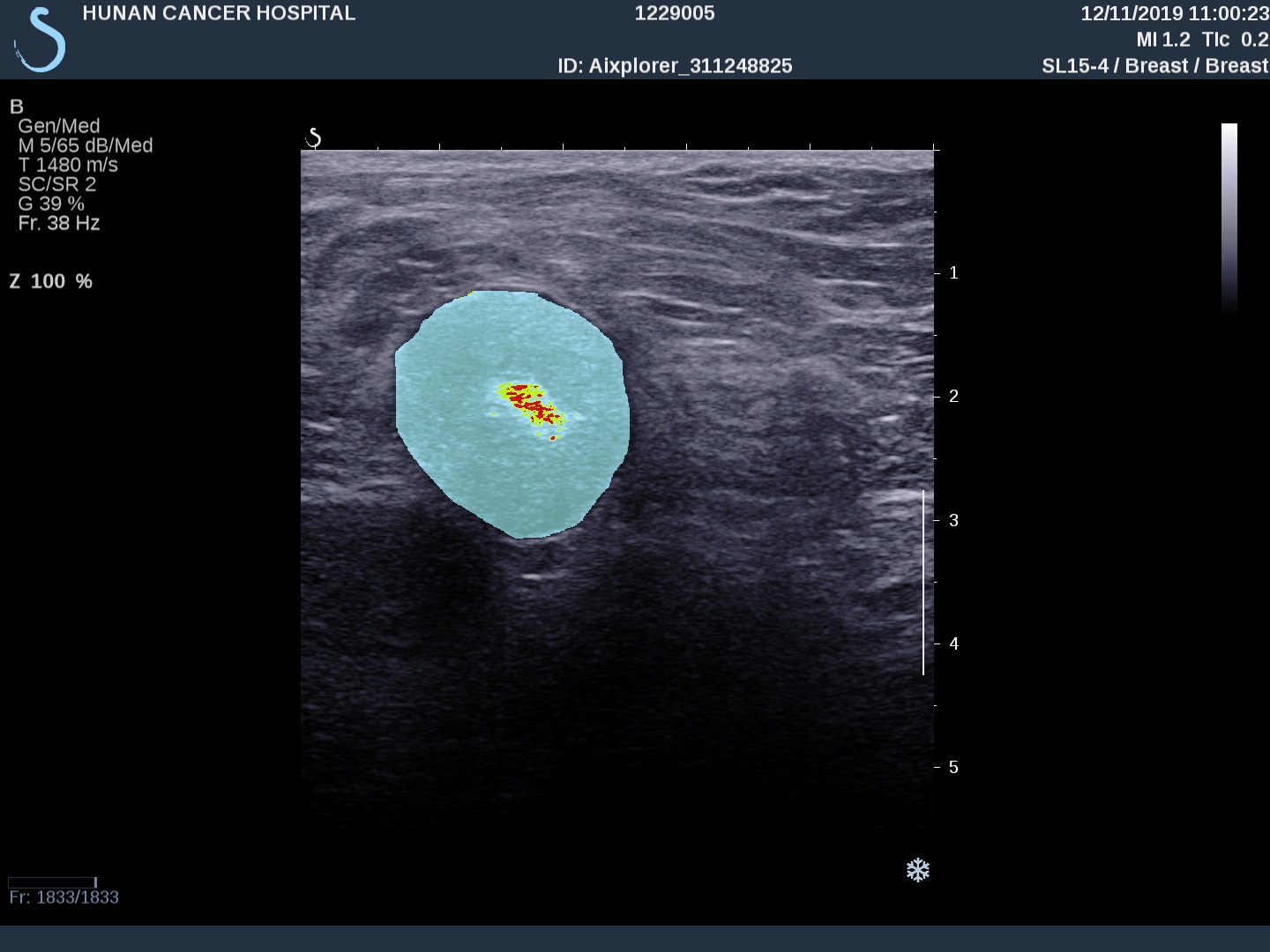

Supplement: Supplementary file 2 [file DataSheet_2.zip › ROI/1229005-1.jpg]

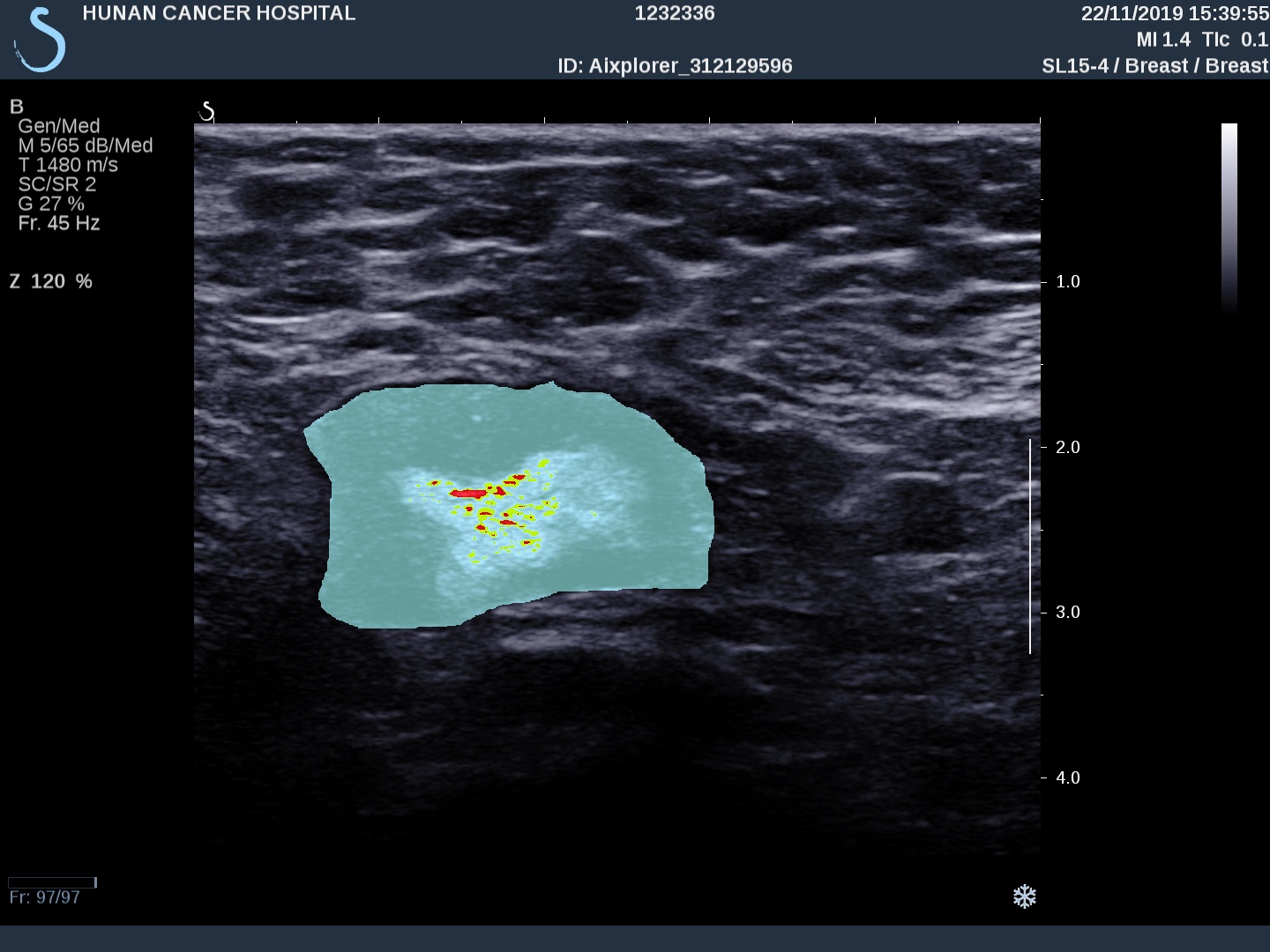

Supplement: Supplementary file 2 [file DataSheet_2.zip › ROI/1232336-1.jpg]

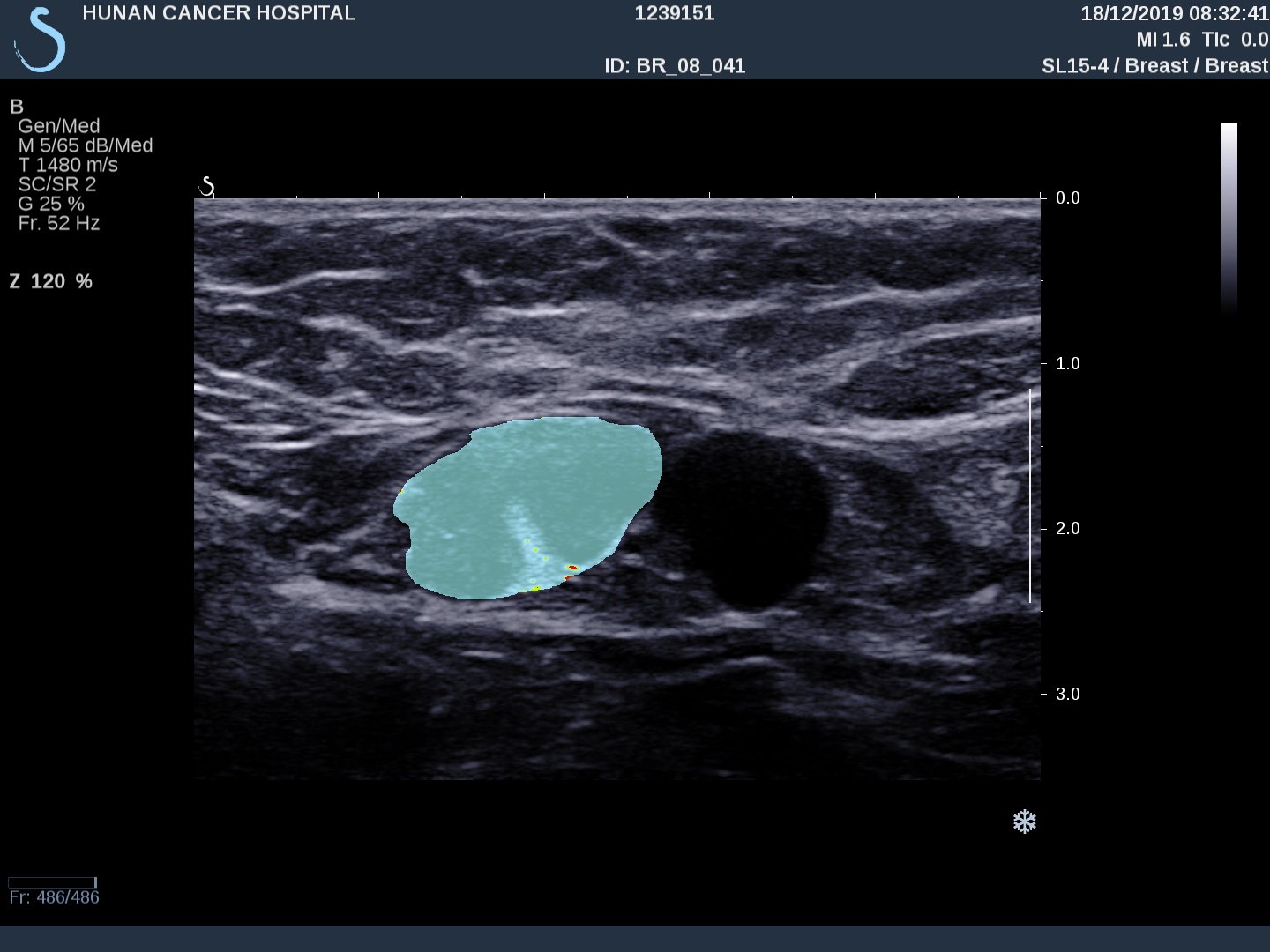

Supplement: Supplementary file 2 [file DataSheet_2.zip › ROI/1239151-1.jpg]

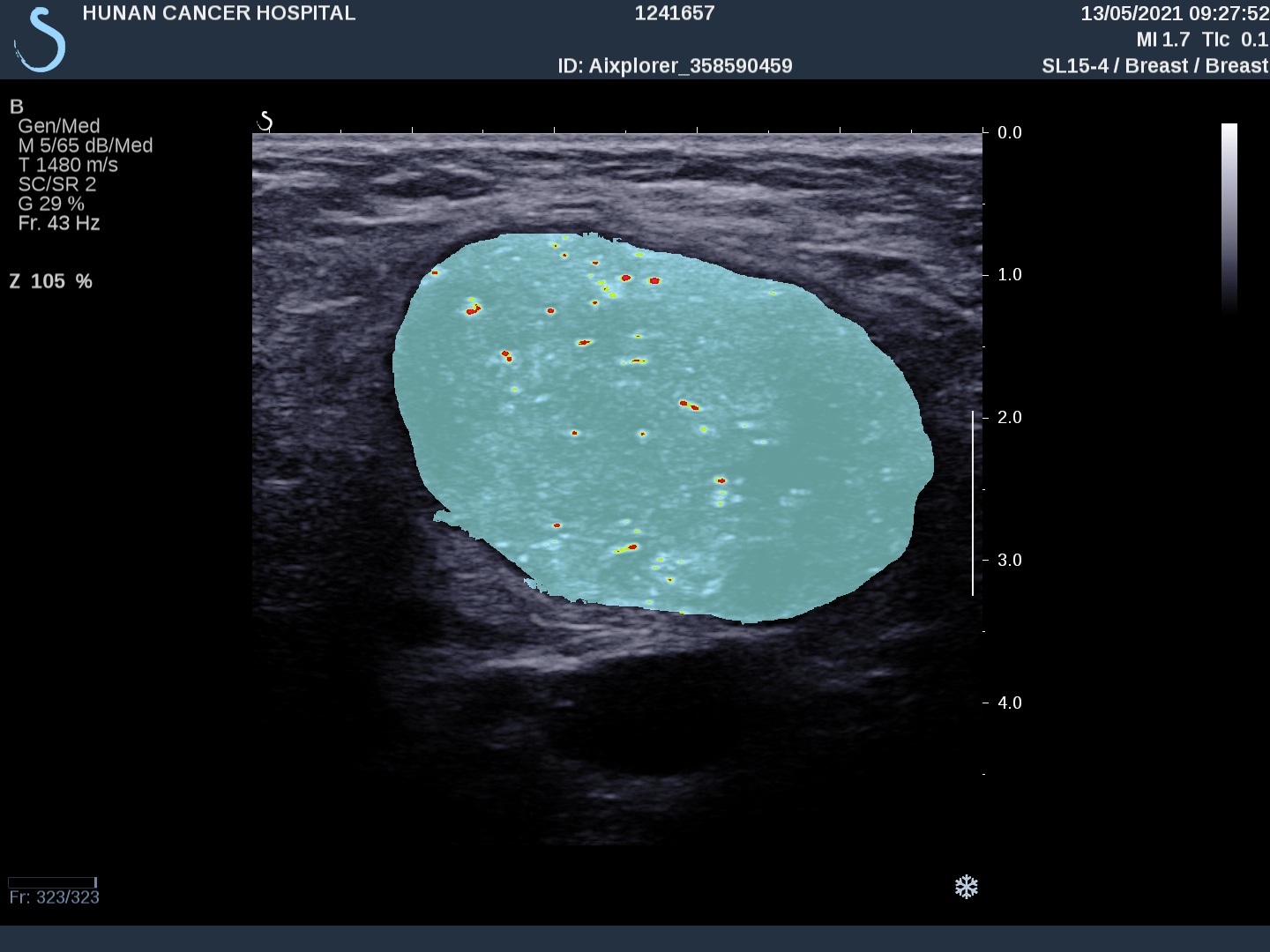

Supplement: Supplementary file 2 [file DataSheet_2.zip › ROI/1241657-1.jpg]

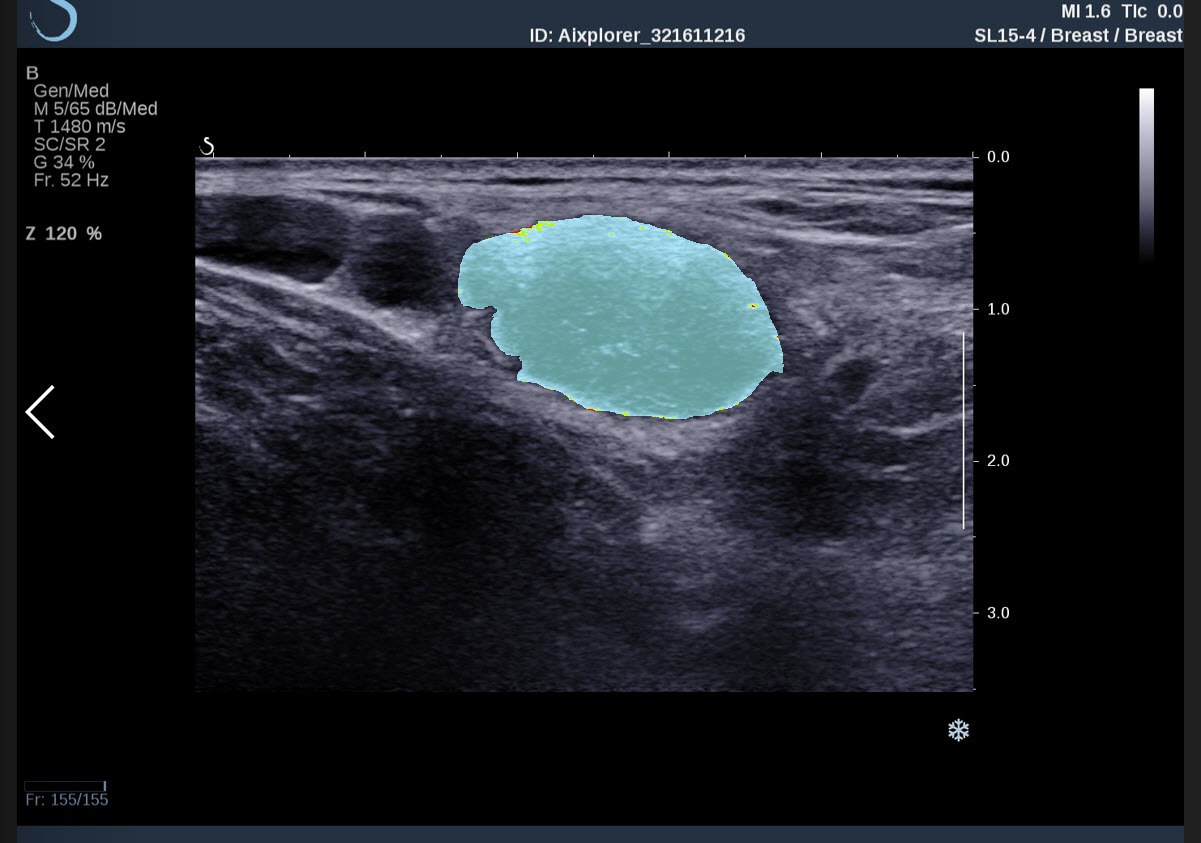

Supplement: Supplementary file 2 [file DataSheet_2.zip › ROI/1254530-1.jpg]

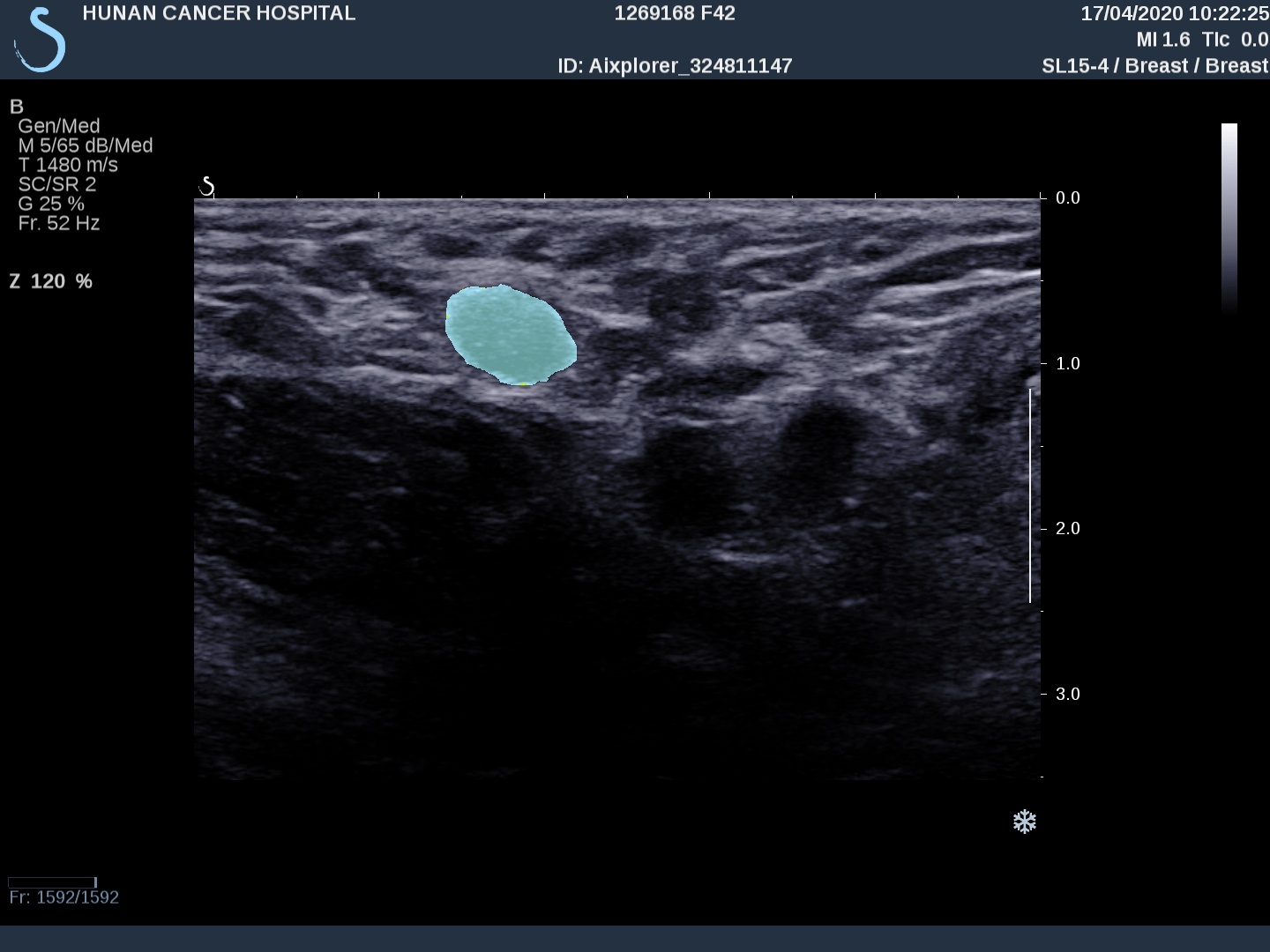

Supplement: Supplementary file 2 [file DataSheet_2.zip › ROI/1269169-1.jpg]

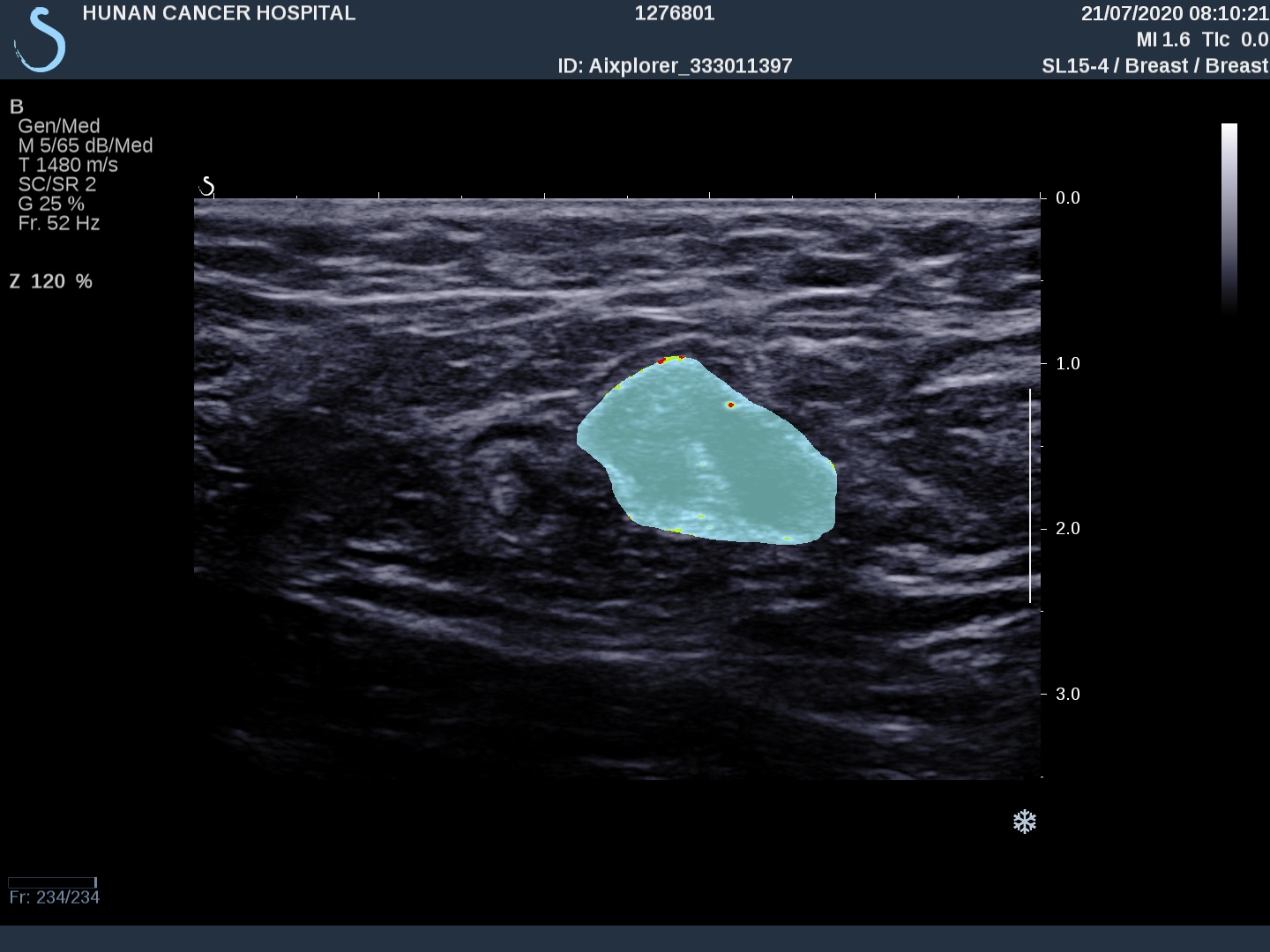

Supplement: Supplementary file 2 [file DataSheet_2.zip › ROI/1276801-1.jpg]

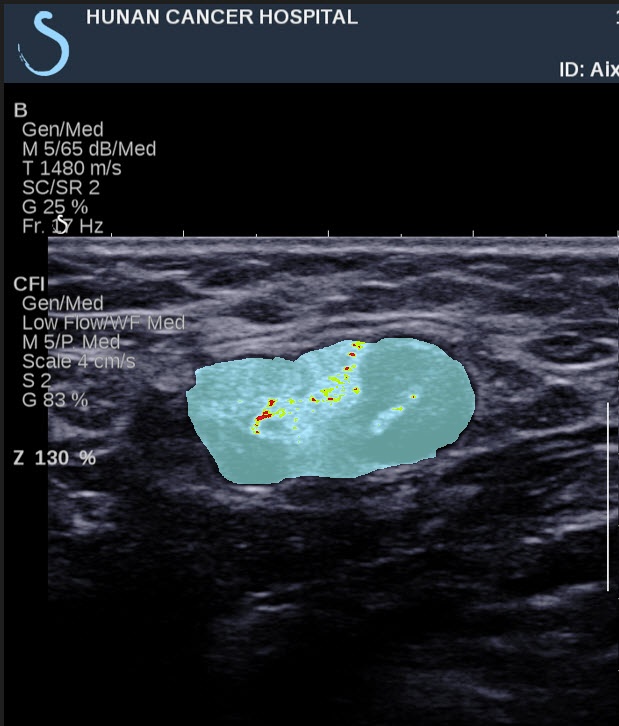

Supplement: Supplementary file 2 [file DataSheet_2.zip › ROI/1298809-1.jpg]

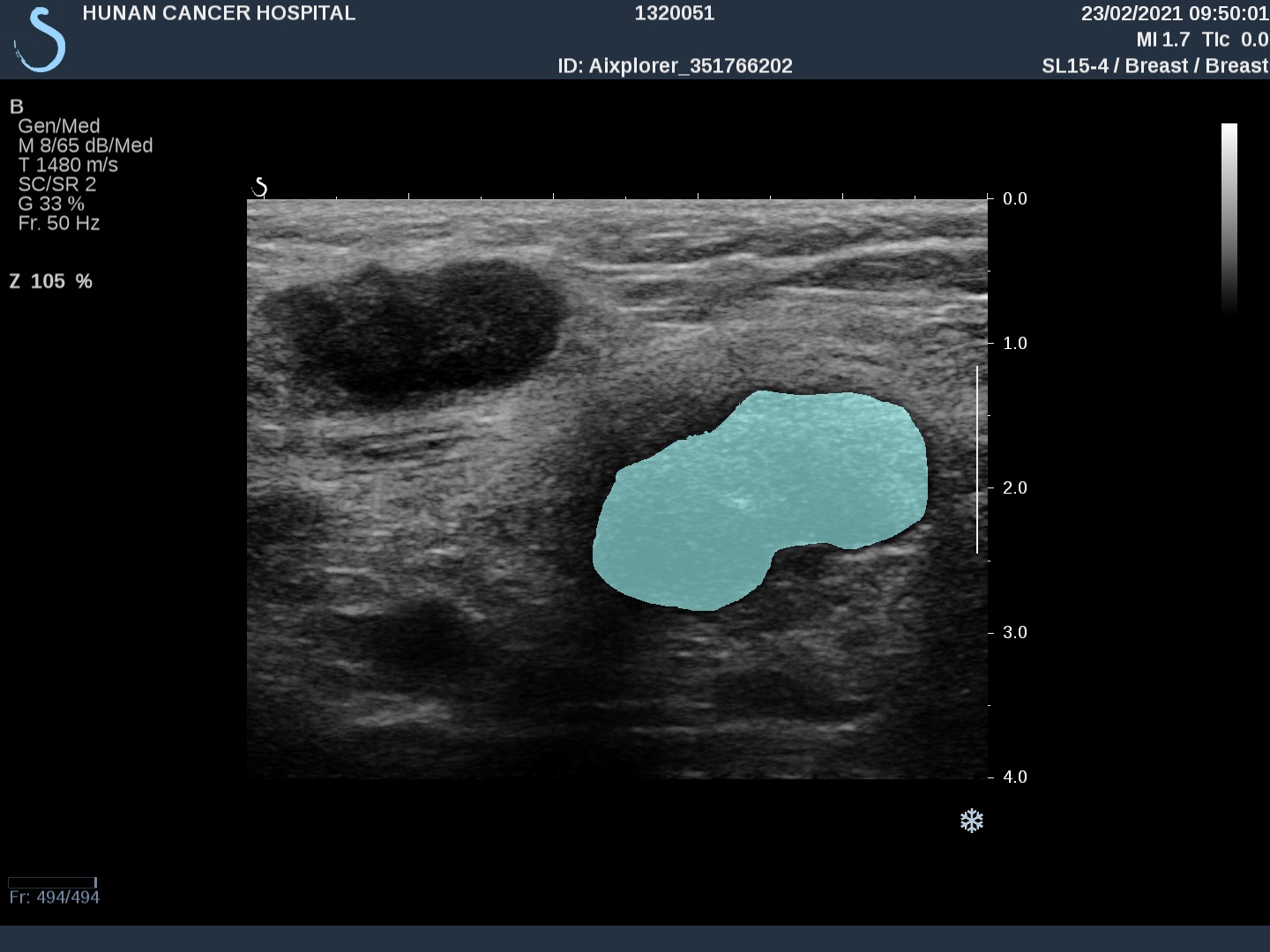

Supplement: Supplementary file 2 [file DataSheet_2.zip › ROI/1320051-1.jpg]

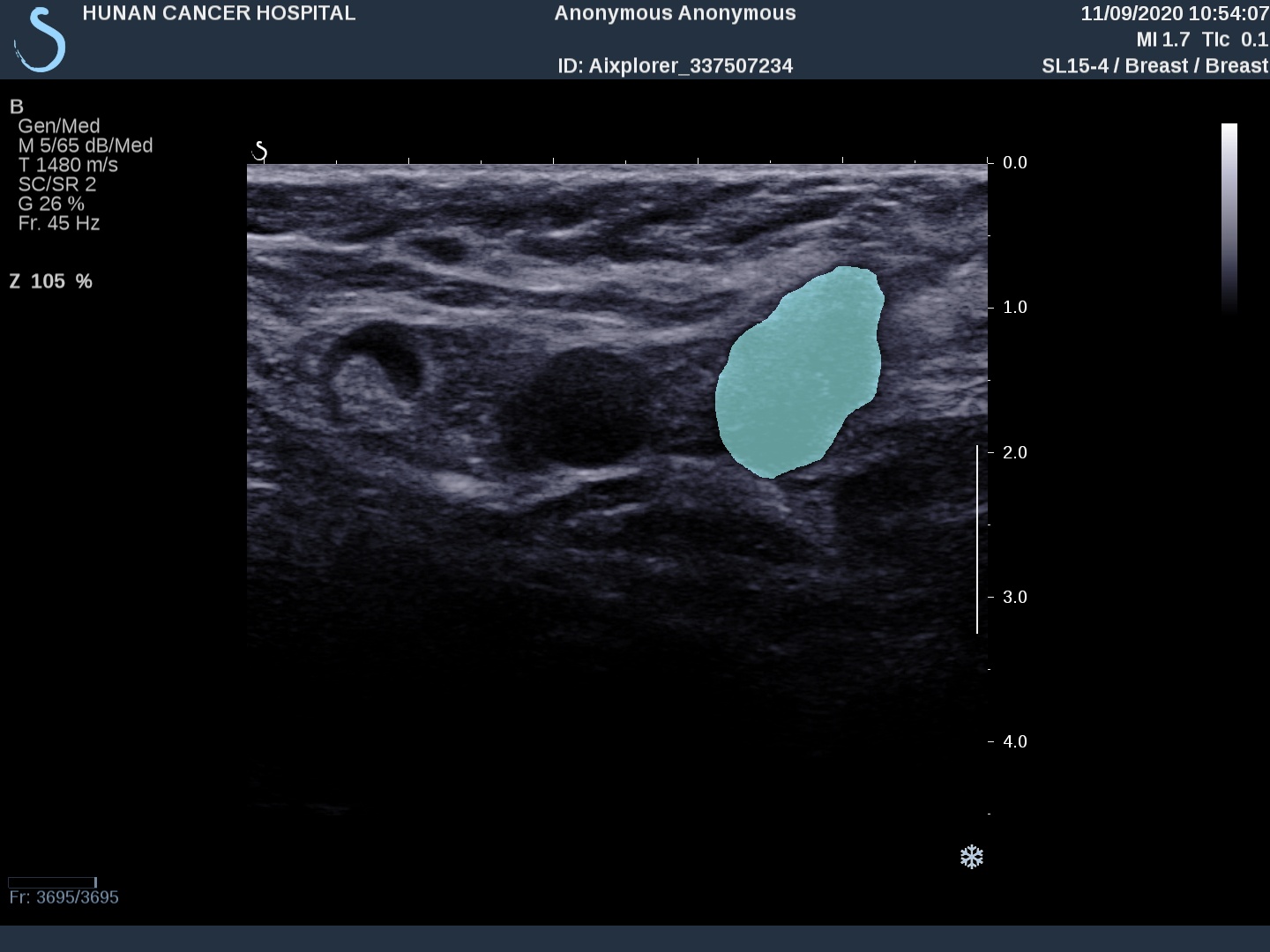

Supplement: Supplementary file 2 [file DataSheet_2.zip › ROI/1331039-1.jpg]

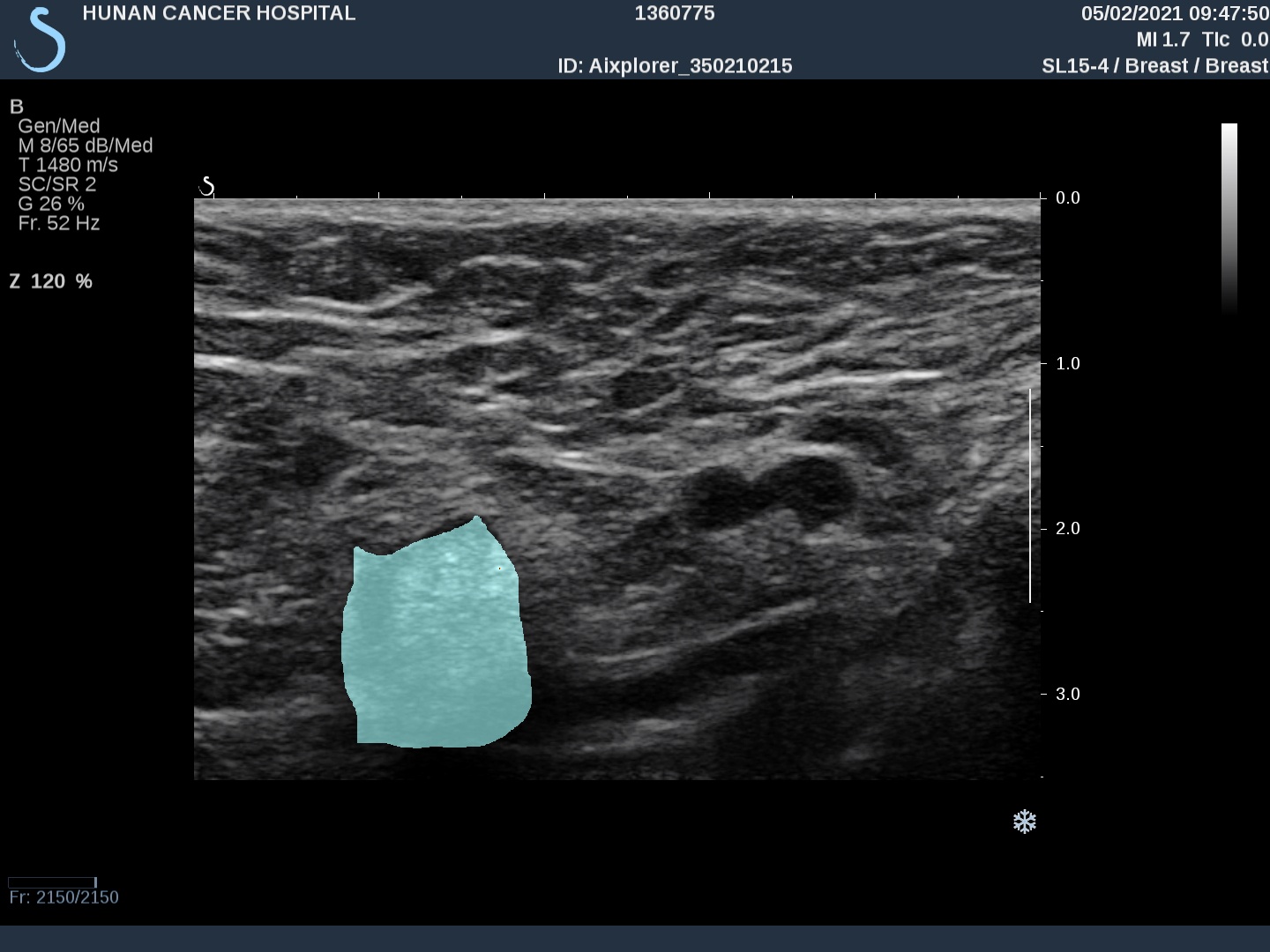

Supplement: Supplementary file 2 [file DataSheet_2.zip › ROI/1360775-1.jpg]

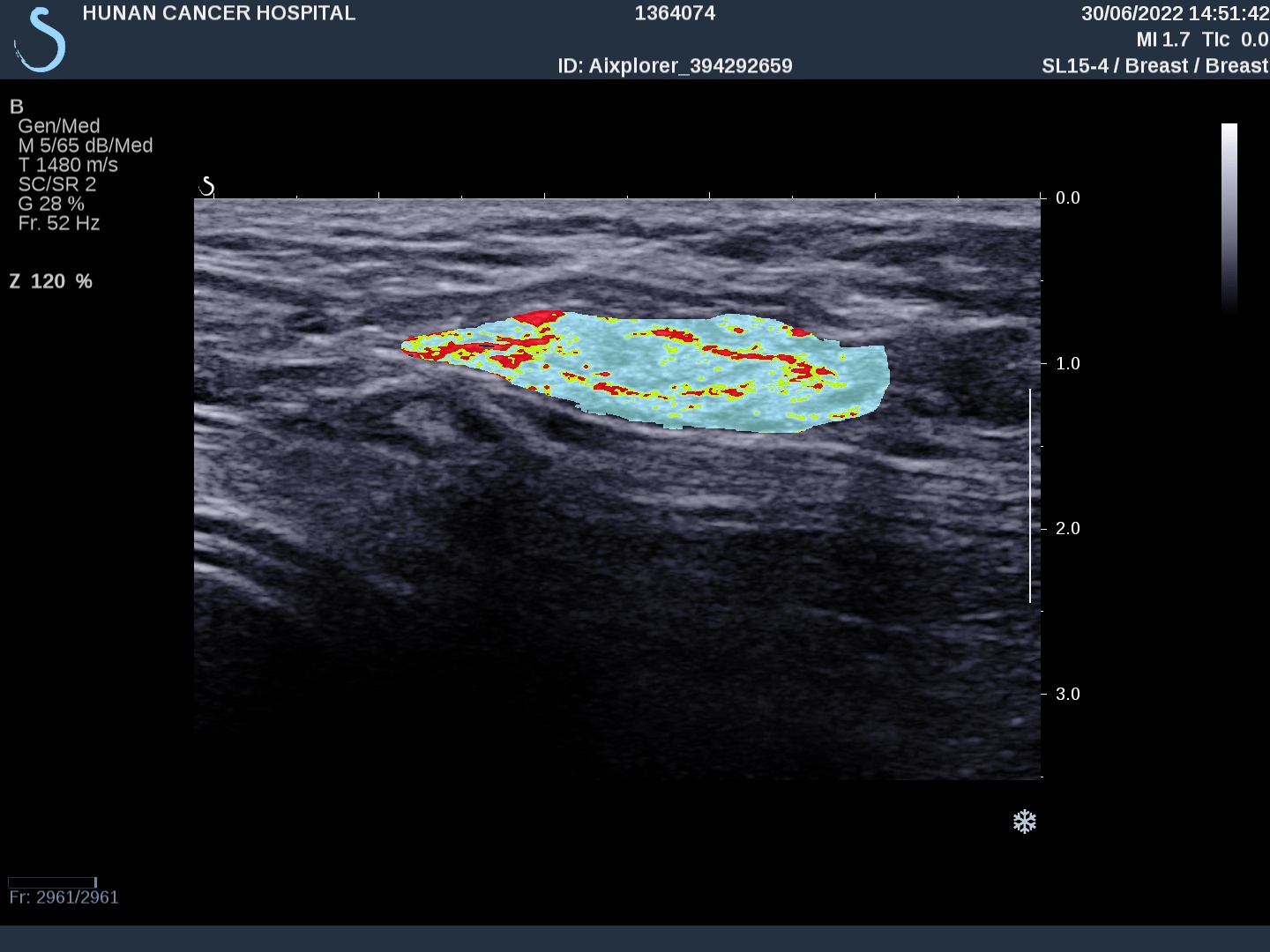

Supplement: Supplementary file 2 [file DataSheet_2.zip › ROI/1364074-2.jpg]

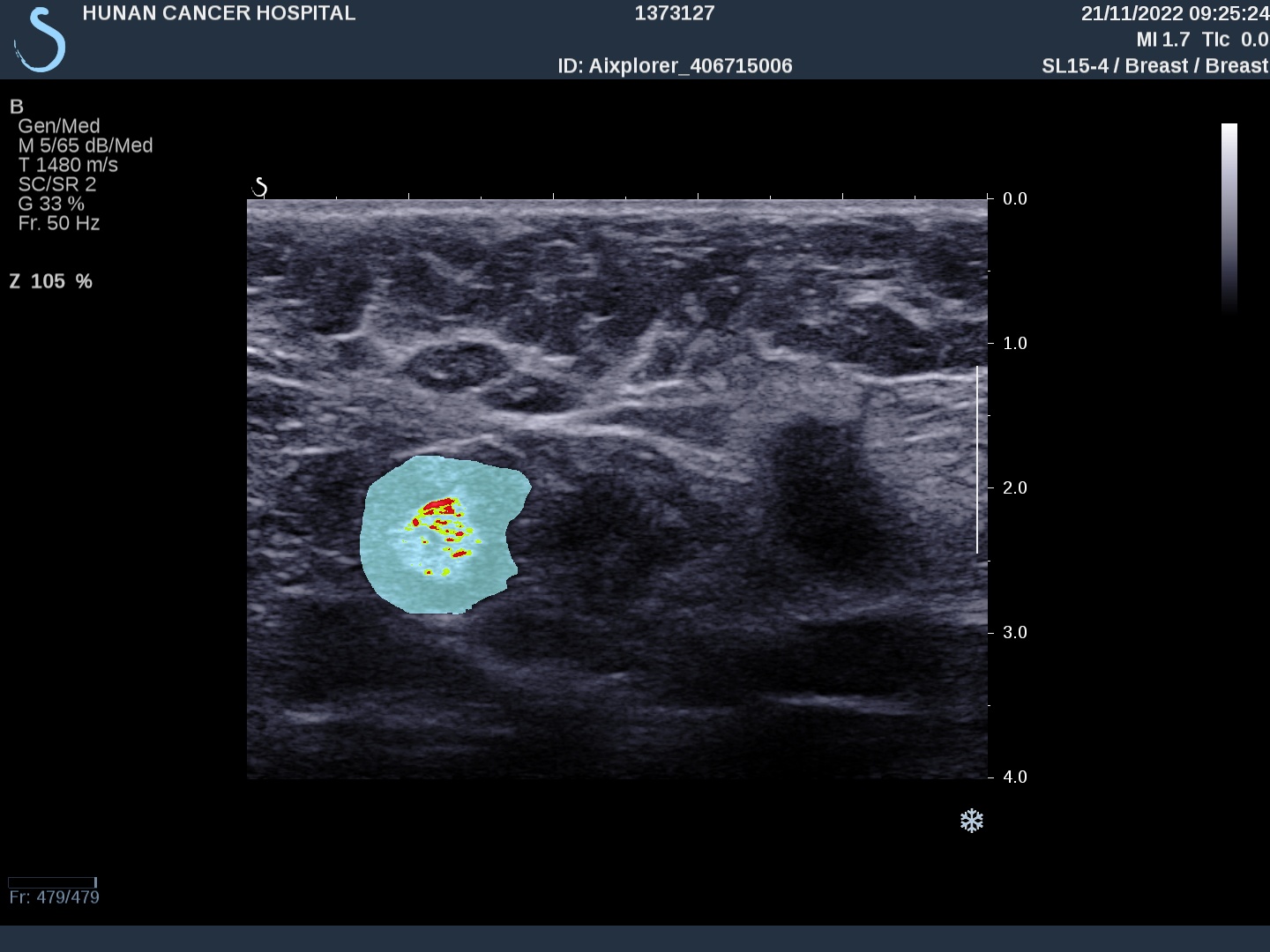

Supplement: Supplementary file 2 [file DataSheet_2.zip › ROI/1373127-2.jpg]

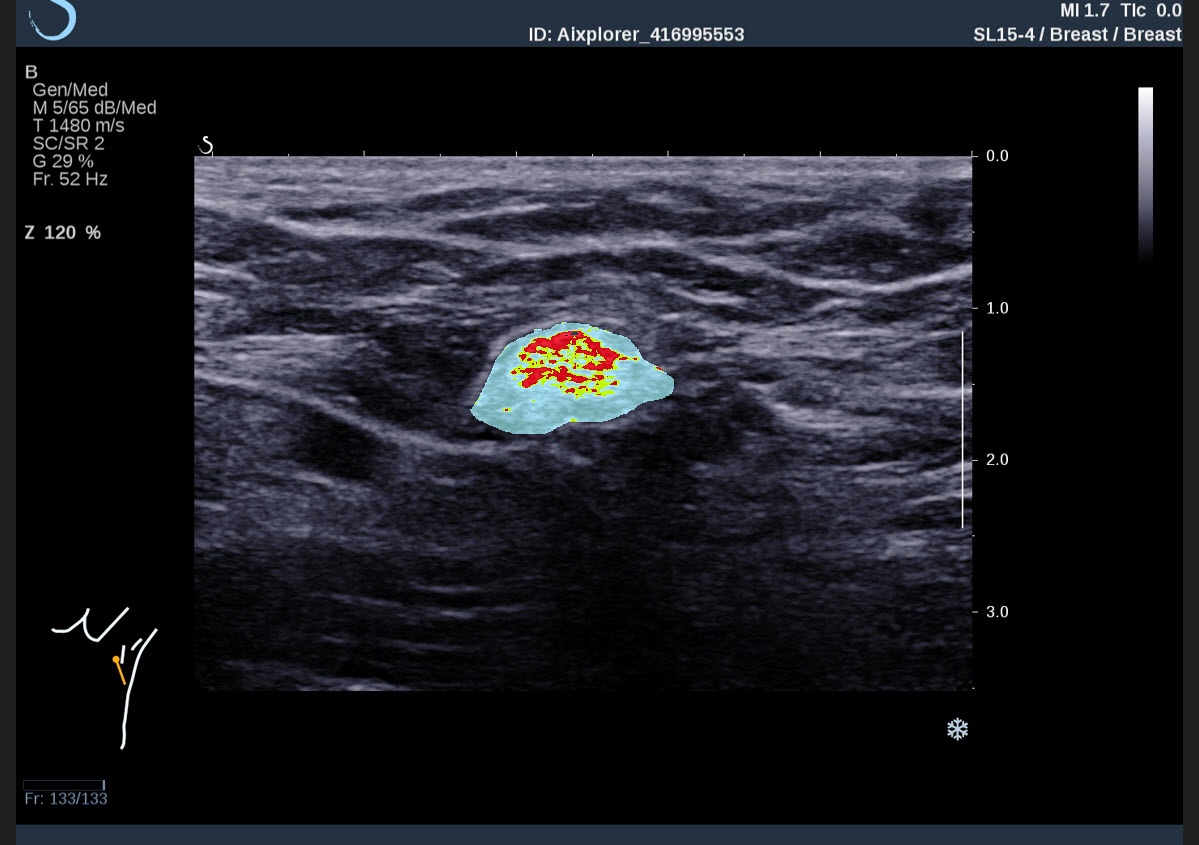

Supplement: Supplementary file 2 [file DataSheet_2.zip › ROI/1378251-1.jpg]

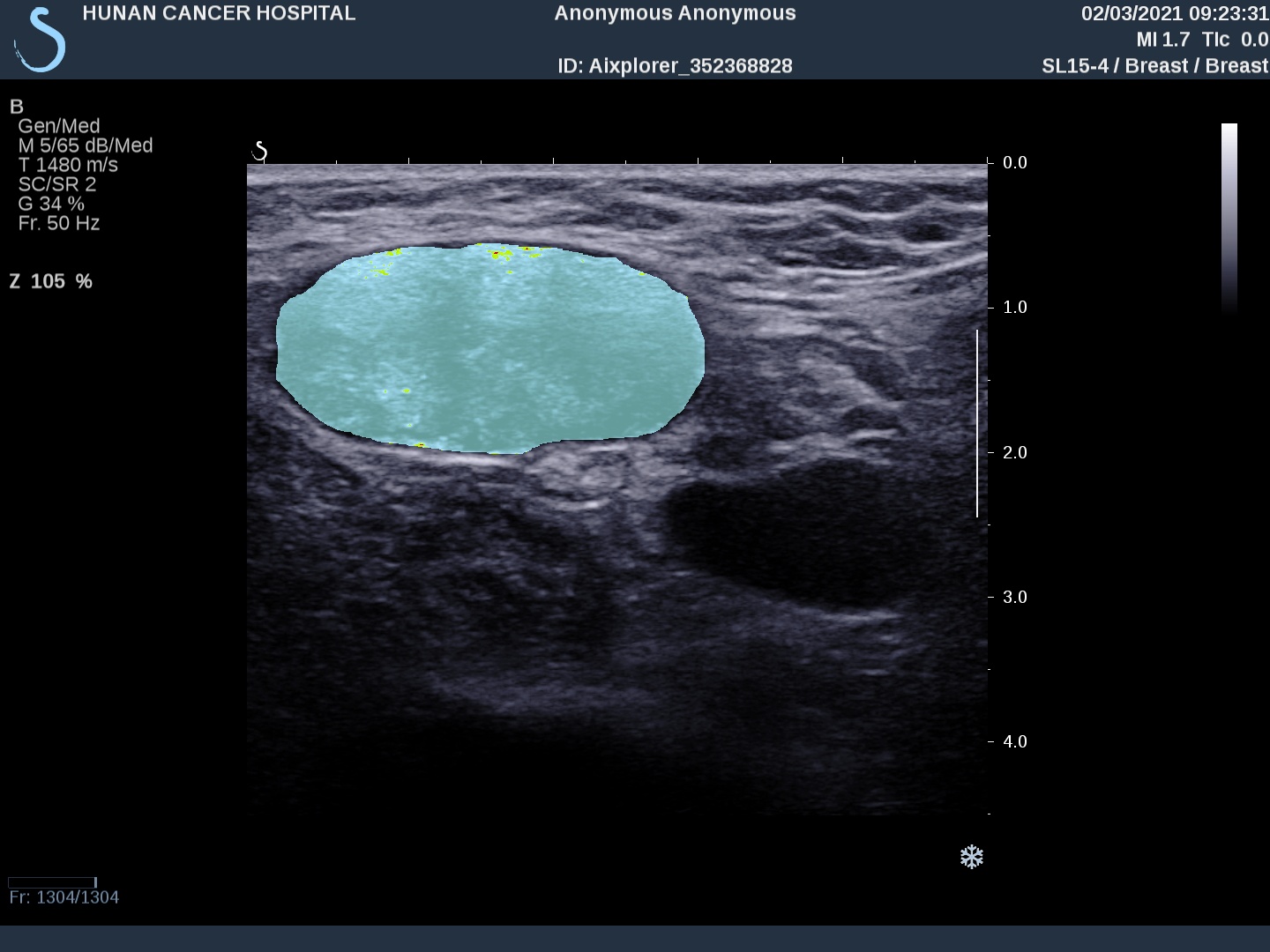

Supplement: Supplementary file 2 [file DataSheet_2.zip › ROI/1402578-1.jpg]

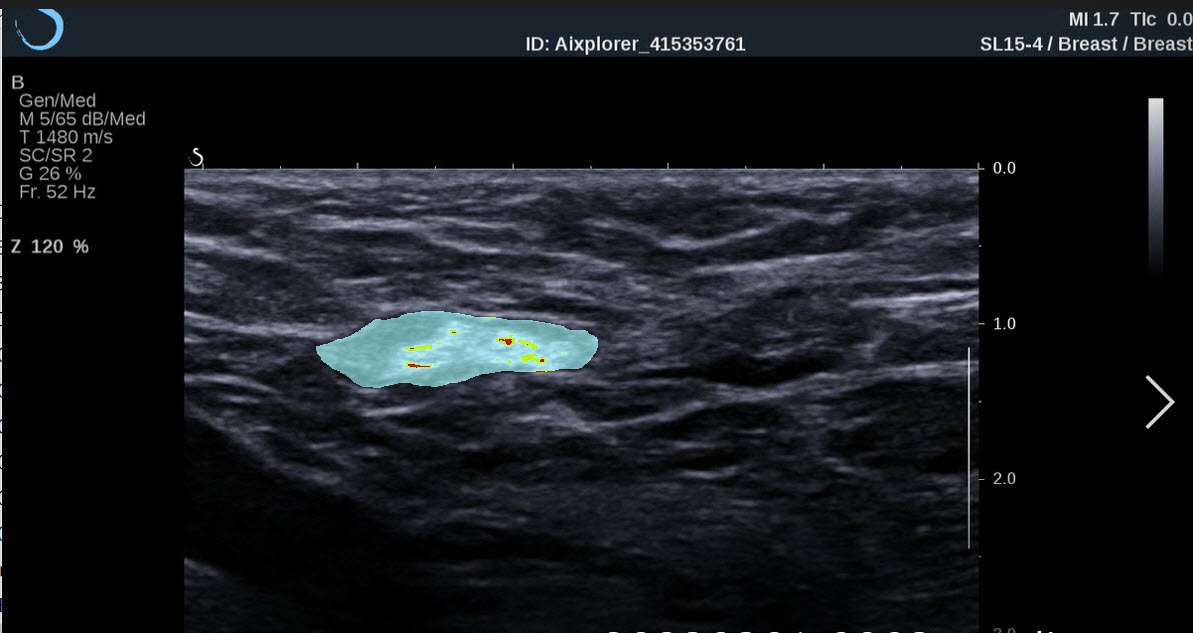

Supplement: Supplementary file 2 [file DataSheet_2.zip › ROI/1404836-2.jpg]

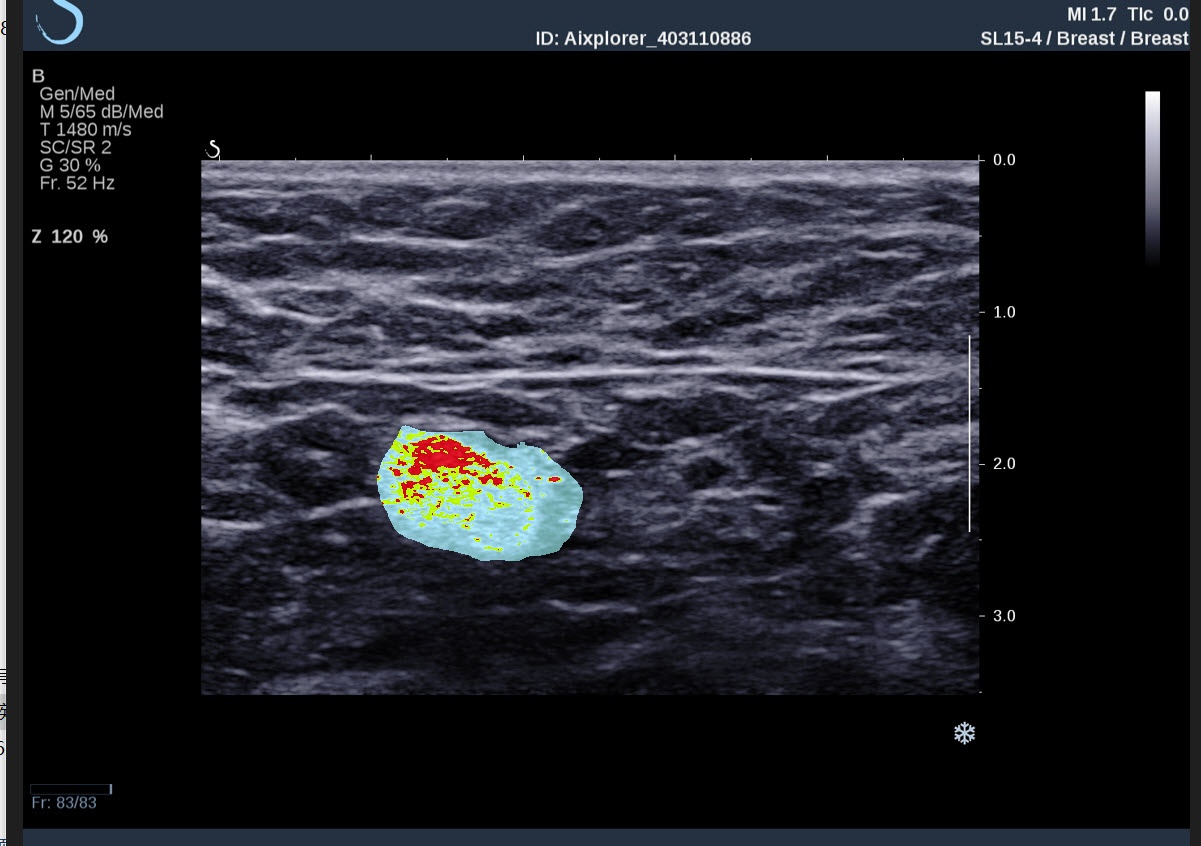

Supplement: Supplementary file 2 [file DataSheet_2.zip › ROI/1404939-1.jpg]

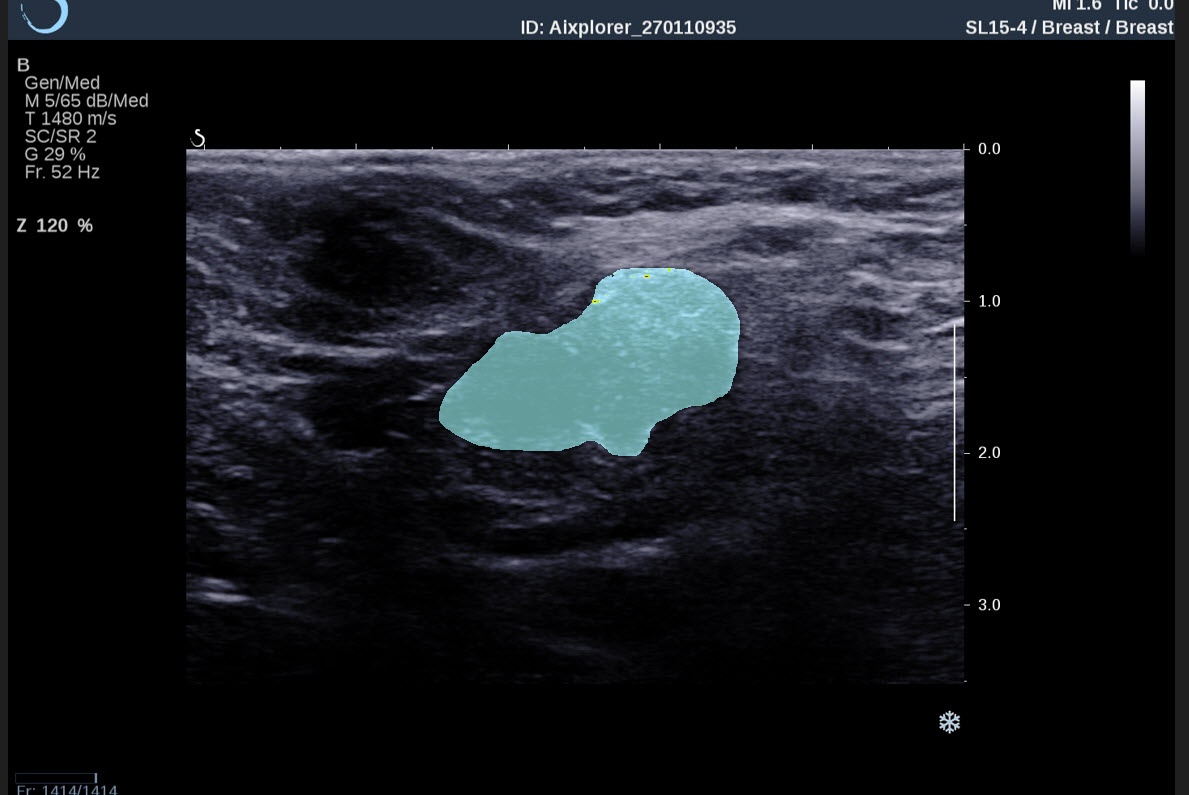

Supplement: Supplementary file 2 [file DataSheet_2.zip › ROI/1406351-4.jpg]

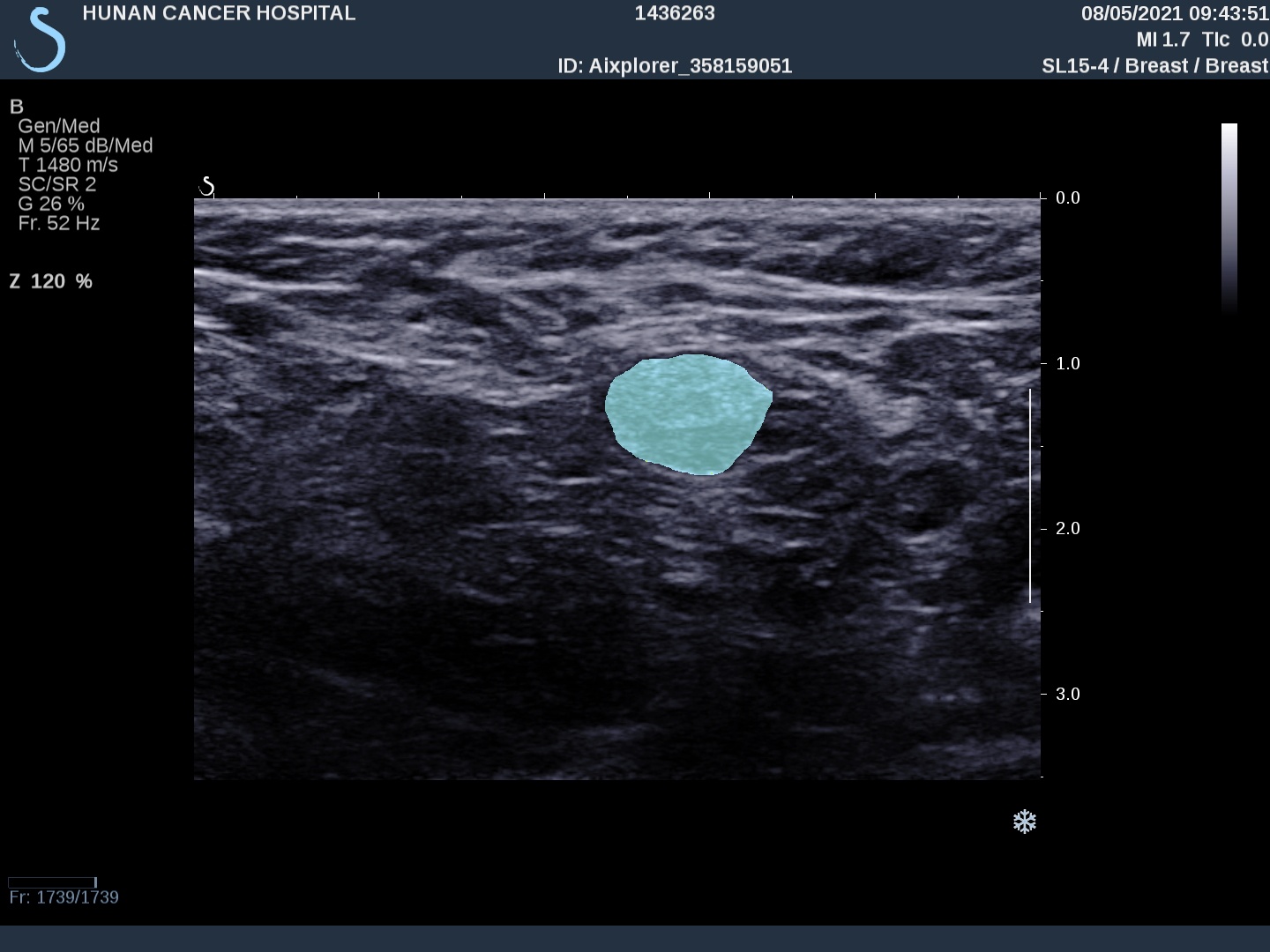

Supplement: Supplementary file 2 [file DataSheet_2.zip › ROI/1436263-2.jpg]

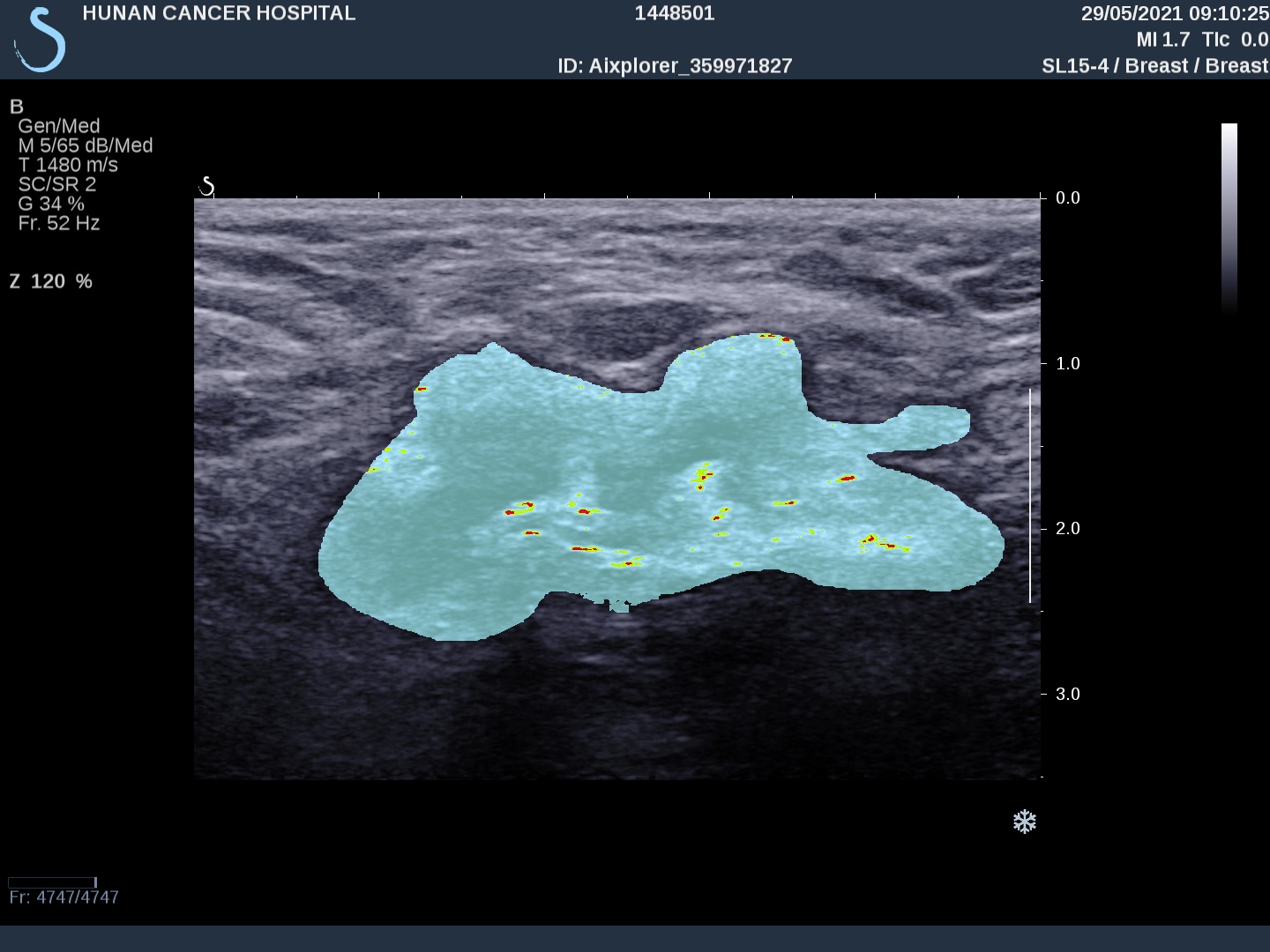

Supplement: Supplementary file 2 [file DataSheet_2.zip › ROI/1448501-1.jpg]

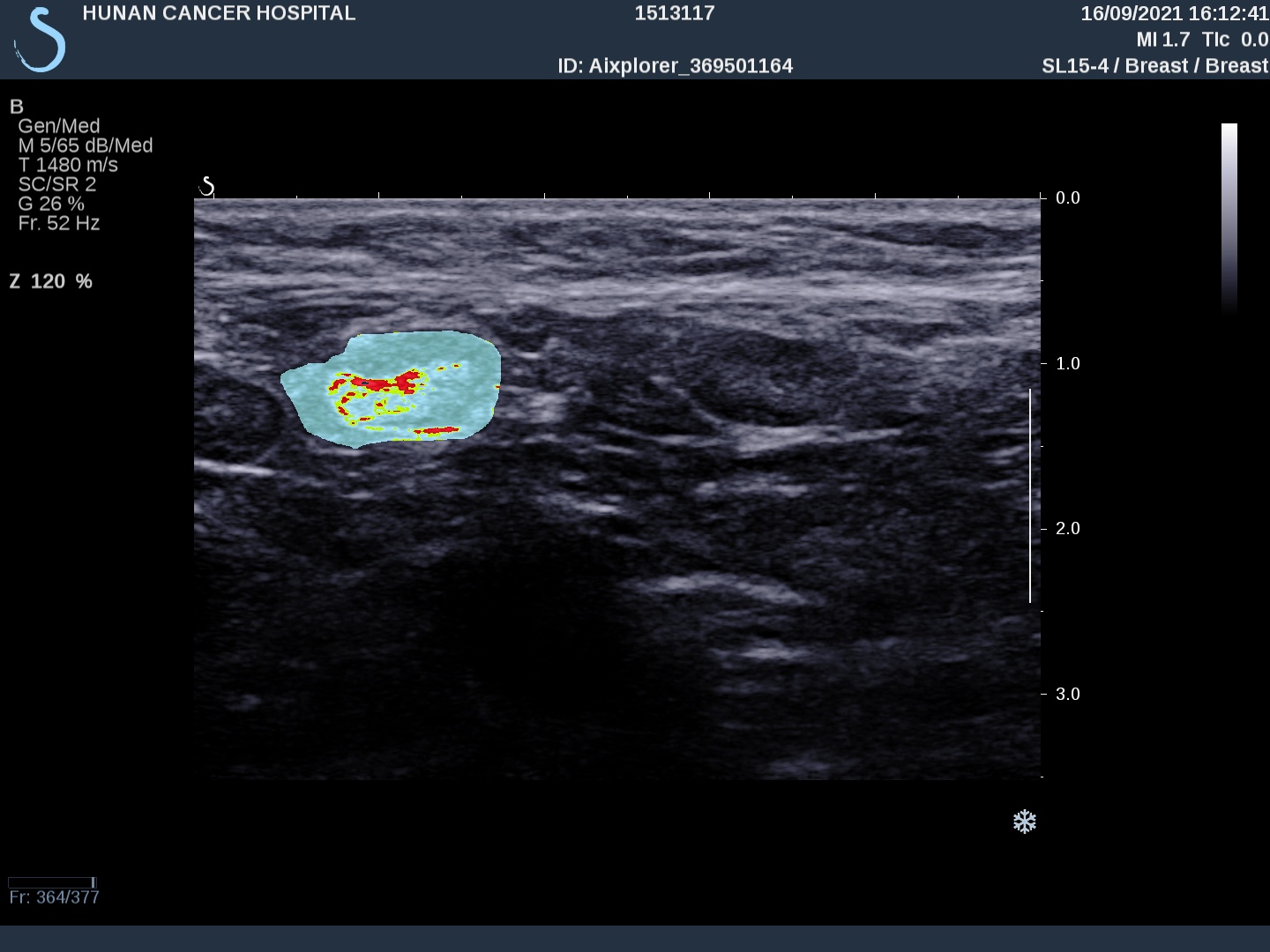

Supplement: Supplementary file 2 [file DataSheet_2.zip › ROI/1513117-1.jpg]

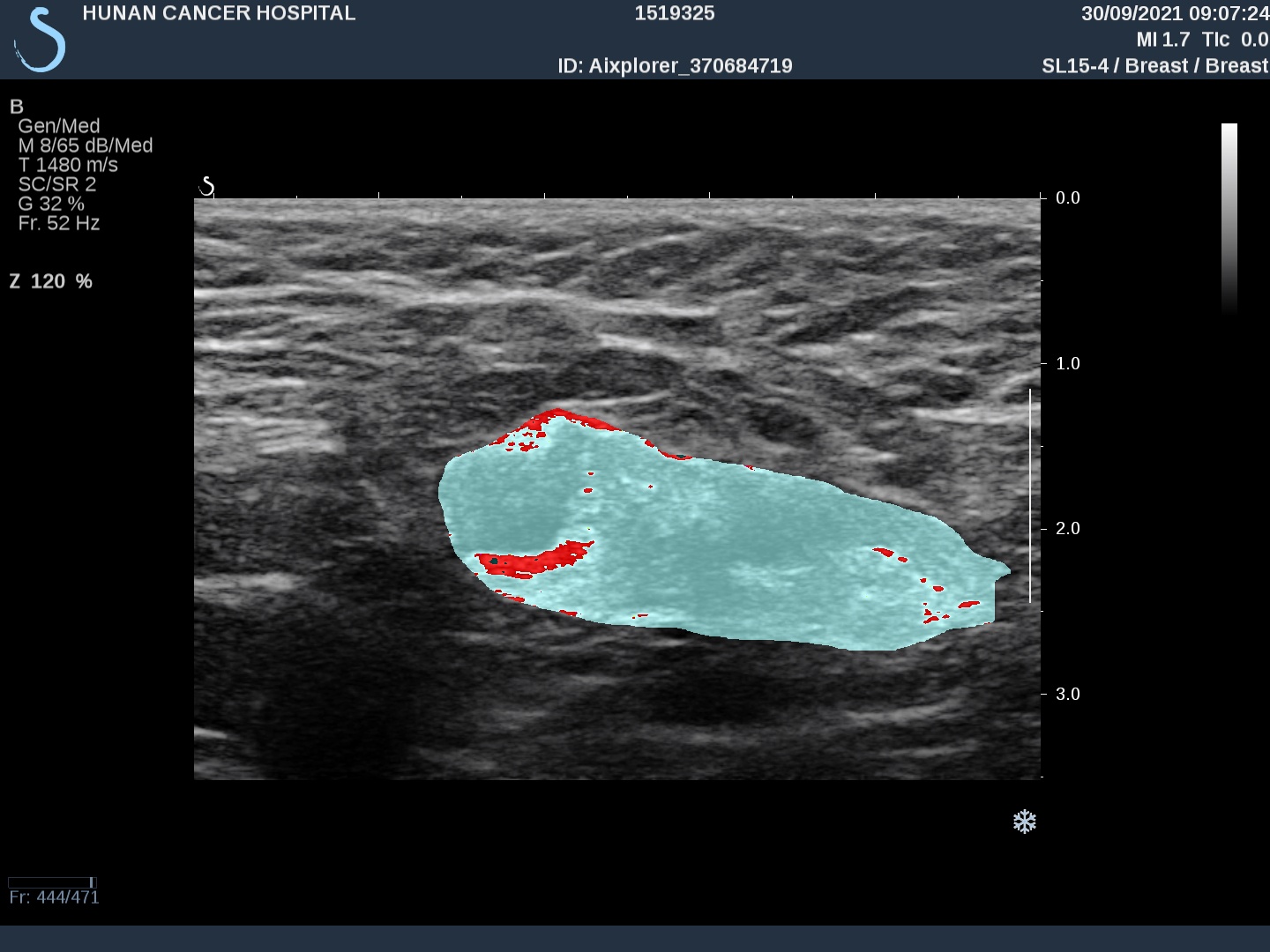

Supplement: Supplementary file 2 [file DataSheet_2.zip › ROI/1519325-1.jpg]

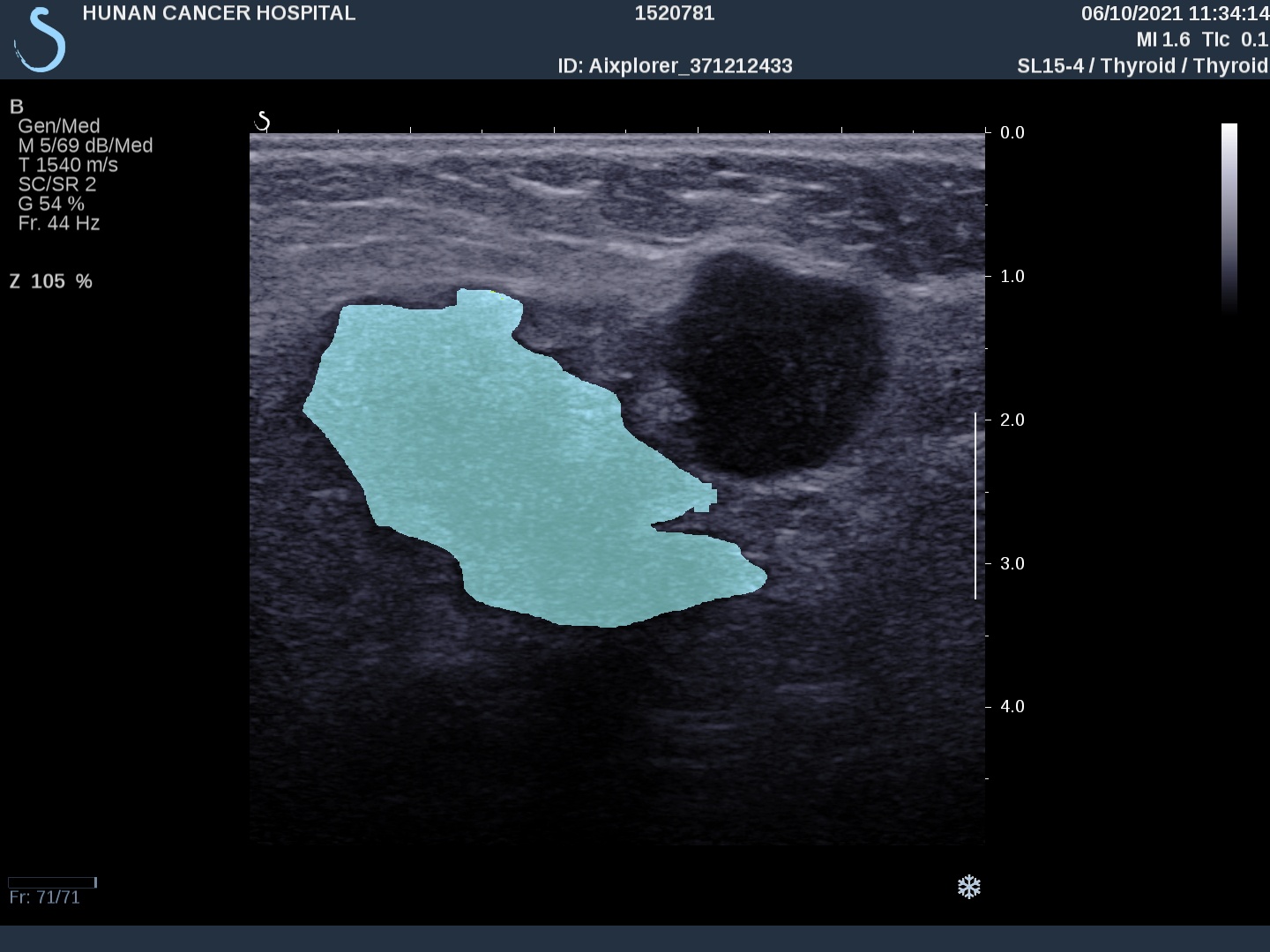

Supplement: Supplementary file 2 [file DataSheet_2.zip › ROI/1520781-1.jpg]

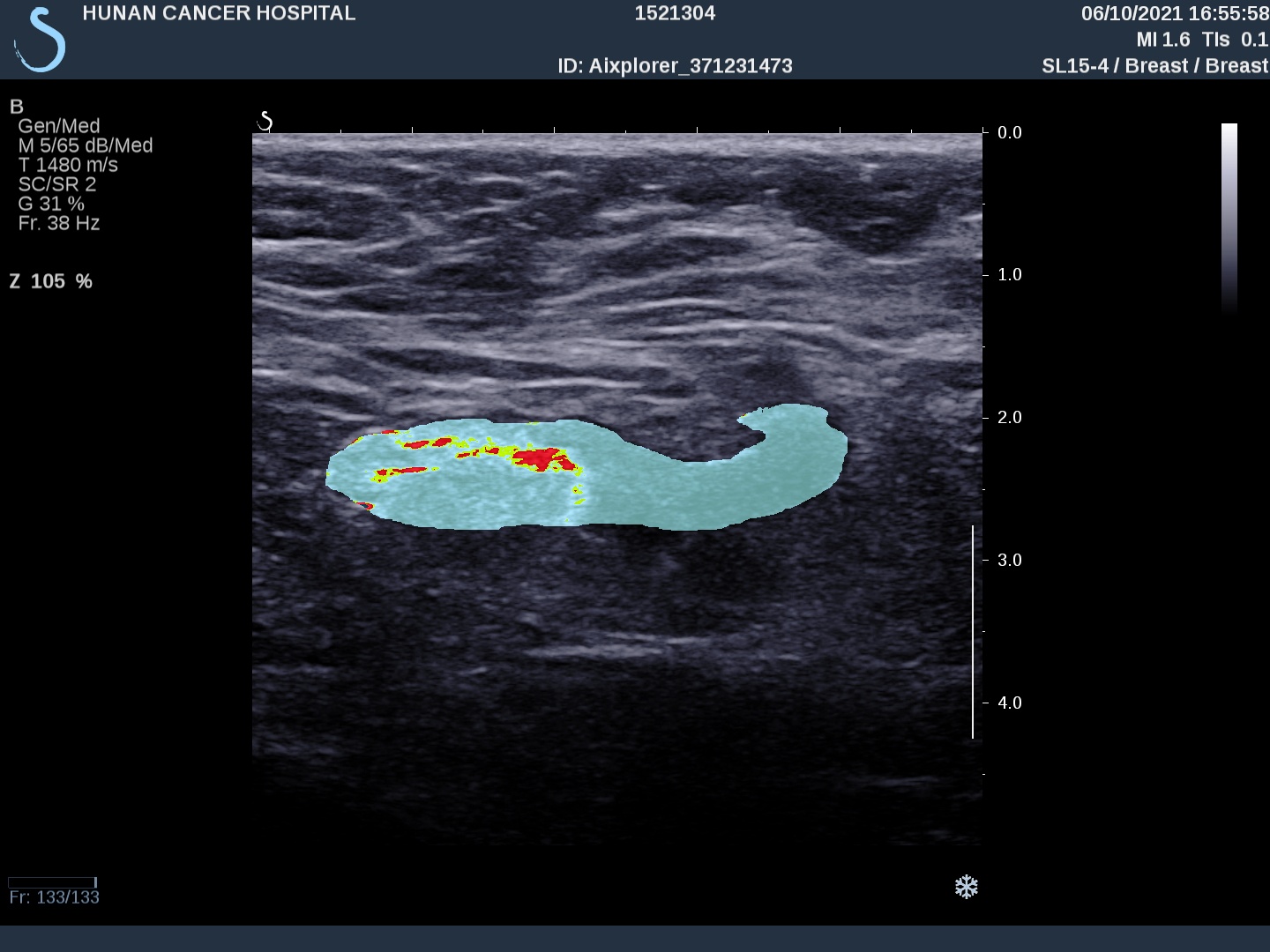

Supplement: Supplementary file 2 [file DataSheet_2.zip › ROI/1521304-2.jpg]

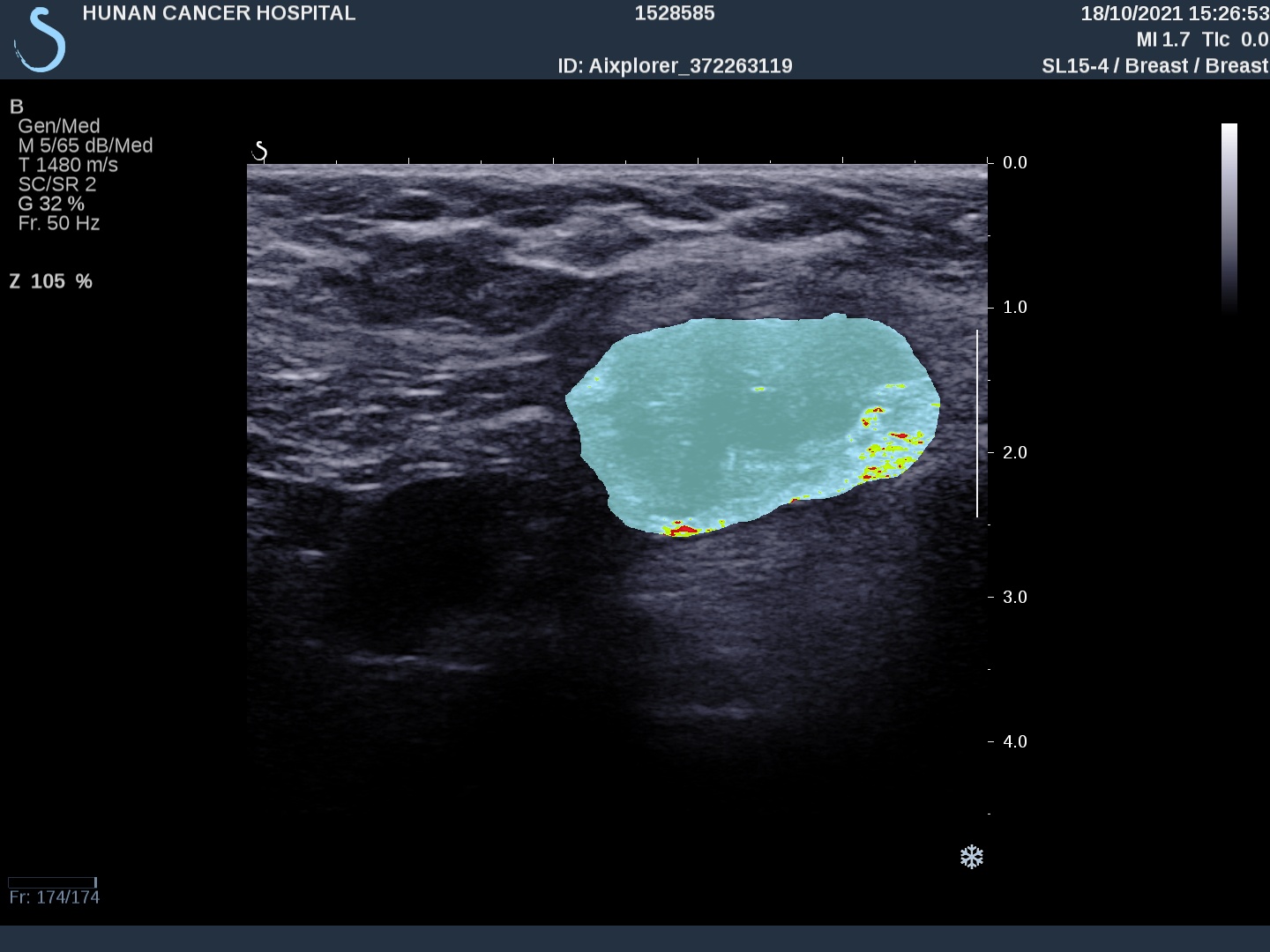

Supplement: Supplementary file 2 [file DataSheet_2.zip › ROI/1528585-1.jpg]

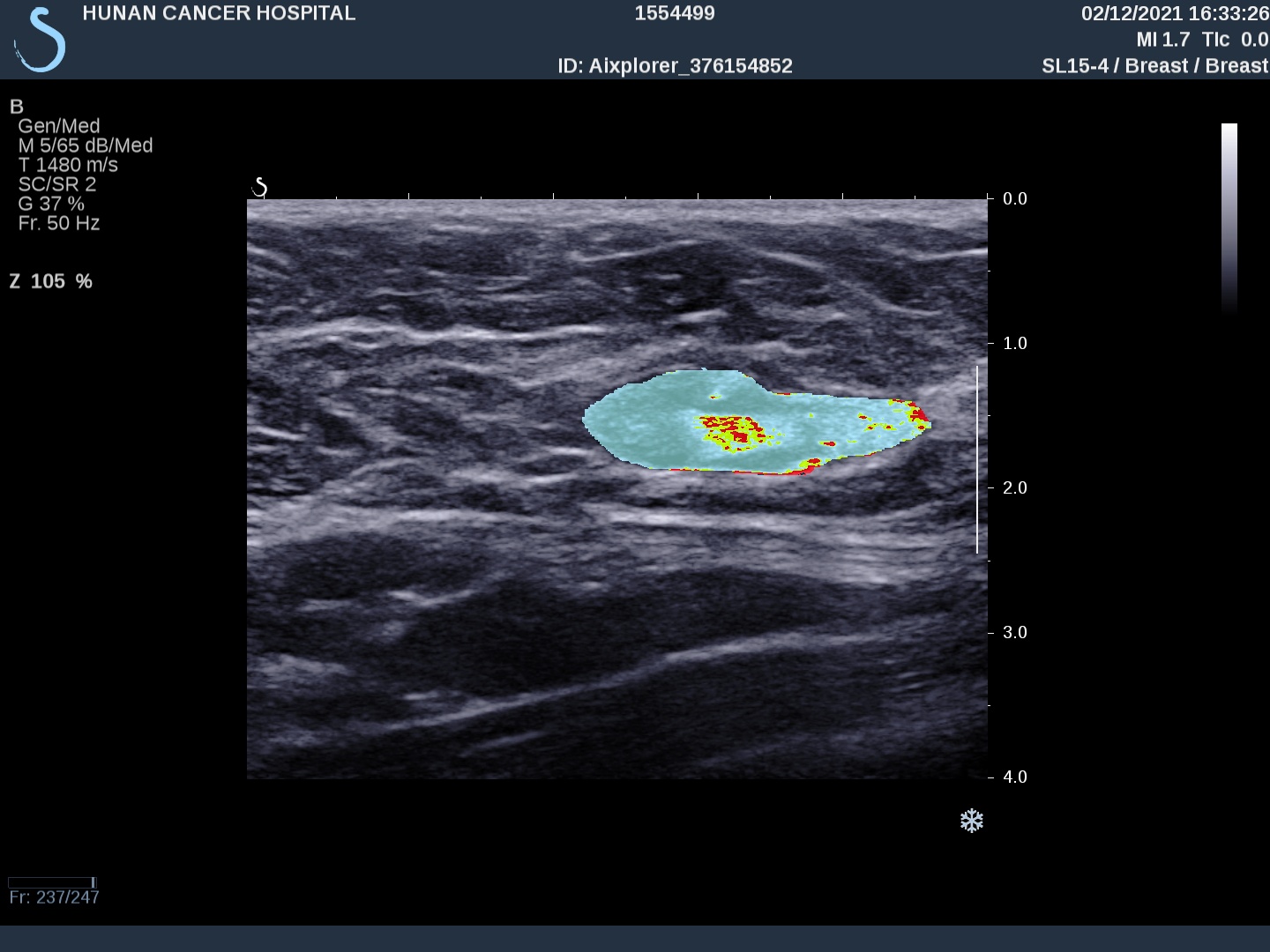

Supplement: Supplementary file 2 [file DataSheet_2.zip › ROI/1554499-2.jpg]

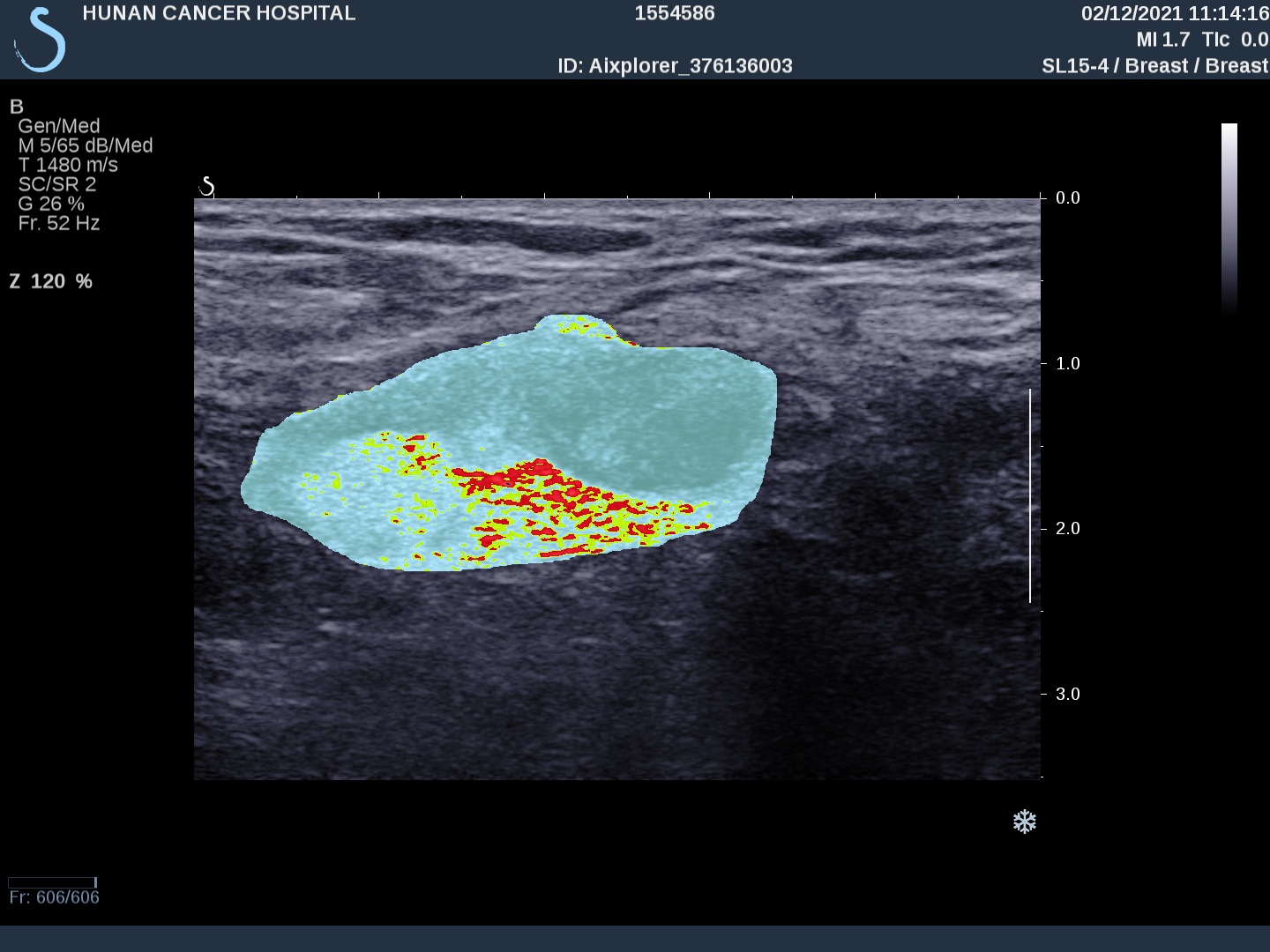

Supplement: Supplementary file 2 [file DataSheet_2.zip › ROI/1554586-1.jpg]

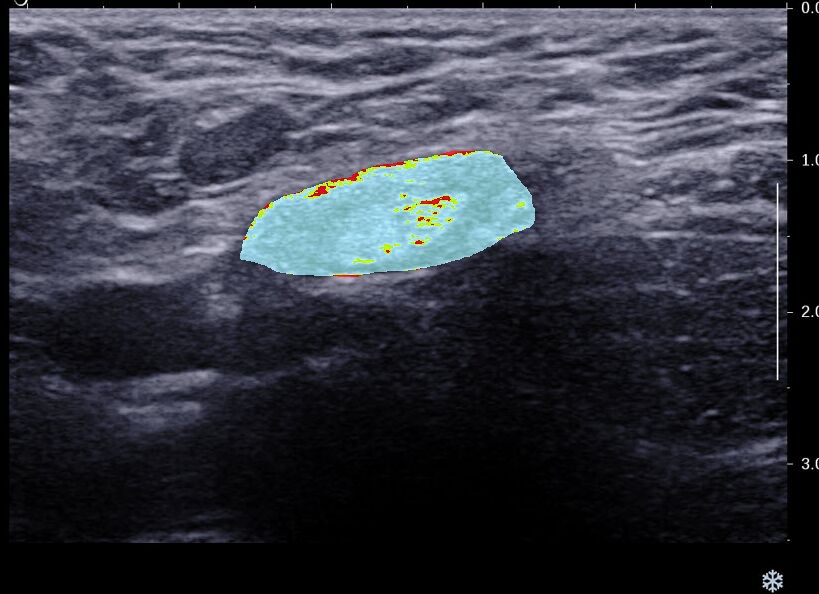

Supplement: Supplementary file 2 [file DataSheet_2.zip › ROI/1557229-3.jpg]

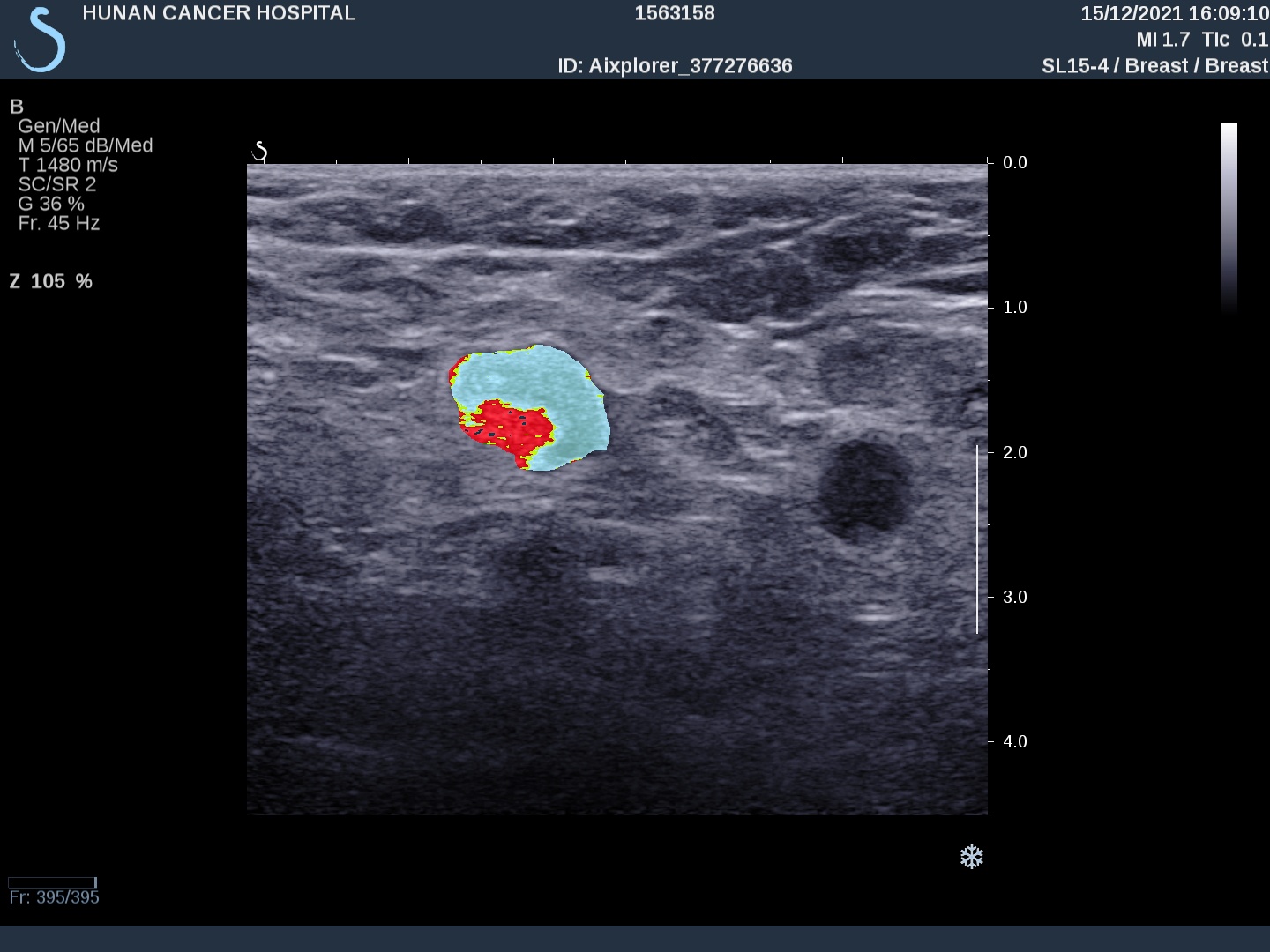

Supplement: Supplementary file 2 [file DataSheet_2.zip › ROI/1563158-1.jpg]

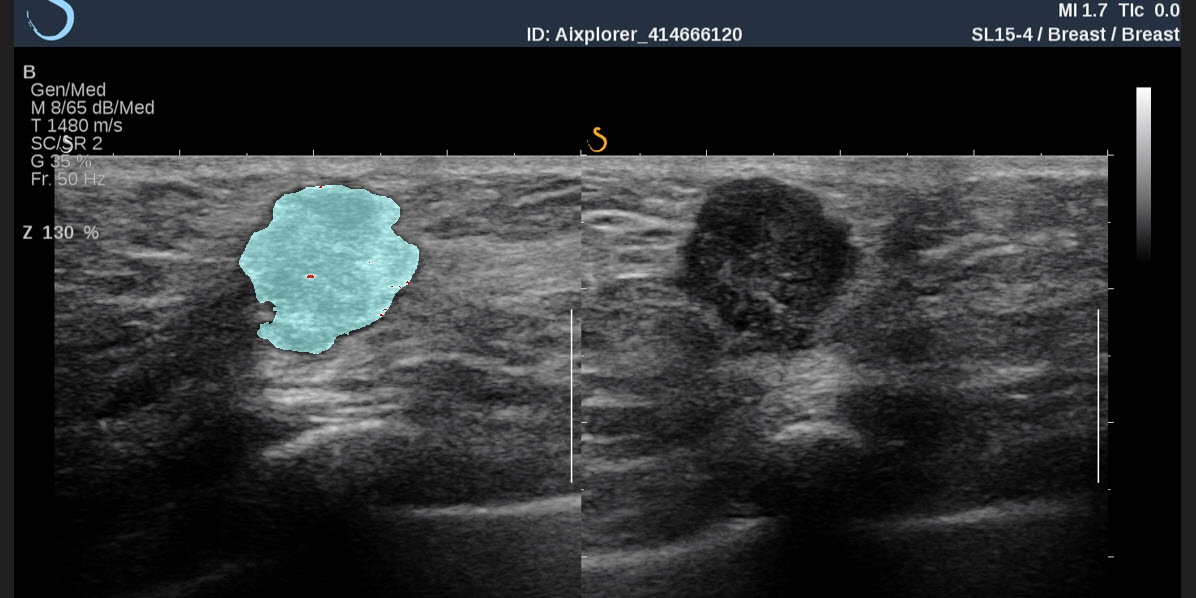

Supplement: Supplementary file 2 [file DataSheet_2.zip › ROI/1565820-1.jpg]

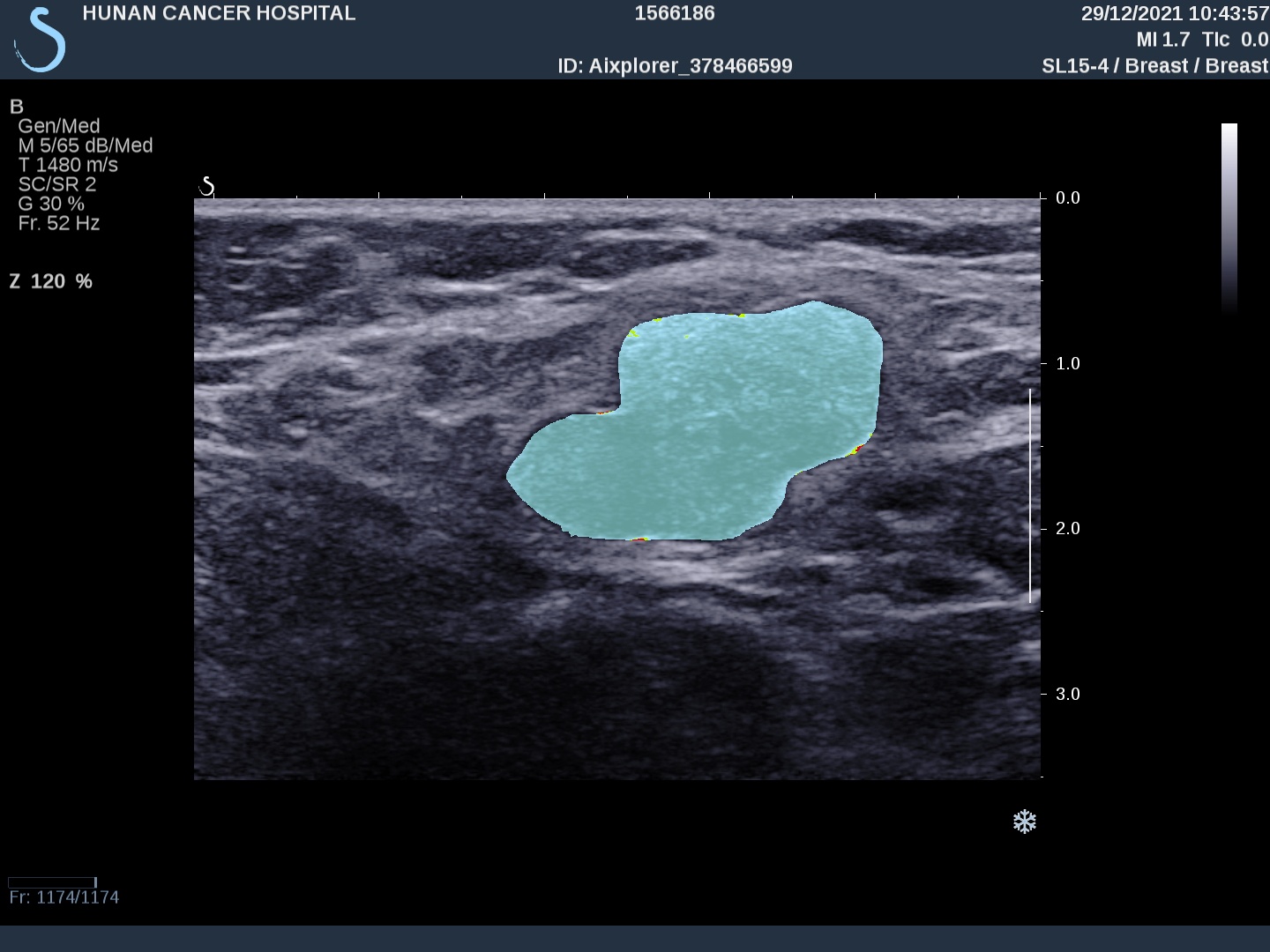

Supplement: Supplementary file 2 [file DataSheet_2.zip › ROI/1566186-1.jpg]

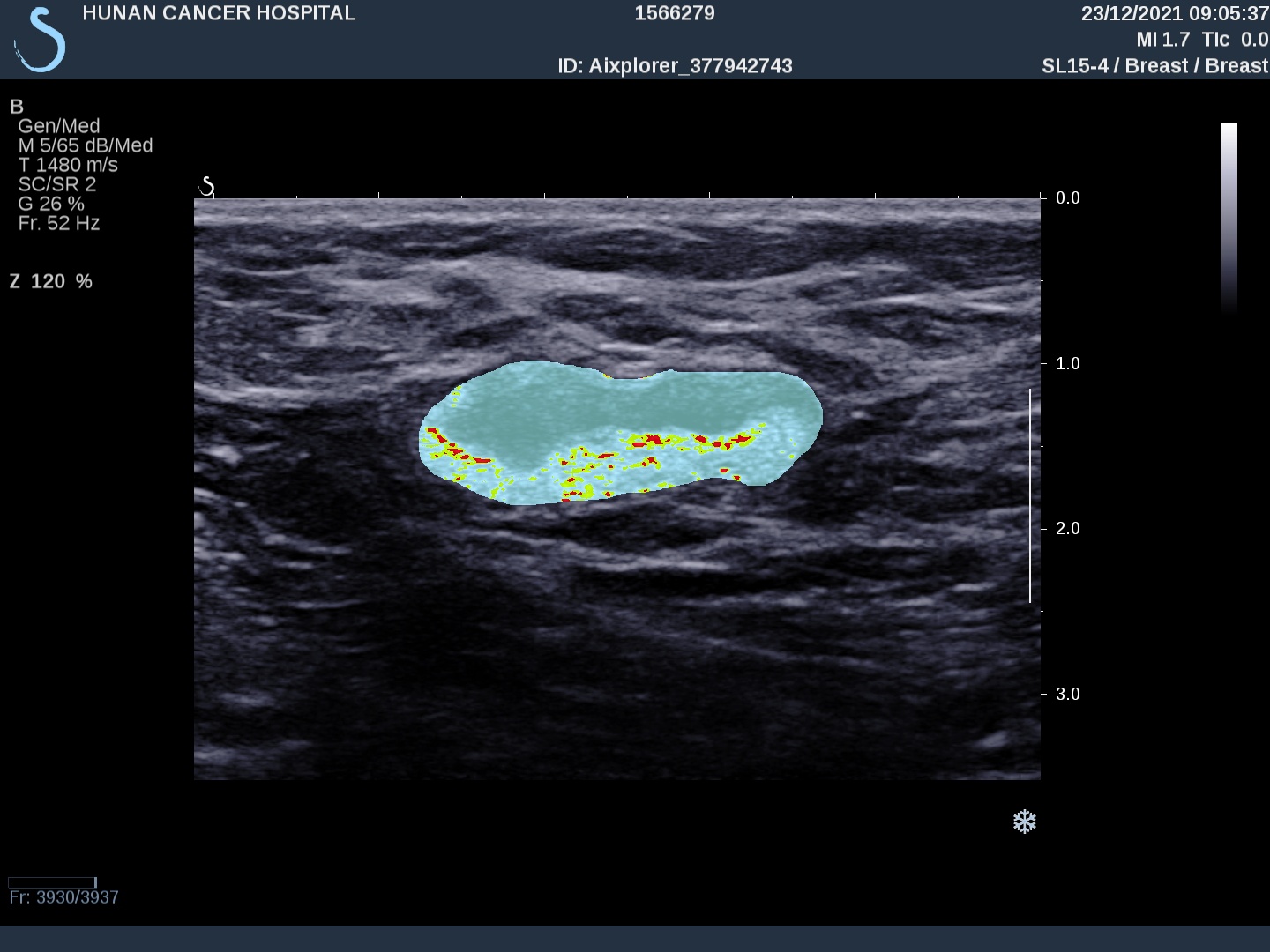

Supplement: Supplementary file 2 [file DataSheet_2.zip › ROI/1566279-1.jpg]

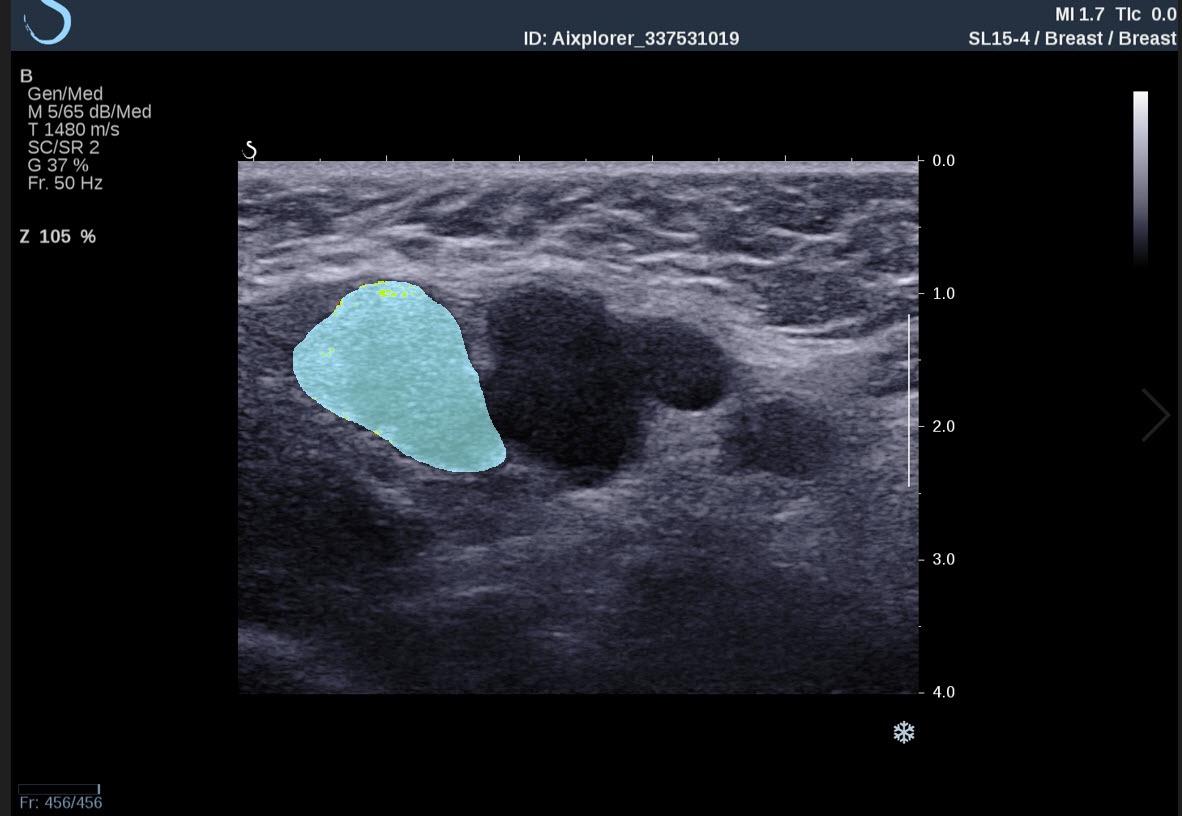

Supplement: Supplementary file 2 [file DataSheet_2.zip › ROI/1569142-3.jpg]

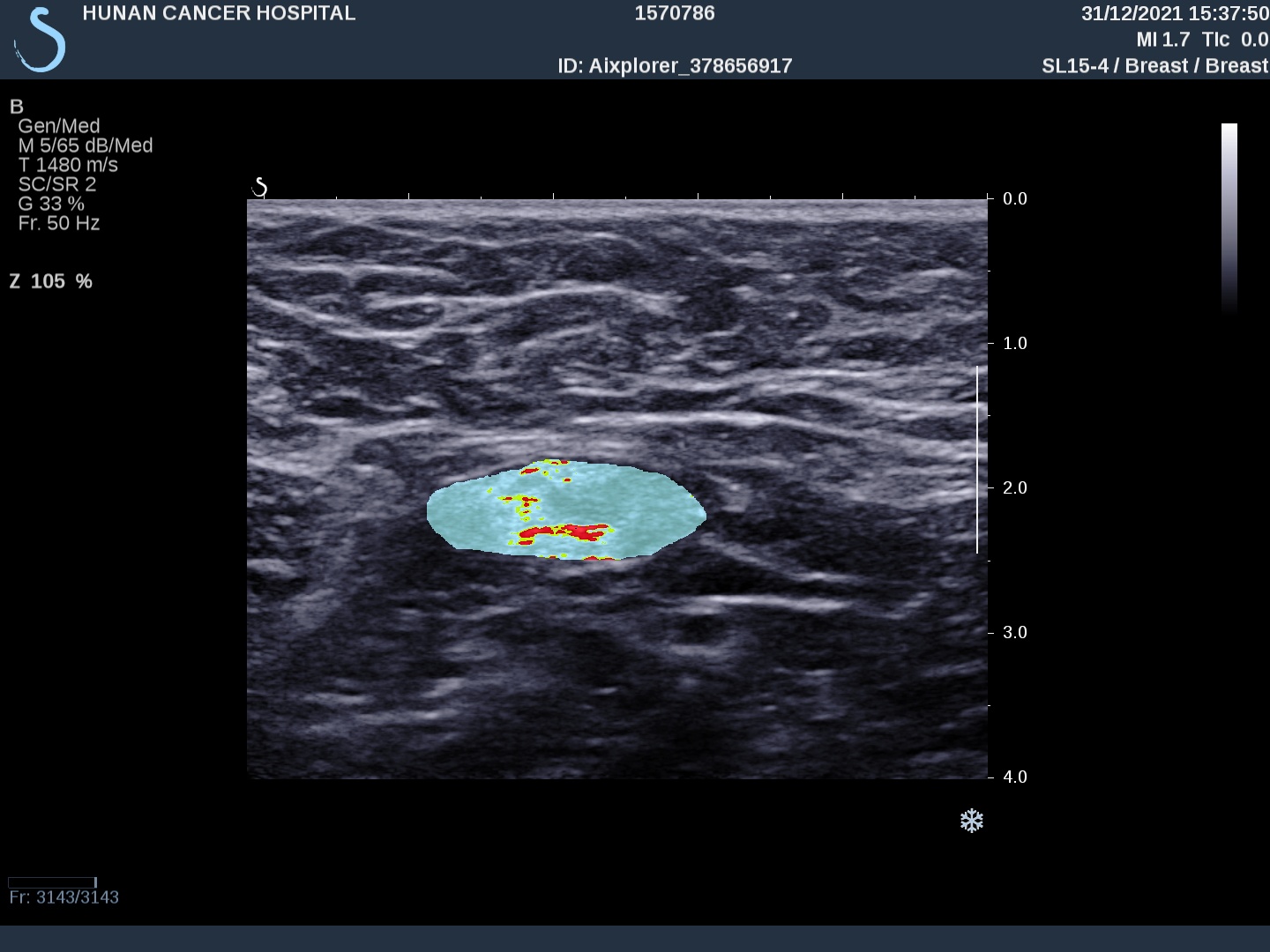

Supplement: Supplementary file 2 [file DataSheet_2.zip › ROI/1570768-1.jpg]

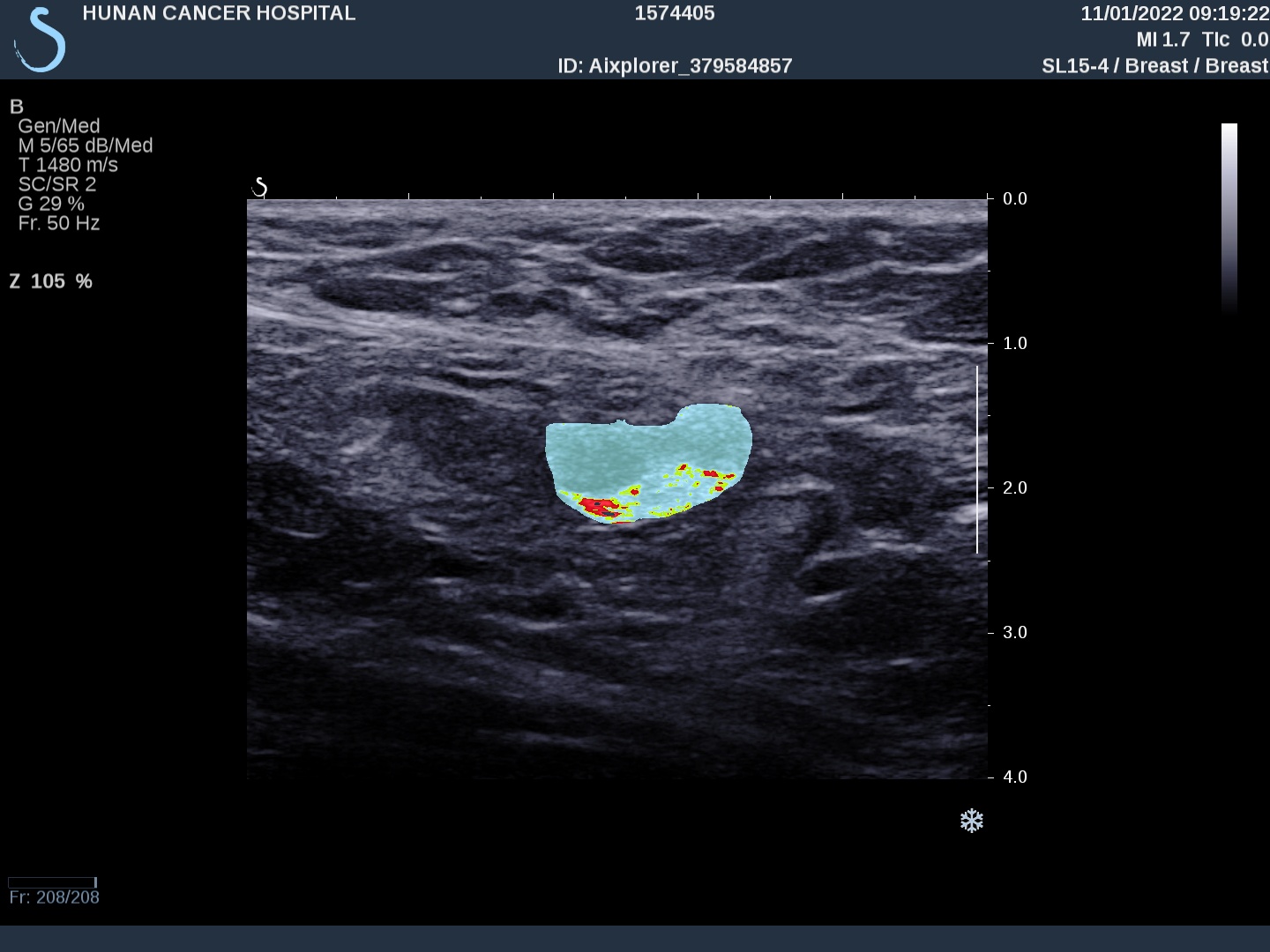

Supplement: Supplementary file 2 [file DataSheet_2.zip › ROI/1574405-1.jpg]

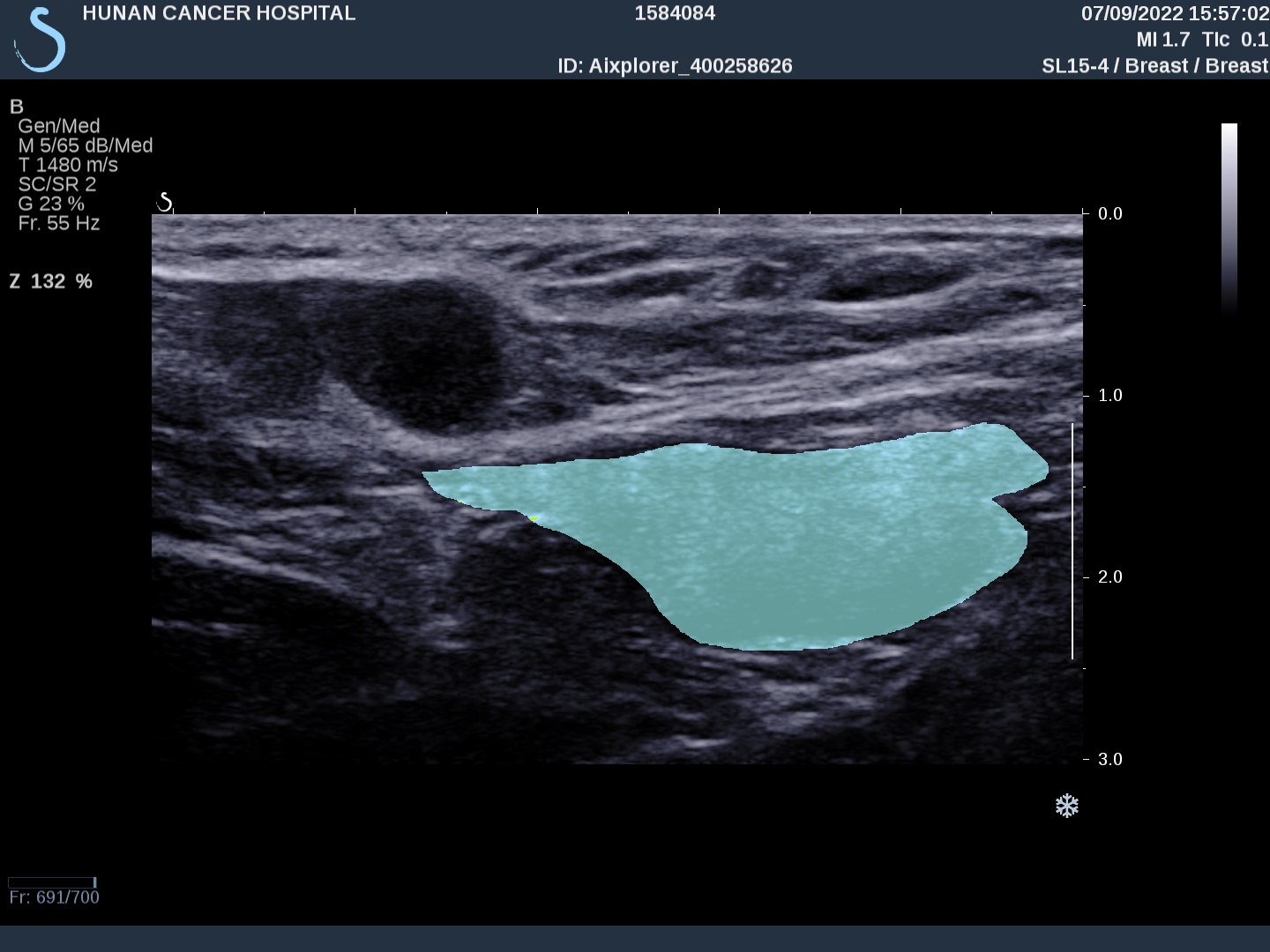

Supplement: Supplementary file 2 [file DataSheet_2.zip › ROI/1584084-1.jpg]

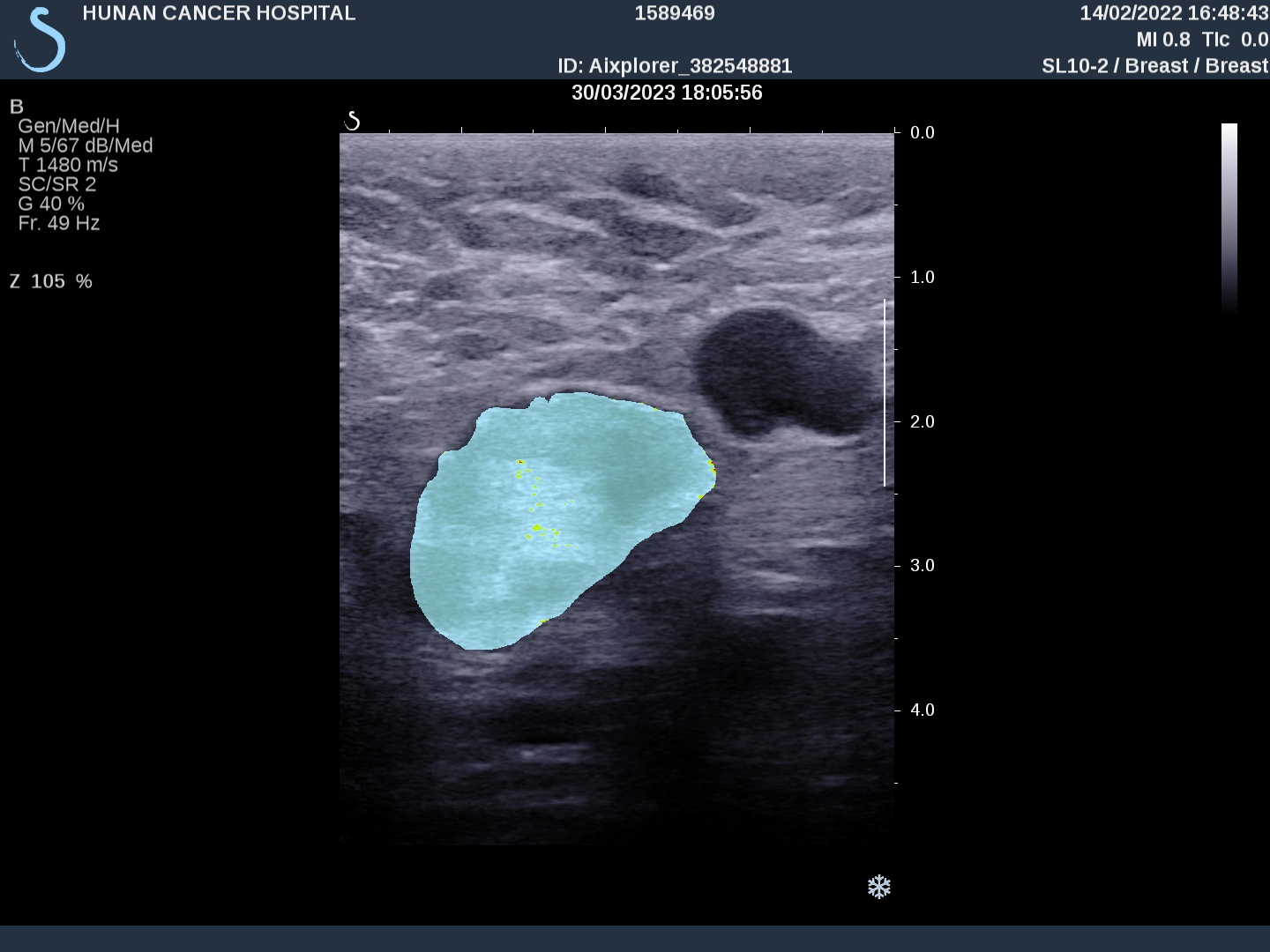

Supplement: Supplementary file 2 [file DataSheet_2.zip › ROI/1589469-1.jpg]

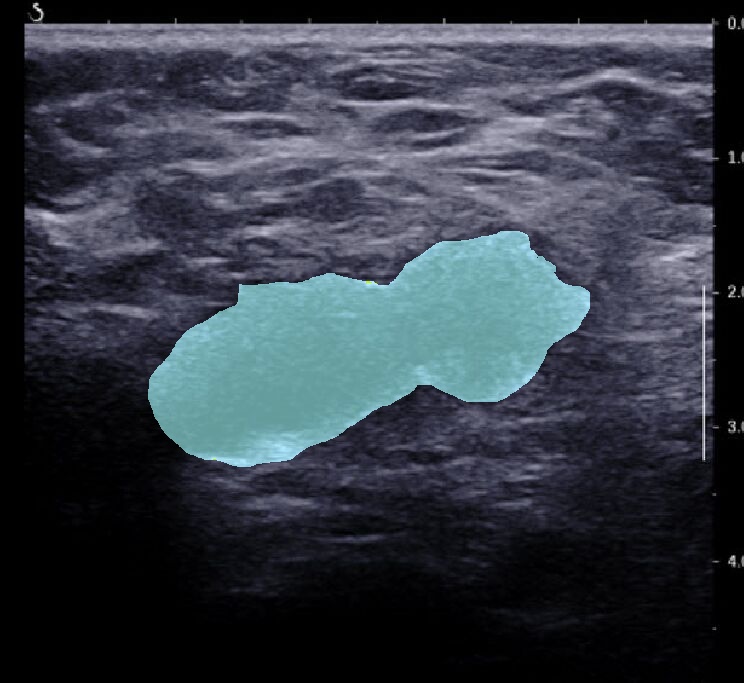

Supplement: Supplementary file 2 [file DataSheet_2.zip › ROI/1590233-1.jpg]

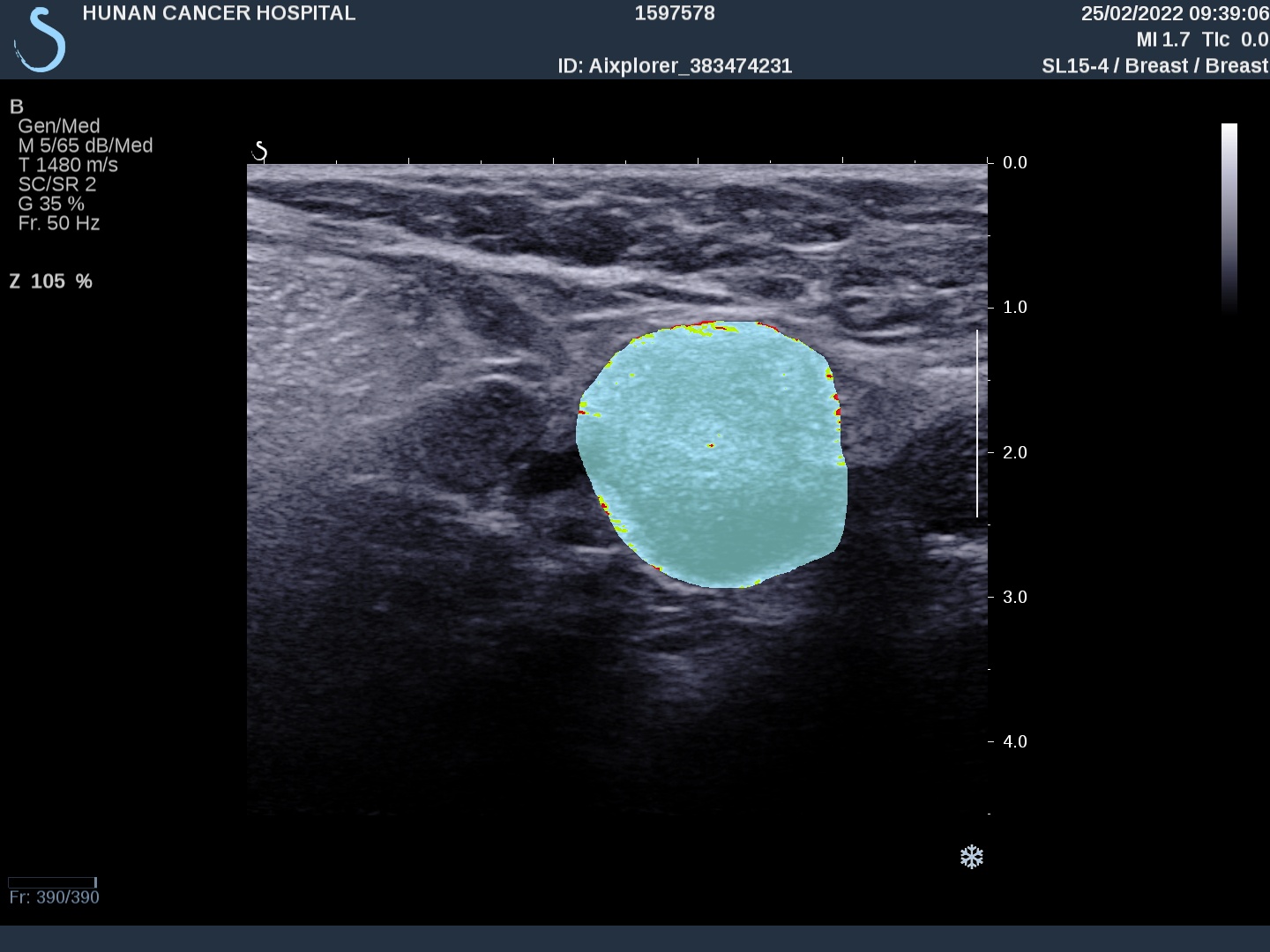

Supplement: Supplementary file 2 [file DataSheet_2.zip › ROI/1597578-1.jpg]

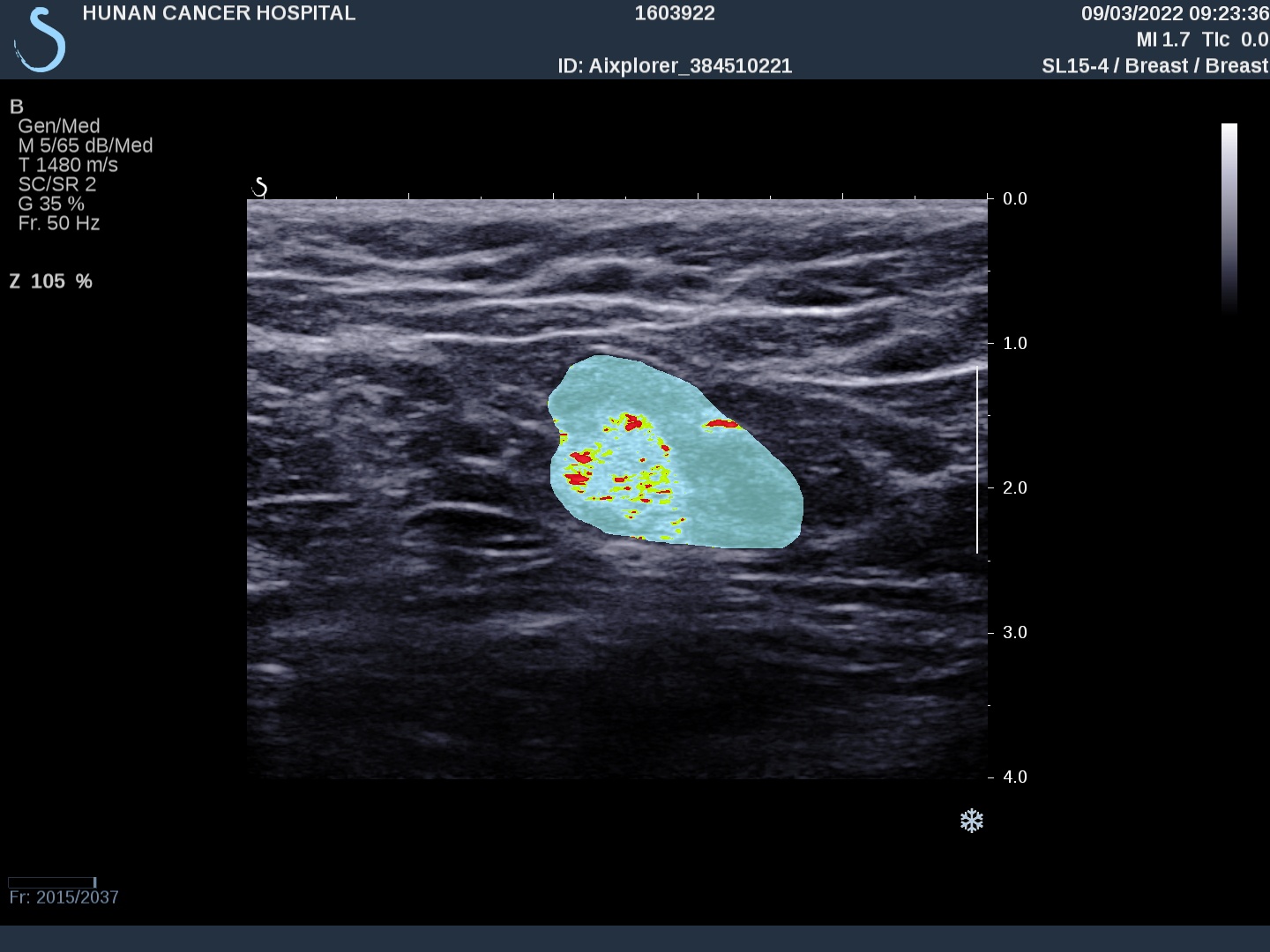

Supplement: Supplementary file 2 [file DataSheet_2.zip › ROI/1603922-1.jpg]

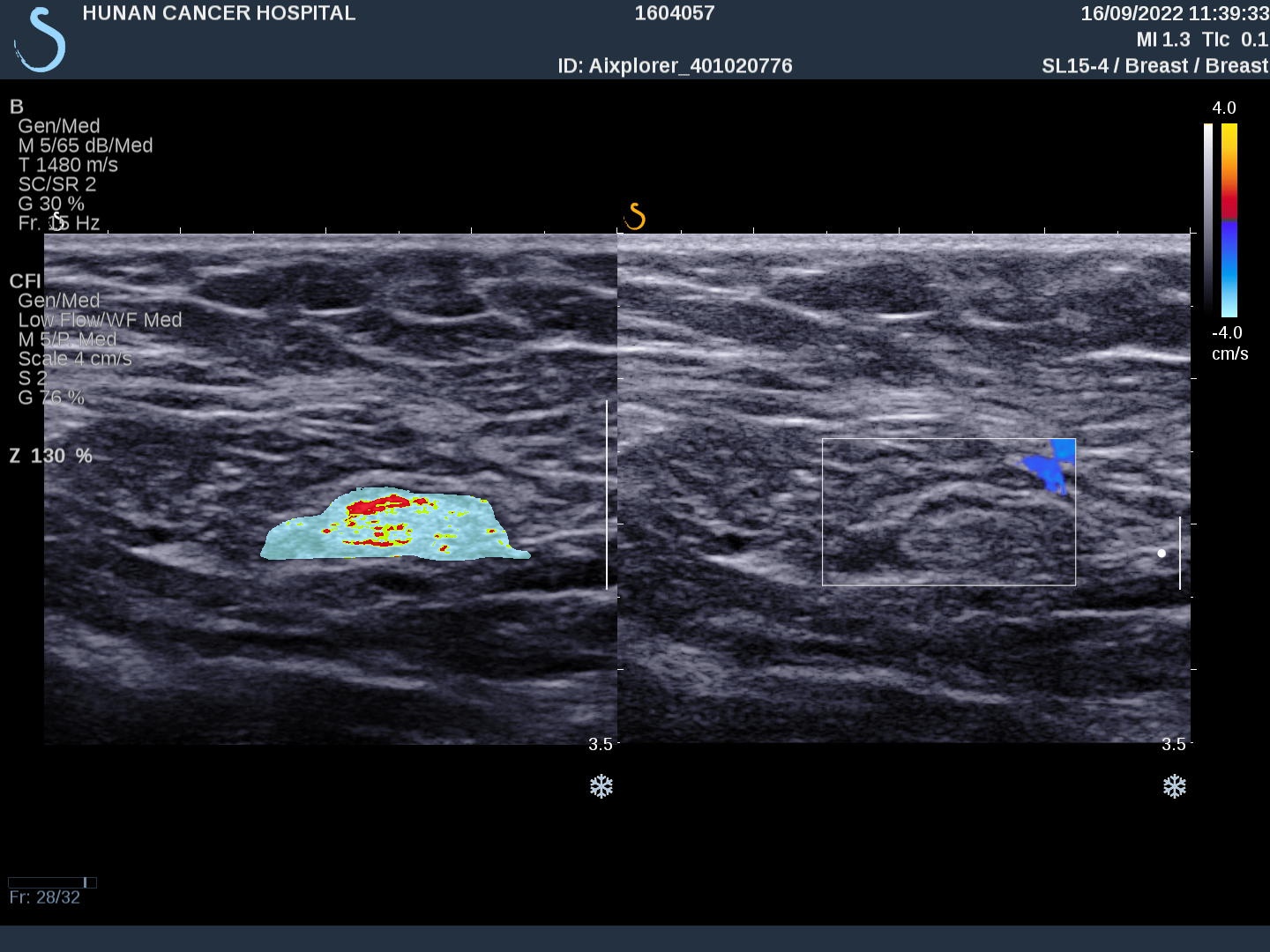

Supplement: Supplementary file 2 [file DataSheet_2.zip › ROI/1604057-1.jpg]

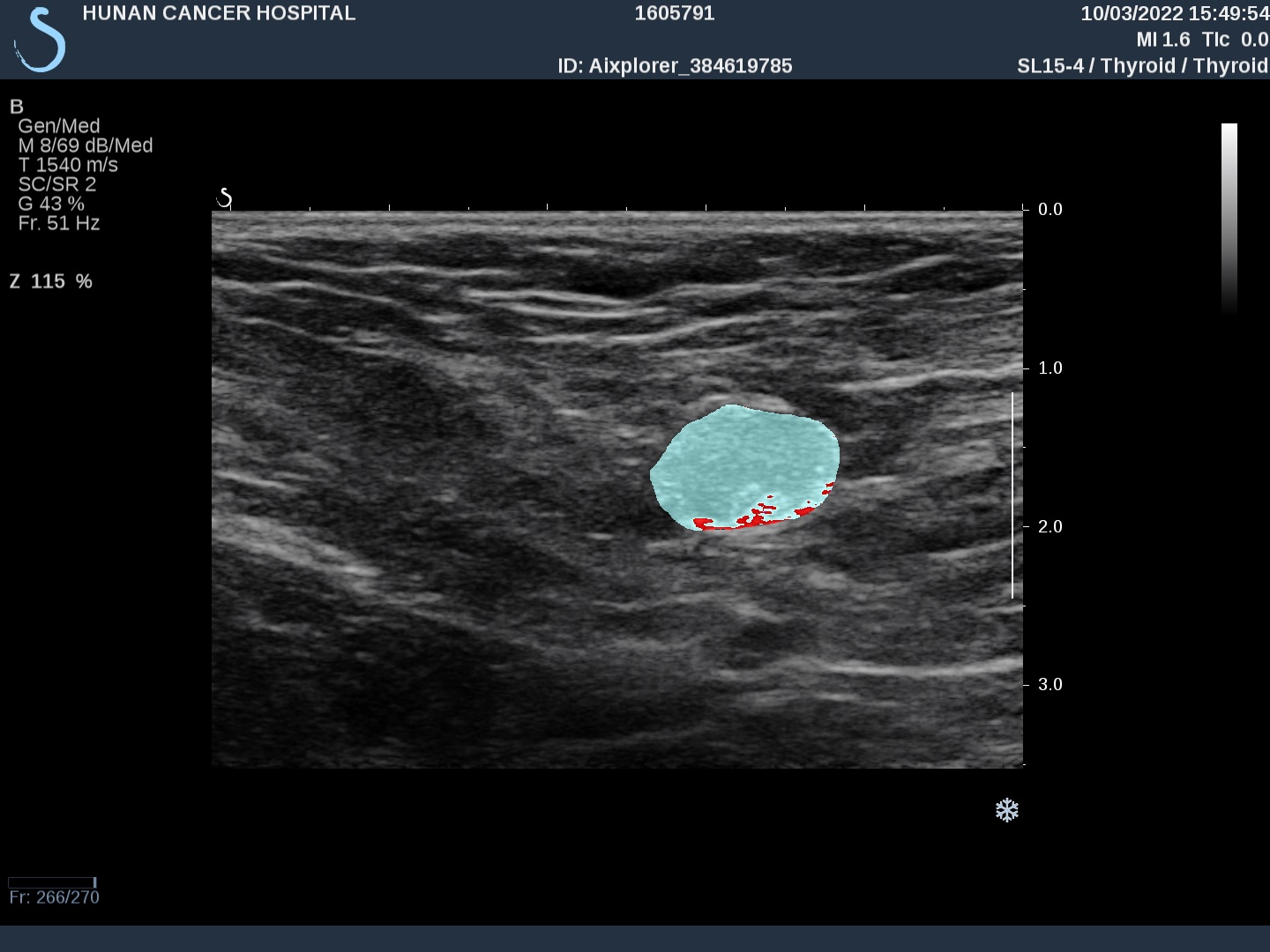

Supplement: Supplementary file 2 [file DataSheet_2.zip › ROI/1605791-1.jpg]

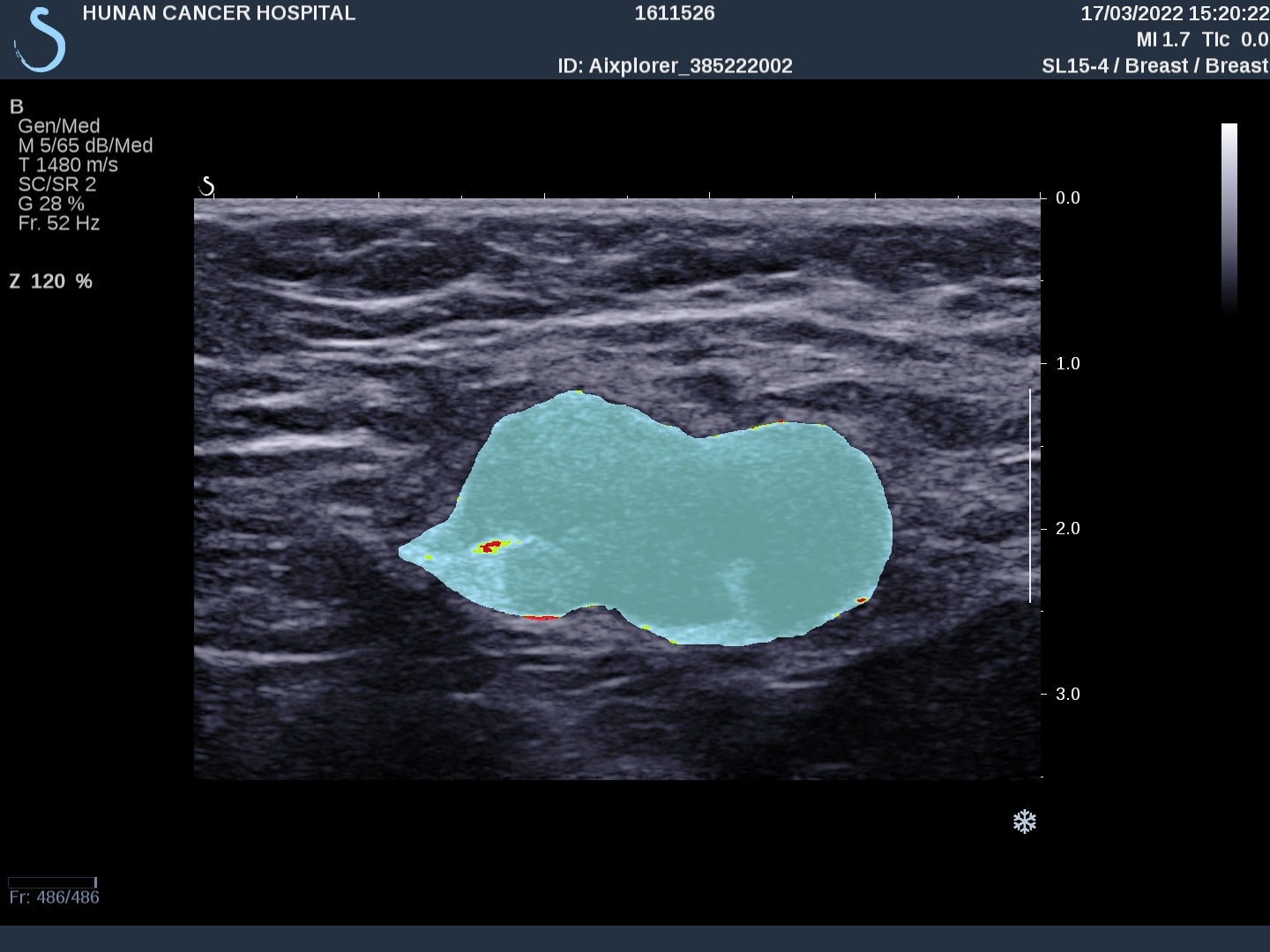

Supplement: Supplementary file 2 [file DataSheet_2.zip › ROI/1611526-1.jpg]

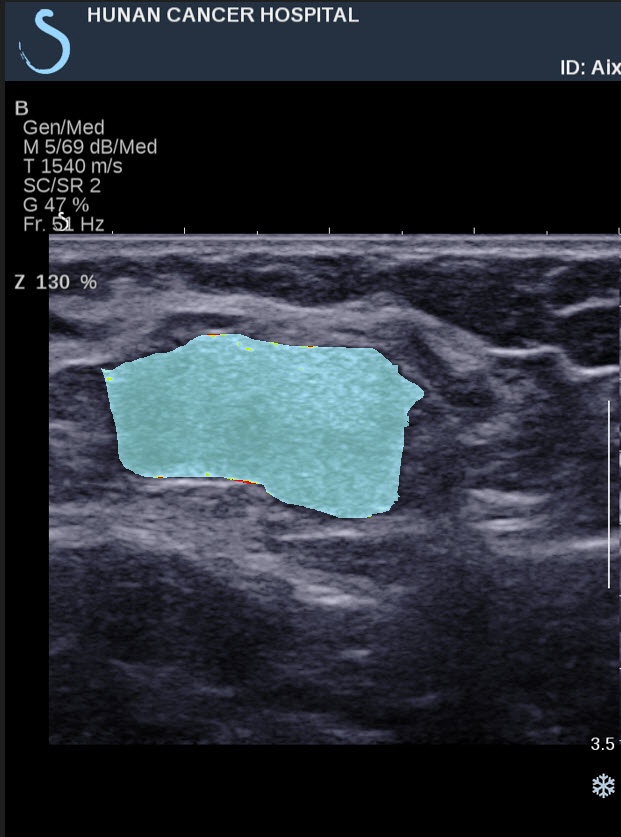

Supplement: Supplementary file 2 [file DataSheet_2.zip › ROI/1612412-1.jpg]

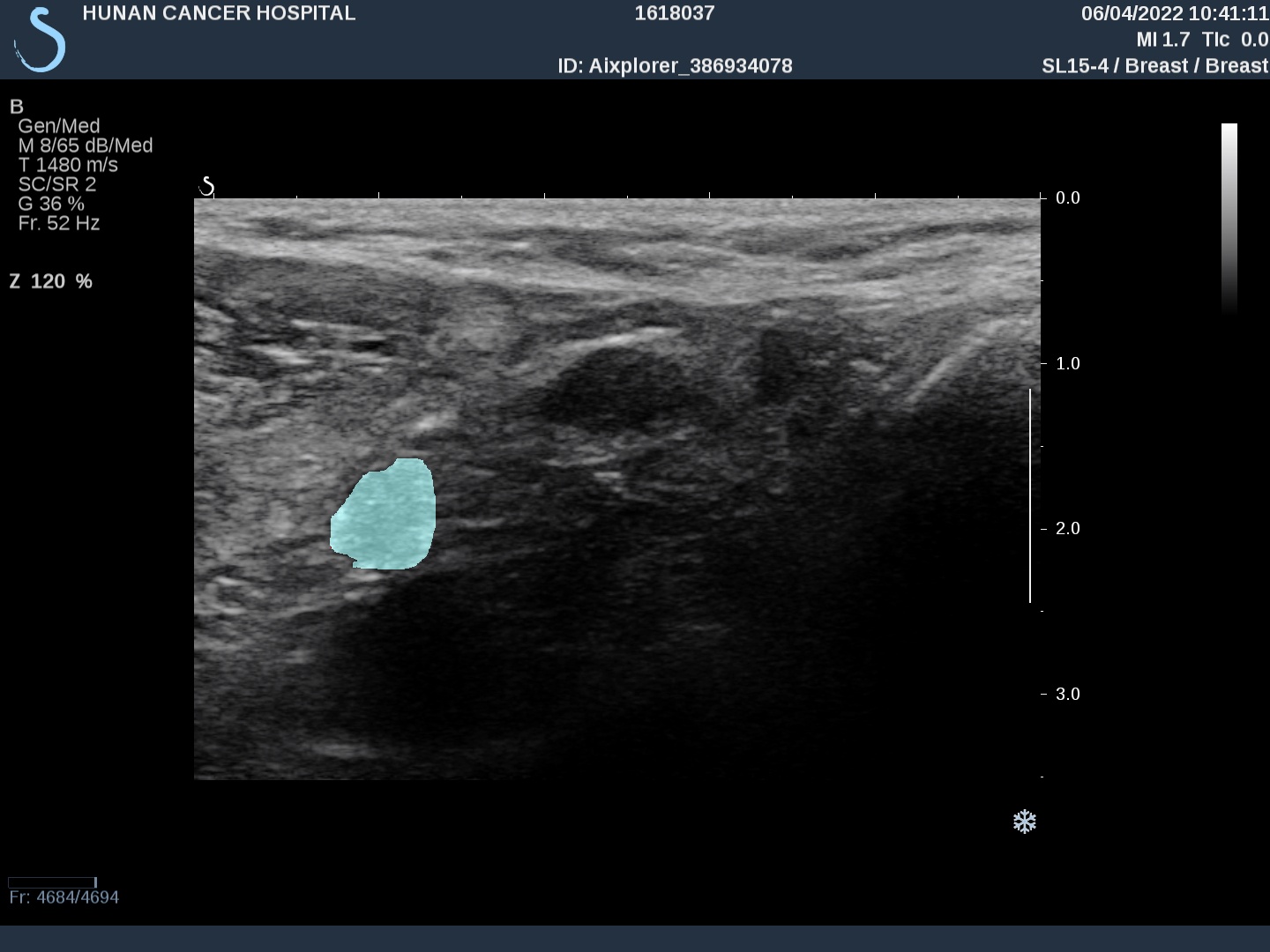

Supplement: Supplementary file 2 [file DataSheet_2.zip › ROI/1618037-1.jpg]

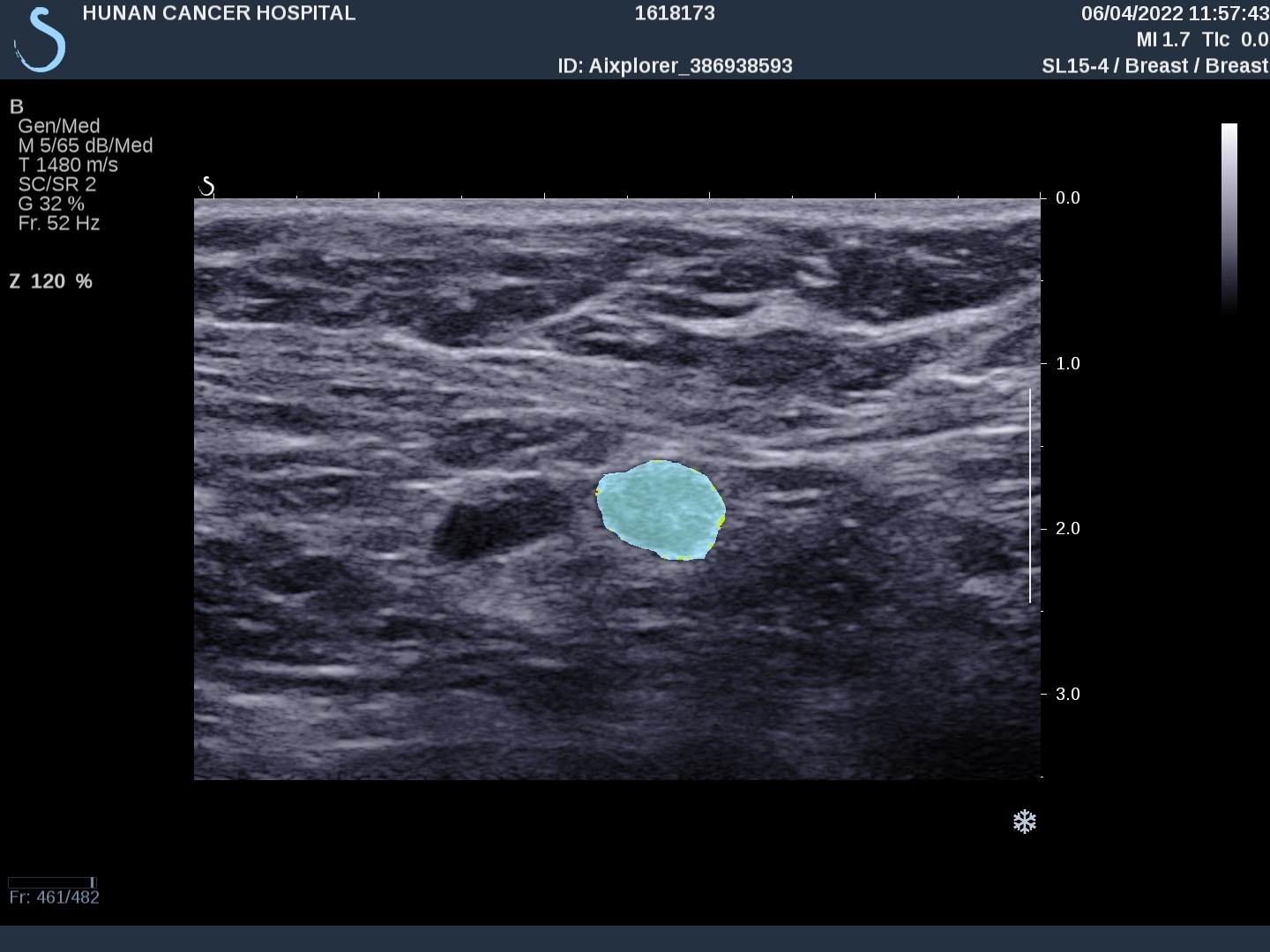

Supplement: Supplementary file 2 [file DataSheet_2.zip › ROI/1618173-1.jpg]

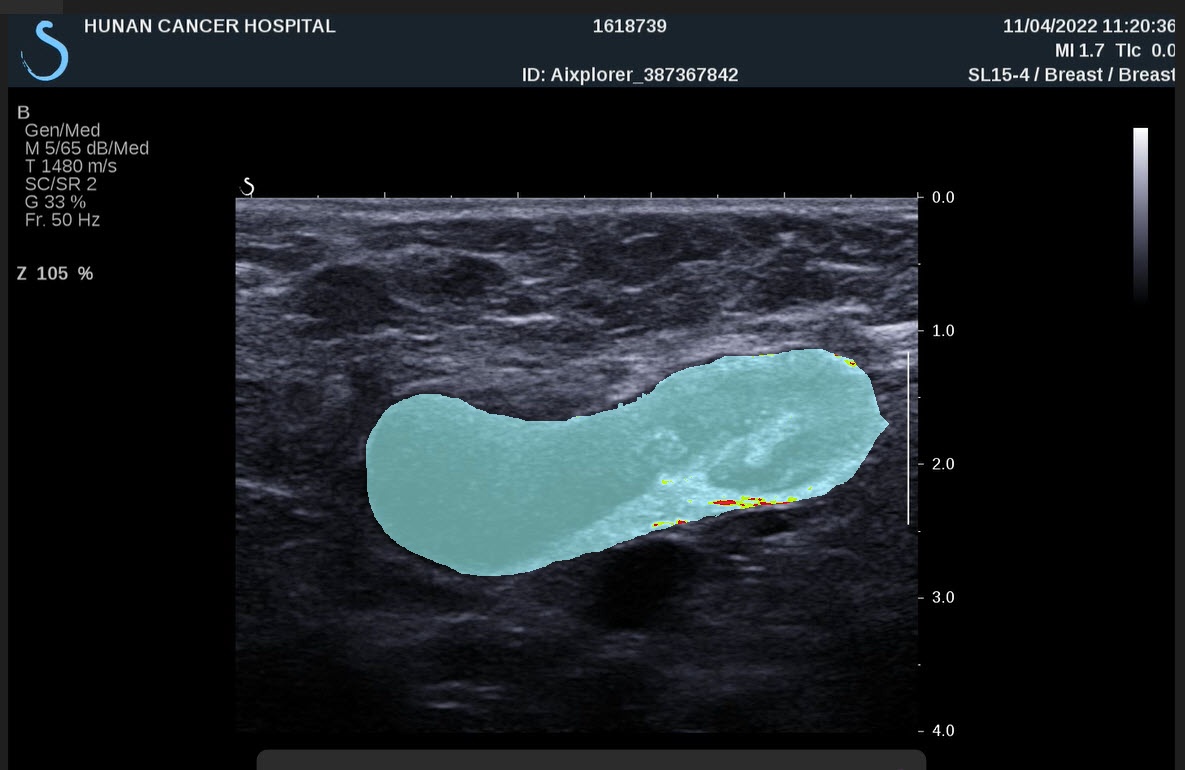

Supplement: Supplementary file 2 [file DataSheet_2.zip › ROI/1618739-1.jpg]

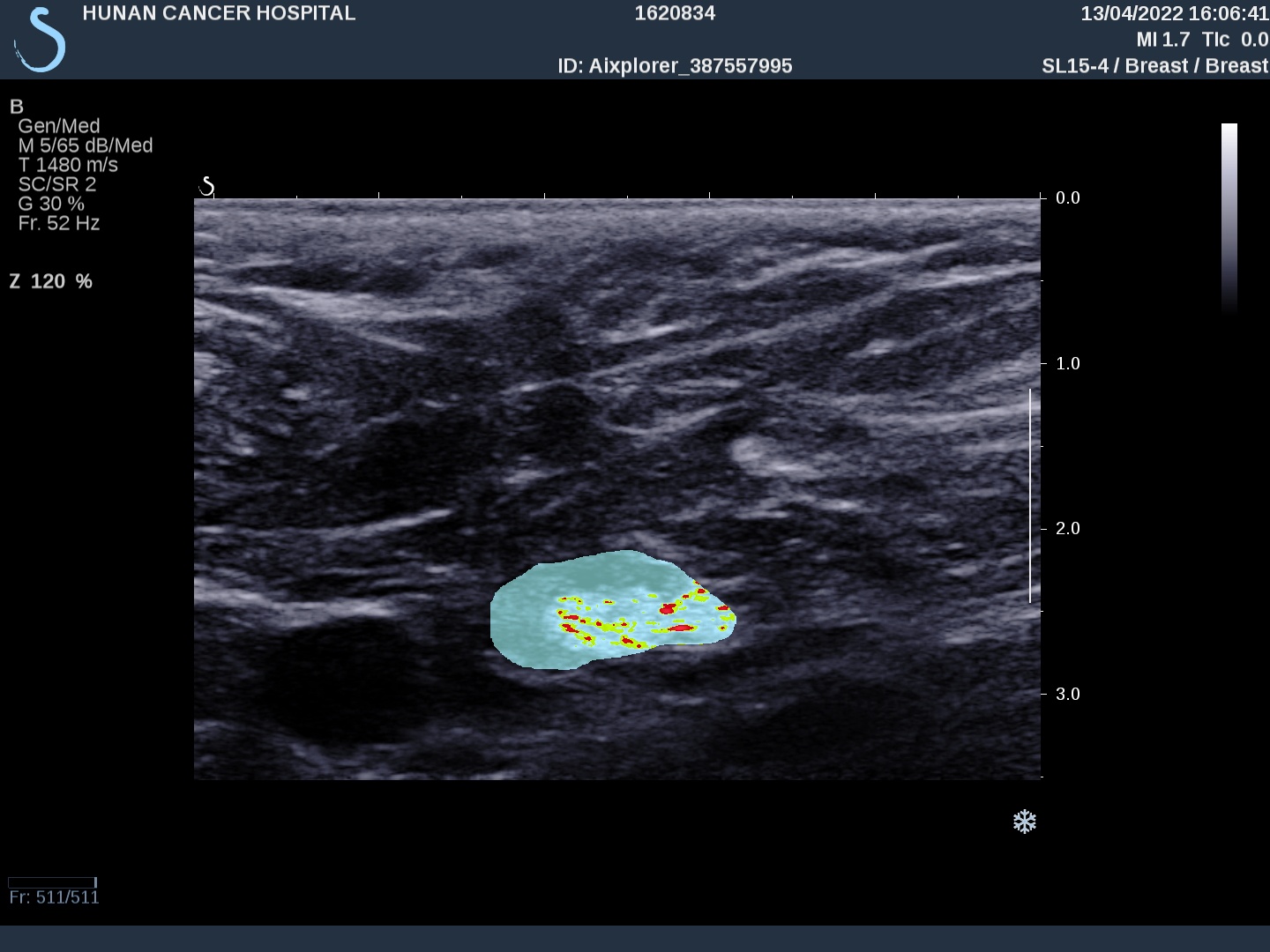

Supplement: Supplementary file 2 [file DataSheet_2.zip › ROI/1620834-1.jpg]

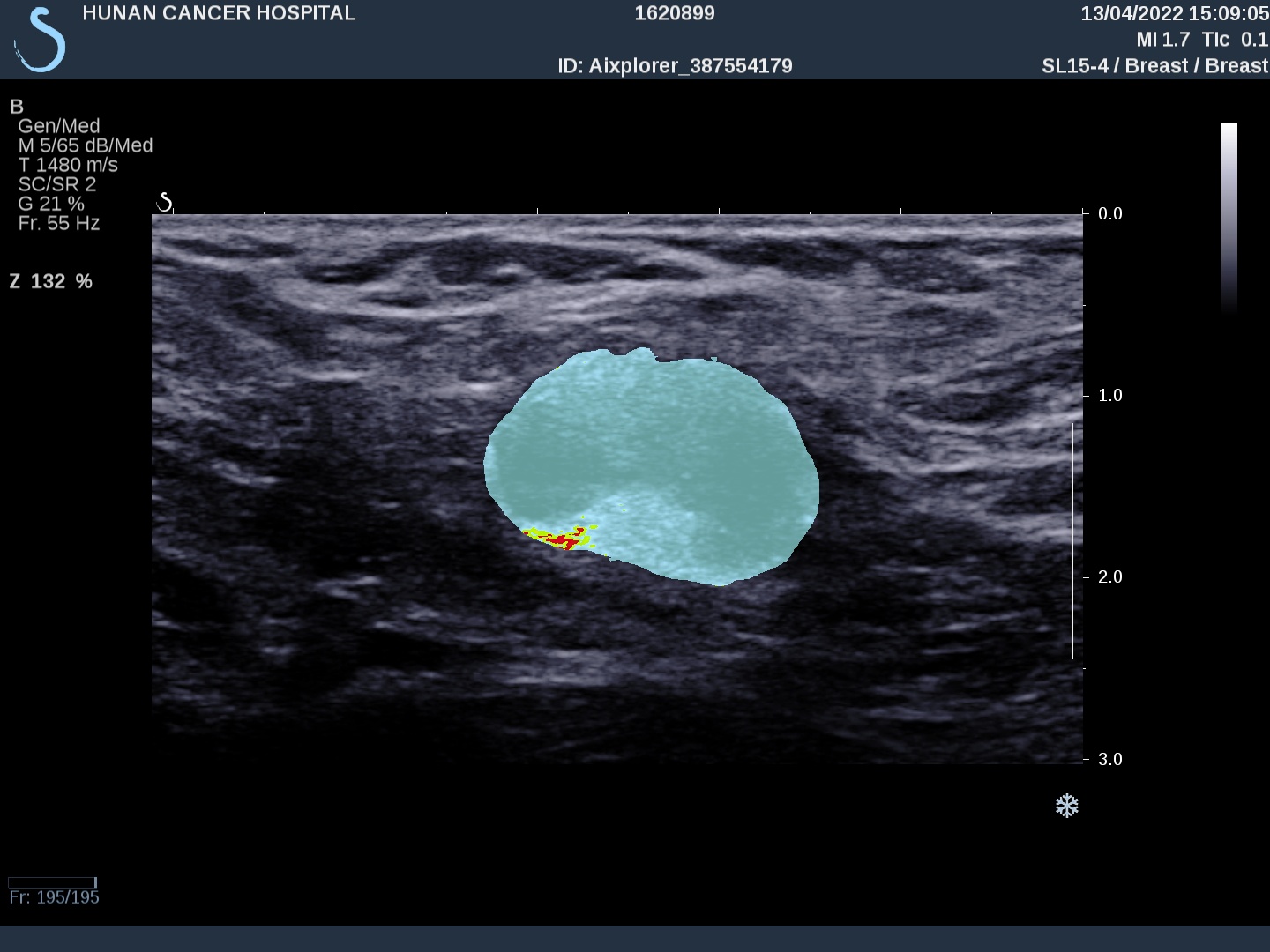

Supplement: Supplementary file 2 [file DataSheet_2.zip › ROI/1620899-1.jpg]

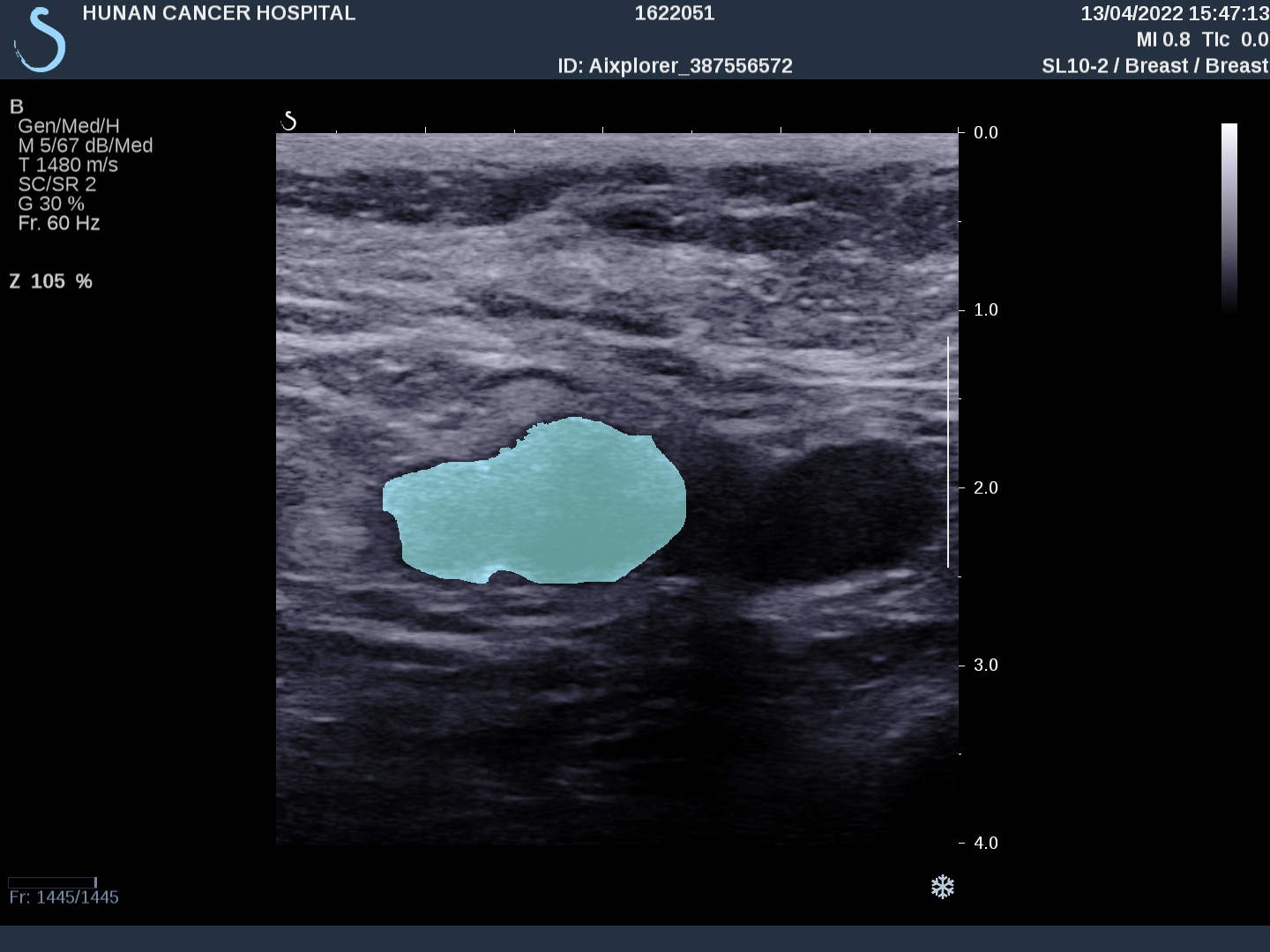

Supplement: Supplementary file 2 [file DataSheet_2.zip › ROI/1622051-1.jpg]

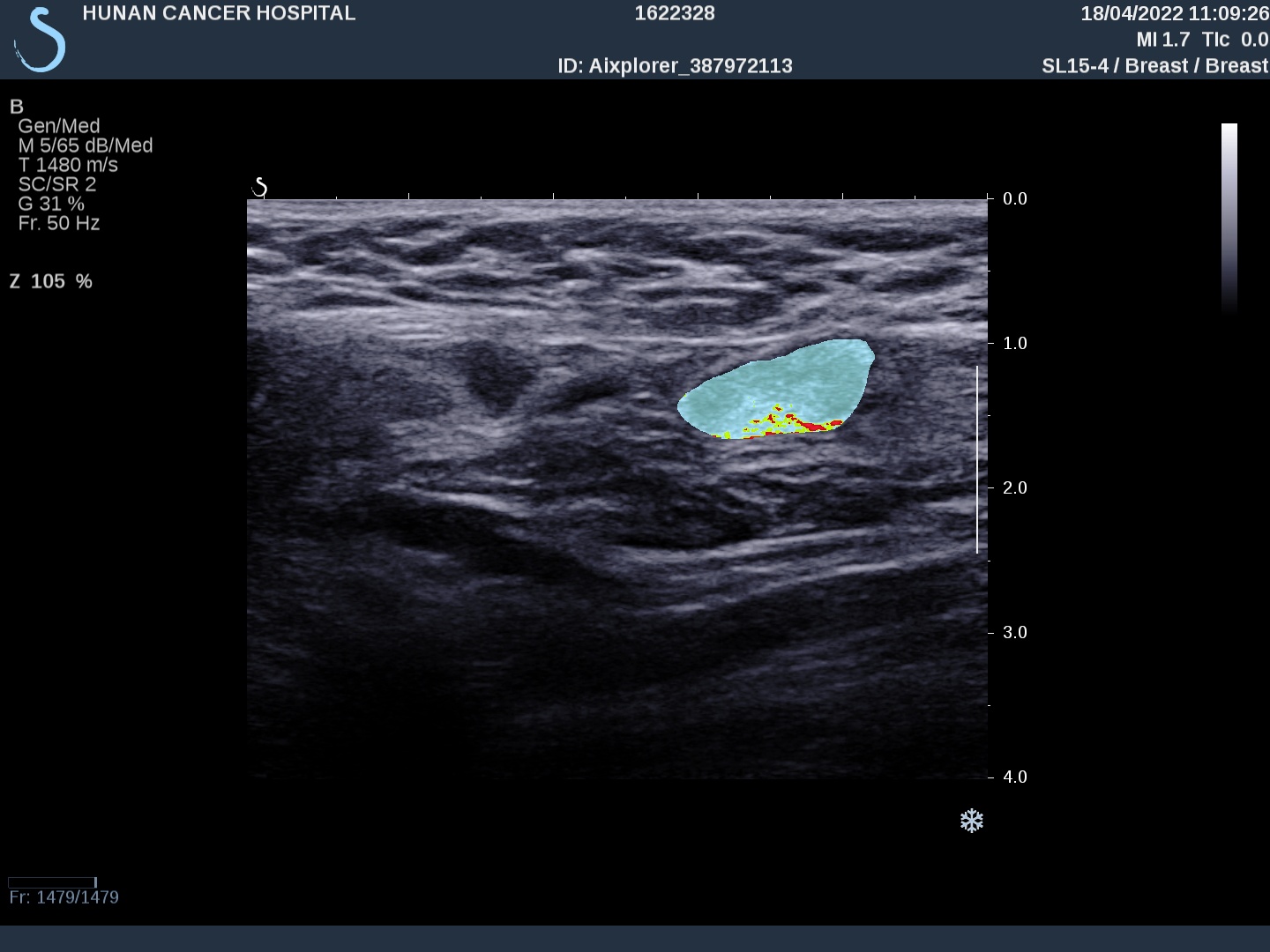

Supplement: Supplementary file 2 [file DataSheet_2.zip › ROI/1622328-1.jpg]

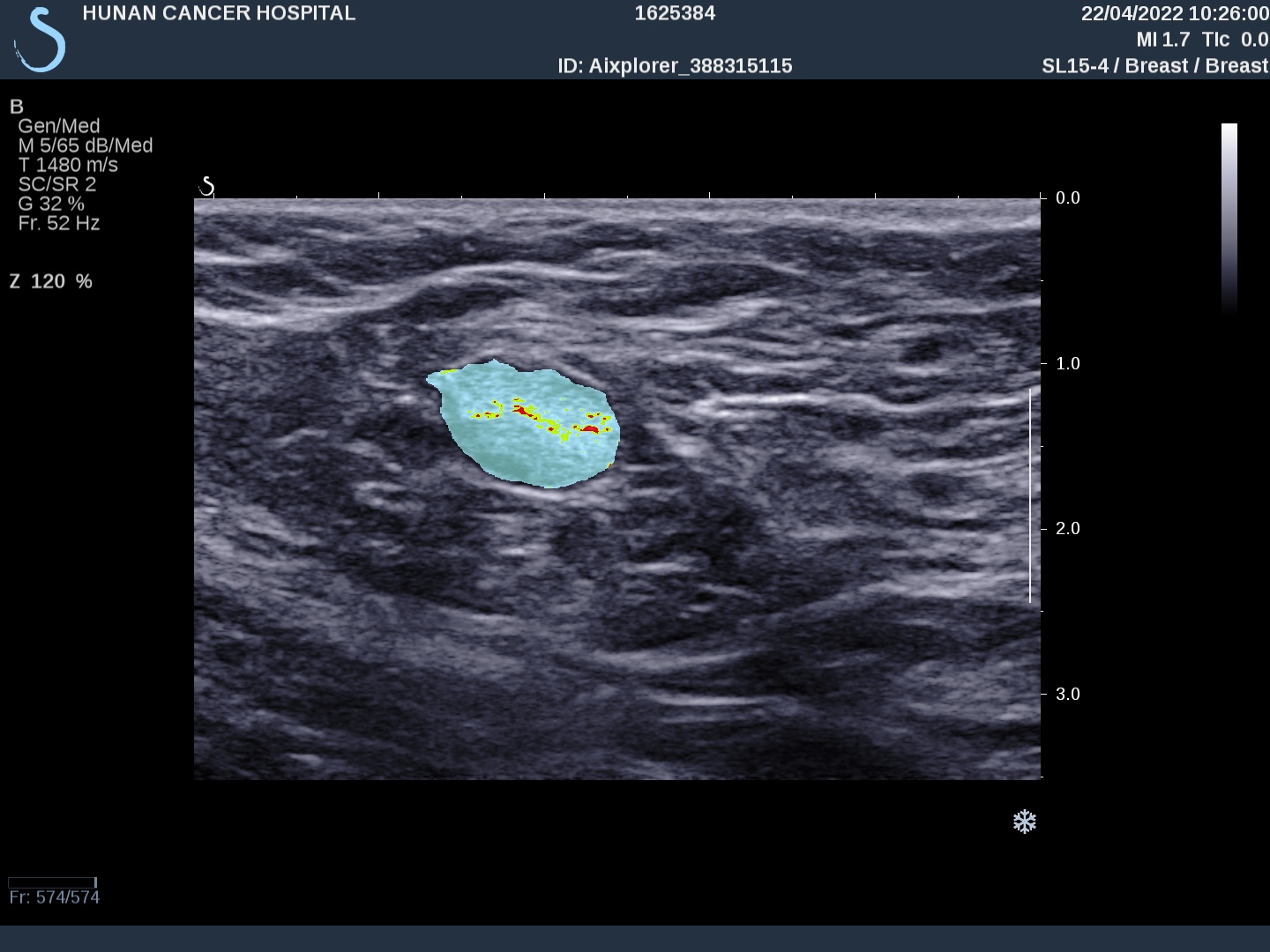

Supplement: Supplementary file 2 [file DataSheet_2.zip › ROI/1625384-1.jpg]

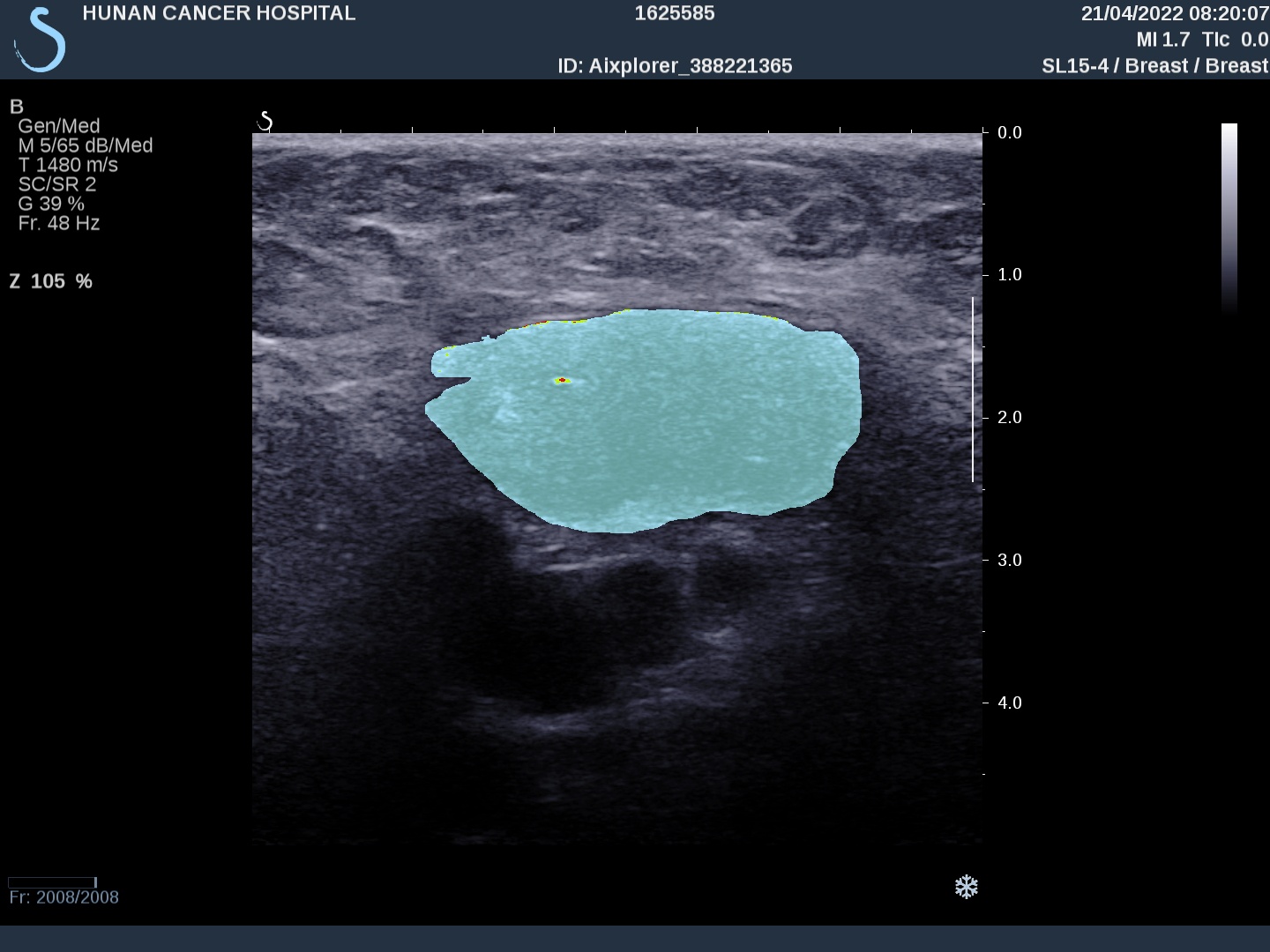

Supplement: Supplementary file 2 [file DataSheet_2.zip › ROI/1625585-2.jpg]

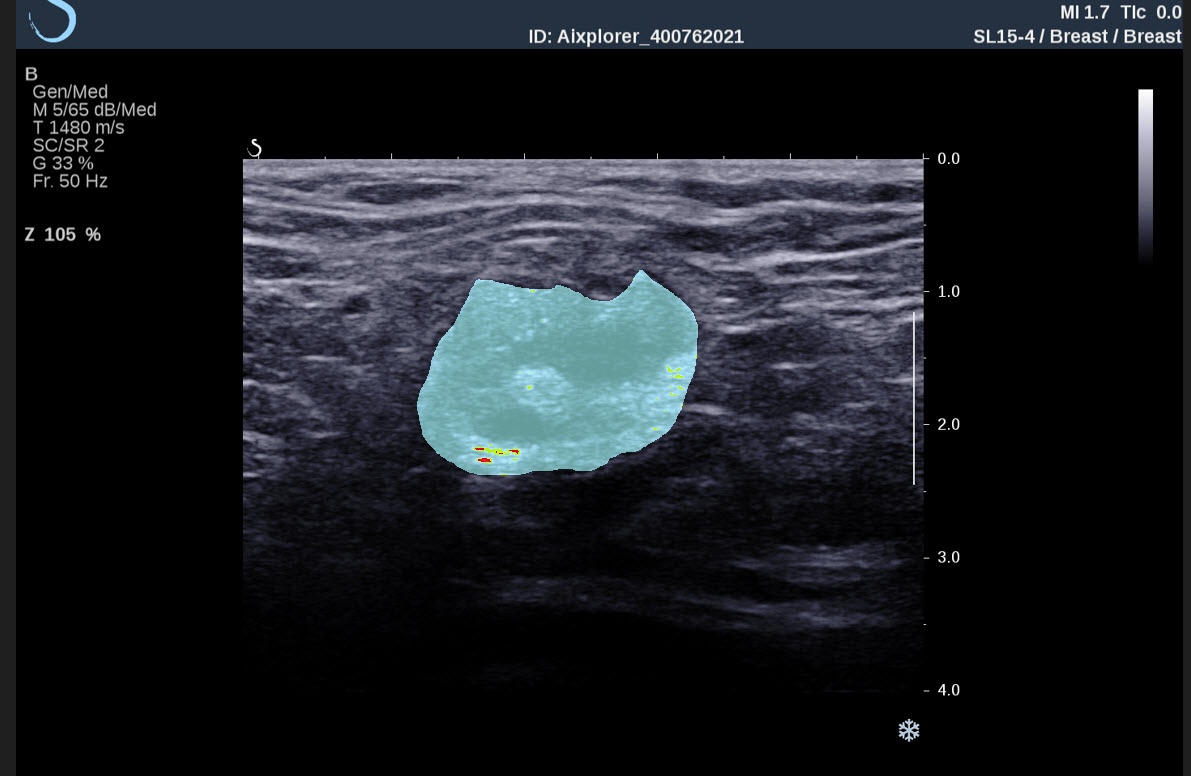

Supplement: Supplementary file 2 [file DataSheet_2.zip › ROI/1625650-1.jpg]

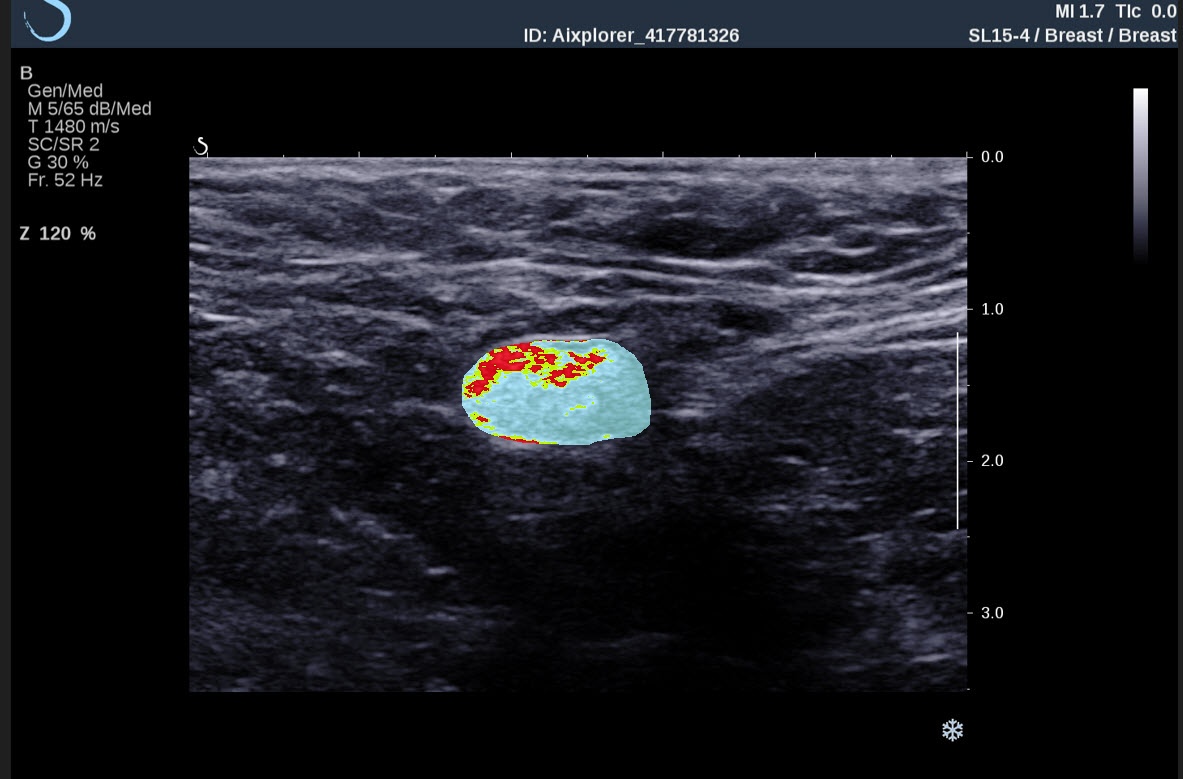

Supplement: Supplementary file 2 [file DataSheet_2.zip › ROI/1642336-1.jpg]

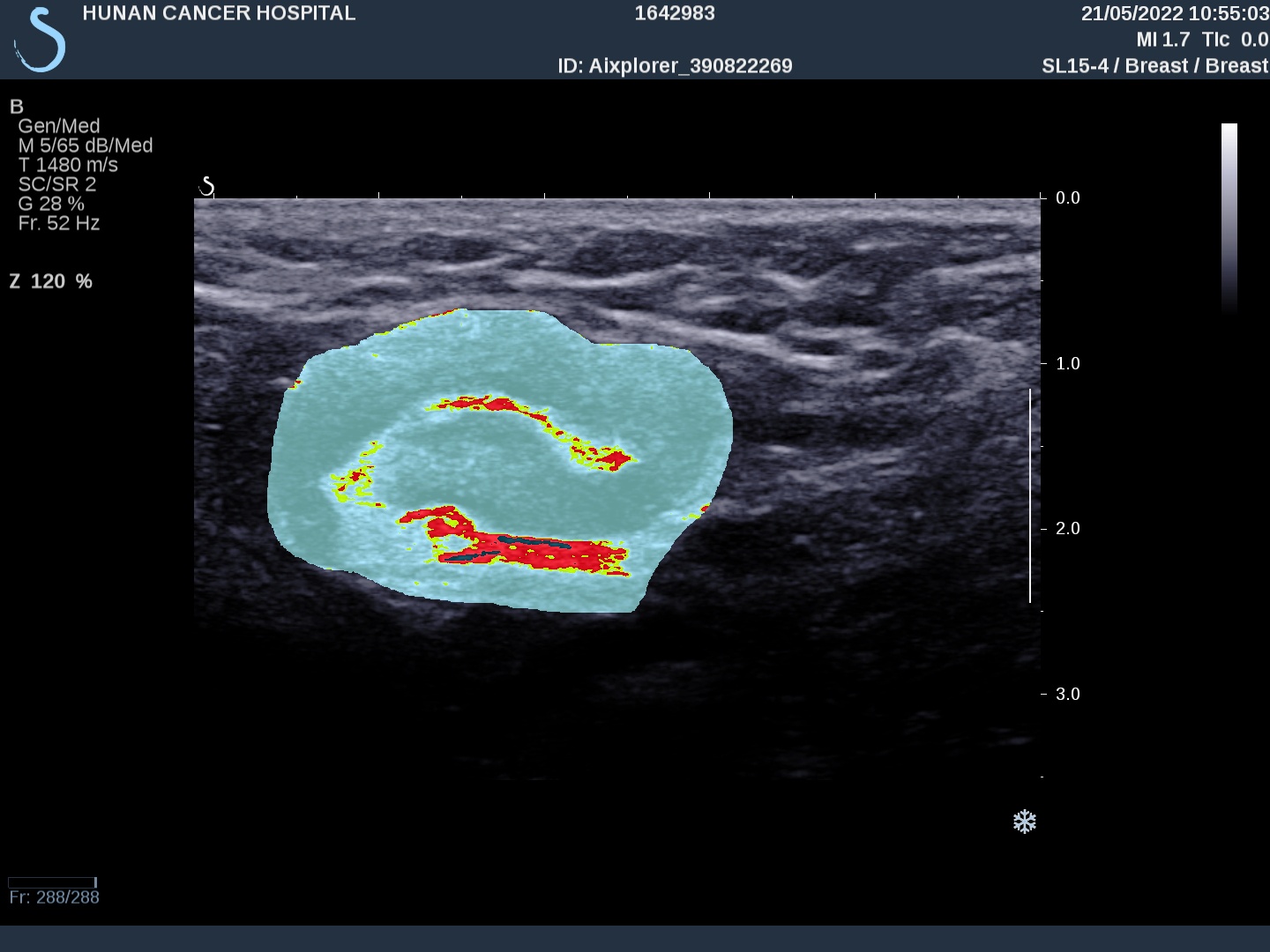

Supplement: Supplementary file 2 [file DataSheet_2.zip › ROI/1642983-1.jpg]

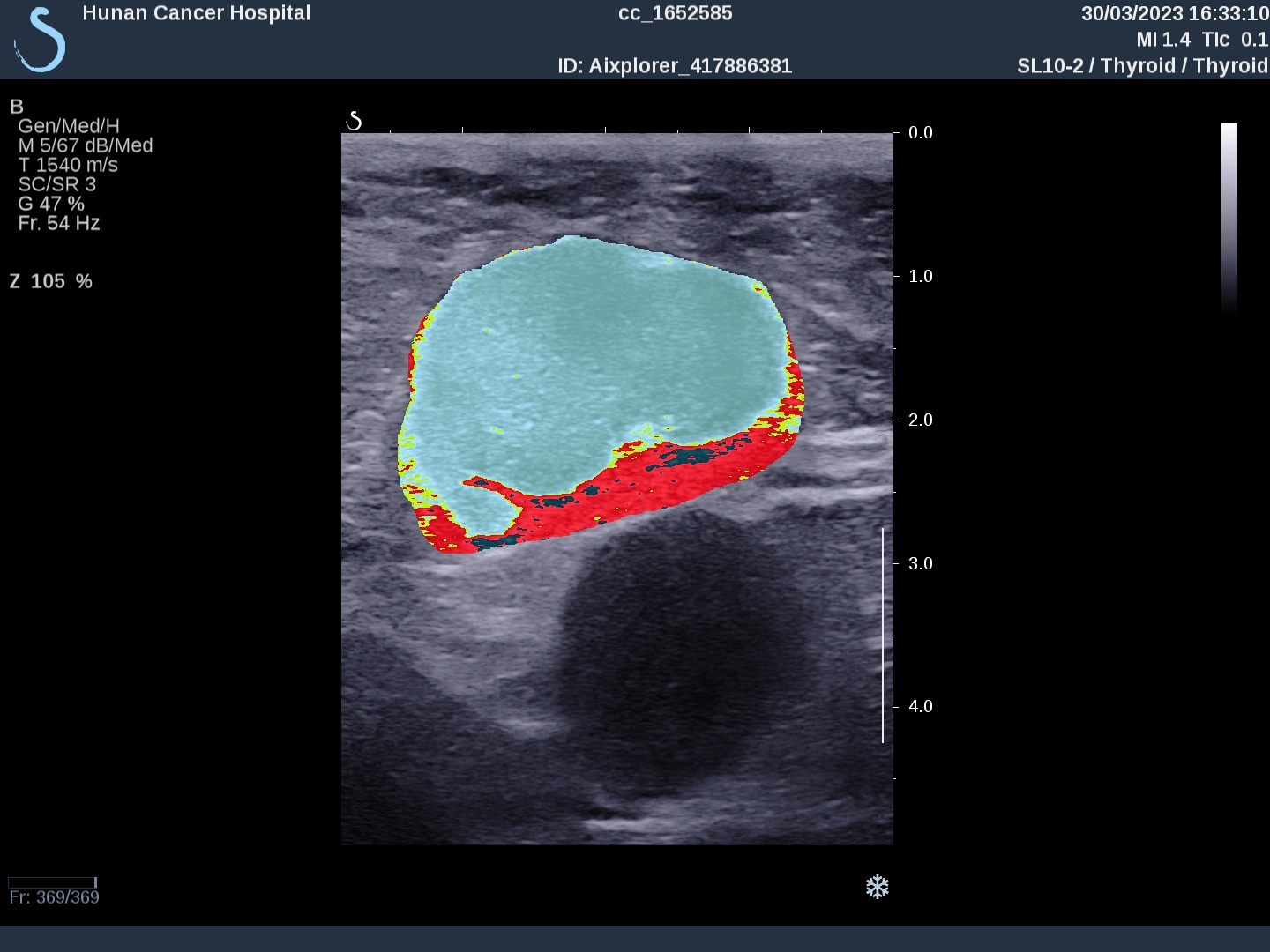

Supplement: Supplementary file 2 [file DataSheet_2.zip › ROI/1652585-1.jpg]

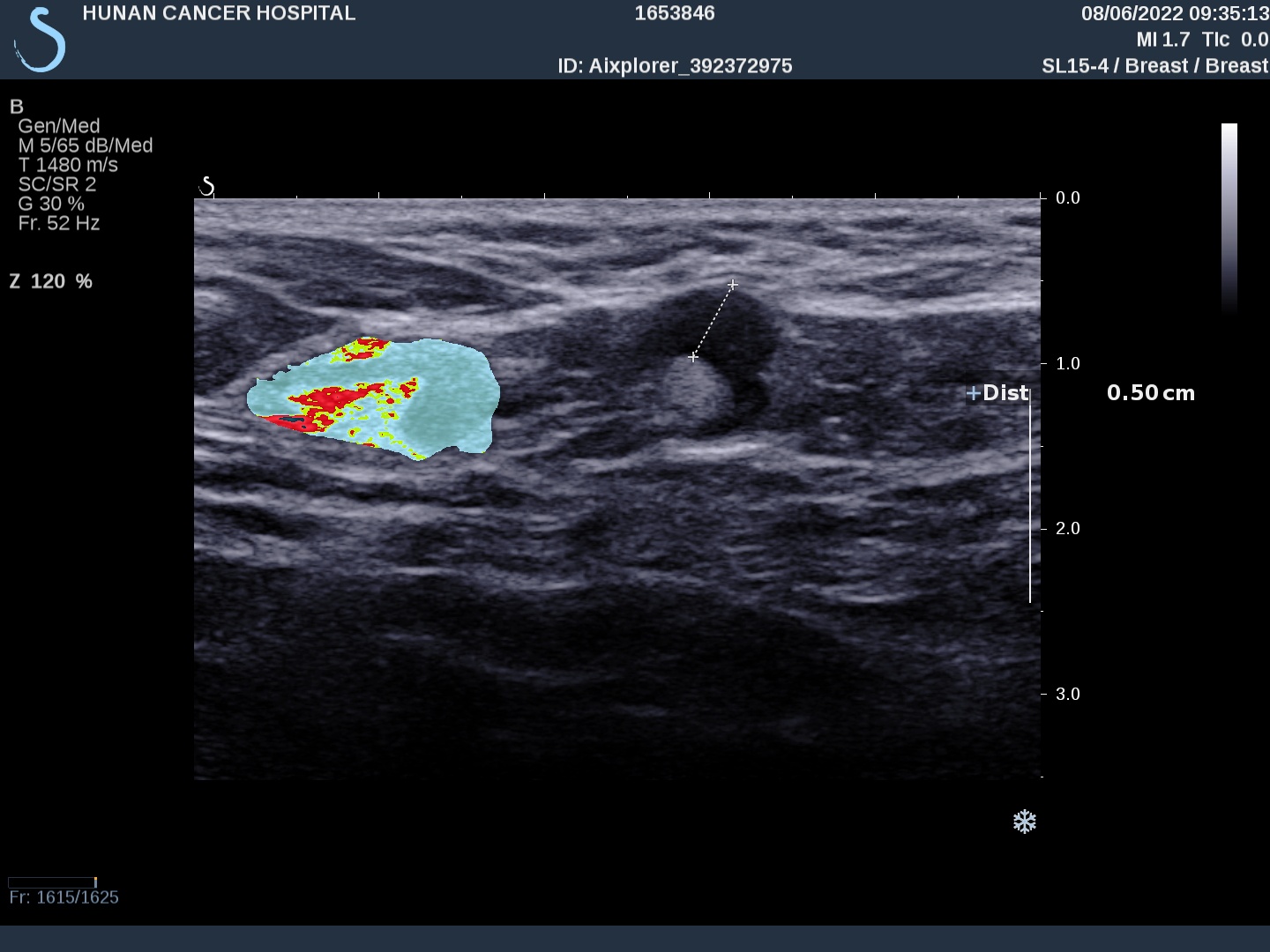

Supplement: Supplementary file 2 [file DataSheet_2.zip › ROI/1653846-2.jpg]

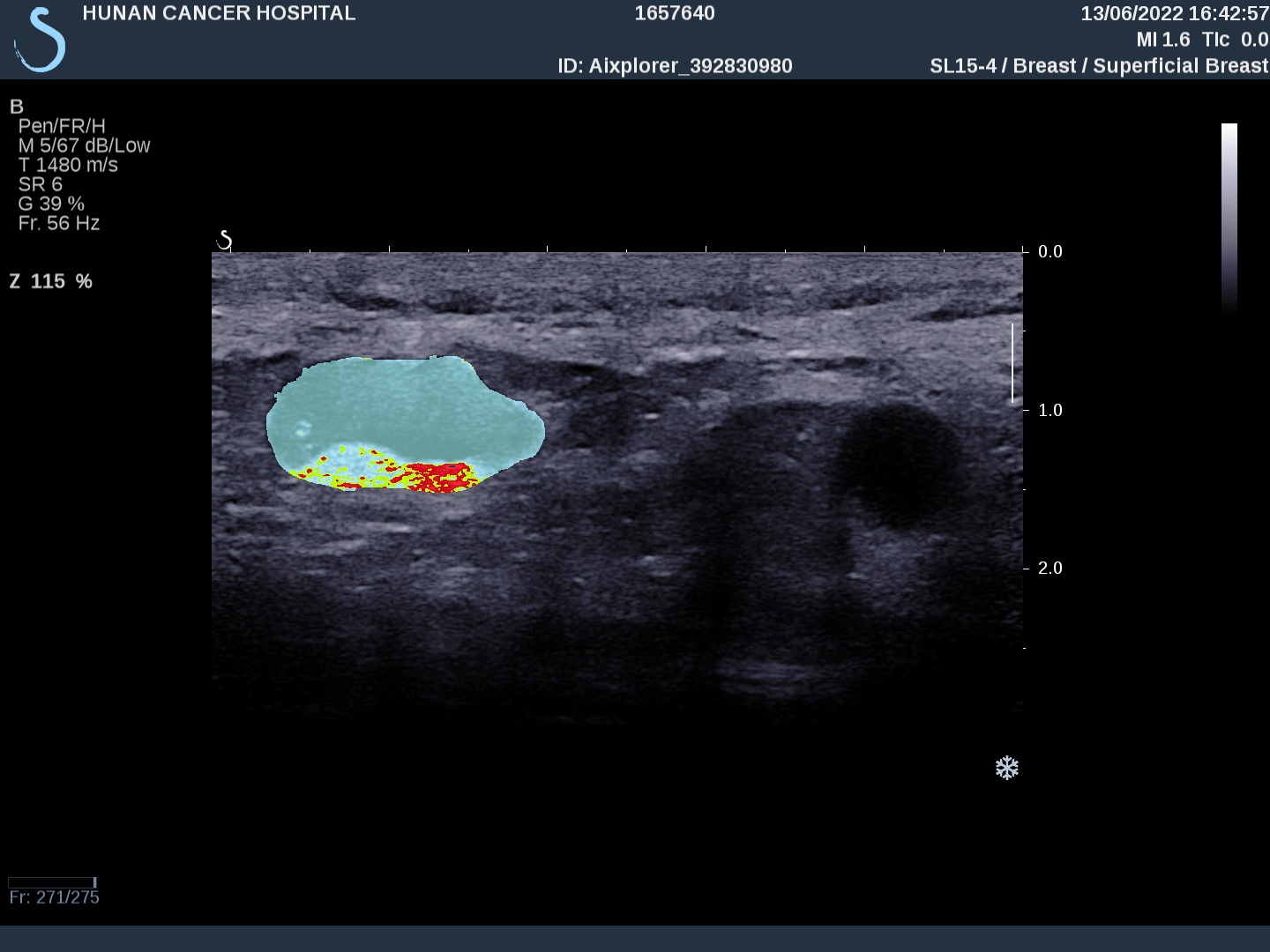

Supplement: Supplementary file 2 [file DataSheet_2.zip › ROI/1657690-1.jpg]

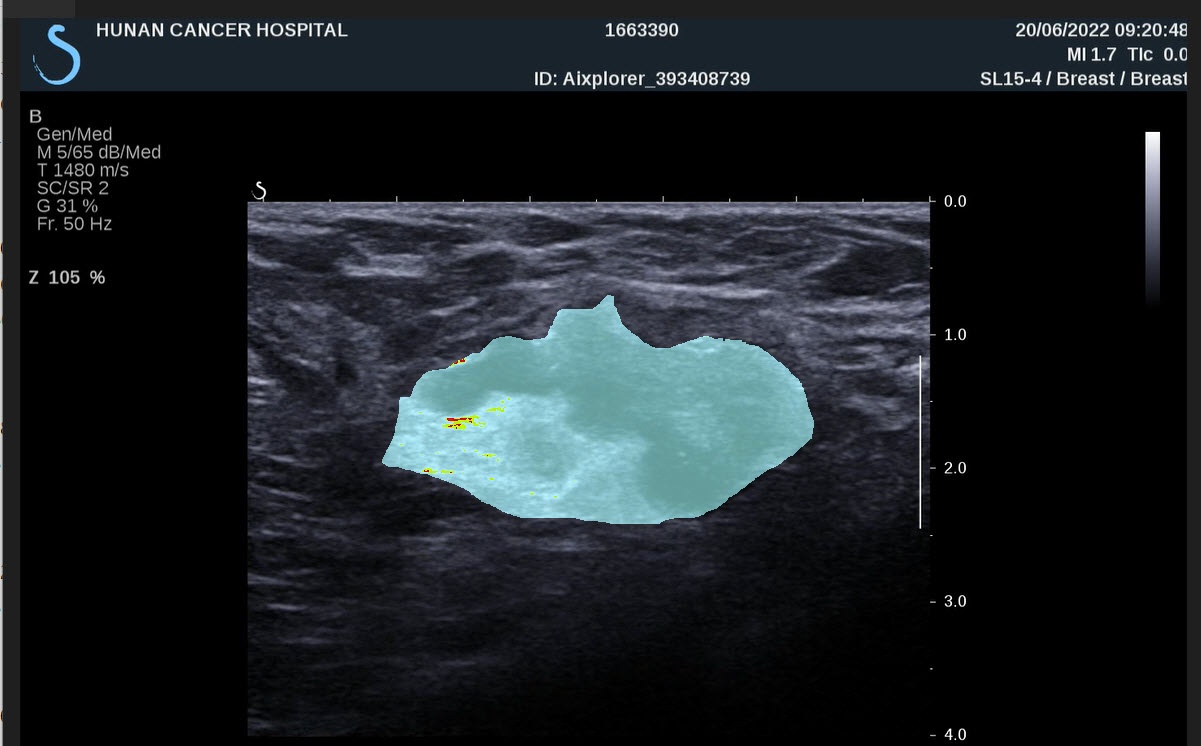

Supplement: Supplementary file 2 [file DataSheet_2.zip › ROI/1663390-1.jpg]

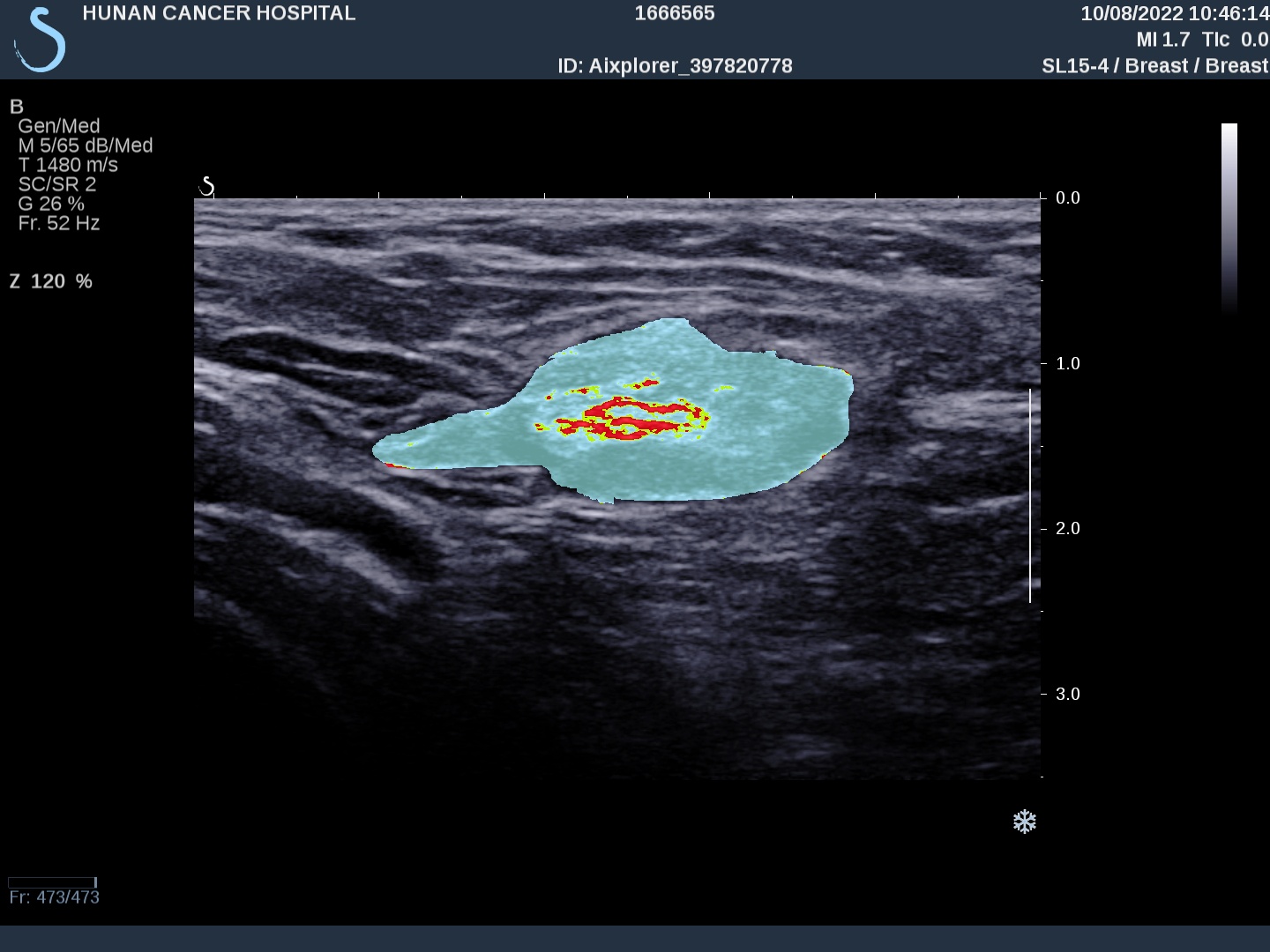

Supplement: Supplementary file 2 [file DataSheet_2.zip › ROI/1666565-1.jpg]

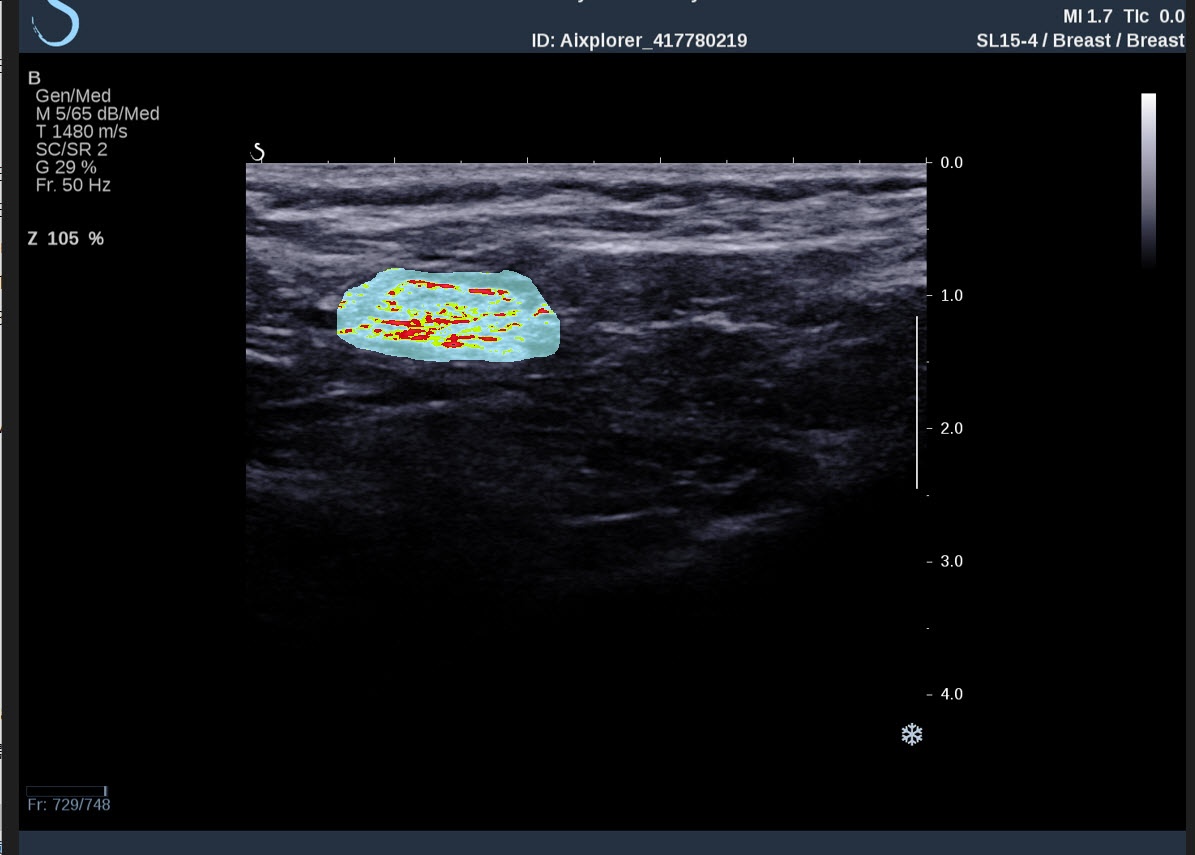

Supplement: Supplementary file 2 [file DataSheet_2.zip › ROI/1667708-1.jpg]

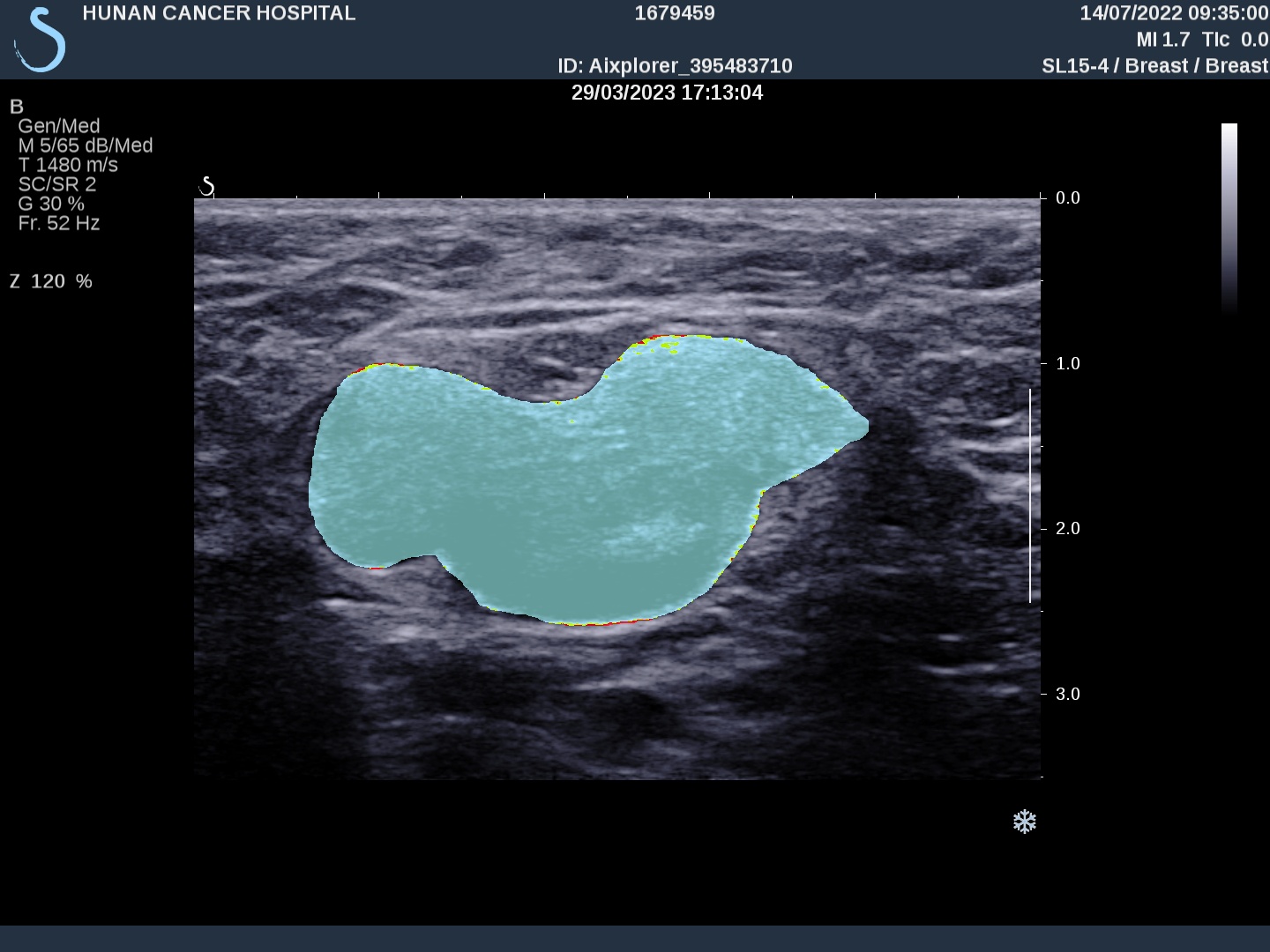

Supplement: Supplementary file 2 [file DataSheet_2.zip › ROI/1679459-1.jpg]

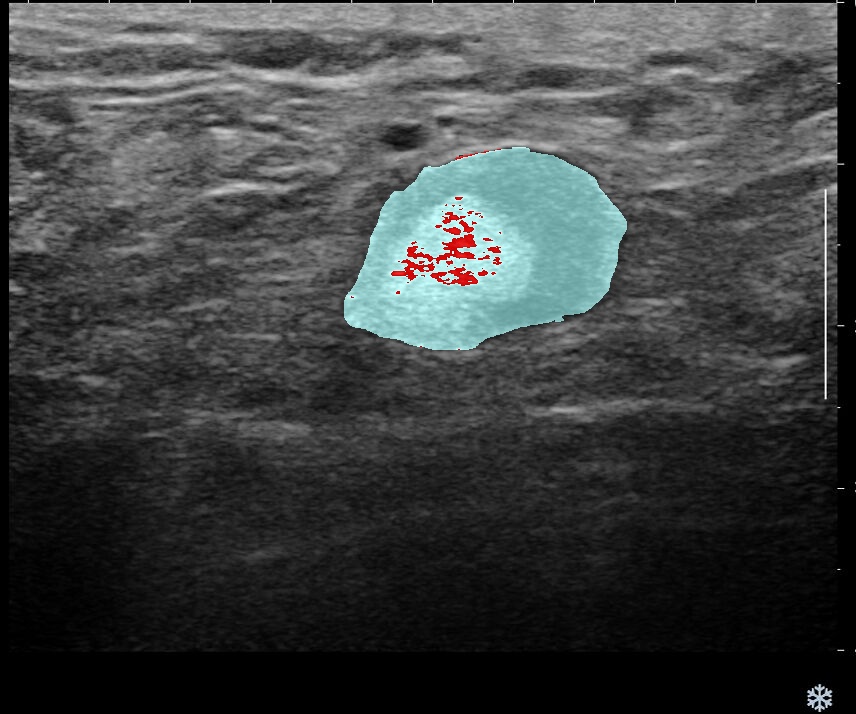

Supplement: Supplementary file 2 [file DataSheet_2.zip › ROI/1682367-1.jpg]

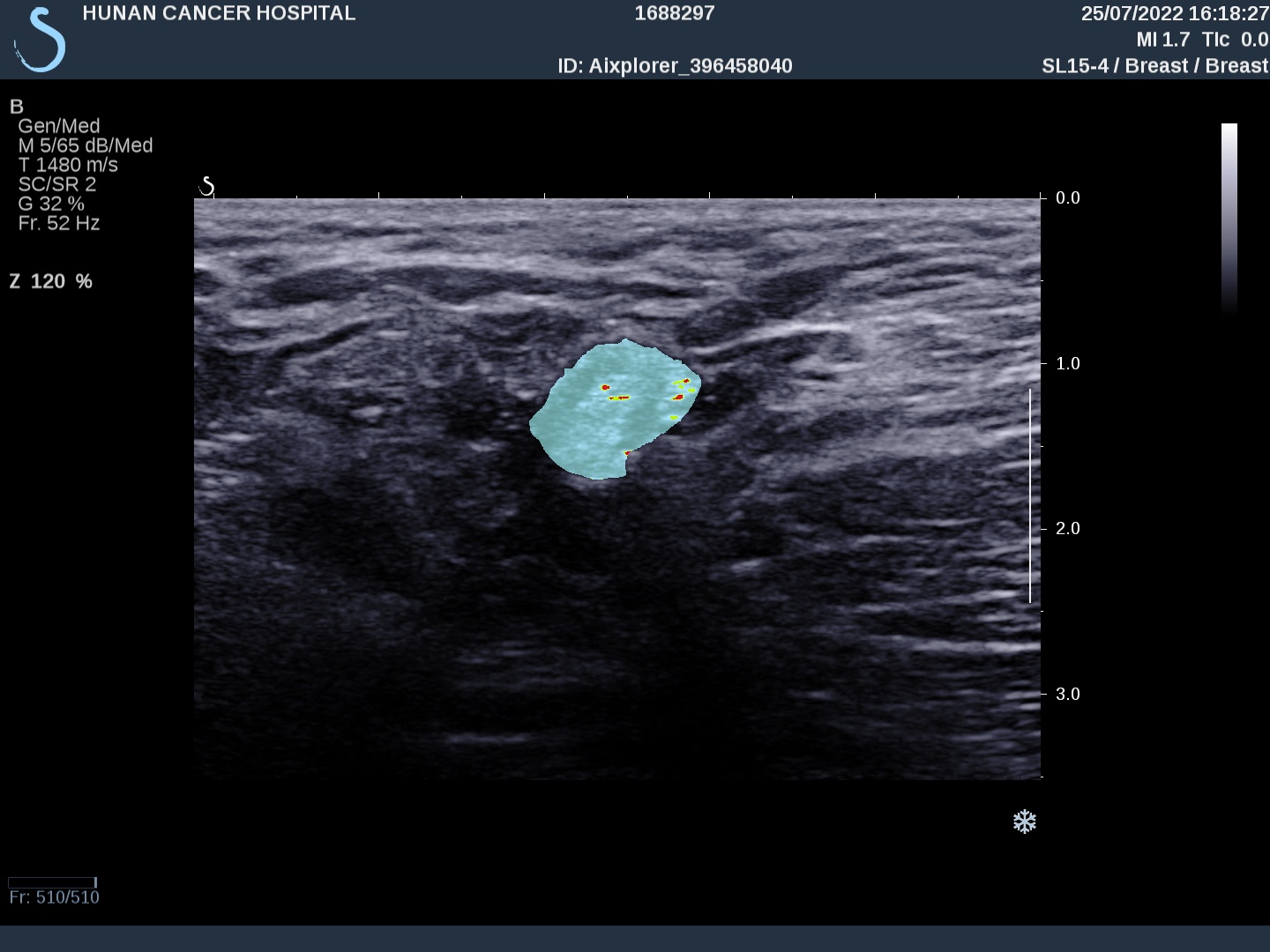

Supplement: Supplementary file 2 [file DataSheet_2.zip › ROI/1688297-1.jpg]

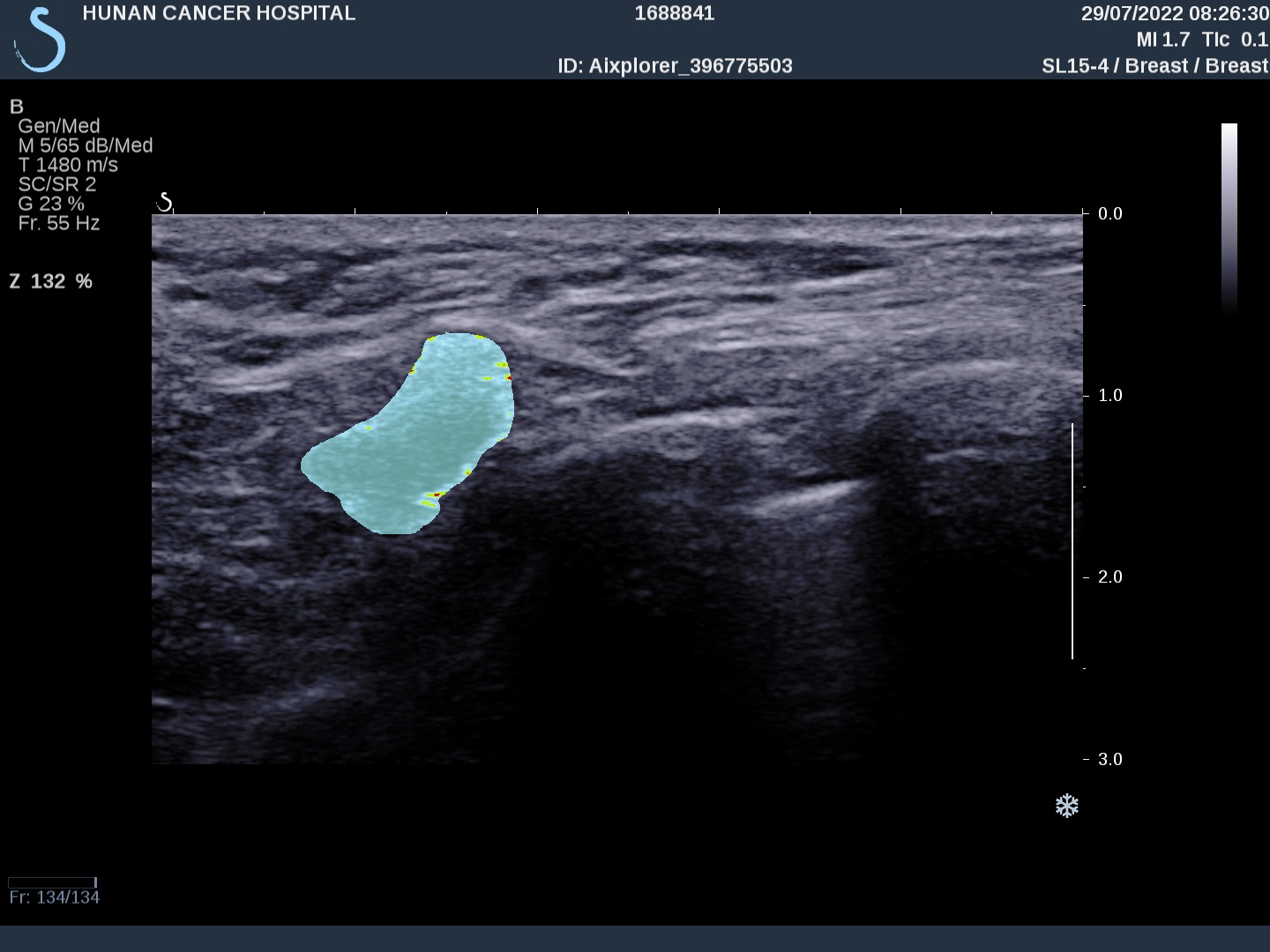

Supplement: Supplementary file 2 [file DataSheet_2.zip › ROI/1688841-1.jpg]

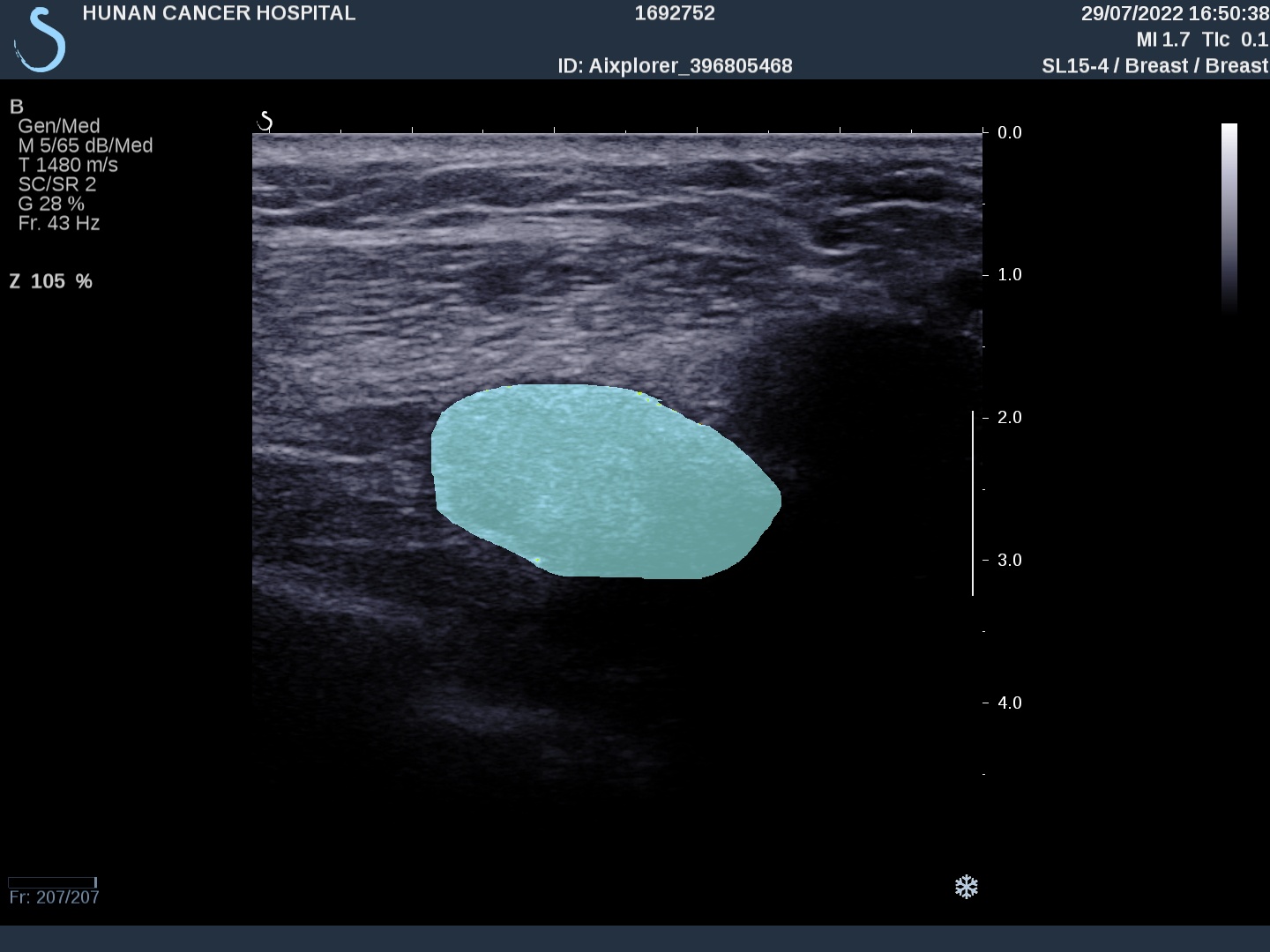

Supplement: Supplementary file 2 [file DataSheet_2.zip › ROI/1692752-1.jpg]

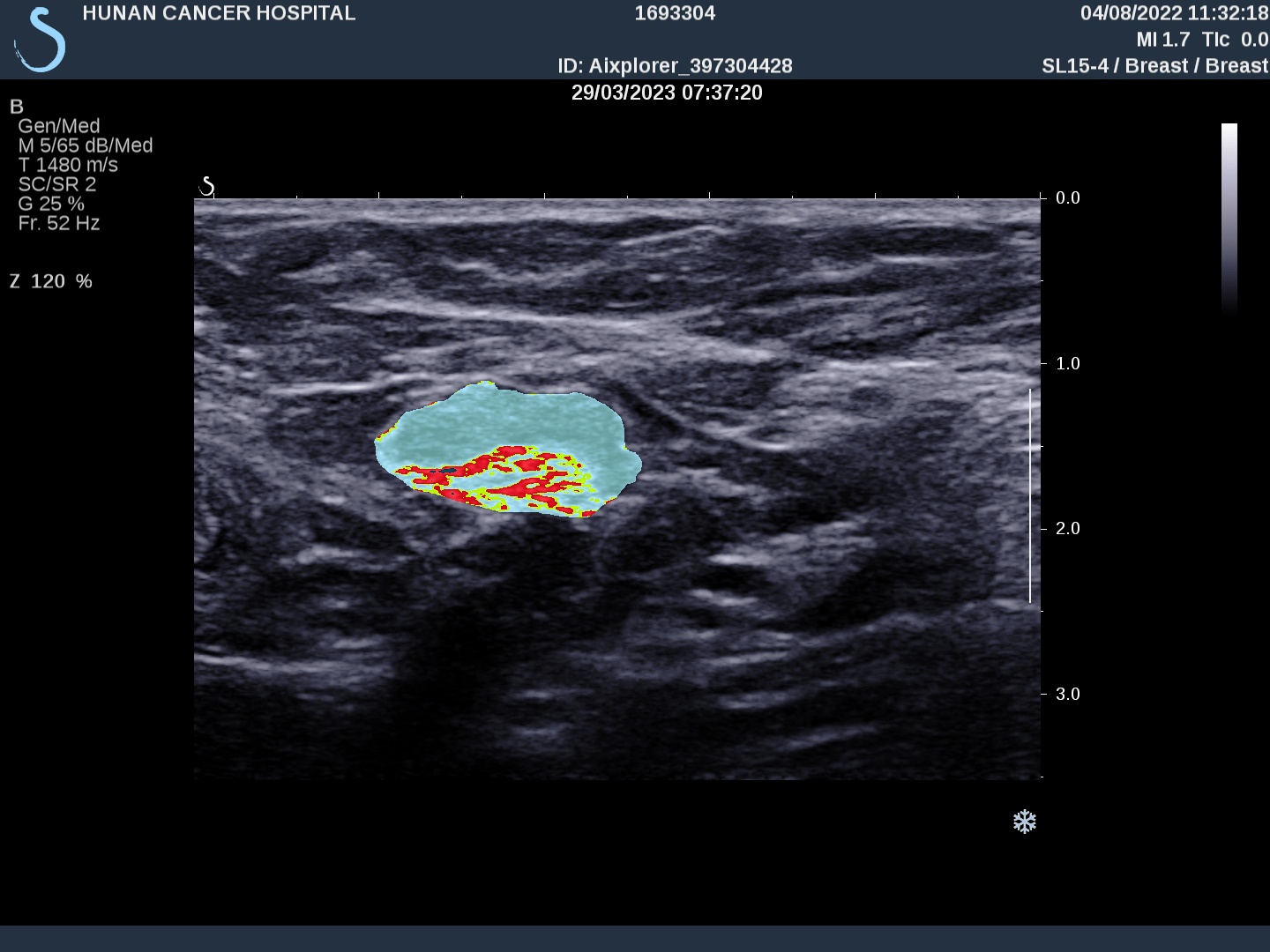

Supplement: Supplementary file 2 [file DataSheet_2.zip › ROI/1693304-1.jpg]

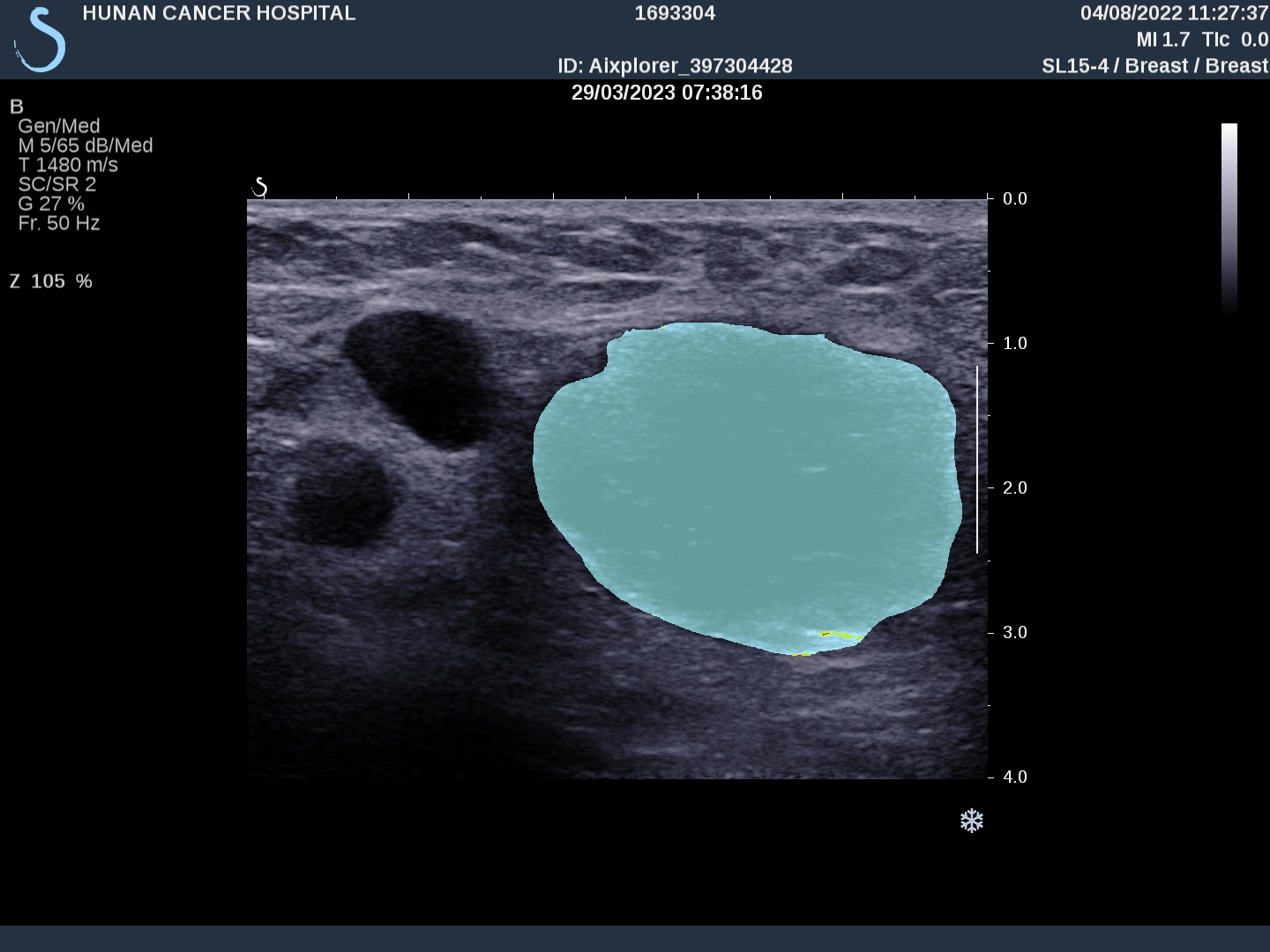

Supplement: Supplementary file 2 [file DataSheet_2.zip › ROI/1693304-2.jpg]

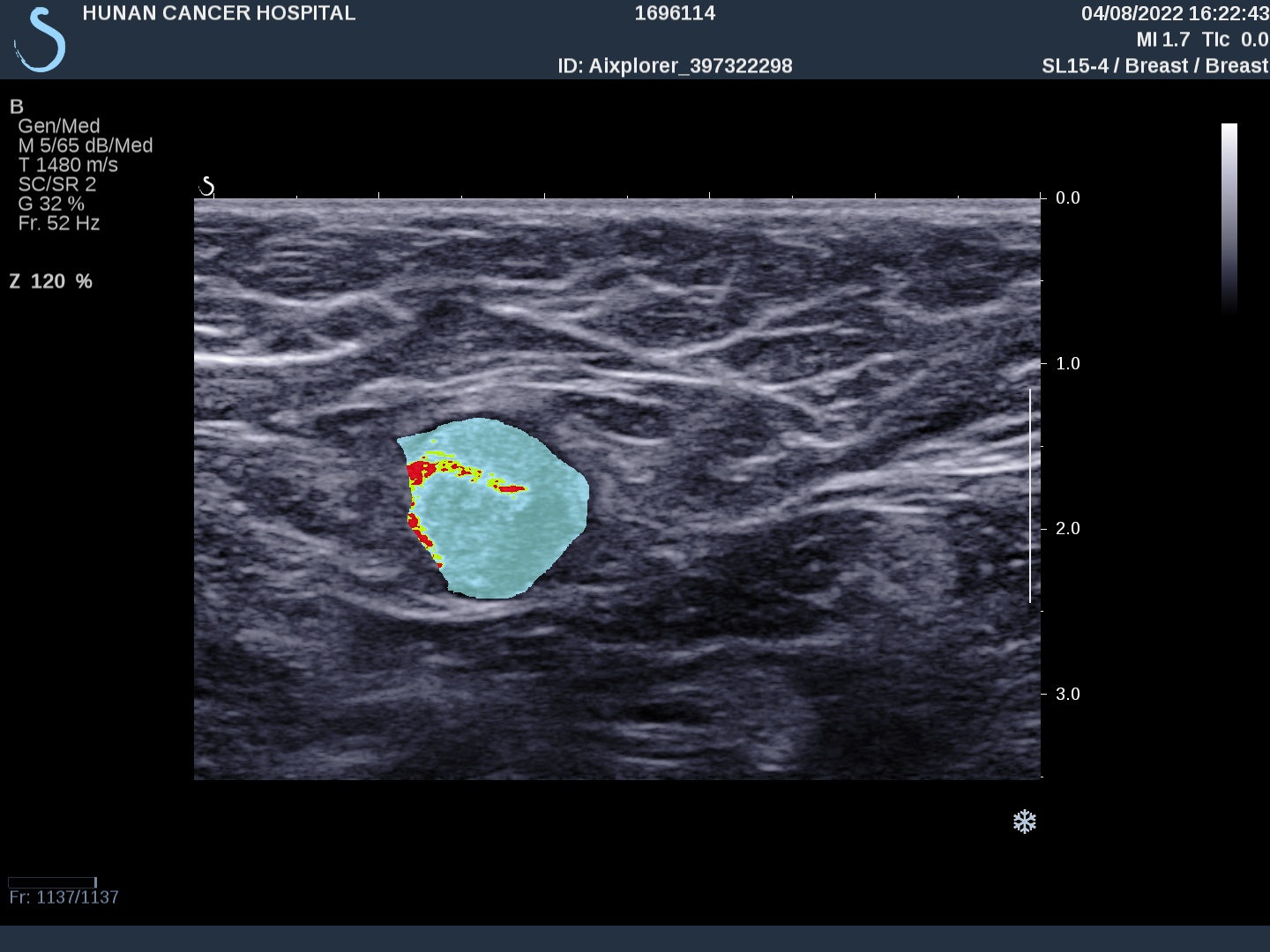

Supplement: Supplementary file 2 [file DataSheet_2.zip › ROI/1696114-1.jpg]

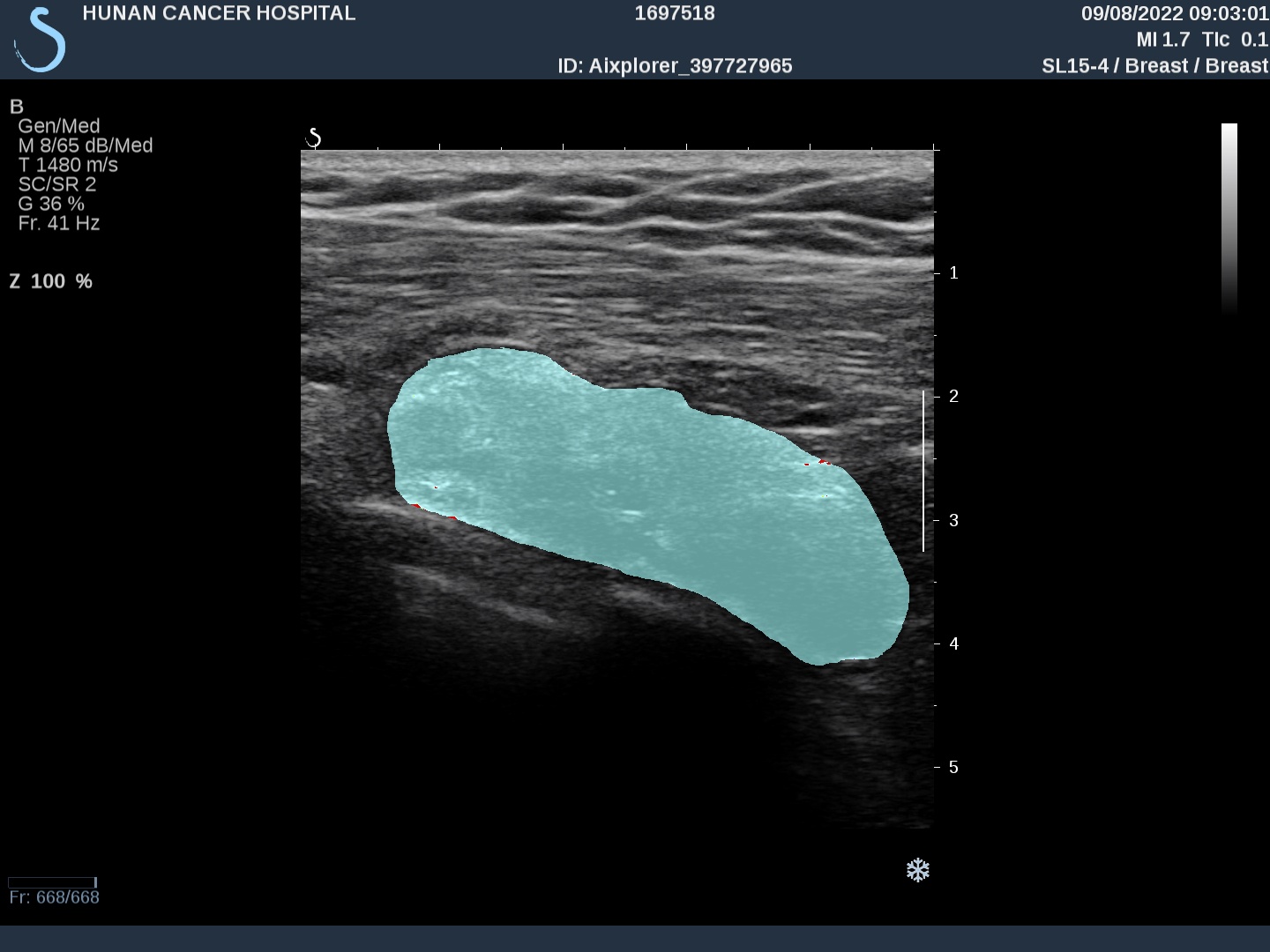

Supplement: Supplementary file 2 [file DataSheet_2.zip › ROI/1697518-1.jpg]

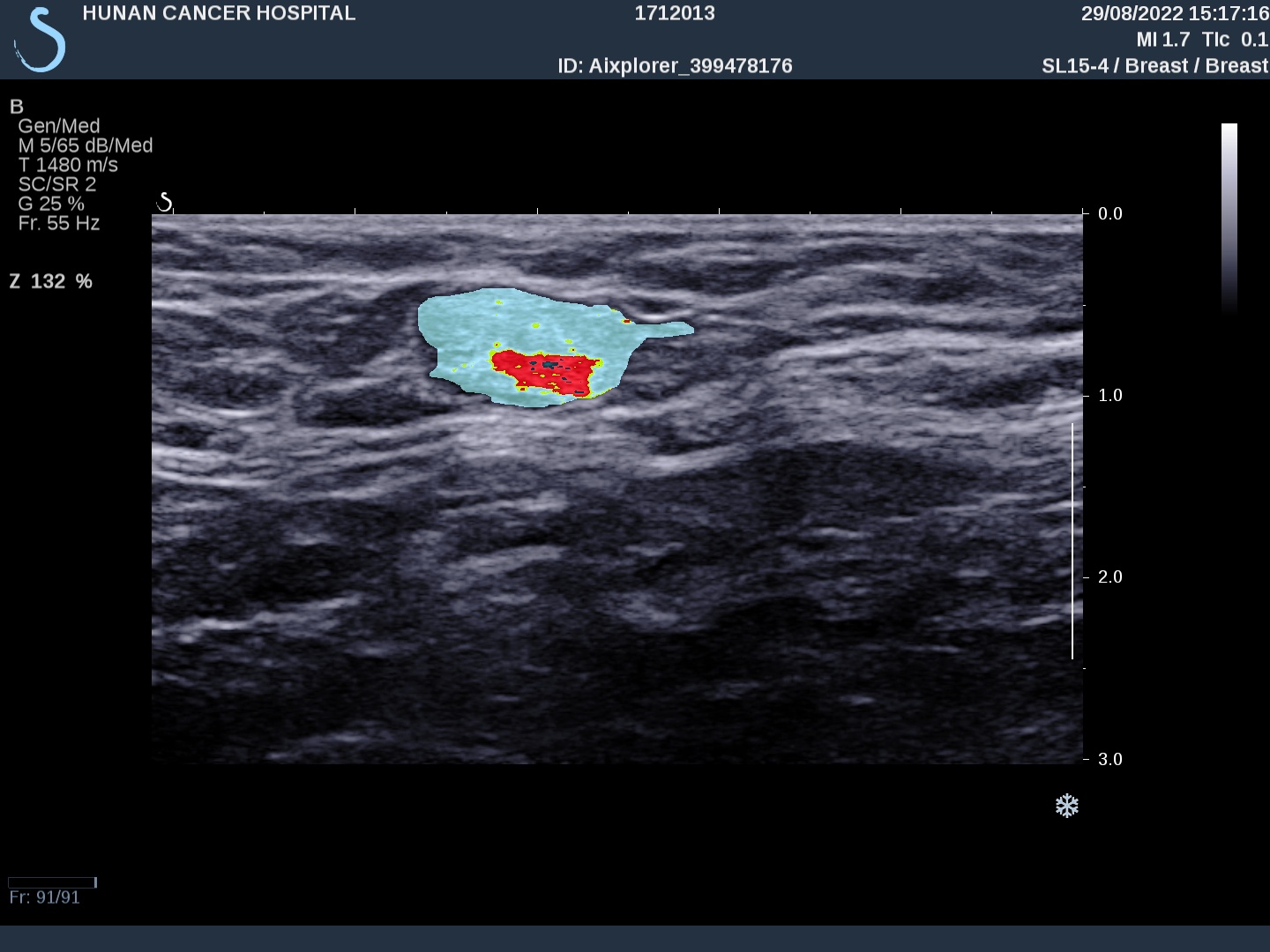

Supplement: Supplementary file 2 [file DataSheet_2.zip › ROI/1712013-1.jpg]

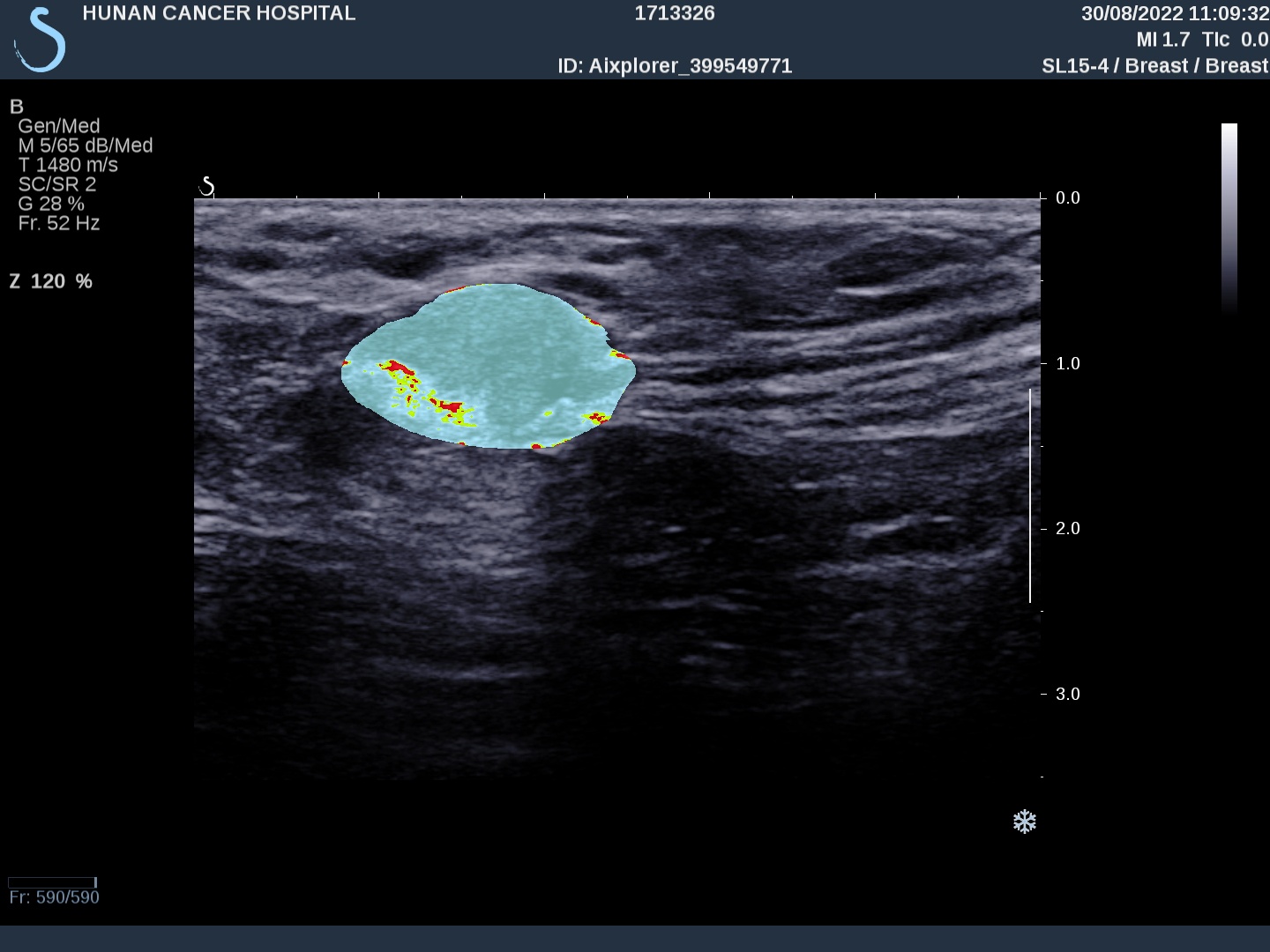

Supplement: Supplementary file 2 [file DataSheet_2.zip › ROI/1713326-1.jpg]

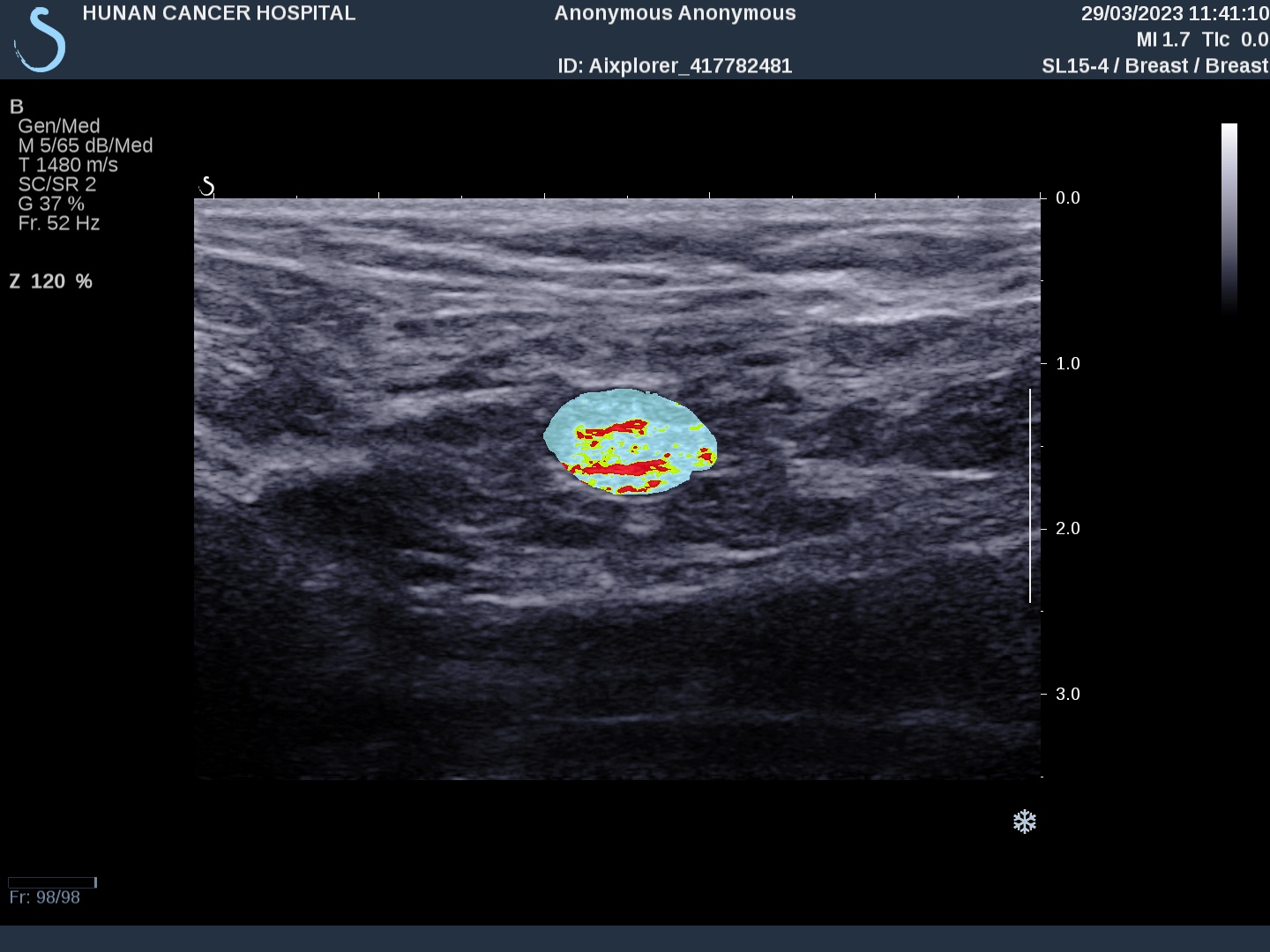

Supplement: Supplementary file 2 [file DataSheet_2.zip › ROI/1714490-1.jpg]

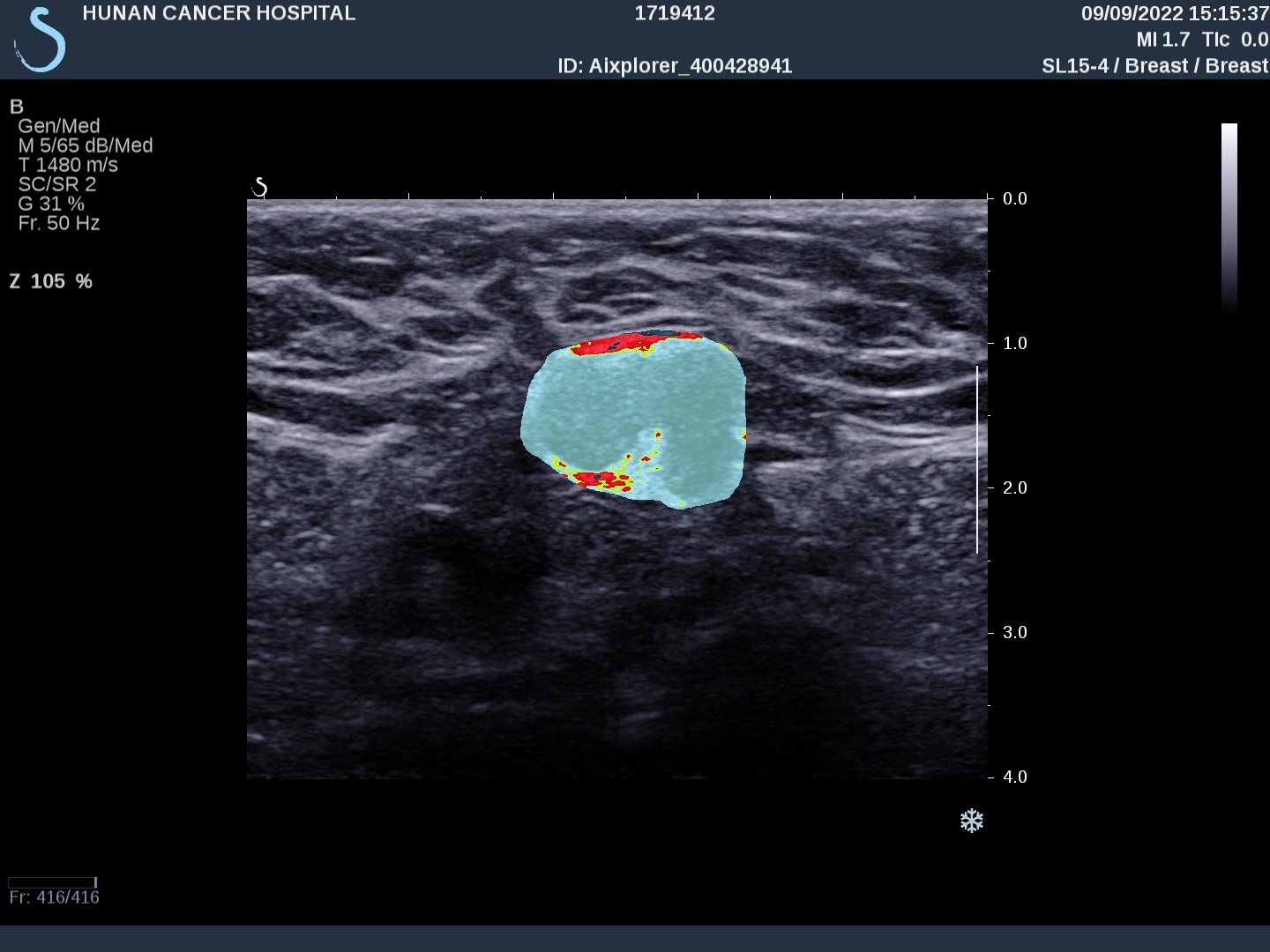

Supplement: Supplementary file 2 [file DataSheet_2.zip › ROI/1719412-1.jpg]

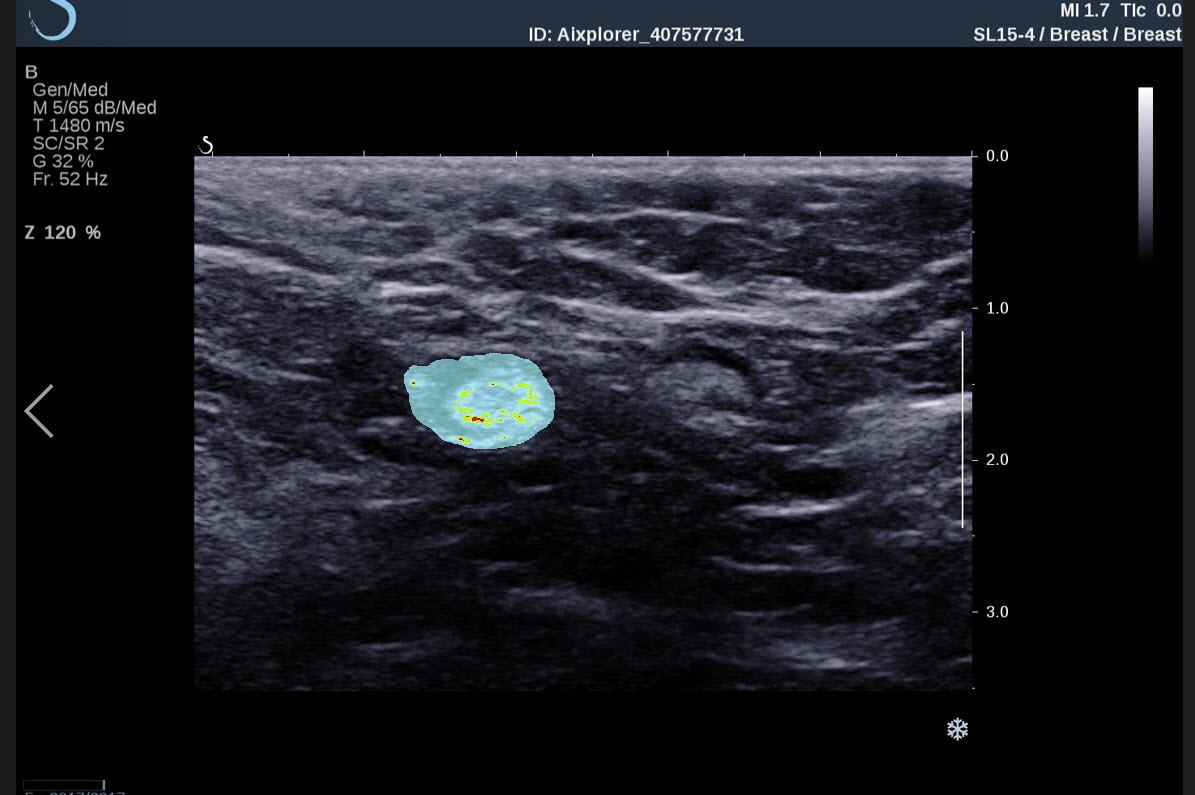

Supplement: Supplementary file 2 [file DataSheet_2.zip › ROI/1724161-1.jpg]

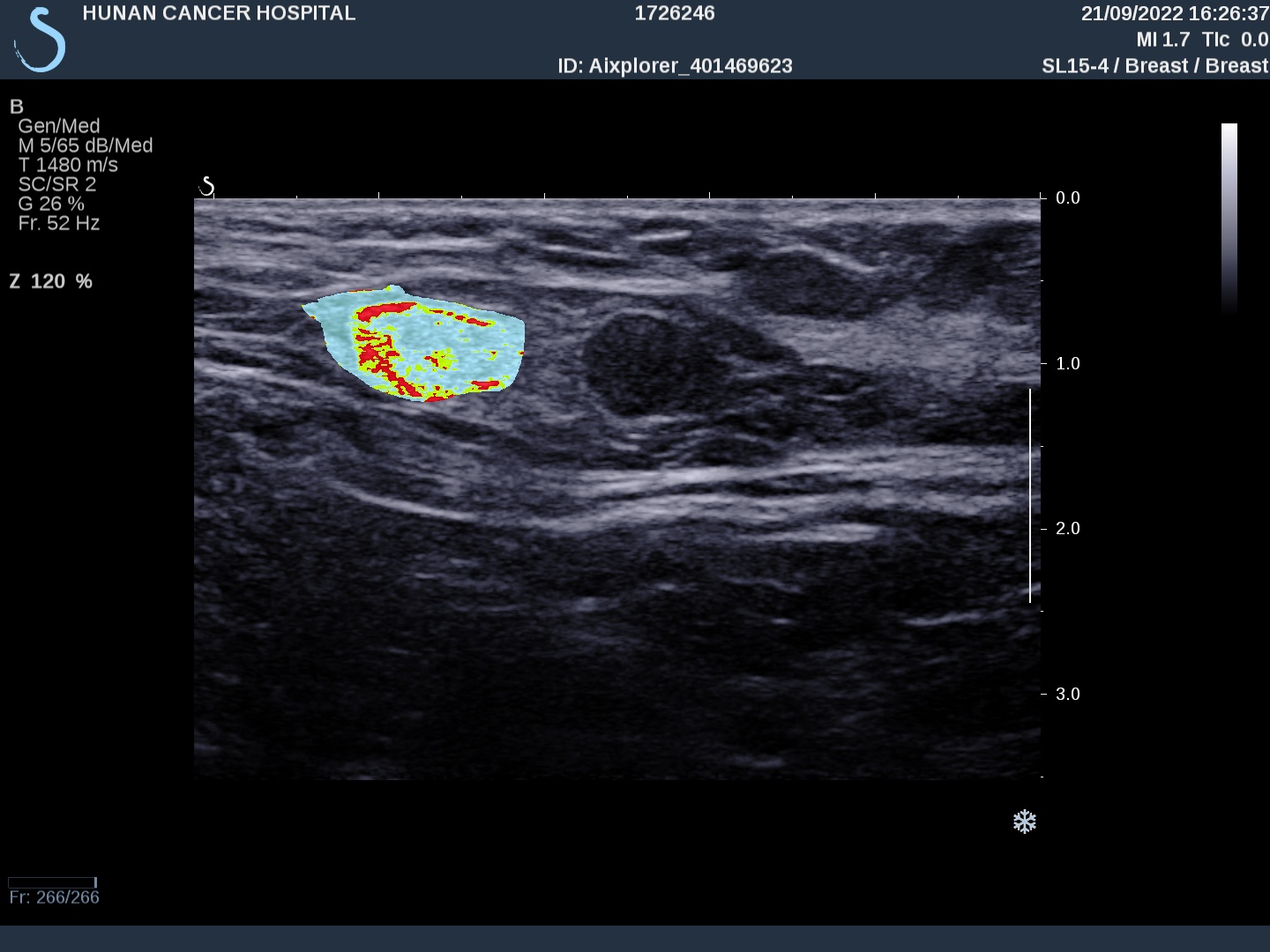

Supplement: Supplementary file 2 [file DataSheet_2.zip › ROI/1726246-1.jpg]

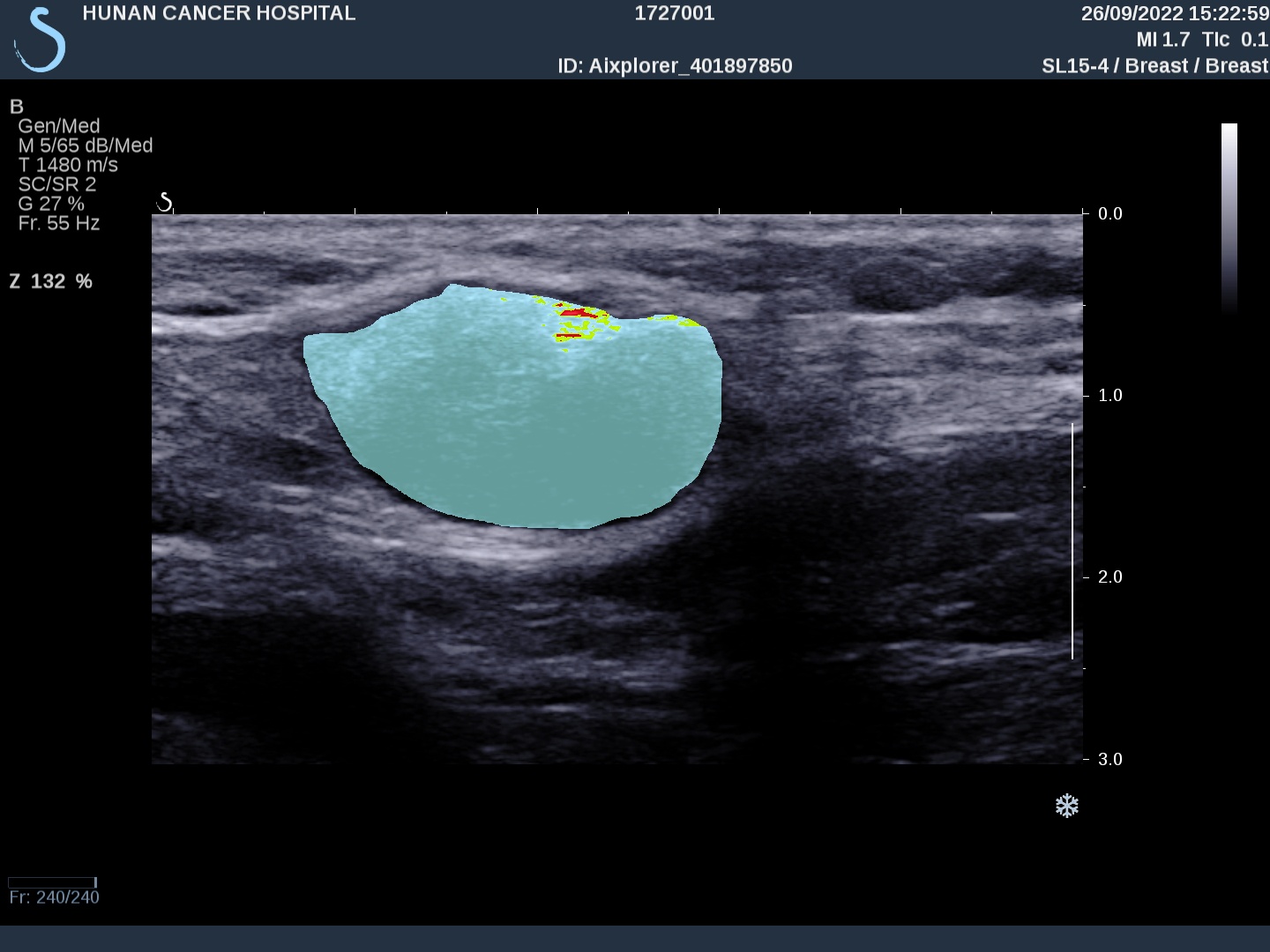

Supplement: Supplementary file 2 [file DataSheet_2.zip › ROI/1727001-2.jpg]

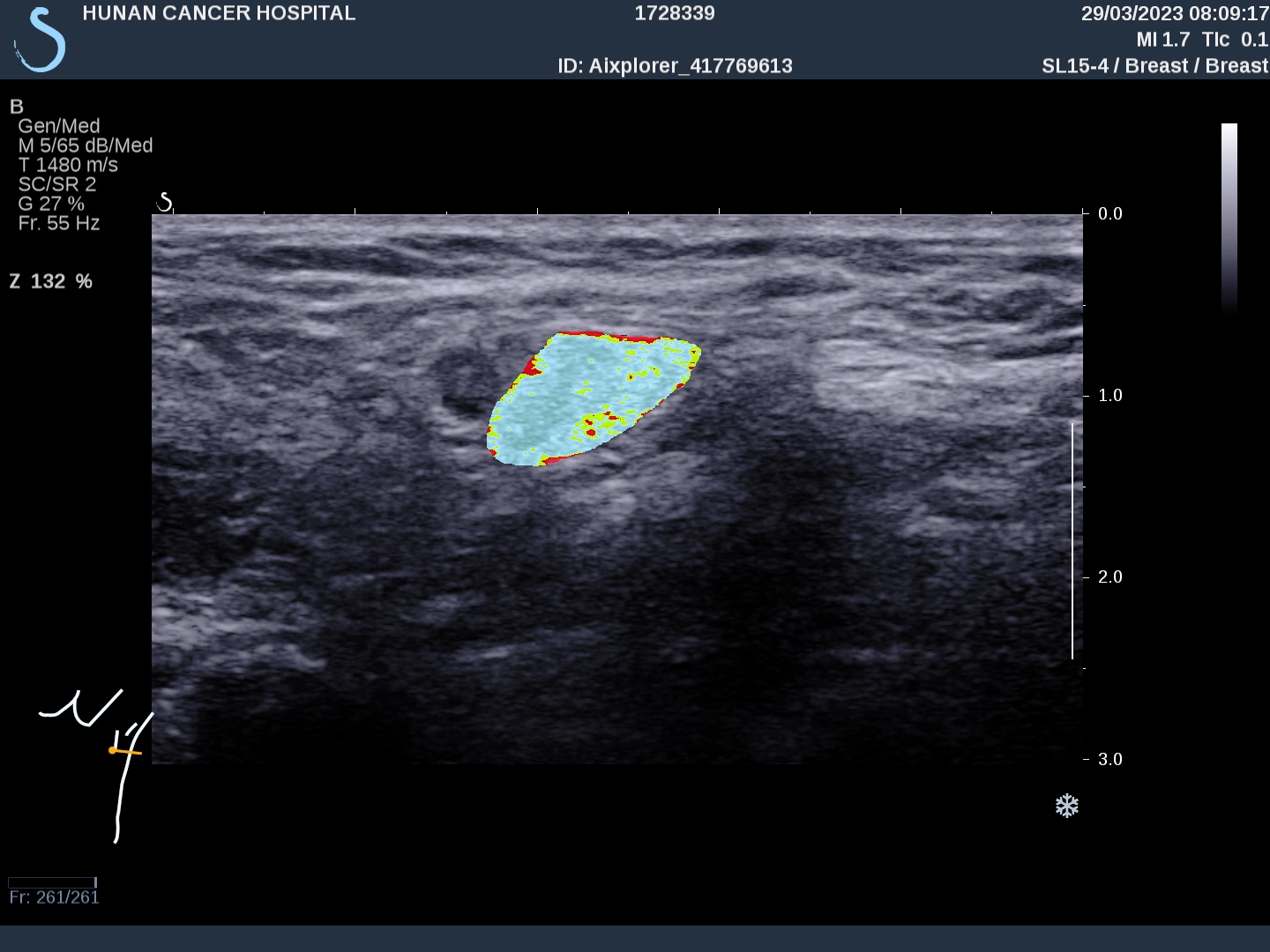

Supplement: Supplementary file 2 [file DataSheet_2.zip › ROI/1728339-1.jpg]

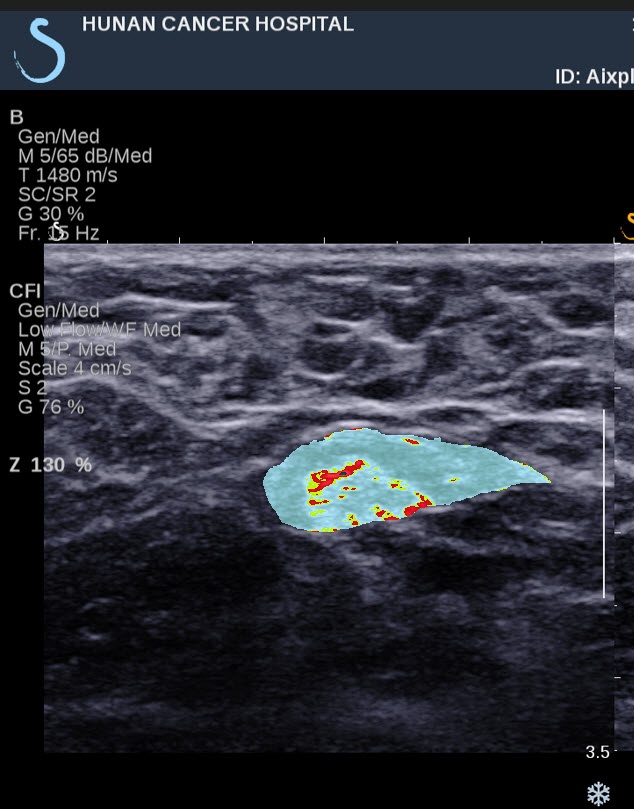

Supplement: Supplementary file 2 [file DataSheet_2.zip › ROI/1731822-1.jpg]

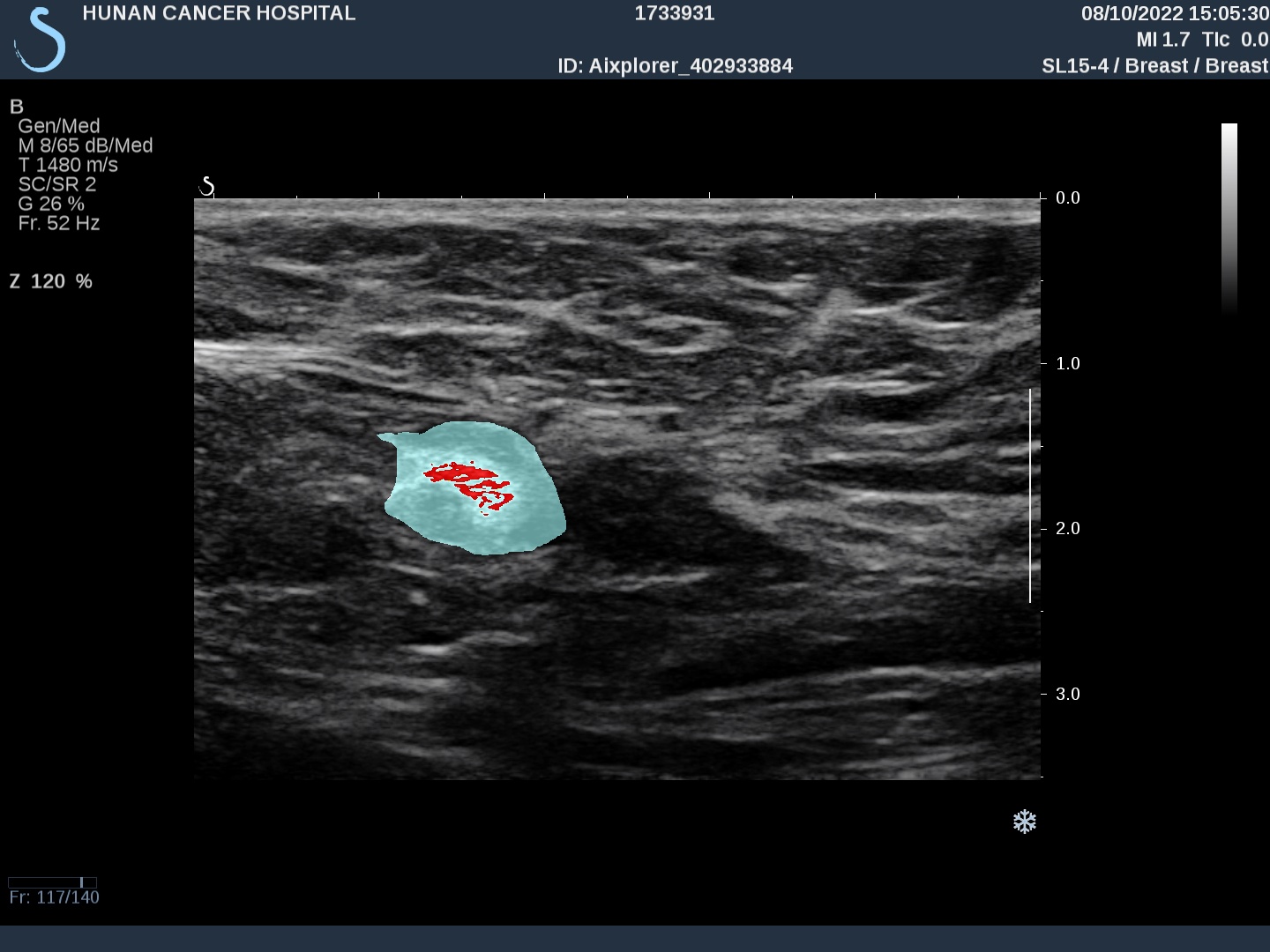

Supplement: Supplementary file 2 [file DataSheet_2.zip › ROI/1733931-2.jpg]

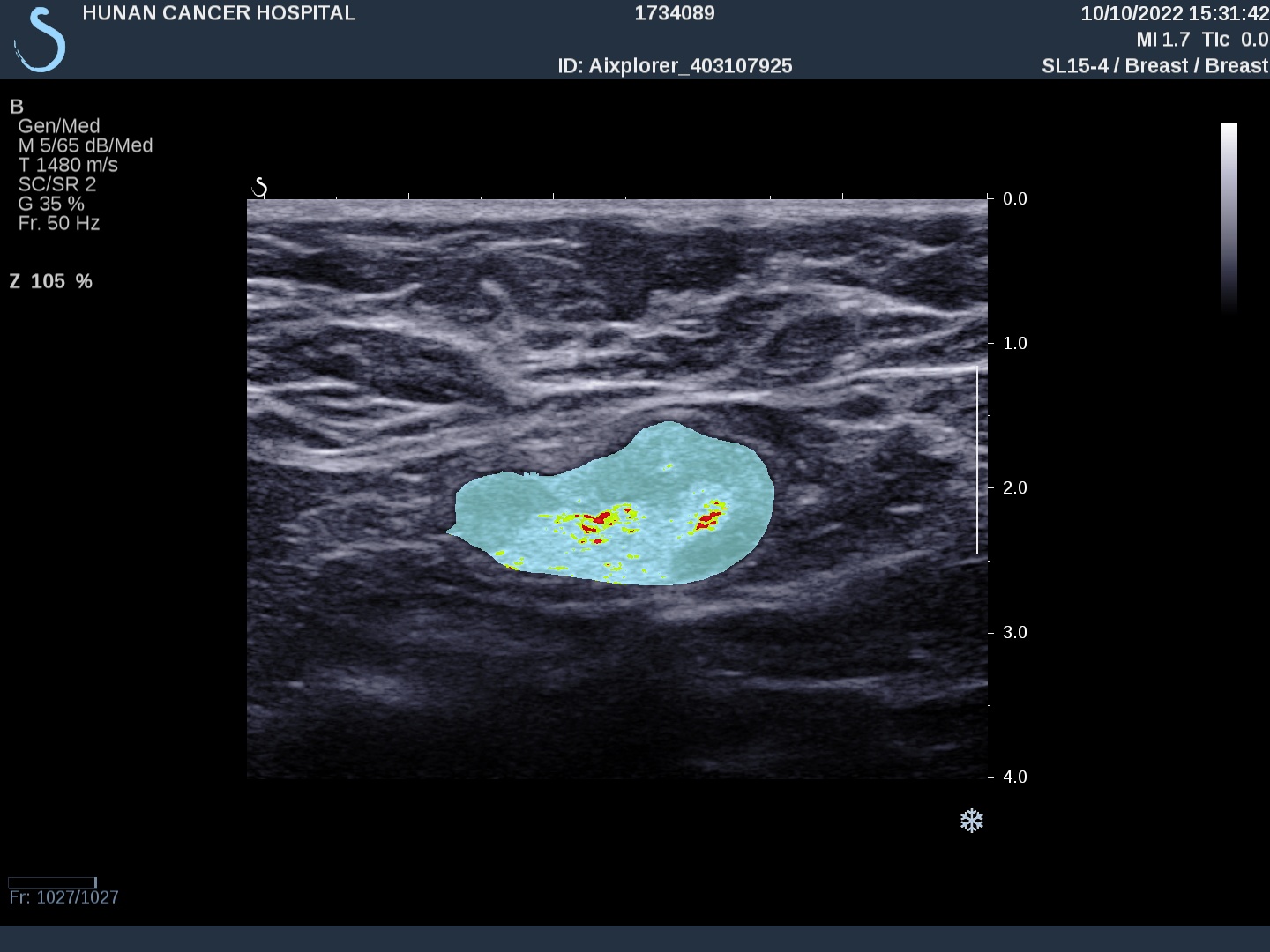

Supplement: Supplementary file 2 [file DataSheet_2.zip › ROI/1734089-1.jpg]

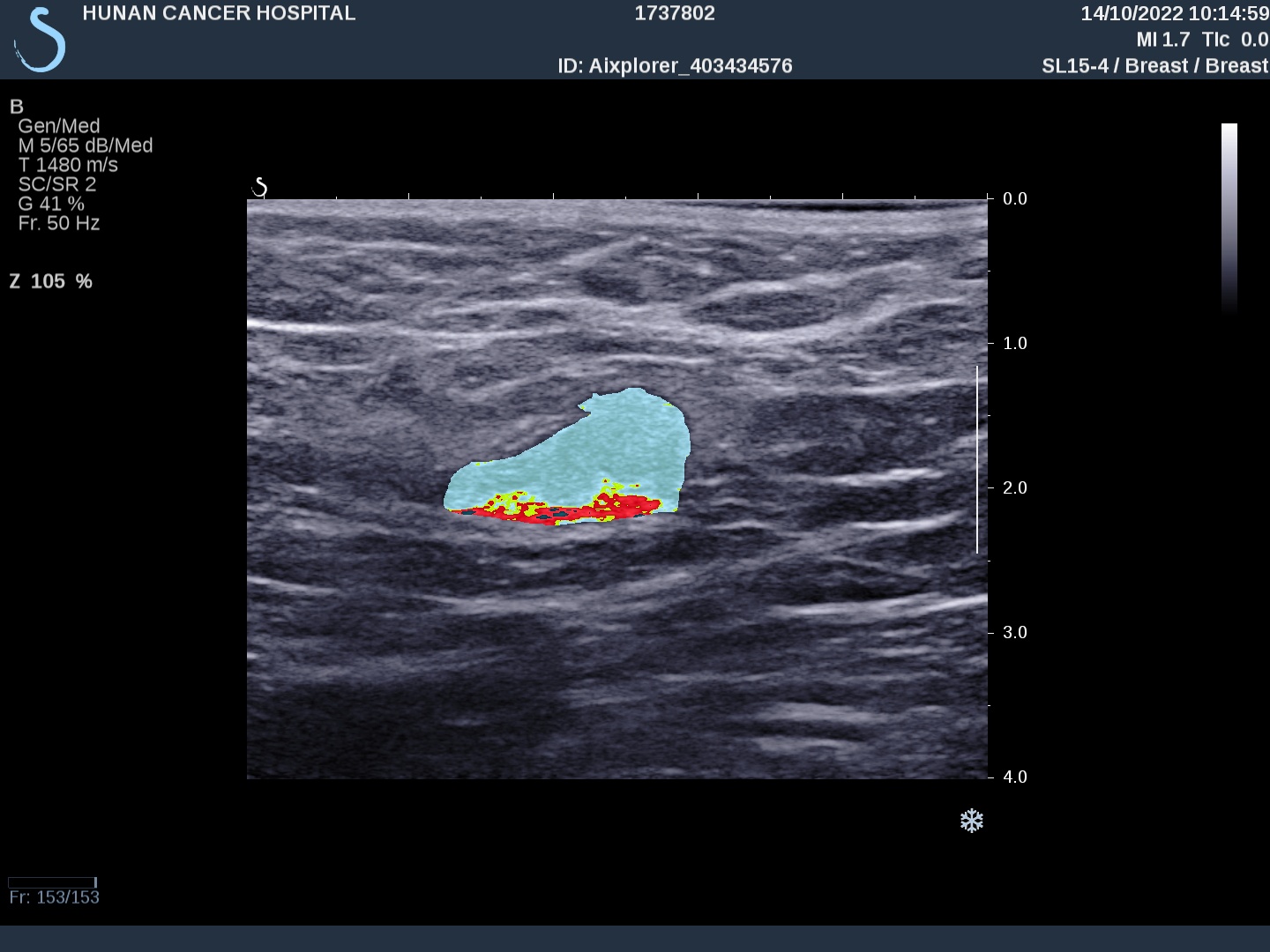

Supplement: Supplementary file 2 [file DataSheet_2.zip › ROI/1737802-1.jpg]

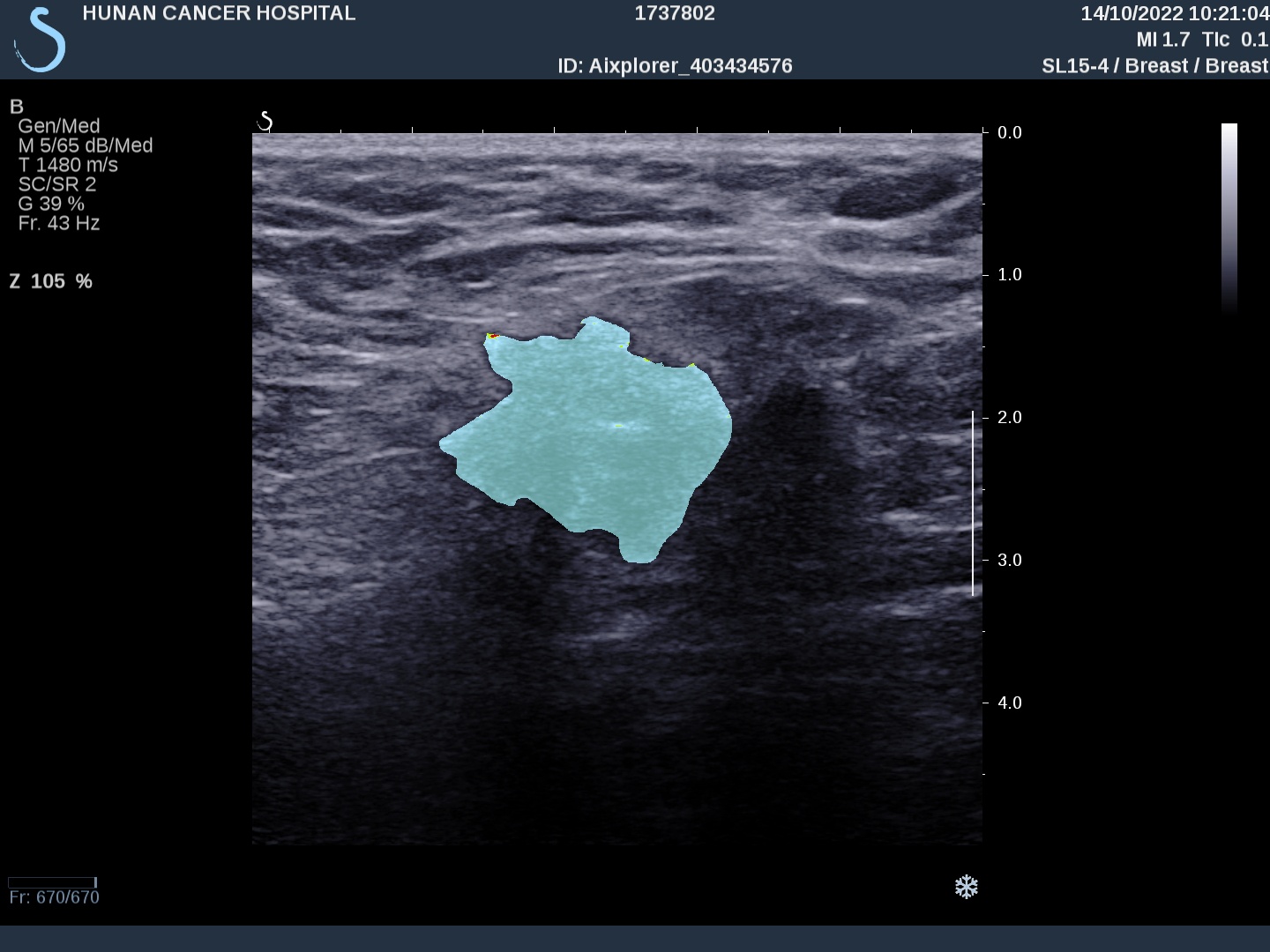

Supplement: Supplementary file 2 [file DataSheet_2.zip › ROI/1737802-7.jpg]

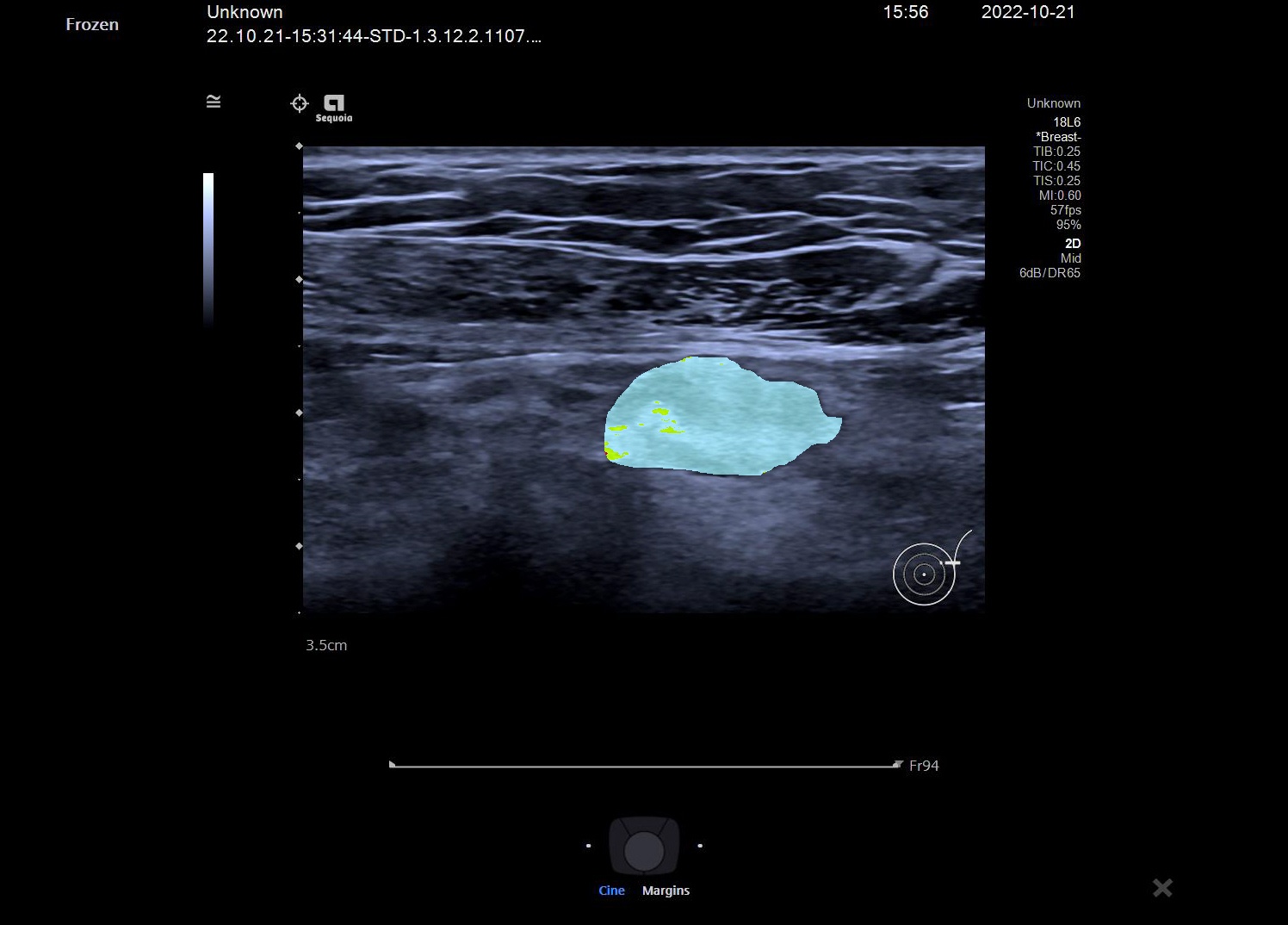

Supplement: Supplementary file 2 [file DataSheet_2.zip › ROI/1738048-2.jpg]

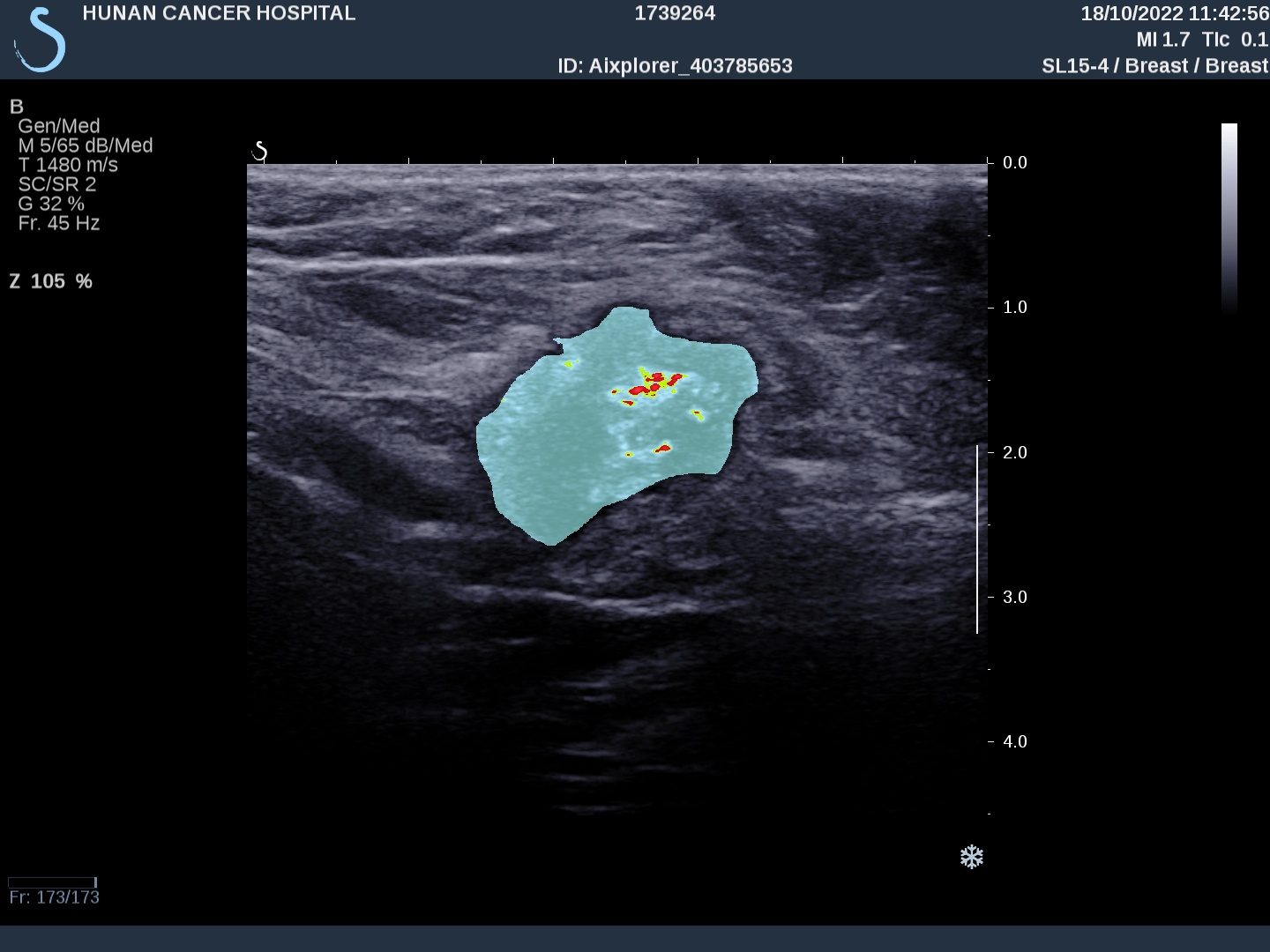

Supplement: Supplementary file 2 [file DataSheet_2.zip › ROI/1739264-3.jpg]
